# Connectivity Homology Enables Inter-Species Network Models of Synthetic Lethality

**DOI:** 10.1371/journal.pcbi.1004506

**Published:** 2015-10-09

**Authors:** Alexandra Jacunski, Scott J. Dixon, Nicholas P. Tatonetti

**Affiliations:** 1 Integrated Program in Cellular, Molecular, and Biomedical Studies, Columbia University, New York, New York, United States of America; 2 Department of Systems Biology, Columbia University, New York, New York, United States of America; 3 Department of Biological Sciences, Columbia University, New York, New York, United States of America; 4 Department of Biomedical Informatics, Columbia University, New York, New York, United States of America; 5 Department of Medicine, Columbia University, New York, New York, United States of America; University of California San Diego, UNITED STATES

## Abstract

Synthetic lethality is a genetic interaction wherein two otherwise nonessential genes cause cellular inviability when knocked out simultaneously. Drugs can mimic genetic knock-out effects; therefore, our understanding of promiscuous drugs, polypharmacology-related adverse drug reactions, and multi-drug therapies, especially cancer combination therapy, may be informed by a deeper understanding of synthetic lethality. However, the colossal experimental burden in humans necessitates *in silico* methods to guide the identification of synthetic lethal pairs. Here, we present SINaTRA (Species-INdependent TRAnslation), a network-based methodology that discovers genome-wide synthetic lethality in translation between species. SINaTRA uses connectivity homology, defined as biological connectivity patterns that persist across species, to identify synthetic lethal pairs. Importantly, our approach does not rely on genetic homology or structural and functional similarity, and it significantly outperforms models utilizing these data. We validate SINaTRA by predicting synthetic lethality in *S*. *pombe* using *S*. *cerevisiae* data, then identify over one million putative human synthetic lethal pairs to guide experimental approaches. We highlight the translational applications of our algorithm for drug discovery by identifying clusters of genes significantly enriched for single- and multi-drug cancer therapies.

## Author Summary

Synthetic lethality is a genetic interaction that has promising implications for informing novel cancer therapies. Over 200 million pairwise tests would be required to identify all pairwise synthetic lethal interactions in humans–currently, an impossibly large experimental burden. To simplify the process, we have developed a method to predict human synthetic lethal pairs in translation from a well-studied species to one in which synthetic lethality is understudied using both species’ protein-protein interaction networks. Here, we explore the model’s success in translation from *S*. *cerevisiae* to *S*. *pombe*. We then predict human synthetic lethality and suggest novel areas of inquiry for cancer therapies.

## Introduction

Synthetic lethality (SL) occurs when two nonessential genes cause cellular inviability after being knocked out simultaneously.[1] Although SL was originally studied and described in yeast, it can be a powerful tool for studying drug action in humans; for example, SL may guide the development of cancer combination therapy[2,3] and inform drug-drug interactions. SL interactions may differ between cellular contexts;[4] a gene pair that is SL in one cell type may not be SL in another. This can provide a tremendous therapeutic boon when two drugs targeting two gene products mimic an SL interaction in cancer cells and leave healthy cells unaffected. However, drug-induced SL interactions may also cause adverse events via unexpected cell death. Thus, mapping SL in humans is necessary to understanding mono- and polypharmacological effects.

Most gene pairs have not been interrogated for SL in humans, and several factors impede a species-wide evaluation of SL. These include the ethical implications of studying SL directly, the inability to discern state-specific SL interactions from global ones in experimental cell lines (*e*.*g*. cancer[4,5]), and–most significantly–the heavy experimental burden. Over 200 million assays would be required to determine the SL status of all human gene pairs in just a single cellular context. *In silico* methods are therefore necessary to guide the identification of SL in humans.

Previous work on leveraging model organisms to predict human SL has focused in particular on genetic homology, under the hypothesis that SL status will be maintained between orthologous gene pairs.[6] This approach has two major limitations. First, there are only ~2,000 genes that genetically homologous between *S*. *cerevisiae* and humans (NCBI Homologene[7]). These homologues account for a mere 1% of all possible human pairs, leaving the majority with no predictive data regarding SL status.

Second, genetic redundancies that developed independently in each species since deviation from a common ancestor may affect synthetic lethal status. For example, 228 gene duplication events have been suggested between *S*. *cerevisiae* and *S*. *pombe*[8]in the ~400 million years of evolution between the two species;[9] this number is likely even higher between *S*. *cerevisiae* and humans. Each of these events may introduce a functional redundancy that alters SL relationships in the organism by causing a gain or loss of SL. Focusing solely on genetic homology does not account for these complexities.

In this work, we first evaluate the performance of genetic homology in predicting SL. We also consider structural similarity using protein structure families, domain similarity using protein domains, and functional similarity with gene ontology annotations. We additionally consider information centrality, a univariate network-based model. We show that homology, structural similarity, and information centrality are limited in their ability to predict SL.

We then introduce the concept of *connectivity homology*, a measure of relatedness between genes that is independent of structure, function, or genetic homology. Relationships between genes and proteins, including redundancies, may be illustrated through the use of biological networks, and we hypothesize that the network connectivity profiles between two genes will better characterize their potential for an SL relationship. In order to compute these profiles, we use well-known graph properties, such as degree centrality and shortest path.[6,10–12] We next use machine learning and data from a well-studied organism, *S*. *cerevisiae*, to train a model of synthetic lethality that can be applied to any species of interest. We show that our algorithm, Species-INdependent TRAnslation (SINaTRA), significantly outperforms previously published models of predicting SL in translation. Importantly, we can predict synthetic lethality in species without any known (i.e. experimentally validated) synthetic lethal pairs. The only necessary information is an experimentally derived protein-protein interaction (PPI) network. We also show that the method is robust to network incompleteness.

We then use SINaTRA to predict SL in humans and assign each human gene pairs a score between 0 and 1, indicating the likelihood that the two genes exhibit a synthetic lethal relationship. As a post-processing step to enrich our predictions, we use databases of population genetic variation in humans to filter out likely false positives. Finally, we evaluate of the biomedical implications of our human SL gene pairs by discovering “hot zones” of putative SL pairs that suggest novel cancer combination therapies.

## Results

### Previous methods of modeling synthetic lethality: genetic homology, structural similarity, and functional similarity

We began our study by considering two published methods of predicting SL, protein homology[13] and bi-nodal information centrality,[8,14] and implemented the algorithms as described by the authors. In addition, we hypothesized that structural homology, domain homology, and functional homology may be able to predict SL and designed models based on these parameters for comparative analysis.

In Wu *et al*.,[9,13] the authors constructed a model to predict SL in *S*. *cerevisiae*, then hypothesized that human gene pairs homologous to SL pairs in *S*. *cerevisiae* would also be SL in humans. We implemented the latter part of the approach and evaluated it by predicting SL in *S*. *pombe*. By restricting our analysis to only genes that are homologous between *S*. *cerevisiae* and *S*. *pombe*, we find a significant predictive effect (OR = 145, 95% CI: 93–219, p < 2.2e-16, Fisher’s exact test), corresponding to an area under the receiver operating characteristic curve (AUC) of 0.60. Model performance decreased to OR = 45.9 (p<2.2e-16) and an AUC = 0.52 when expanding the model to include all gene pairs (Materials and Methods).

We next hypothesized that structural, domain, and functional similarity may be predictors of SL. We trained these models in *S*. *cerevisiae* and applied them to *S*. *pombe*. We used SCOP protein classifications to describe the former, and assigned each gene pair a value between 0 (no similarity) and 4 (same class) based on their products’ structural similarity. The model was trained and tested only on pairs with SCOP data associated with both genes. Only 399 SL pairs and 109,357 NSL pairs had SCOP data for *S*. *cerevisiae* (16,765,399 pairs skipped) and 2 SL/298 NSL for *S*. *pombe* (1,840,021 pairs skipped). The SCOP-based model had an AUC of 0.62. We additionally created a domain-based model from PFam[15] to predict SL. Domain data exists for a larger number of proteins (9,424SL/10,280,492 NSL in *S*. *cerevisiae;* 514/1,431,764 for *S*. *pombe*), allowing us to score more pairs than the SCOP-based model (Materials and Methods). The AUC in the domain-based model was 0.56.We described functional homology using annotations from Gene Ontology (GO) (Materials and Methods). Functional similarity attained an AUC of 0.81.

Finally, we calculated the pairwise information centrality[14] in *S*. *pombe* and found no significant predictive performance identifying SL pairs (AUC = 0.46, Logistic Regression). Bi-nodal information centrality did not require interspecies translation.

We hypothesized that multivariate, network-based models of synthetic lethality would be able to capture SL interactions both within and between species more successfully.

### Defining connectivity homology

We define two proteins as being *connectivity homologous* if they share similar connectivity profiles in their respective networks. A connectivity homologous relationship may exist between two proteins in the same species, or between proteins of different species. This concept can be generalized for pairs of proteins, or even groups of proteins (*i*.*e*. modules). For example, two pairs of proteins may be connectivity homologous because both pairs are connected to each other in a similar way.

**Fig 1:**
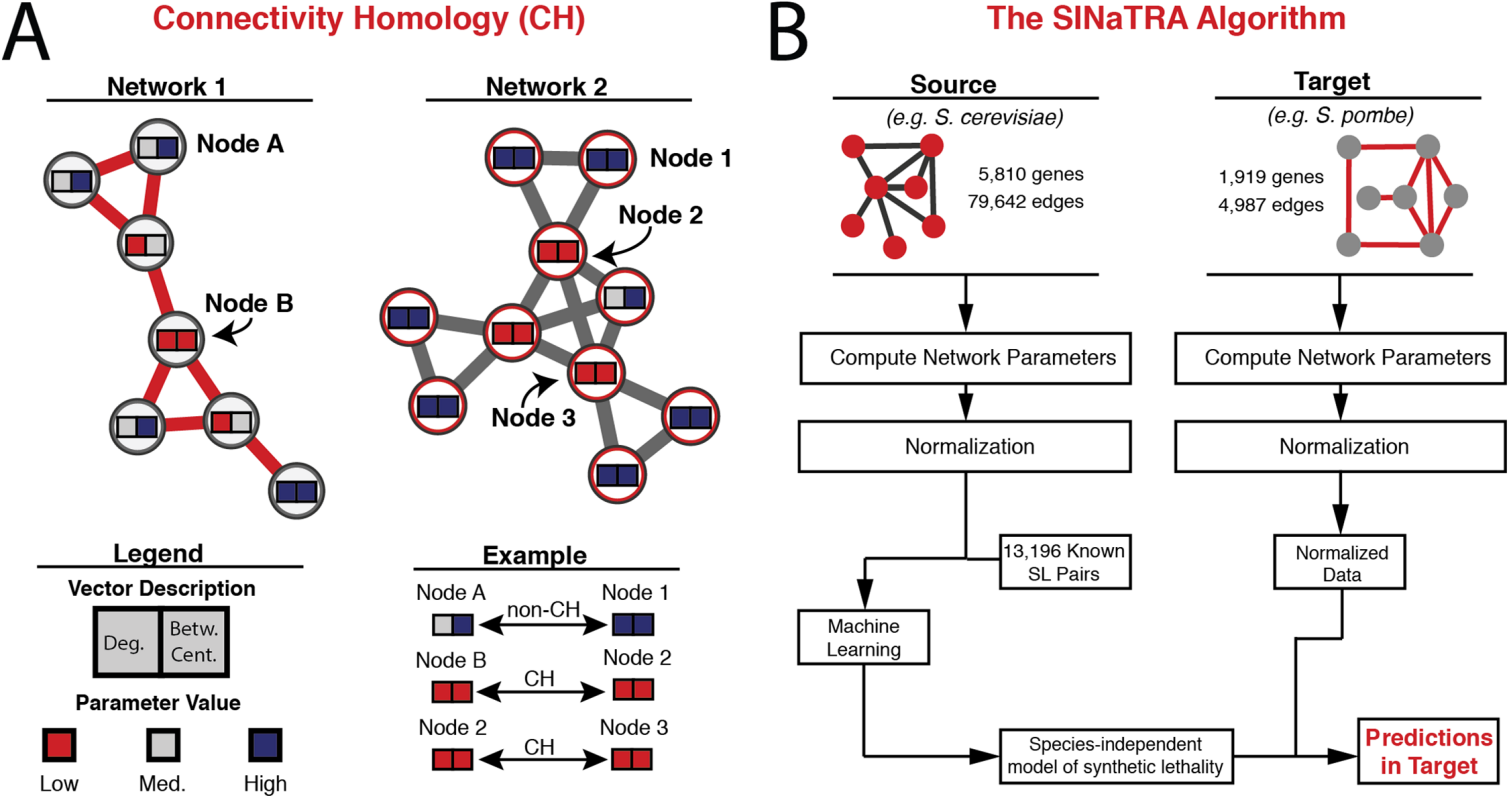
A) An illustration of connectivity homology (CH). Each node is described by two parameters (degree [deg.] and betweenness centrality [bet.cent.]) at three levels: low, medium, and high. Certain nodes have the same vectors (Node B/Node 2/Node 3); these nodes can be said to be *connectivity homologous* (CH). Other nodes do not (Node A/Node 1); these are non-connectivity homologous (non-CH). B) Schematic of the SINaTRA algorithm. We begin with the PPI networks of both our source and target species, calculate the network parameters (independently), and normalize the values of all parameters. Next, we use machine learning methods on the normalized network parameters of our source species as well as experimentally derived labels of synthetic lethality to construct a species-independent model of SL. Finally, we apply this model to the normalized network data of our target species in order to attain SL predictions in our target.

We illustrate this concept in Fig 1A, where we present two networks of different sizes and topologies. Two network parameters are used to describe the network: degree (*deg*.) and betweenness centrality (*bet*. *cent*.). Each node contains its connectivity profile as a vector depicting the degree and betweenness centrality as ‘low’ (blue), ‘medium’ (white), or ‘high’ (red). Although the values may not be immediately comparable between networks, it is obvious that certain nodes share similar connectivity profiles, while some nodes do not have an interspecies, connectivity homologous pair (high deg./medium bet.cent.).

### Connectivity homology can be evaluated with network parameters

In this paper, we represent connectivity profiles using vectors of network parameters. Each gene is represented by a vector of eight parameters (Tables 1 and S1). Each gene pair is represented by a vector of four node-pair parameters (Tables 1 and S1) as well as the individual profiles for each gene in the pair, leaving each pair with a connectivity profile defined by 20 network parameters. For the purposes of this investigation, we chose to use protein-protein interaction (PPI) networks because of the wide availability of data across many species. PPI data was downloaded from BioGRID[16] to construct graphs of one connected component (Materials and Methods). We computed the connectivity profiles for 5,810 proteins in *S*. *cerevisiae*, 1,919 in *S*. *pombe*, 4,233 in *M*. *musculus*, and 14,820 proteins in humans as well as for 16.8 million, 1.8 million, 8.9 million, and 109.8 million pairs of proteins for *S*. *cerevisiae*, *S*. *pombe*, *M*. *musculus*, and humans, respectively.

**Table 1:** Network parameters used in our model. A single-node network parameter provides two values to the feature vector per pair (8 single-node parameters create 16 values per pair). Each node-pair parameter contributes one value describing that pair. Parameter importance is measured using Gini importance[17,18] in the NetworkX Python package.[19]

Parameter	Context	Description	Parameter Importance
2^nd^ degree shared neighbors	Single node	The sum of all nodes two edges away from the node of interest.	0.036, 0.030
Betweenness centrality	Single node	The sum of the fraction of shortest paths between two other nodes passing through the node of interest.	0.056, 0.056
Closeness centrality	Single node	The inverse sum of all shortest paths that originate at the node of interest.	0.035, 0.032
Communicability	Node pair	The sum of all closed walks between a pair of nodes.	0.043
Current-flow betweenness centrality	Single node	Analogous to betweenness centrality, but with all paths instead of shortest paths. Also known as random walk betweenness centrality.	0.057, 0.045
Degree centrality	Single node	The fraction of edges a node has of all possible edges.	0.074, 0.055
Eccentricity	Sindle node	The maximum distance from the node of interest to any other node in the network.	0.038, 0.035
Eigenvector centrality	Single node	The eigenvector for the largest eigenvalue of the matrix adjacency network.	0.042, 0.034
Inverse shortest path	Node pair	The inverse of the smallest number of edges connecting two nodes of interest.	0.048
PageRank	Single node	The rank of graph’s nodes based on the number of incoming links.	0.080, 0.072
Shared neighbors	Node pair	The intersection of two nodes’ sets of immediate neighbors.	0.067
Shared non-neighbors	Node pair	The number of nodes that are not immediate neighbors of both nodes of interest.	0.063

We found that the distributions and ranges of network parameter values differed significantly between species (S1 Fig; S2 Table). To correct for these differences (S2 Fig), we evaluated four normalization strategies (Table 2) and chose to use rank normalization to rescale the values of each parameter between 0 and 1. Rank normalization makes parameter values comparable between species. We refer to normalized data as being “translated.”

**Table 2:** Methods of network parameter normalization.

Method	Description
Normalization	Each value is divided by the maximum occurring value of the parameter
Rank-normalization	Each value is ranked from smallest to largest, with tie breaks at random in case of equal values. These are then divided by the total number of values.
Tied-rank normalization	Each value is ranked from smallest to largest; entries with the same value are given the average of all their ranks. These are then divided by the total number of values.
Quantile normalization	Parameter values are collected for two or more conditions. Values are ranked for each condition. The values are then sorted, and each row is averaged. These values are then sorted back into order according to rank.

### Similarity between connectivity vectors is indicative of shared function

We found that proteins with similar connectivity profiles (*i*.*e*. those that are connectivity homologous) were more likely to share functional annotations. We used the Euclidean distance between connectivity profiles as a measure of connectivity homology (Materials and Methods). We compared this distance between genes that shared genetic homology (orthologs) and specific functional annotations (Gene Ontology [GO]) between *S*. *cerevisiae* and *S*. *pombe* (Sc/Sp) (S3A Fig) and between *S*. *cerevisiae* and humans (Sc/H) (S3B Fig). We found that proteins annotated with the same function had significantly had significantly lower distances (Sc/Sp median = 1.04, Sc/H median = 0.92) than those annotated with different functions (Sc/Sp median = 1.08, p<2.2e-16; Sc/H median = 1.04, p<2.2e-16).

This result holds even when orthologs are not considered. Non-orthologous genes annotated with the same function had significantly lower distances than non-orthologous genes annotated with different functions (S3 Fig, p<2.2e-16). We also found that orthologs had significantly lower distances than non-orthologous pairs (S3 Fig, p<2.2e-16). These differences were consistent across all levels of functional specificity (S4 Fig). These results suggest that network substructure, and therefore network signals, are conserved between species based on both homology and function.

### Building connectivity-homology-based models of synthetic lethality

#### Networks successfully predict within-species synthetic lethality

We used machine learning algorithms to build two models of synthetic lethality (SL) using the connectivity profiles we derived for pairs of proteins–one for *S*. *cerevisiae* and one for *S*. *pombe*. We trained these models using experimentally established SL gene pairs from BioGRID (N = 13,196 for *S*. *cerevisiae* and N = 628 for *S*. *pombe*) as our positive training examples. We randomly selected pairs not described as SL in the database as non-synthetic lethal (NSL) pairs, and used these as negative training examples. Our assumption that any pair without experimental evidence for synthetic lethality is NSL will be incorrect for a small number of pairs that are SL but have not yet been investigated; however, this will introduce negligible error due to the rarity of SL interactions (estimated 0.1% in dipoid organisms[15,20]).

We evaluated these models using cross-validation and area under the receiver operating characteristic curve (AUC). Random forest (RF) significantly outperformed logistic regression (LR) for both *S*. *cerevisiae* (AUC_RF_ = 0.92, AUC_LR_ = 0.77; p<2.2e-16, De Long’s Test) and *S*. *pombe* (AUC_RF_ = 0.93, AUC_LR_ = 0.86; p<2.2e-16, De Long’s Test) (Supplementary Materials, S5A Fig). We found that within-species model performance is consistent regardless of translation method (Supplementary Materials, S5B and S5C Fig).

#### Translation of synthetic lethality between *S*. *cerevisiae* and *S*. *pombe*

In order to create network models of synthetic lethality in translation, we developed the SINaTRA algorithm (Species INdependent TRAnslation). The schematic is illustrated in Fig 1B.

To apply SINaTRA to *S*. *cerevisiae* and *S*. *pombe*, we created two translational, network-based models that use data from a *source* species to infer the SL status of gene pairs in a *target* species. The first was trained on *S*. *cerevisiae* to predict SL in *S*. *pombe*; the second was trained on *S*. *pombe* to predict in *S*. *cerevisiae*. For each model, we randomly selected an equal number of NSL pairs as SL pairs (13,196 for *S*. *cerevisiae*; 628 for *S*. *pombe*). We built random forest models with 100 trees for each species. We evaluated these two models for their ability to predict SL gene pairs in the target species. Each model generates a SINaTRA score for each pair between 0 (predicted NSL) and 1 (predicted SL).

**Fig 2:**
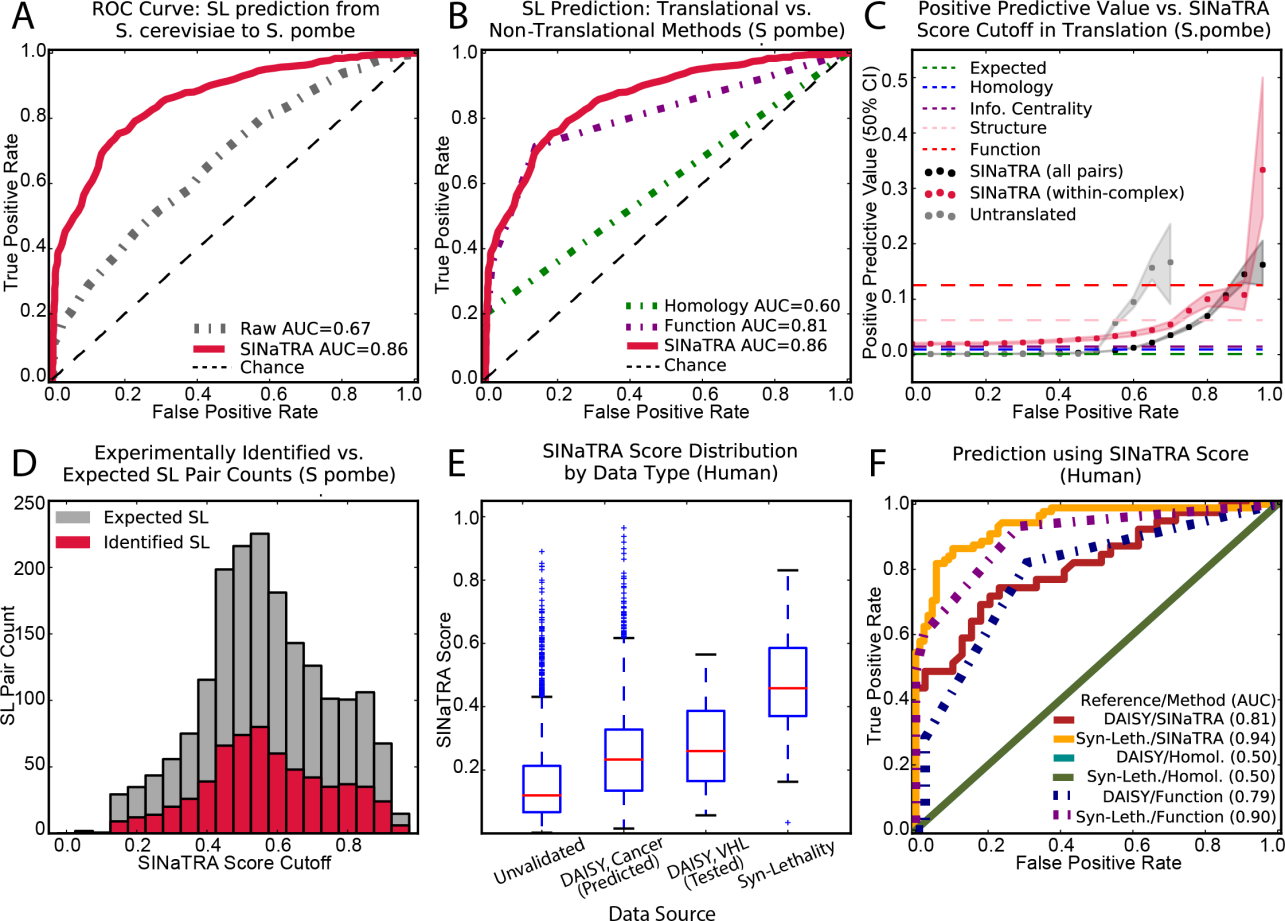
A. Receiver operating characteristic (ROC) curves for classification of SL/non-SL gene in *S*. *pombe* using *S*. *cerevisiae* as source. Comparison of untranslated (“raw”) parameters (gray, AUC = 0.67) and the translated parameters used in SINaTRA (red, AUC = 0.86). B. ROC curve of SL predictions using SINaTRA (AUC = 0.86) compared functional homology of gene pair products (AUC = 0.81) and gene homology (AUC = 0.60). The model based on gene homology was created using only gene pairs with homology data. C. Positive predictive value (PPV) of all (dark gray) and within-complex (red) gene pairs. When accounting for the expected ratio of SL:NSL (1:1000), a SINaTRA score threshold of 0.95 yields a median PPV of 17% (a 170-fold increase over what is expected by chance). At 0.85, the PPV drops to 7%. PPV increases in within-complex gene pairs, suggesting that this may be a good initial filter for experimental validation. D. At each SINaTRA score cutoff, we plot the number of experimentally identified SL pairs in that bin (red), as well as the number we expect to find at each level (gray). E. SINaTRA scores of all human predictions, as well as pairs predicted or found to be SL in two datasets: DAISY and Syn-Lethality. F. We compare the predictive ability of SINaTRA score to identify genes belonging to DAISY (tested) and Syn-Lethality datasets compared to functional similarity and homology.

Using *S*. *cerevisiae* as the source and *S*. *pombe* as the target, we found that untranslated parameters resulted in poor inter-species SL prediction (AUC = 0.67). We tested all methods of normalization in translation (S6A Fig) and found that the model significantly improves with any translational method with rank normalization performing best (AUC = 0.86; p<2.2e-16, De Long’s method) (Fig 2A). We also found that parameter normalization improved the precision from 50% to 98% at a recall rate of 30% (S6B Fig) in our testing data. The translated model also significantly outperforms the untranslated one when using *S*. *pombe* as the source species and *S*. *cerevisiae* as the target (AUC_translated_ = 0.74, AUC_raw_ = 0.67, p < 2.2.e-16, DeLong’s method, S7 Fig).

#### SINaTRA outperforms translation-free and non-network methods

After evaluating SINaTRA in *S*. *pombe* and *S*. *cerevisiae*, we compared its performance to those of models based on genetic homology and functional similarity. We show ROC curves of each previously discussed methods and compared it to that of SINaTRA (Fig 2B) and use the AUC as a summary performance statistic. We additionally compared the performance of SINaTRA to domain similarity, structural similarity, and information centrality (S8 Fig). We found that SINaTRA had significantly higher AUC than any other method we considered (p<2.2e-16, DeLong’s test, all comparisons).

We then estimated the PPV for all gene pairs at 20 SINaTRA score thresholds (Fig 2C); the ratio of SL:NSL pairs was held at the expected ratio (1:1000[20]). We found a significant improvement over chance (Odds ratio = 121.1, p = 2.72e-32, Fisher exact test). For example, at a SINaTRA score of 0.85, the PPV is approximately 7%—70 times higher than expected by chance (0.1%). It increases to 17% at a cutoff of 0.95, corresponding to a 170-fold increase. In comparison, the untranslated method of SL prediction rises to a PPV of 17% at a cutoff of 0.65 and dips sharply at 0.70. No gene pairs receive a score higher than 0.70 in the untranslated model.

We also found that no model out of genetic homology, functional similarity, structural similarity, or pairwise information centrality had a gene pair score higher than 0.05; therefore, we first identified which cutoff would provide the highest PPV, and plotted each value as dotted lines in Fig 2C. We also provide a direct comparison between true and false positives and negatives for SINaTRA compared to homology in S9 Fig. We found that, for all homologous pairs, the model achieves an OR of 144.9 (p<2.2e-16, Fisher’s exact test), corresponding to an AUC of 0.60. In contrast, SINaTRA achieves an OR of 929.6 (p<2.2e-16, Fisher’s exact test; S9 Fig) and a corresponding of AUC = 0.91 (S8 Fig) when using a SINaTRA cutoff of 0.85 on this same subset of pairs (any pair where at least one gene is not in the network is given a SINaTRA score of 0).

When we expand our data to the ‘whole genome,’ comprising all possible pairs from the set of Homologene and network genes (Materials and Methods), the homology-based method attains a lower, but significant, OR (OR = 60.1, p<2.2e-16) and an AUC of 0.52. A similar expansion in SINaTRA yields an OR of 304.2 (p<2.2e-16) when considering gene pairs with SINaTRA scores ≥ 0.85 as SL (S9 Fig).

We used Analysis of Variance (ANOVA) to evaluate the independent contributions of the methods when combined with SINaTRA. We found that genetic homology, protein similarity, and univariate connectivity contributed no significant improvement in performance over the SINaTRA-only model. This result held for genetic homology even when considering only the subset of ~2 million gene pairs that are homologous between *S*. *cerevisiae* and *S*. *pombe* (*Χ* ^2^ = 407.66, p = 0.64). Functional similarity (GO) significantly improved the SINaTRA model (*Χ* ^2^ = 445.09, p<2.2e-16, ANOVA) (S3 Table).

#### SINaTRA identifies missing SL in *S*. *pombe*

We estimated the number of previously unidentified synthetic lethal pairs at 20 SINaTRA thresholds (Fig 2D). For example, at a SINaTRA ≥ 0.85, we expect to find 177 SL pairs but only 65 have previously been experimentally identified. 1,759 gene pairs have a score of 0.85 or greater in S. pombe, corresponding to an expected hit rate of 1 in 15.

#### Synthetic lethality is enriched in protein complexes

We identified all within-complex gene pairs in *S*. *pombe* (N = 5,806, Materials and Methods) and found 46 experimentally identified SL pairs. We found that the positive predictive value (PPV) is consistently higher in within-complex pairs, reaching 0.27 at a cutoff of 0.95 (Odds ratio = 148.4, p = 1.33e-37, Fisher exact test).

#### Translated models are robust to network completeness

Species-specific PPI networks vary in their completeness. We can approximate completeness by using network density: the fraction of edges that exist in the network compared to the total possible number of edges.[21] *S*. *cerevisiae* has one of the most complete PPI networks (density = 0.04), while those of *S*. *pombe*, *M*. *musculus*, and *H*. *sapiens* are less complete, with densities of approximately 0.02, 0.01, and 0.01, respectively. We tested the extent to which SINaTRA was sensitive to these differences by ablating the target network (*S*. *pombe*) to densities between 90% and 50% of the original network (Materials and Methods). The lowest density approximates that of the human and mouse PPI networks. Untranslated parameters achieve AUCs between 0.43 and 0.60 for all ablated graphs. We found that ablation by 10% decreased rank-normalized AUC from 0.86 to 0.83, and ablation by 50% dropped the AUC to 0.79. (S10 Fig).

#### Prediction of synthetic lethality is not driven by node popularity

Higher degree nodes are more likely to be studied, and more popularly studied genes may be more likely to be synthetic lethal. As a measure of this potential bias, we defined a normalized popularity measure (degree/popularity), where popularity is the number of times a particular gene appears in the BioGrid database. As expected, SINaTRA score is correlated with degree and, thus, popularity. However, SINaTRA score is not correlated with normalized popularity in any of the three species (S11 Fig). Further, we found that the predictive performance of SINaTRA is independent of each of the three measures (degree, popularity, and node popularity) according to ANOVA (p < 0.0001 for all three comparisons).

#### Prediction of synthetic lethality in mice

We used the model trained on *S*. *cerevisiae* as the source species and *M*. *musculus* as the target. There is no comprehensive database of SL in mouse, and only nine mouse SL pairs are recorded in BioGrid. Of these, eight were predicted to be SL with a score ≥0.5; five had scores ≥0.70. SL prediction achieved an AUC of 0.937, significantly outperforming GO similarity (AUC = 0.687; p = 1.556e-11, DeLong’s method). Gene pairs with SINaTRA scores ≥0.8 are included in S4 Table.

### Human synthetic lethality

#### Prediction of synthetic lethality in humans

We applied the SL model trained on *S*. *cerevisiae* to human network parameters and generated a SINaTRA score between 0 and 1 for all human gene pairs; a higher score indicates greater evidence of SL according to the model. We next compiled a database of severe, tolerated, homozygous, deleterious co-mutations. These occur when at least one patient is homozygous for a deleterious mutation in both genes of a given pair in either of two datasets (1000 Genomes,[22] and Sweden-Schizophrenia Population-Based Case-Control Exome Sequencing (dbGaP accession: phs000473.v1.p1) [16,23–25]). We evaluated all gene pairs and found 450,010 pairs that match these criteria (0.4% of all possible pairs). We found that, on average, these gene pairs had significantly lower SINaTRA scores (median score = 0.116) versus all gene pair scores (median = 0.122; Mann Whitney U = 98,055,441,225.5, p ≤ 2.2e10-16). We then filtered these pairs from our predictions as false positives. Using a SINaTRA cutoff ≥0.85, we find the false discovery rate (FDR) from this filtering is 0.36% (61 false positives to 16,886 true positives).

We provide a filtered list of 1,309 predicted human SL pairs with SINaTRA scores >0.95 in S5 Table and provide the complete list of 109,358,780 gene pairs and SINaTRA scores as a database download.

#### Putative synthetic lethal pairs are more likely to be in the same pathway

Previous work has shown that SL pairs tend to be part of the same pathway.[1,19,26,27] We validated this in our predicted human SL pairs using KEGG annotations.[28] We found that gene pairs with SINaTRA scores ≥0.95, 0.90, and 0.80 were all significantly enriched for intra-pathway interactions compared to pairs selected at random (p<2.2e-16, Fisher’s exact test, all cutoffs). The ten highest-scoring gene pairs with the same pathway annotation are shown in Table 3.

**Table 3:** The top ten highest scoring within-pathway, putative SL gene pairs.

Gene 1	Gene 2	SINaTRA Score	Pathway Name
KYNU	SMS	0.990	Tryptophan metabolism
KYNU	GSR	0.987	Tryptophan metabolism
SOS1	BCR	0.986	MAPK signaling pathway
MSH3	PMS2	0.986	Mismatch repair
RCOR1	REST	0.985	Huntington’s disease
BIRC5	CASP9	0.985	Pathways in cancer
KYNU	NAGK	0.984	Tryptophan metabolism
POLR1B	POLR1A	0.980	Purine metabolism
RIPK1	RIPK3	0.980	Apoptosis
MAPK9	MAP2K7	0.980	MAPK signaling pathway

#### Protein complexes are significantly enriched for putative synthetic lethal pairs

**Fig 3:**
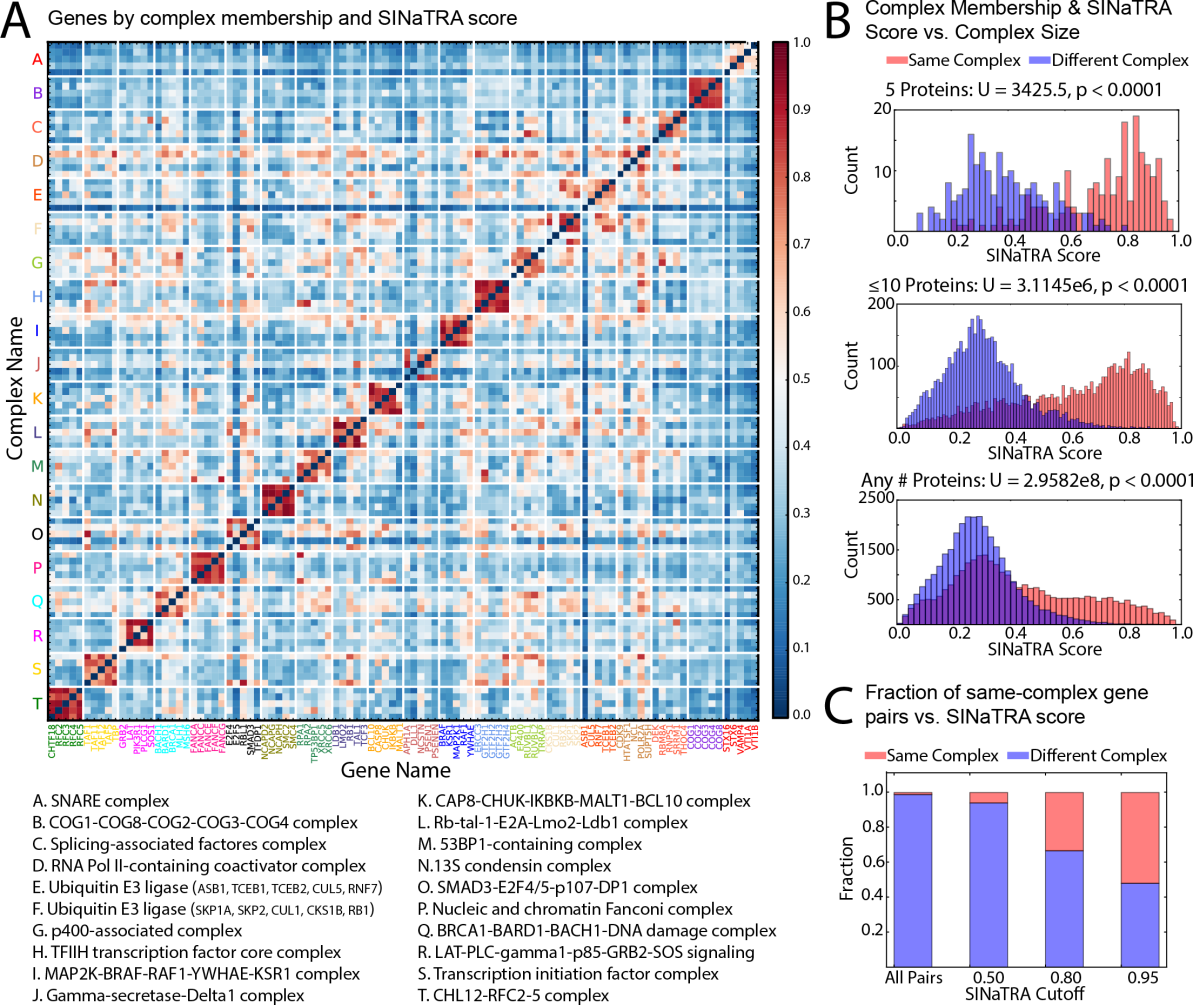
A. We randomly selected 20 mutually exclusive groups of protein complexes that contained exactly five subunits; we mapped the corresponding gene pairs to SINaTRA scores, and plotted a heat map of the results. Data are not clustered and only one randomly sampling was performed. We observed that within-complex gene pairs have significantly higher SINaTRA scores (p<0.0001, Fisher’s exact test). B. We compared the SINaTRA scores of gene pairs with products in the same vs. different complexes for complexes with of 5 protein subunits (top), ≤10 proteins (middle), and any number (bottom). Although proteins in the same complex are always enriched for higher SINaTRA score, as complex size increases, complex membership becomes less indicative of two genes being SL. C. We compared the fraction of gene pairs with products in the same vs. different complexes for three SINaTRA cutoffs (0.95, 0.80, 0.50) as well as for all gene pairs. A SINaTRA cutoff of 0.95 has approximately half of its pairs associated with the same complex; however, a decrease in the cutoff shifts this balance. This may indicate an increase in different mechanisms of SL in pairs with lower scores. “All Pairs” shows the expected proportion of in-complex pairs in our data.

A protein complex may be functional with one deleteriously mutated component, but present a lethal phenotype with two such mutations.[26] Our results corroborate this pattern. We randomly selected 20 sets of mutually exclusive protein complexes with five subunits from the Comprehensive Resources of Mammalian Protein Complexes (CORUM) [29] and plotted the SINaTRA scores of all the associated genes as a heat map (Fig 3A). We observed that genes with their products in the same protein complex had significantly higher SINaTRA scores (U = 3,425.5, p<2.2e-16; Fig 3B). Additionally, within-complex pairs were significantly enriched for higher SINaTRA scores for complexes of size ≤10 proteins (U = 3,114,511.5, p<0.0001), and complexes of all sizes (U = 295,820,010, p<0.0001). Finally, as the size of the complexes increases, the distributions of within-complex gene pair SINaTRA scores shifts to a left skew, echoing the distribution of gene pairs not in complexes. The proportion of gene pairs that have products in the same complex is significantly higher than expected by chance (p<0.0001, Fisher’s exact test, all SINaTRA score cutoffs) (Fig 3C).

#### Context-specific synthetic lethality

Synthetic lethality can change between contexts;[4] a gene pair that is SL in a cancer cell may not have the same property in healthy tissue. This may occur due to changes in protein expression, as well as activation or inactivation of protein pathways.

*S*. *cerevisiae* and *S*. *pombe* are unicellular organisms; therefore, models based on these species will necessarily focus on high-level, context-free synthetic lethal predictions. As such, the initial predictions from SINaTRA present all pairs that have synthetic lethal *potential* in their global connectivity patterns.

In order to explore context-specific SL pairs, we identified all human gene pairs with SINaTRA score ≥0.85. We next created tissue- and cell-line-specific lists of SL pairs by removing a gene pair if that tissue is not known to express both gene products according to the Human Protein Atlas.[21,30] The proportion of SL pairs retained after filtering is illustrated in S12A (tissue) and S12B Fig (cell); bars are color-coded by biological system. Although the number of proteins removed from the network is correlated with the number of SL pairs filtered from each given tissue or cell line (S12C and S12D Fig), we find that the number of filtered SL pairs is, at times, lower or higher than expected by chance (S6 Table) (Materials and Methods). For example, rectal tissue has approximately half as many SL pairs filtered out (70) as expected (146; OR = 0.477,p = 1.6e-5, Fisher’s exact test). In contrast, tissue of the small intestine has over twice as many SL pairs filtered (1653) than expected (826; OR = 2.11, p<2.2e-16, Fisher’s exact test). Respiratory epithelial cells also have a high number of filtered SL pairs (O: 550, E: 280; OR = 2.00,p<2.2e-16).

#### Comparisons with previously-published methods

Recent work on human SL includes the Syn-Lethality database,[31] which compiles experimentally identified human SL pairs, and the DAISY method,[5] a computational method of identifying SL pairs. We found that the gene pairs from both datasets had significantly higher SINaTRA scores (Syn-Lethality: U = 12,265, p<2.2e-16; DAISY (VHL): U = 299, p = 5.86e-6; DAISY (cancer): U = 1992856, p<2.2e-16; Fig 2E). Compared to the median of untested pairs (0.122; 99% CI: [0.122,0.122]), DAISY’s cancer predictions had a median score of 0.233 (99% CI: [0.225,0.243]); its VHL preditions had a median score of 0.255 (99% CI:[0.195,0.368]) and the Syn-Lethality dataset had a median score of 0.459 (99% CI: [0.397,0.514]).

From the Syn-Lethality database, we selected only SL gene pairs involving genetic deficiency, inactivation, or mutation. Of the 88 pairs matching these criteria, all were in our network, and we found 34 of these to have SINaTRA ≥0.5 (p = 4.8e-11, Fisher’s exact test), and 11 with SINaTRA≥0.75 (p = 0.0070, Fisher’s exact test). 2,816 gene pairs were predicted to be SL specifically in cancer using DAISY, 2,576 were in our network; we found that 151 had SINaTRA≥0.5 (p = 7.5e-24, Fisher’s exact test), and 14 had SINaTRA≥0.75 (p = 0.00096, Fisher’s exact test).

We observed that SINaTRA score was able to predict genes present in both the DAISY and Syn-Lethality datasets with AUCs of 0.73 and 0.93, respectively. (Fig 2F). In turn, homology was not at all predictive in either dataset (AUC = 0.50 for both; no homology data present for the pairs), unlike functional annotations (AUC = 0.786, DAISY; AUC = 0.904, Syn-Lethality). We then considered the precision-recall curves of these data and found that SINaTRA in both datasets outperformed function in DAISY, while function in Syn-Lethality had similar performance to that of SINaTRA (S13 Fig).

#### The landscape of human synthetic lethality

**Fig 4:**
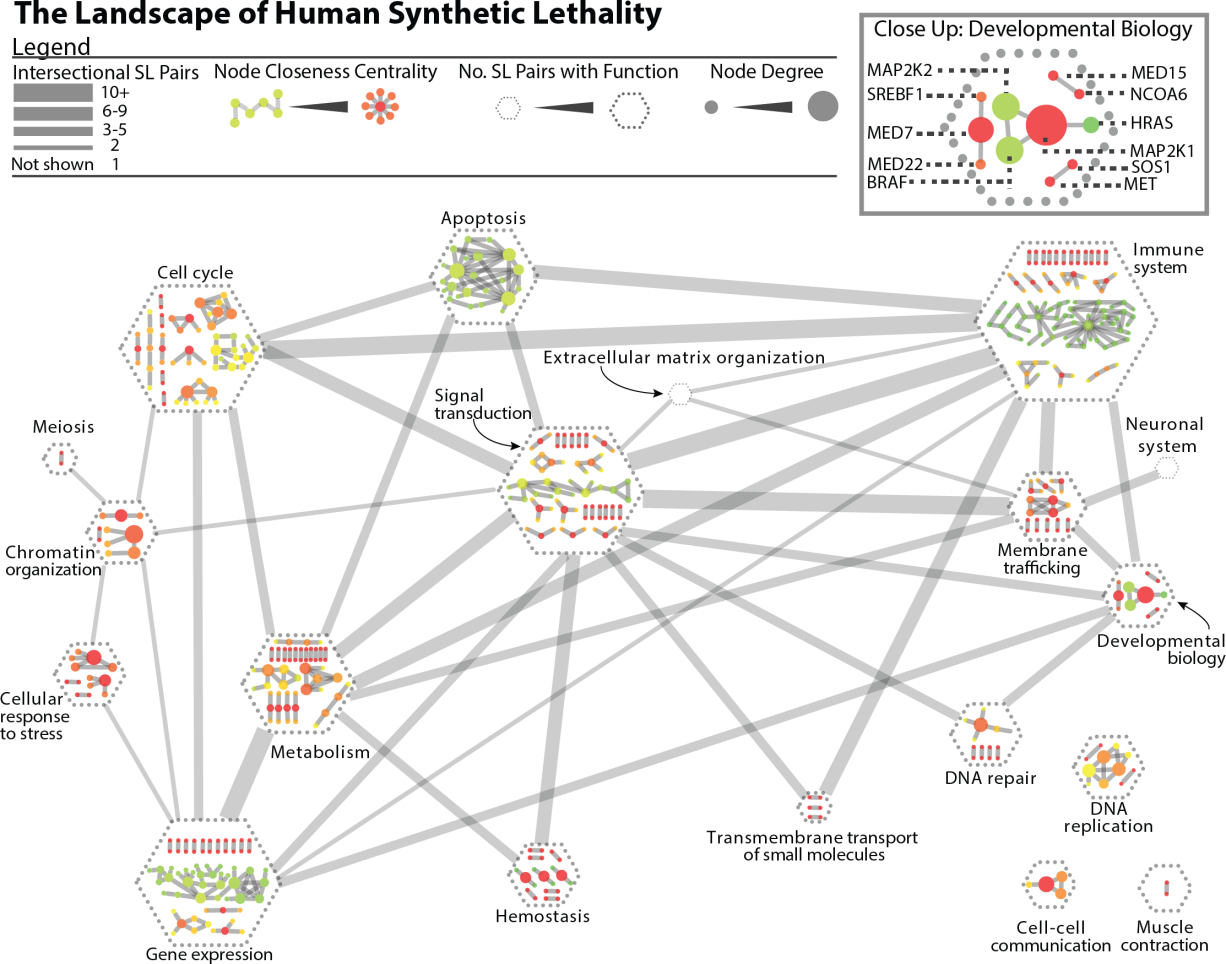
The landscape of human synthetic lethality. This network depicts all gene pairs with SINaTRA score ≥0.95 (1,229 SL pairs) that map to Reactome pathways (458 pairs). Here, each hexagon represents one high-level pathway designation in Reactome. Larger nodes indicate more SL pairs with that designation. Within the hexagonal nodes, we show the networks of synthetic lethality where both members have the same function in Reactome. Each node is a gene and an edge represents a predicted SL interaction. Gene nodes are weighted by degree and colored by closeness centrality. In turn, weighted edges join hexagonal nodes if pathway-divergent pairs exist; that is, one member of the pair is of one pathway while the second member is of the other. Edges are weighted by the number of pathway-divergent gene pairs associated with both pathways.

We categorized 458 predicted SL genes pairs using biological pathway data from Reactome[32] and present them as a network diagram (Fig 4), where hexagonal nodes represent pathways, and edges connect pathways when SL pairs are predicted between-pathway (i.e. with one member in each). We found that 334 (73%) of these interactions are within-pathway and 124 (27%) are between-pathway (OR = 3.69, p<0.0001, Fisher Exact Test).

Among the within-pathway SL pairs, we found that apoptosis, the immune system, and gene expression have highly interconnected SL networks, indicated by low closeness centrality. The immune system has the highest number of associated SL gene pairs (101); the most central of these is RIPK1, with 15 connections. Several functions have no associated SL pairs, including extracellular matrix organization, metabolism of proteins, and reproduction. These functions may have little functional redundancy that allows for SL to occur. Of the between-pathway SL pairs, we found that each pair of pathways share an average of 2.8 SL pairs. The immune system/signal transduction between-pathway pairs are the most numerous (11 pairs).

#### Function-specific mechanisms of synthetic lethality

**Fig 5:**
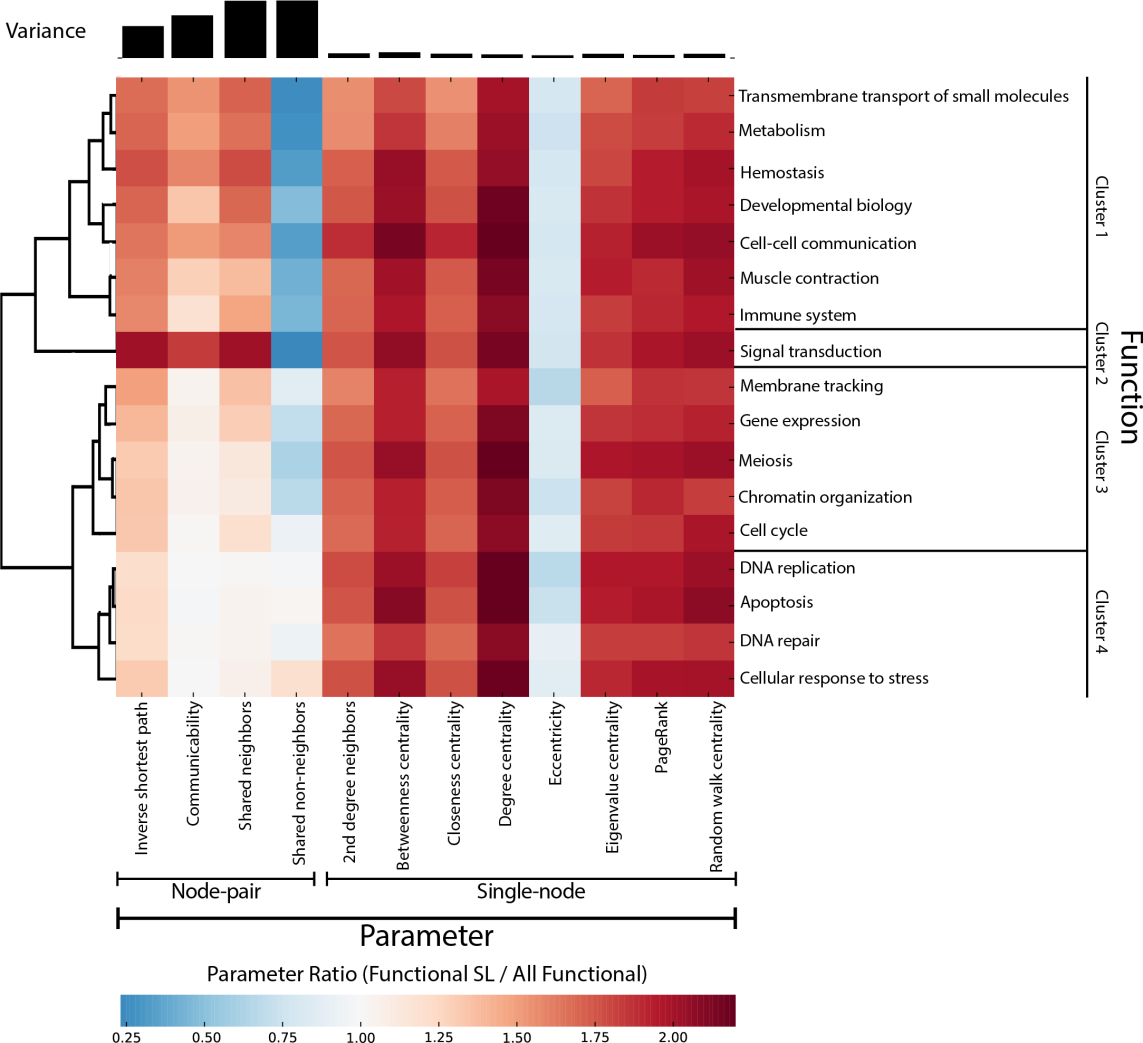
Function-specific patterns of synthetic lethality. The heatmap represents the ratio of median parameters for the SL pairs of a given function versus all pairs of a given function. For example, the SL pairs of Signal Transduction have two-times greater values for inverse shortest path than for the non-SL pairs of Signal Transduction. Rows are clustered by node-pair parameter values (see Table 1). Parameter variance is plotted above the heat map. Single-node parameters (see Table 1) are consistently altered in SL regardless of function. However, node-pair parameters differ between functions. This distinction suggests that network substructure may dictate SL mechanisms associated with a specific function.

We grouped gene pairs into 17 high-level Reactome functional categories and clustered them by their parameter values (Materials and Methods). We found pathway-specific parameter enrichment exists in node-pair parameters (inverse shortest path, communicability, shared neighbors, and shared non-neighbors), but not in single-node parameters, as evidenced by the increase in variance of paired parameters versus single-node parameters (Fig 5). For example, the signal transduction pathway has higher values for node-pair parameters than other functions and all SL pairs. In contrast, apoptosis, DNA repair, and DNA replication have node-pair signals that are closer to the mean of all of its within-function pairs than other functions.

We then annotated each putative SL gene pair from these 17 functional categories for three possible mechanisms: (1) complex, where the proteins products of the pair are known to form a complex, (2) parallel, where the proteins function in the same pathway with no known direct or indirect interaction, and (3) other, for gene pairs that do not fit in (1) or (2). In total there were 5,249 putative SL gene pairs for the 17 categories. Most of these pairs were in the same complex (56.2%, N = 2,950), followed by parallel (24.0%, N = 1,260) and other (19.8%, N = 1,039). We tested each function category for enrichments for particular mechanisms of SL. We found that each function has different proportions of putative mechanistic annotations (S14 Fig)

We found that immune system (OR = 1.48, p = 0.000001) and signal transduction (OR = 1.42, p = 0.000894) were significantly enriched for SL genes that function in parallel, after multiple hypothesis correction (Table 4). We found four categories were enriched for SL genes that were components in complexes: gene expression (OR = 1.38, p = 0.000298), meiosis (OR = 4.31, p = 0.046), chromatin organization (OR = 2.10, p = 0.008499), and DNA repair (OR = 4.76, p < 2.2e-16) (Table 4). Finally, we found that Cluster 1 (Fig 5), which includes transmembrane transport, metabolism, hemostasis, developmental biology, cell-cell communication, muscle contraction, and the immune system, is significantly enriched for SL genes that function in parallel (OR = 1.36, p = 0.00008).

**Table 4:** Within-function enrichment of putative SL pairs based on gene product interactions. *Complex* describes all gene pairs that are within the same pathway. *Other* represents all pairs that have another described PPI. *Parallel* refers to all pairs with no known PPI between them. Interactions are determined using Reactome data.

Function	Complex (Count/OR)	Other (Count/OR)	Parallel (Count/OR)	Cluster
**Transmembrane transport of small molecules**	52/***2*.*04*** *†*	8/*0*.*5*	12/*0*.*63*	
**Metabolism**	330/1.04	86/**0.68** **†**	162/**1.27** **†**	
**Hemostasis**	86/0.75	44/1.39	44/1.07	
**Developmental biology**	191/1.13	70/1.13	62/**0.74** **†**	*Cluster 1*
**Cell-cell communication**	20/1.2	8/1.3	5/0.56	
**Muscle contraction**	2/**0.22** **†**	5/**5.08** **†**	2/0.9	
**Immune system**	606/**0.64** *	286/**1.25** **†**	377/**1.48** *	
**Signal transduction**	352/**0.55** *	213/**1.58** *	239/**1.42** *	*Cluster 2*
**Membrane trafficking**	71**1.51** **†**	18/0.81	19/0.67	
**Gene expression**	572/**1.37** *	143/**0.71** **†**	199/0.86	
**Meiosis**	22/**4.31** **†**	3/0.53	1/**0.13** **†**	*Cluster 3*
**Chromatin organization**	77/**2.1** **†**	9/**0.37** **†**	20/0.73	
**Cell cycle**	124/**1.48** **†**	46/1.31	20/**0.36** *	
**DNA replication**	96/**1.54** **†**	6/**0.17** *	43/1.35	*Cluster 4*
**Apoptosis**	124/**1.48** **†**	46/1.31	20/**0.36** *	
**DNA repair**	124/**4.76** *	15/0.46	6/**0.13** *	
**Cellular responses to stress**	101/1.27	33/1.03	29/0.68	

*p<0.05, without multiplicity correction

†: p<0.001, with multiplicity correction

#### Putative synthetic lethal pairs suggest novel cancer therapies

**Fig 6:**
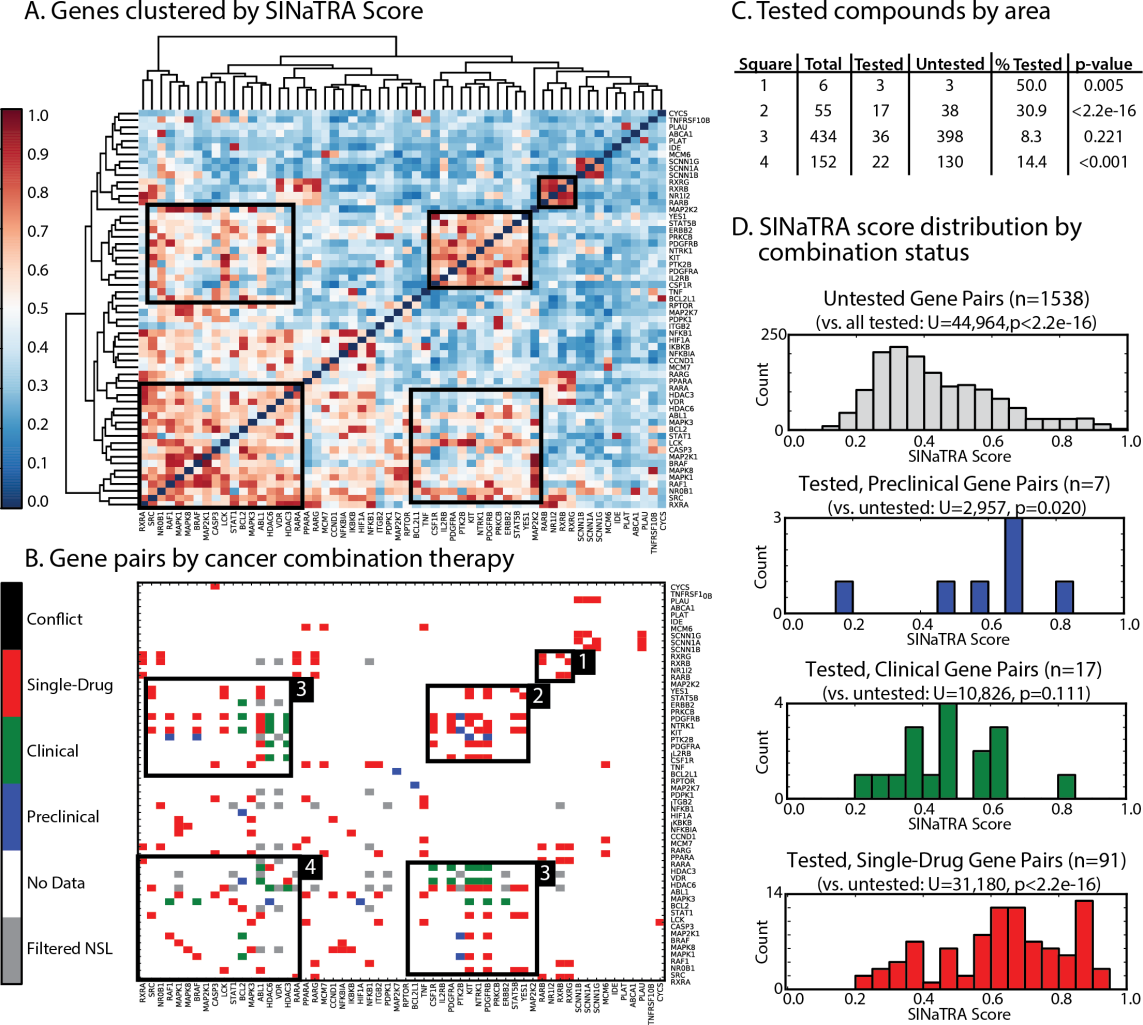
A. Druggable gene pairs clustered by SINaTRA score. Sixty-two unique genes that participated in predicted SL interactions with SINaTRA scores >0.85, where both genes mapped to drugs in DCDB, were identified. All pairwise SINaTRA scores were computed and clustered by score. Areas of high- and low SINaTRA scores are clearly visible. B. All possible gene pairs identified in Part A were mapped to DCDB, and gene pairs whose products are targeted by single drugs and combination therapies in the clinical pipeline were highlighted (pre-clinical, blue; clinical trials, green; single drug, red; gene pairs filtered out by genetic analysis, gray; filtered gene pairs associated with drugs, black [n = 0]). Areas enriched for drug combinations were highlighted in both parts A and B. C. Enrichment of tested compounds in the four areas of interest were calculated using the Fisher Exact Test, and p-values were calculated. Areas 1, 2 and 4 were significantly enriched. D. Distributions of SINaTRA score by drug type.

We identified 58 unique genes from high-scoring gene pairs (SINaTRA≥0.85) where both members were targets of cancer therapies (68 unique drugs). These genes were clustered by SINaTRA score (Fig 6A) using hierarchical clustering; areas of high (red) and low (blue) SINaTRA scores are easily be observed. We found that gene pairs that are targeted by drugs have significantly higher SINaTRA scores than those that are not; median SINaTRA score increases significantly from pairs that are targeted by only one drug (median score = 0.156), to those targeted by two drugs (median score = 0.166), to those targeted by only one cancer drug (median score = 0.211), to those targeted by two cancer drugs (median score = 0.283) (S15 Fig).

Next, we identified which of these gene pairs were filtered out through our co-mutation analysis (gray), as well as those linked to single-drug therapies (red), drug combination therapies in the clinical pipeline (blue: preclinical; green: in clinical trials). These data were overlaid on the heat map (Fig 6B). We found that gene pairs targeted by cancer drugs have significantly higher SINaTRA scores than filtered pairs and pairs not under investigation (Fig 6D; U = 44,964, p < 0.0001, Mann-Whitney U test).

We also visually identified “hotspots” of drug combinations (black boxes, Fig 6A and 6B) that correspond to gene pairs with high SINaTRA scores (Fig 6C). We found that Area 1 alone contains genes related to gene expression (p = 0.040), transcription initiation from RNA polymerase II promoter (p = 0.025), and steroid hormone receptor activity (p = 0.025; Fisher’s exact test with multiple hypothesis testing). In addition, Area 2 is associated with protein autophosphorylation (OR = 39.1, p = 0.000613; Fisher’s exact test). Areas 3 and 4 are not significantly associated with any GO terms.

## Discussion

In this paper, we present a computational method, Species INdependent TRAnslation (SINaTRA), for predicting synthetic lethal (SL) relationships in any species with an available protein-protein interaction (PPI) network. Our approach uses SL data from *S*. *cerevisiae*–the most well-characterized organism for this interaction–to train a statistical model that identifies network connectivity profiles indicative of synthetic lethality. Once trained, the model can be applied to any other species for which PPI data exist. The model takes the PPI network data as its input, and returns a probabilistic score between 0 and 1 for every pair that we deem the SINaTRA score. These scores represent the likelihood of an SL relationship between those two genes.

We validated our method by predicting which pairs are likely to be SL in *S*. *pombe*, another species for which a large number of SL pairs are known. Our approach significantly ourperforms others we tested. Most notably, our method does not rely on any knowledge of gene structure, sequence, or function; instead, it uses only the connectivity patterns exhibited by synthetic lethal pairs of genes as they appear in a protein-protein interaction network. Future work will focus on the integration of other sources of knowledge with the goal of improving predictive performance and understanding the role of connectivity under different functional conditions.

### Previous interspecies methods of predicting synthetic lethality

Previous work on interspecies SL prediction has focused on the use of genetic homology.[33] We found that the method has fairly high predictive power between *S*. *cerevisiae* and *S*. *pombe* when considering only gene pairs with known homology (Fig 2B). Unfortunately, many genes have no known homology information and, because of this, the model performance suffers when considering all interspecies gene pairs. An additional complication stems from genes with multiple homologues, resulting in ambiguous predictions. In an effort to address some of these challenges with using established orthologs, we also implemented two additional methods: one using shared structural domains, and one derived from structural families. Neither method outperformed SINaTRA. The most successful comparison method was the number of shared functional annotations in the Gene Ontology (AUC = 0.81), which performed almost as well as SINaTRA (AUC = 0.86). We additionally found that the information contained in the functional annotations and SINaTRA was not redundant, suggesting that a model that combines connectivity profiles with functional annotations may yield better performance.

### Connectivity homology as a novel method for predicting synthetic lethality

In this paper, we introduce the idea of *connectivity homology*, which exists when two genes share a similarity connectivity patterns quantified by network and graph theoretic parameters. We performed a small exploration of connectivity homology and its relation to genetic homology and function, and found that homologous genes and genes that share function exhibit higher connectivity homology (S3 Fig).

We hypothesized that there are connectivity patterns between pairs of genes that are indicative of a synthetic lethal relationship. These patterns are discovered using supervised machine learning in a *source* species–one where synthetic lethality has been well-characterized–and then identify these patterns in a *target* species to predict synthetic lethal pairs of genes. We performed a small exploration of connectivity homology and its relation to genetic homology and function, and found that homologous genes exhibit higher connectivity homology; in turn, interspecies gene pairs that share the same specific function have higher connectivity homology than interspecies gene pairs of different functions (S3 Fig).

We validated our approach in two species where SL has been experimentally explored (*S*. *cerevisiae* and *S*. *pombe*). We found that our approach, called SINaTRA, significantly outperformed published methods at predicting SL genes in the target species and we achieve precision up to 150 times higher than expected by chance. This precision increased to over 250 times higher than chance when using additional biological priors.

### Possible mechanisms of synthetic lethality

Several mechanisms of synthetic lethality have previously been proposed;[34] these include within complex, parallel pathways, and essential linear pathways. Hints regarding the mechanism driving a given gene pair may be provided by our connectivity parameters. Our data suggest that function-specific network substructures are different, and may be related to trends of SL mechanism within a function. For example, metabolism has a much higher proportion of ‘unknown’ pathway annotations than does apoptosis (S13 Fig). This suggests that putative metabolic SL pairs act through parallel pathways, while apoptotic pairs may act through within-complex mechanisms. Further, gene pairs in apoptotic pathways are farther apart and have lower communicability than gene pairs in metabolic pathways, which may also change how many SL pairs are likely to exist with that function.

We also observe that a fraction of the predicted SL pairs had between-pathway interactions, where members of an SL pair do not share any single function (Fig 4). The respective gene products may act at an interface between two related functions; the putative SL pair may be a false positive; or–most interestingly–one (or both) genes have previously unidentified functions that cause their SL behavior. One such example is the putative SL pair, BAIAP2 (insulin receptor signaling; UniProt DB) and ALDH7A1 (protection from oxidative stress; UniProt DB) (SINaTRA score: 0.957). Oxidative stress is associated with insulin resistance,[35] and knocking out both of these genes may mimic or exacerbate insulin resistance, leading to complications and adverse events.

### False positive rate in predictions of synthetic lethality

For very rare biological phenomena, it is essential to consider the false positive rate of any experimental or computational approach. An unbiased random selection of gene pairs would yield approximately 1 synthetic lethal pair for every 1,000 tested. If biased by biological priors, such as limiting the analysis to pairs of genes whose products are partners in protein complexes, this yield may increase 8-fold, to 1 out of every 125 pairs tested.

The SINaTRA score we present can also be used as a biological prior. In this case, it is the connectivity pattern of the pair of proteins that makes them more likely to participate in a synthetic lethal interaction. For example, a score of 0.85 or greater would yield approximately 1 SL for every 10 pairs tested. If this is coupled with protein complex prior, this could improve to 1 out of every 3 or 4 pairs tested. Combined with other biological priors, the SINaTRA score can be a powerful tool for directing experimental exploration of synthetic lethality. Fig 2D illustrates this expected hit rate versus the number of experiments that would be necessary. These scores can be used to guide experimental exploration depending on the throughput and cost of the experimental approach.

### Context-specific synthetic lethality

Biological contexts, such as tissue type and disease state, can influence synthetic lethal interactions.[4] At this time, cellular and tissue specificity are not captured by the SINaTRA model. However, we can customize our predictions for a given cell or tissue by pruning away any predicted genes that are known not to be expressed in the given context. We used the Protein Atlas[36] to perform this customization and found that certain tissues and cell types had significantly more or fewer SL pairs filtered. These deviations may suggest tissue or cell types that are particularly robust, or susceptible, to SL interactions. For example, respiratory epithelial cells and endothelial cells have many more SL pairs filtered out than expected by chance; this suggests that the tissues are not as susceptible to SL reactions–a hypothesis that requires further investigation.

### Predicted synthetic lethal pairs in humans inform cancer polypharmacology

For nearly a decade, leveraging synthetic lethal relationships specific to cancer cells has been a strategy in drug discovery. Therefore, we applied our predictions of synthetic lethality to the study of pharmacology. We found that many cancer combination therapies currently in the clinical pipeline target genes with high SINaTRA scores, suggesting that they use mechanisms of synthetic lethality as their modes of action. Clustering reveals hotspots of high SINaTRA scores that are significantly enriched for combination therapies under investigation. Importantly, our algorithm was able to identify these without any *a priori* knowledge of the drug combination. Gene pairs found in these hotspots that have not been previously investigated may be promising leads for novel polyphamacological treatments.

### Limitations

Our method for predicting SL relies on the availability of protein-protein interaction data. Due to the high-throughput experimental techniques, such as tandem affinity purification and yeast two-hybrid, these are some of the most widely available–omic data. However, comprehensive networks are only available for a handful of species. Future expansions of the approach will focus on integrating other available data, such as genetic sequence or gene expression. These other data sources may help address the issue of context-specificity in our predictions.

In this study, we used 12 distinct graph theoretic parameters to describe each gene pair. The choice of these parameters was based on what was available and has been used in prior work, and is not an exhaustive list. Other methods for computing connectivity may be incorporated in future versions of the algorithm, such as spectral methods.

### Conclusion

In summary, the methodology presented in this paper can help to inform a wide variety of studies in human health by fully utilizing information gathered in model species. In particular, the differential mechanistic analysis that highlights how biological functions may be targeted using synthetic lethality and the “hot spots” of drug synergy highlighted by our cancer therapy analysis indicate promising areas for novel therapeutics. We provide the SINaTRA scores for almost 110 million human gene pairs as a freely available resource for basic and translational science.

## Materials and Methods

### Previous methods of modeling synthetic lethality: genetic homology, structural similarity, and functional similarity

We downloaded protein homology data from Homologene,[37] protein structure data from SCOP, [29,38,39] and GO data from Entrez.[4,40,41] We used PFam[15,42] data for protein domain similarity; IDs were mapped to Entrez gene IDs for *S*. *cerevisiae* and *S*. *pombe* using DAVID.[32,33,43,44] We calculated binodal information centrality for each gene pair based on Kranthi *et al*.[14]

In order to create the homology-based model, we replicated a previous paper[45] that defined a gene pair as SL if its homologous pair in another species is SL. Gene pairs were defined as SL if the homologous pair in the source species was SL. In the case of multiple homologous pairs in the source species, gene pairs described by the fraction of homologous pairs defined as SL. A pair score >0 resulted in a classification of SL. Homology-based models use only genes with known homologs between the two species of interest. Whole-genome, homology-based models are the union of all genes in the homologous dataset with all genes that appear in our protein-protein interaction network. Genes with no known homologs are given a feature value of 0.

Protein similarity was defined using values between 0 (no match) and 4 (same class) according to SCOP annotations. Functional similarity was defined using GO process and function terms, excluding “molecular_function” and “biological_process.” Gene pairs were assigned a value based on the number of overlapping GO terms assigned to each gene. Using PFam domain data, we used the size of PFam ID overlap (range: [0,8)) for within-species gene pairs. For SCOP-, GO-, and PFam-based models, we trained the logistic regression model on *S*. *cerevisiae* and applied it to *S*. *pombe*. The homology-based model was already “translated,” and the model was trained and tested in *S*. *pombe* alone using logistic regression and five-fold cross-validation. Information centrality does not require translation and was calculated in *S*. *pombe* alone; the model was constructed using logistic regression and tested with five-fold cross-validation.

### Defining connectivity homology

Fig 1A was drawn with, and network parameters of each network were calculated using Cytoscape.[46]

### Calculation of translated network parameters

Regular normalization of a parameter returns each value divided by the maximum value of that parameter, such that each value is between 0 and 1. To rank-normalize data for a given species, we calculated all individual single- and two-node parameters. Then, for each parameter, we ranked all calculated values from smallest to largest, resolving ties at random. We then divided all values by the total number of genes in the network (for single-node parameters) or the total number of gene pairs (for node-pair parameters). This resulted in all genes or gene pairs having all parameter values be a value between 0 and 1. Tied-rank normalization assigns the median rank to all equal values, then normalizes single-node parameters by the number of genes in the network, and node-pair parameters by the total number of pairs. Quantile normalization is described in previous work,[47] where networks with fewer nodes/edges are up-sampled. Rank-normalized translation and construction of models is illustrated in S16 Fig.

### Similarity between connectivity vectors is indicative of shared function

We defined a vector of single-node network parameters (see Table 1) for each gene in the *S*. *cerevisiae*, *S*. *pombe*, and human networks. We calculated the connectivity homology of each interspecies node pair using Euclidean distance. A lower distance implies greater connectivity homology (similarity).

We first divided all gene pairs into same specific function or different specific function. We then further divided these groups into homologous/non-homologous. Specific functions were defined as all GO terms related to process or function (excluding molecular_function or biological_process) where the number of genes annotated with that GO in each species was less than or equal to a given cutoff. This cutoff was set to 100 at first, then expanded to cutoffs of 10, 15, 20, 25, 50, 75, 100, 150, 200, 250, 500, and 750.

### Building connectivity homology methods of synthetic lethality

We generated PPI networks using data gathered from BioGrid;[16,36] each node represents a gene, while edges represent a physical interaction between gene protein products. We pruned all networks to contain one connected component.

BioGrid additionally provided SL data used in this investigation. *Saccharomyces cerevisiae* had over 14,000 unique SL pairs and *Schizosaccharomyces pombe* have over 700, while *Mus musculus* and *Homo sapiens* have 14 and 1 pairs, respectively. Gene pairs may have one of two classes: SL or non-SL. Because of the scarcity of SL pairs, pairs not explicitly labeled as SL are considered non-SL.

We used the NetworkX (version 1.8.1) package for Python[19,37] to calculate all network parameters except shared neighbors, shared non-neighbors, and shared 2nd-degree neighbors, which were elucidated from adjacency matrices for each network. All single-parameter classifiers employ logistic regression due to its high interpretability and simple nature. We implemented multi-parameter classifiers using random forests,[17] which are accurate and efficient on large datasets, as well as resistant to over-fitting data. We used five-fold cross-validation in classifier construction, where training occurs with 80% of the data, and classifier evaluation uses the remaining 20%. Finally, to avoid positional bias in case of a single node having exceptionally high values, we shuffled the order in which each single-node parameter appears. We calculated parameter importance using the built-in function from Python’s sklearn package.

### Networks successfully predict within-species synthetic lethality

We predicted SL within a species using the network parameters defined in Table 1 without any normalization (raw) as the features of the classifier, and experimental data from BioGrid[16] as the known classes. From these, we performed five-fold cross-validation by randomly selecting 1/5 of the data on which to train our classifier, and testing it on the remaining 4/5. We trained models using logistic regression or random forest.

### Translation of synthetic lethality between *S*. *cerevisiae* and *S*. *pombe*

To predict synthetic lethality, we trained classifiers on raw and translated parameters of our source species, using SL status downloaded from BioGrid as labels. We then applied the classifier to data from our target species. Here, *S*. *cerevisiae* is the source species, and we used its network parameters to train classifiers. *S*. *pombe* is the target species. Classifier inputs were vectors of network parameters.

### SINaTRA outperforms translation-free and non-network methods

Synthetic lethality is expected to occur in 1/1000 gene pairs in diploid organisms; therefore, the PPV expected by chance is 0.001. We calculated positive predictive value (PPV), the fraction of true positives out of all called positives, on all *S*. *pombe* gene pairs, and on all gene pairs in the same complex. We selected 1000X the number of NSL pairs as SL pairs and bootstrapped the 99% CI of the PPV for both untranslated and SINaTRA-based predictions. To calculate PPV at each cutoff C, gene pairs with SINaTRA ≥ C were considered to be SL, while pairs with SINaTRA < C were considered NSL.

Complex membership was identified by using the Entrez GO database, and filtering all GO terms that contained the word “complex” and were in the “component” category. This amounted to 8,365 pairs, of which 5,806 appeared in our network. 46 of these were experimentally known SL pairs, leaving a ration of approximately 3:400 SL:NSL. We estimated that, because many SL pairs are unknown in *S*. *pombe*, the ratio of SL:NSL in within-complex pairs will be approximately 1:50, and selected SL:NSL pairs in a ratio of 1:50 in order to estimate within-complex PPV. This simulation was performed 1,000 times to identify the 99^th^ percentile CI.

We additionally plotted the PPV of SL prediction using genetic homology, structural similarity, functional similarity, and information centrality. The expected PPV of all of these were calculated using SL:NSL gene pairs in ratios of 1:1000; because the cutoffs occurred in a range significantly smaller than [0,1], we selected the cutoff that would provide the optimal PPV for the given model (all pairs), then calculated the PPV when adjusting for SL:NSL ratio. The PPV of genetic homology was calculated using only *S*. *pombe* pairs that have homologs in *S*. *cerevisiae*.

We identified the true and false positives and negatives for homology and whole-genome homology as follows: if the input score was >0 and the target species pair was SL, it was a true positive; else it was a false positive. If the input score was 0 and the target species was NSL, it was a true negative; else, it was a false negative. In whole-genome models, all node pairs with no homology information for at least one node were given a score of 0. Odds ratios were calculated using confusion matrices of form [[TP,FP],[FN,TN]] and Fisher’s exact test.

For whole-genome SINaTRA methods, if the gene pair SINaTRA score ≥ given cutoff and the target species pair was SL, it was a true positive; else, it was a false positive. If the gene pair SINaTRA score < given cutoff and the target species pair was NSL, it was a true negative; else, it was a true positive. In a whole-genome SINaTRA model, nodes that appeared in the Homologene database but not in the network were assigned SINaTRA scores of 0.

We identified the expected number of unidentified SL pairs in *S*. *pombe* by taking the PPV at each SINaTRA cutoff and multiplying it by the number of putative hits at that cutoff. We then transformed this cumulative plot into bins, such that for cutoff C, the number in that bin represents all expected pairs with C ≤ SINaTRA < C+0.05.

### Translated models are robust to network completeness

We ablated the *S*. *pombe* network to 90, 80, 70, 60, and 50% of its original side by removing (100-N)% edges at random. We trained a random forest classifier on the complete *S*. *cerevisiae* network and tested it on the ablated *S*. *pombe* networks and measured classifier success again using AUROC.

### Prediction of synthetic lethality is not driven by node popularity

We plotted the median SINaTRA score of genes in *S*. *cerevisiae*, *S*. *pombe*, and humans by the node’s degree, popularity (the number of times it appeared in the BioGRID database), and normalized popularity (degree/popularity). We calculated the Spearman correlation coefficient for all plots, for all species.

### Prediction of synthetic lethality in mice

We predicted SL pairs in mice as we did with *S*. *pombe*, using *S*. *cerevisiae* as the source species.

### Human synthetic lethality

#### Predictions of synthetic lethality in humans

After establishing the success of parameter translation, we applied the rank-normalized inter-species classifier to human and mouse gene pairs.

In order to filter human predictions for false positives, we annotated the VCF files from two studies for patients homozygous for significantly deleterious mutations (high impact, resulting in nonsense mutation, early stop, or loss of start). We then identified gene pairs where both genes were simultaneously significantly deleteriously mutated in at least 1 patient but no more than 5% of patients in one study, and filtered these out as confirmed NSL pairs (N = 405,010).

We compared the SINaTRA scores of the ‘confirmed NSL’ pairs to all SINaTRA scores by randomly selecting an equal number of the remaining pairs and applying the Mann-Whitney U test.

We chose high-confidence SL predictions to be those which our classifier assigned SL-scores of >0.95 that were not filtered out by our genetic screen.

#### Putative synthetic lethal pairs are more likely to be in the same pathway

We identified all putative SL pairs with SINaTRA scores >0.95, 0.90, and 0.80; these groups consisted of 1,224, 6,366, and 32,290 gene pairs, respectively. For all cutoffs, we mapped the genes to their respective pathways using the KEGG database. We compared the number of putative SL gene pairs with the same pathway to the number expected in a group of that size at random. Significance was assessed using the Fisher exact test.

#### Protein complexes are significantly enriched for putative synthetic lethal pairs

We identified all complexes from the CORUM mammalian protein complex database where all members of the complex mapped unambiguously to one Entrez gene ID. We then randomly selected 20 mutually exclusive complexes composed of five proteins each, and identified the SINaTRA scores for all pairwise combinations of the genes associated with these products. We plotted the SINaTRA scores as a heatmap. To test significance, we randomly selected the same number of inter-complex gene pairs as there were intra-complex gene pairs, and applied the Mann-Whitney U test.

We additionally investigated whether this trend of significance would hold for all protein complexes that were composed of ≤10 proteins from our filtered list, and for all protein complexes in our filtered list. Significance was tested using the same methodology and the Mann-Whitney U test.

#### Context-specific synthetic lethality

Protein expression data in tissues was downloaded from the Protein Atlas. ENS identification codes were mapped to Entrez gene IDs, and putative SL pairs at each SINaTRA cutoff were determined to be NSL in context if both proteins were not detected in the tissue of choice. We identified all gene pairs with SINaTRA≥0.85. For each tissue and cell line, we removed a gene pair from the context-specific SL pair list if both genes’ products were found not to be expressed in the given context. The SL pairs that were not filtered out by this method were considered the retained SL pairs. We calculated the number of expected retained gene pairs as follows:

$$\left(1-\frac{\#removed\;pairs}{total\;\#\;human\;pairs}\right)*N$$

where N is the total, unfiltered number of gene pairs that are SL at the chosen cutoff.

#### Comparison to previously published methods

SL predictions from the Syn-Lethality and DAISY papers were mapped to their Entrez gene terms, and we found the SINaTRA score of each pair. Significance compared to random SINaTRA pairs was evaluated using the Mann-Whitney U test. We constructed classifiers for DAISY and Syn-Lethality using SINaTRA scores as the features and status in the given dataset as the class. We compared this with homology and functional similarity (GO).

We next tested the ability of three methods (SINaTRA, functional similarity, homology) to predict membership in the DAISY and Syn-Lethality datasets. Only pairs from the tested VHL predictions were used form DAISY. We selected an equal number of gene pairs belonging in the dataset (positive examples) and not in the dataset (negative examples), and identified the SINaTRA scores, homology-based SL status from *S*. *cerevisiae*, and within-species functional similarity (discrete) score for each. These scores were used in calculation of the ROC curve and precision-recall curves.

#### The landscape of human synthetic lethality

In order to graphically explore the landscape of human synthetic lethality, we identified all gene pairs with SINaTRA scores ≥0.95. These were mapped to the Reactome database, using the highest terms in the hierarchy: apoptosis; binding and uptake of ligands by scavenger receptors; cell cycle; cell-cell communication; cellular response to stress; chromatin organization; circadian clock; developmental biology; disease; DNA repair; DNA replication; extracellular matrix organization; gene expression; hemostasis; membrane trafficking; metabolism; metabolism of proteins; muscle contraction; neuronal system; organelle biogenesis and maintenance; reproduction; signal transduction; and transmembrane transport of small molecules. Of the 1,229 gene pairs with SINaTRA scores ≥0.95, 458 existed where both members mapped to a Reactome label.

SL pairs were represented in pathway-specific networks visualized in Cytoscape, [46] where both genes were part of the same pathway. Genes are nodes, and two nodes were connected if their SINaTRA score is ≥0.95. Nodes are colored by closeness centrality, and their size depends on node degree. Pathway-specific networks are designated by hexagons, which are joined to each other with edges weighted by the number of inter-pathway SL pairs that exist; that is, gene pairs with mutually exclusive pathway designations.

#### Function-specific mechanisms of synthetic lethality

We identified all gene pairs of the functions from the previous section, as well as an SL subset (SINaTRA score ≥0.85). We then calculated the median value of all node-pair and single-node parameters and plotted a heat map of the ratio of SL to all gene parameters. Because of the low variance between single-node parameters, we clustered each function by the node-pair parameters.

We next annotated all SL pairs with Reactome pathways into three groups: complex, parallel, and other. Two genes were annotated with “complex” if their protein products were known to participate in a protein complex together. Two genes were annotated with “parallel” if they had the same functional annotation but no direct interaction according to Reactome. Finally, two genes were annotated as other if they did not fit these either the “complex” or “parallel” definitions. For each functional category we tested if the gene pairs were enriched for parallel or complex annotations using a Fisher’s exact test.

### Putative synthetic lethal pairs suggest novel cancer therapies

#### Mapping drugs to gene product targets

We first mapped all gene pairs with SL score > 0.85 to drugs in the Drug Combination Database (DCDB),[48] such that both genes in a pair mapped to a cancer drug that targeted their products. Cancer drugs were identified from DCDB as those with indications containing the terms *cancer*, *leukemia*, *carcinoma*, *myeloma*, *tumor*, *sarcoma*, *lymphoma*, or *neoplasm*. From these gene pairs, we identified all unique genes among the pairs. We found a list of 62 unique genes from a list of 381 pairs.

#### Putative human synthetic lethal pairs are predictive of investigative cancer therapy

Using the aforementioned list of genes, we identified the SINaTRA score for all pairwise combinations of genes. We plotted these as a heat map, clustering the rows and columns by SINaTRA score. We then identified all known single-drug and cancer combination therapies in experimental and clinical pipelines using DCDB, and overlaid these data on the clustered heat map to visually identify clusters of therapies and their correspondence to SINaTRA score. We additionally identified which pairs of genes were filtered out using our co-mutation analysis, and confirmed that no gene pairs that were filtered out were also targets of cancer drugs. We performed a Mann-Whitney U test on distributions of SINaTRA scores for non-tested and filtered gene pairs vs. gene pairs associated with drugs, vs. single-drug gene pairs, vs. drug combinations in preclinical testing, and vs. drug combinations in clinical testing.

In order to identify GO enrichment, we tested the GO terms of within-box genes compared to all remaining genes from the Fig 6. Statistical testing was performed using Fisher’s exact test.

### Statistical analyses and software

We calculated network parameters using the NetworkX version 1.8.1. We performed statistical analysis in R version 3.0.2. De Long’s test for comparing ROC curves was implemented using the pROC library.[49] Scripts use Python version 2.7.5. Graphics were generated using Python’s Matplotlib.[50] BioGrid release 3.2.104 was used in all analyses.

## Supporting Information

S1 Fig Distribution of network parameters for the *S*. *cerevisiae* (red) and *S*. *pombe* (blue) networks. Mann-Whitney U test indicates that the parameters are significantly differently distributed between species.
(TIF)

S2 Fig Use of SINaTRA makes network parameters that are not comparable before translation (red) easily compared after translation (gray). (TIF)

S3 Fig Interspecies gene-pair connectivity homology is measured using the Euclidean distance between vectors of single-node parameters for both genes (lower distance implies higher similarity). We find that gene pairs with the same specific function (≤100 genes annotated with that GO term) are significantly more similar to each other than gene pairs with different functions; this effect is consistent even when accounting for homology (*: p<0.05; ***: p<2.2e-16; Mann-Whitney U test).
(TIF)

S4 Fig Interspecies gene-pair connectivity homology is measured using the Euclidean distance between vectors of single-node parameters for both genes (lower distance implies higher similarity). The maximum number of genes annotated by each GO term was changed to determine how specific each function is (x-axis). For each cutoff, the median distance between non-homologous gene pairs with different functions is higher than for all homologous gene pairs, and for non-homologous gene pairs with the same function.
(TIF)

S5 Fig A. We performed classification of SL within two species: *S*. *cerevisiae* and *S*. *pombe*. We considered logistic regression (LogReg) vs. random forest (RanFor) to pick the more robust method. We found that random forest significantly outperformed logistic regression in both species (p<0.0001, De Long’s Method). B.Receiver operating characteristic for within-species classification of SL in *S*. *cerevisiae* using raw (red) and rank-normalized (yellow) data; both achieved an AUC of 0.91. In addition, SL labels were permuted (blue), achieving an AUC no better than chance. C. Correlation between 5,000 gene pairs’ SINaTRA scores using raw and rank-normalized data. Pearson R correlation is 0.97 (p<0.0001). D. SINaTRA score cutoff vs. positive predictive value. We computed PPV at each SINaTRA score cutoff (all gene pairs with SINaTRA score greater than the cutoff were considered to be SL), and found that it increased to approximately 0.1 at a SINaTRA score cutoff of 0.95.
(TIF)

S6 Fig A. Normalization method performance in SL prediction from *S*. *cerevisiae* to *S*. *pombe*. Normalization methods are described in Table 2 in the main text. B. Precision-recall curves for SINaTRA (red) and untranslated (blue).
(TIF)

S7 Fig Prediction of SL from *S*. *pombe* to *S*. *cerevisiae* using untranslated (blue) and translated (red) parameters. The black dotted line represents expected ROC by chance. Raw and SINaTRA ROC curves were significantly different (DeLong’s test).
(TIF)

S8 Fig We create classifiers based on genetic homology (AUC = 0.60), genetic homology extrapolated to the whole genome (WG Homology; AUC = 0.52), protein domain (PFam; AUC = 0.56), protein structure (SCOP; AUC = 0.62), bi-nodal information centrality (AUC = 0.46), and function (GO; AUC = 0.81), and compare these performances to SINaTRA (AUC = 0.86) and SINaTRA restricted to only pairs existing in the homology database (SINaTRA (Hom.); AUC = 0.91) when predicting SL in *S*. *pombe*. (TIF)

S9 Fig For each table, the upper left corner is true positives (TP); upper right is false positives (FP); bottom left is false negatives (FN); and bottom right is true negatives (TN). We found that the number of true positives, as well as the PPV, is significantly higher in SINaTRA-based methods than homology-based ones. See Materials and Methods for details.
(TIF)

S10 Fig Network ablation. A. SL prediction from full *S*. *cerevisiae* to ablated *S*. *pombe* networks using untranslated parameters. Black line represents AUC, while colored lines represent ROC; red is highest ablation (50%), while violet is lowest (10%). B. SL prediction from full *S*. *cerevisiae* to ablated *S*. *pombe* networks using SINaTRA. Black line represents AUC, while colored lines represent ROC; red is highest ablation (50%), while violet is lowest (10%). C. Precision-recall curves of SL prediction from full *S*. *cerevisiae* to ablated *S*. *pombe* networks using untranslated parameters. D. Precision-recall curves of SL prediction from full *S*. *cerevisiae* to ablated *S*. *pombe* networks using SINaTRA.
(TIF)

S11 Fig We plotted the median SINaTRA score of all genes for *S*. *cerevisiae*, *S*. *pombe*, and humans vs. node degree, node popularity (the number of times it appears in the BioGrid database), and normalized popularity (popularity/degree). We found that, while SINaTRA score is correlated with the former two measures, it is not correlated with the latter, which gives a better approximation of research bias.
(TIF)

S12 Fig We identified all human gene pairs with SINaTRA≥0.85 and all tissue- and cell-line-specific SL pairs by filtering out all gene pairs where neither gene product is expressed in the tissue/cell-line. A) The proportion of retained SL pairs by tissue. Tissues are color-coded by the system to which they belong (legend: far left). B) The proportion of retained SL pairs by cell type. Cells were associated with tissue and mapped to system. Cells occurring in multiple tissues from different systems are coded as “other.” C) The observed number of retained tissue-specific SL pairs (blue) versus the expected number (red; model described in Materials and Methods). D) The observed (blue) vs. expected (red) number of retained cell-specific SL pairs. The presence of higher- or lower-than-expected numbers of retained SL pairs may indicate context-specific resistance or susceptibility to SL interactions.
(TIF)

S13 Fig Precision-recall curves for SINaTRA and functional homology’s abilities to predict members of the DAISY and Syn-Lethality studies. (TIF)

S14 Fig Putative functional SL pairs were annotated using Reactome pathways and grouped into three sets: within-complex interaction, other interaction, and unknown. The fraction of SL pairs in each group is illustrated here by function.
(TIF)

S15 Fig We observed that gene pairs targeted by drugs are significantly enriched in SINaTRA score, and the median scores increase from genes that contain only one non-cancer drug target, to those that are affected by two non-cancer drug targets, to those that contain one cancer drug target, to those that contain two. The differences are significant for all comparisons.
(TIF)

S16 Fig Creating network-based classifiers using untranslated data (top) and rank-normalized (translated) data (bottom). (TIF)

S1 Table A description of SINaTRA parameters, including equations where applicable. (PNG)

S2 Table The comparison of distributions of all network parameters between species, described using the Mann-Whitney U test (“MWU”) and associated p-values. (PDF)

S3 Table Columns 2–3 represent AUCs of models based on non-translational or non-network methods of predicting SL, and those methods plus SINaTRA. Columns 4–5 describe results of ANOVAs of nested general linear models of SINaTRA, then SINaTRA plus each of the methods. Only functional similarity provides an improved model when combined with SINaTRA.
(XLSX)

S4 Table SINaTRA predictions of mouse SL pairs. (PDF)

S5 Table SINaTRA predictions of human SL pairs. (PDF)

S6 Table The number of edges removed in each tissue- and cell-specific context compared to the expected number removed. OR and p-values are calculated using Fisher’s exact test.
(XLSX)
