# Supplementary figures and images for: Connectivity Homology Enables Inter-Species Network Models of Synthetic Lethality
Source: PLoS Comput Biol. 2015 Oct 9;11(10):e1004506. doi: 10.1371/journal.pcbi.1004506 (PMC4599967; doi:10.1371/journal.pcbi.1004506)

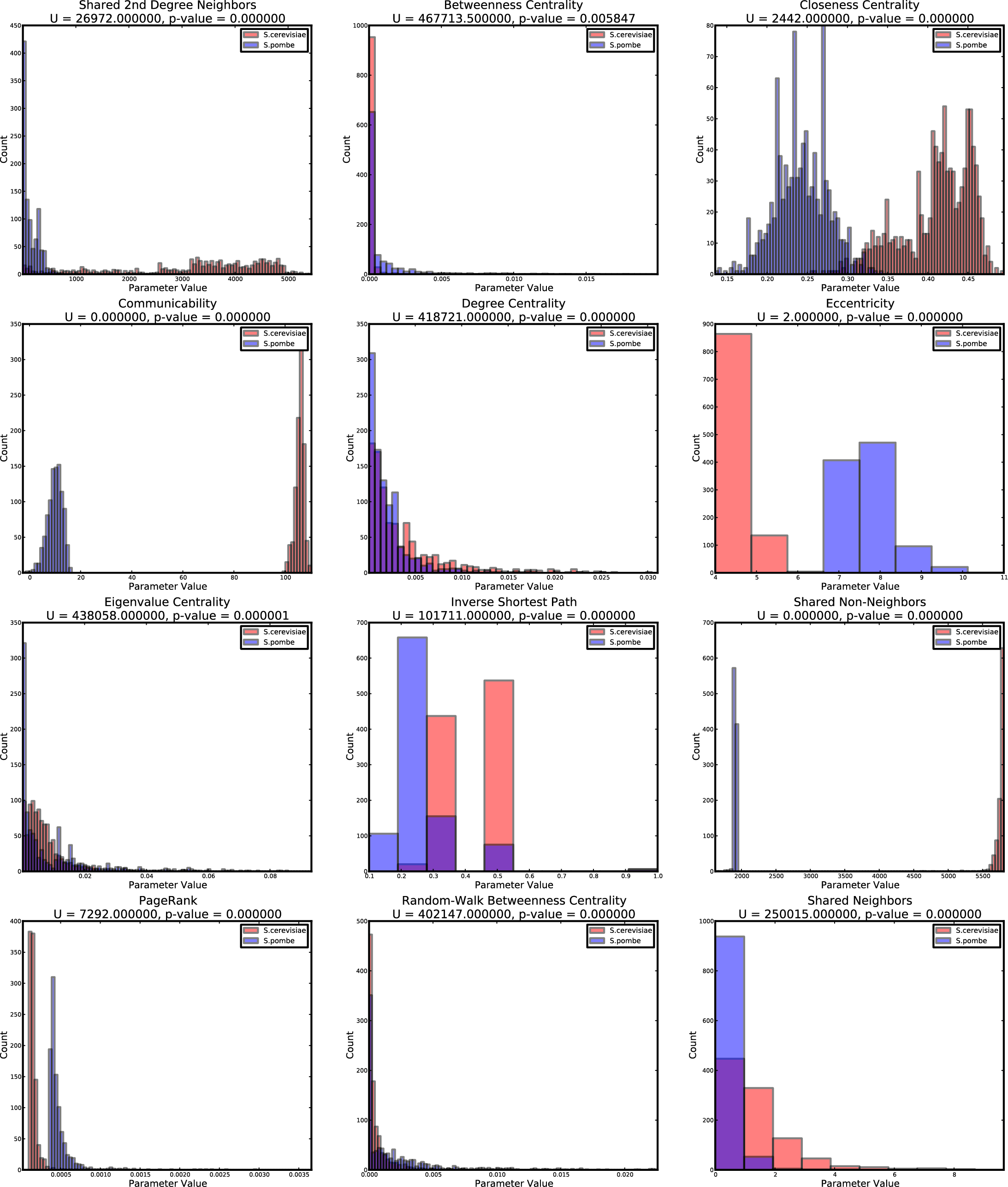

Supplement: S1 Fig — Mann-Whitney U test indicates that the parameters are significantly differently distributed between species. (TIF) [file pcbi.1004506.s001.tif]

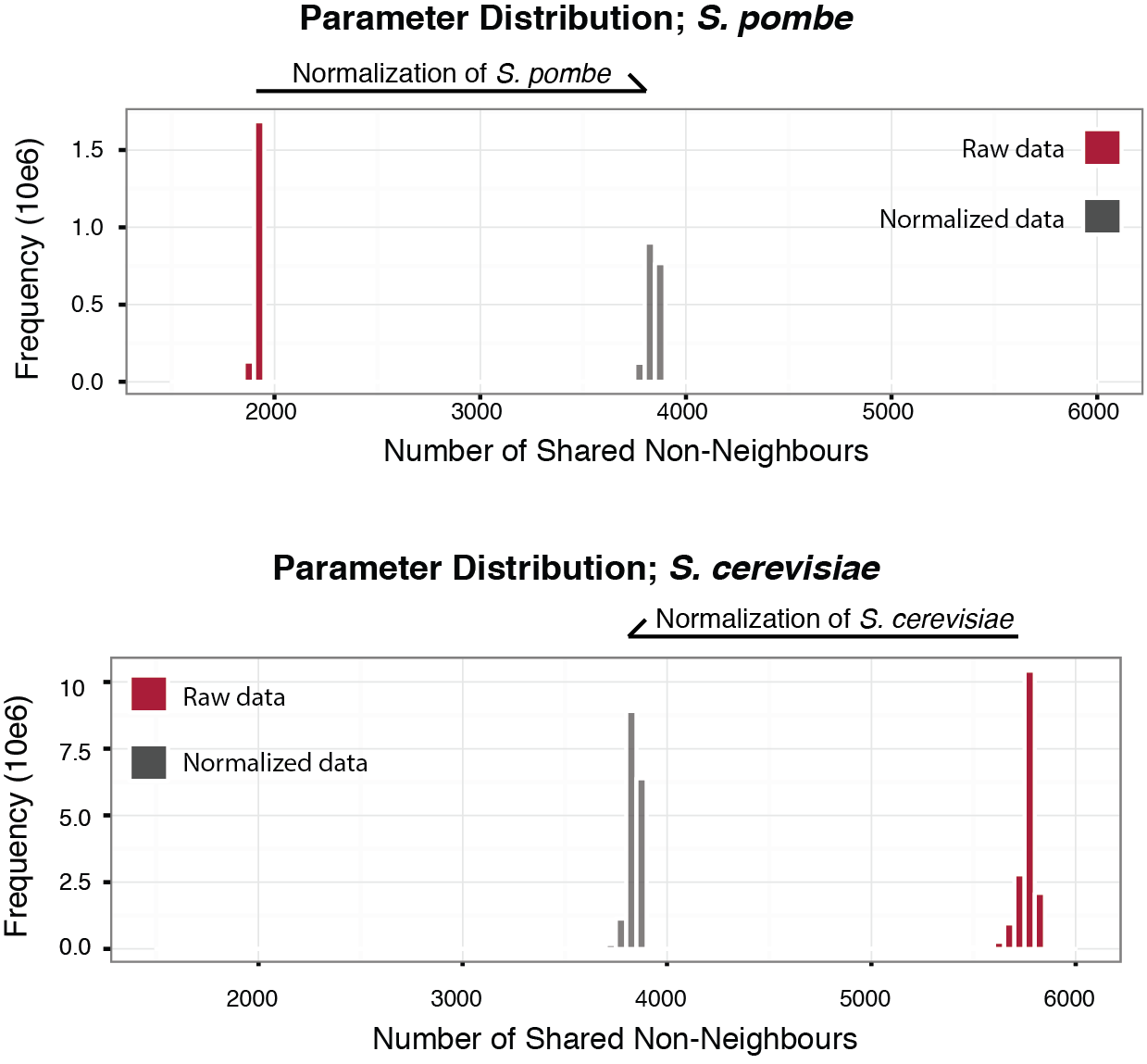

Supplement: S2 Fig — (TIF) [file pcbi.1004506.s002.tif]

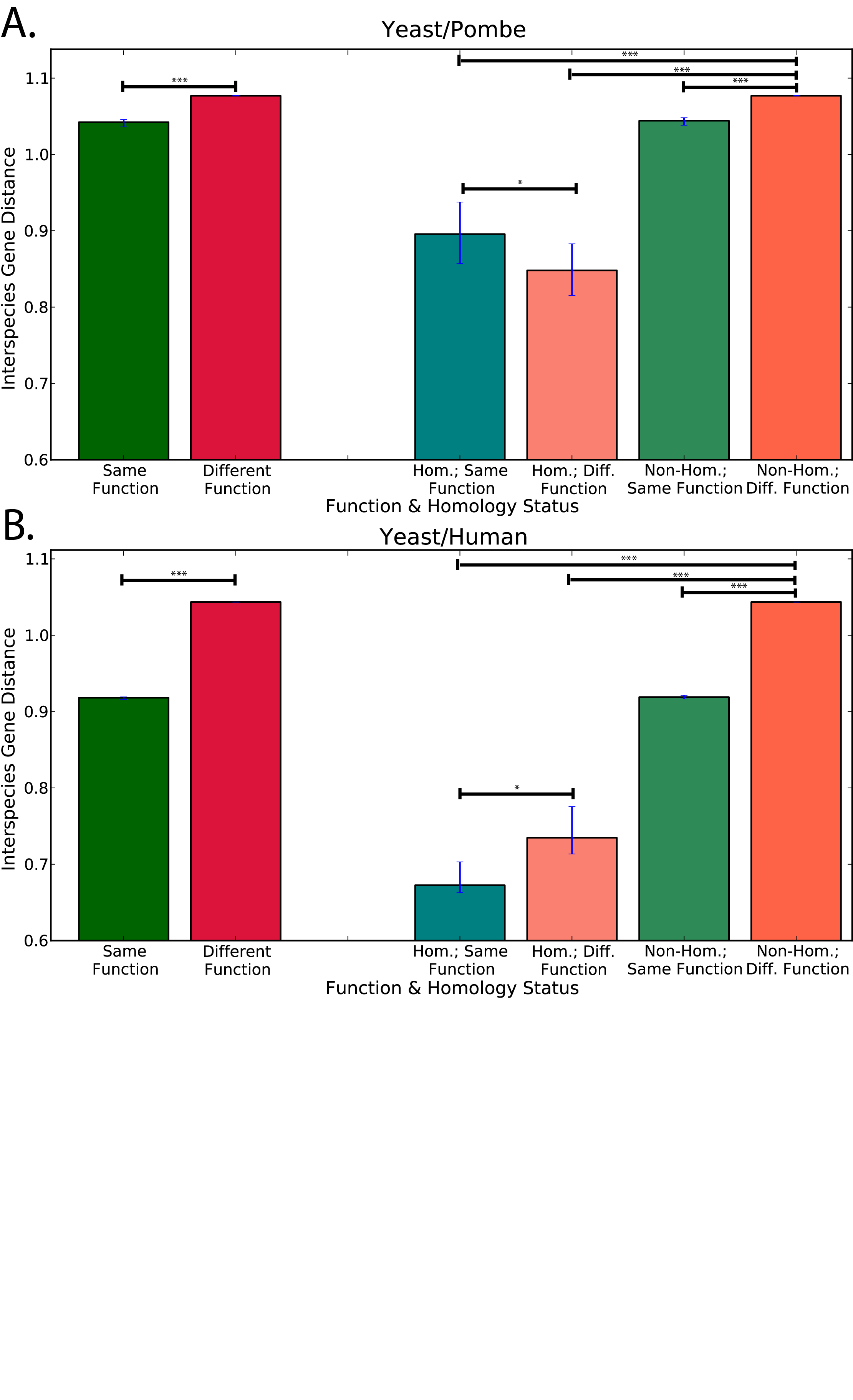

Supplement: S3 Fig — We find that gene pairs with the same specific function (≤100 genes annotated with that GO term) are significantly more similar to each other than gene pairs with different functions; this effect is consistent even when accounting for homology (*: p<0.05; ***: p<2.2e-16; Mann-Whitney U test). (TIF) [file pcbi.1004506.s003.tif]

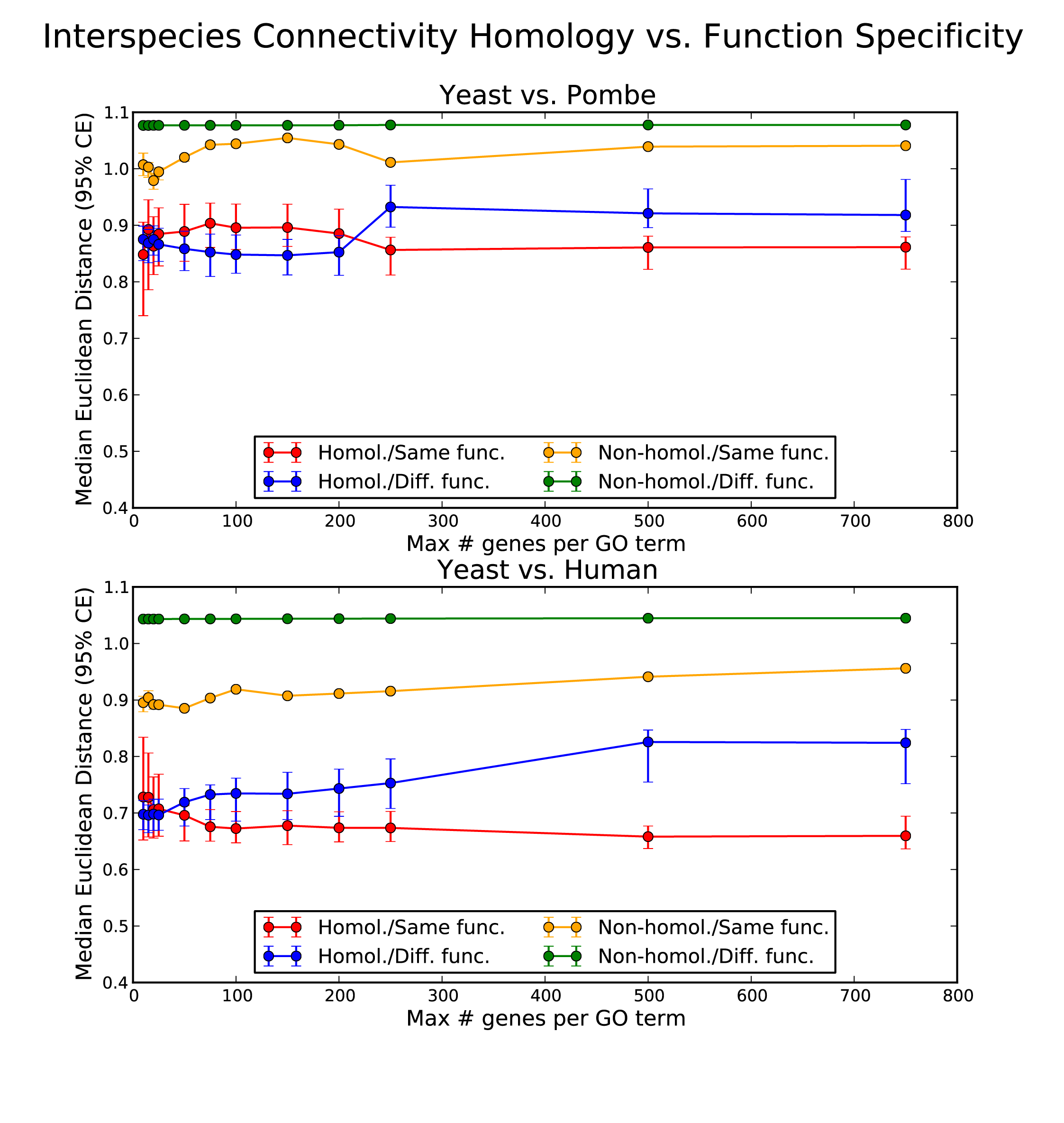

Supplement: S4 Fig — The maximum number of genes annotated by each GO term was changed to determine how specific each function is (x-axis). For each cutoff, the median distance between non-homologous gene pairs with different functions is higher than for all homologous gene pairs, and for non-homologous gene pairs with the same function. (TIF) [file pcbi.1004506.s004.tif]

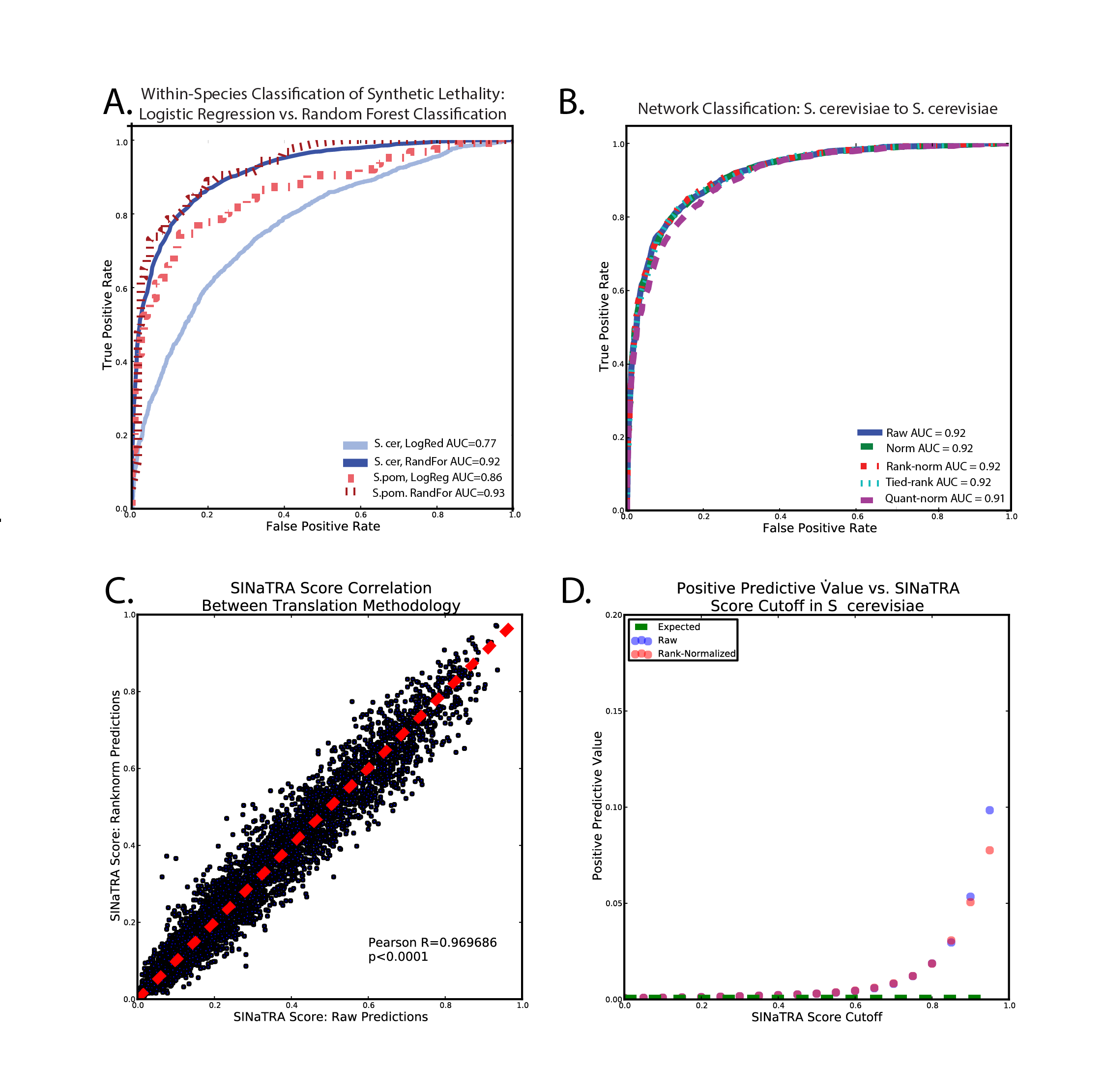

Supplement: S5 Fig — We considered logistic regression (LogReg) vs. random forest (RanFor) to pick the more robust method. We found that random forest significantly outperformed logistic regression in both species (p<0.0001, De Long’s Method). B.Receiver operating characteristic for within-species classification of SL in S. cerevisiae using raw (red) and rank-normalized (yellow) data; both achieved an AUC of 0.91. In addition, SL labels were permuted (blue), achieving an AUC no better than chance. C. Correlation between 5,000 gene pairs’ SINaTRA scores using raw and rank-normalized data. Pearson R correlation is 0.97 (p<0.0001). D. SINaTRA score cutoff vs. positive predictive value. We computed PPV at each SINaTRA score cutoff (all gene pairs with SINaTRA score greater than the cutoff were considered to be SL), and found that it increased to approximately 0.1 at a SINaTRA score cutoff of 0.95. (TIF) [file pcbi.1004506.s005.tif]

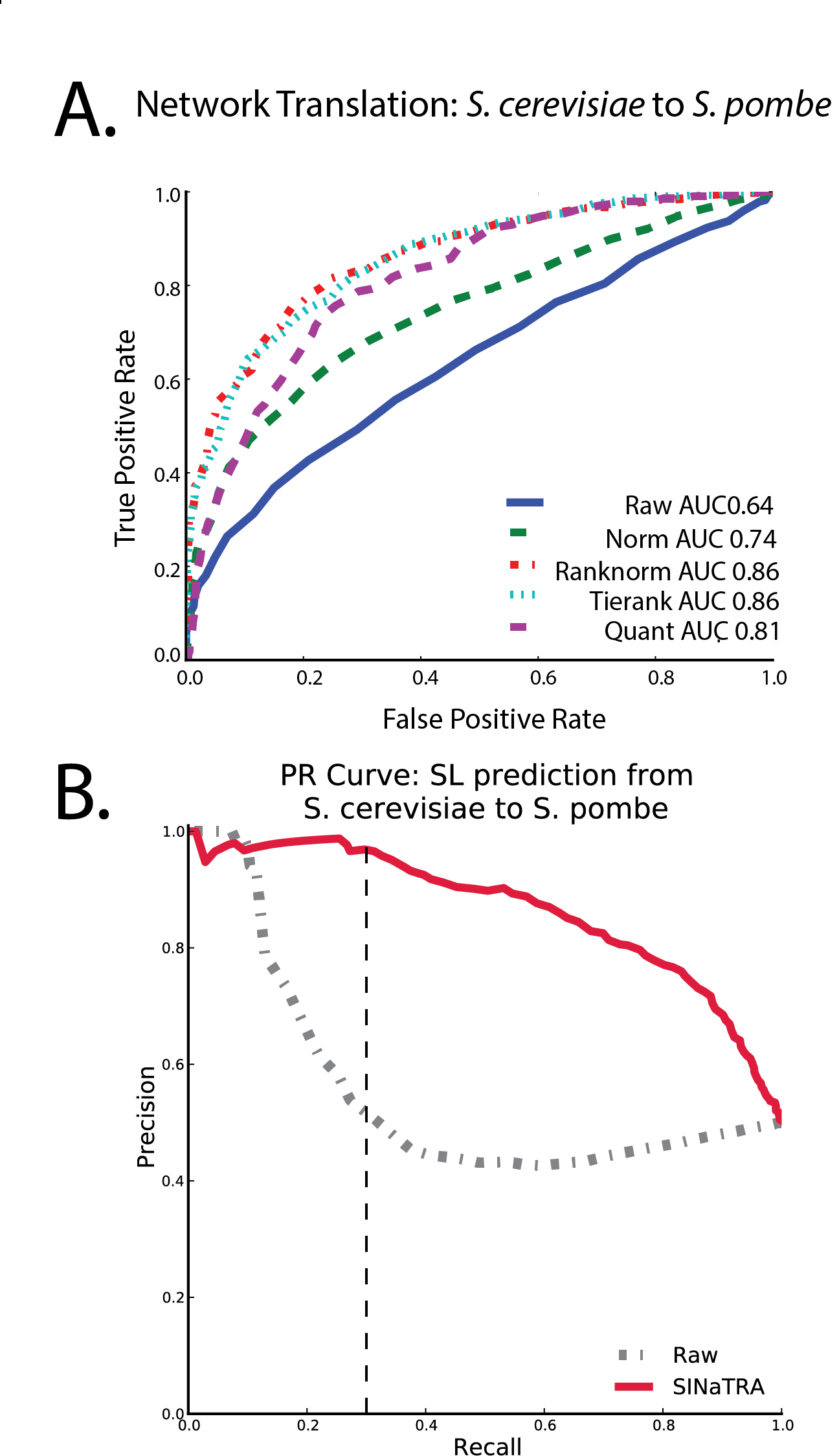

Supplement: S6 Fig — Normalization methods are described in Table 2 in the main text. B. Precision-recall curves for SINaTRA (red) and untranslated (blue). (TIF) [file pcbi.1004506.s006.tif]

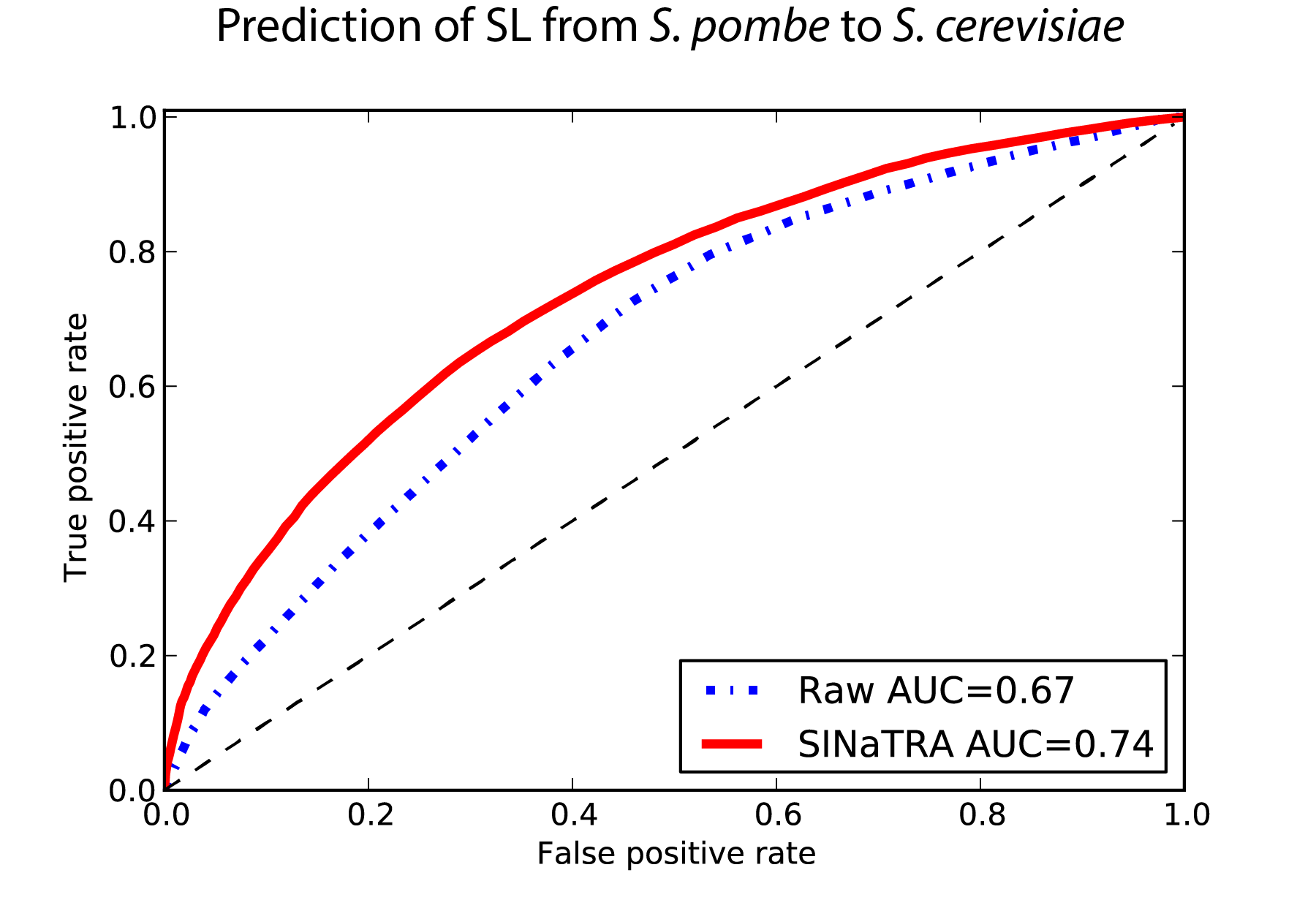

Supplement: S7 Fig — The black dotted line represents expected ROC by chance. Raw and SINaTRA ROC curves were significantly different (DeLong’s test). (TIF) [file pcbi.1004506.s007.tif]

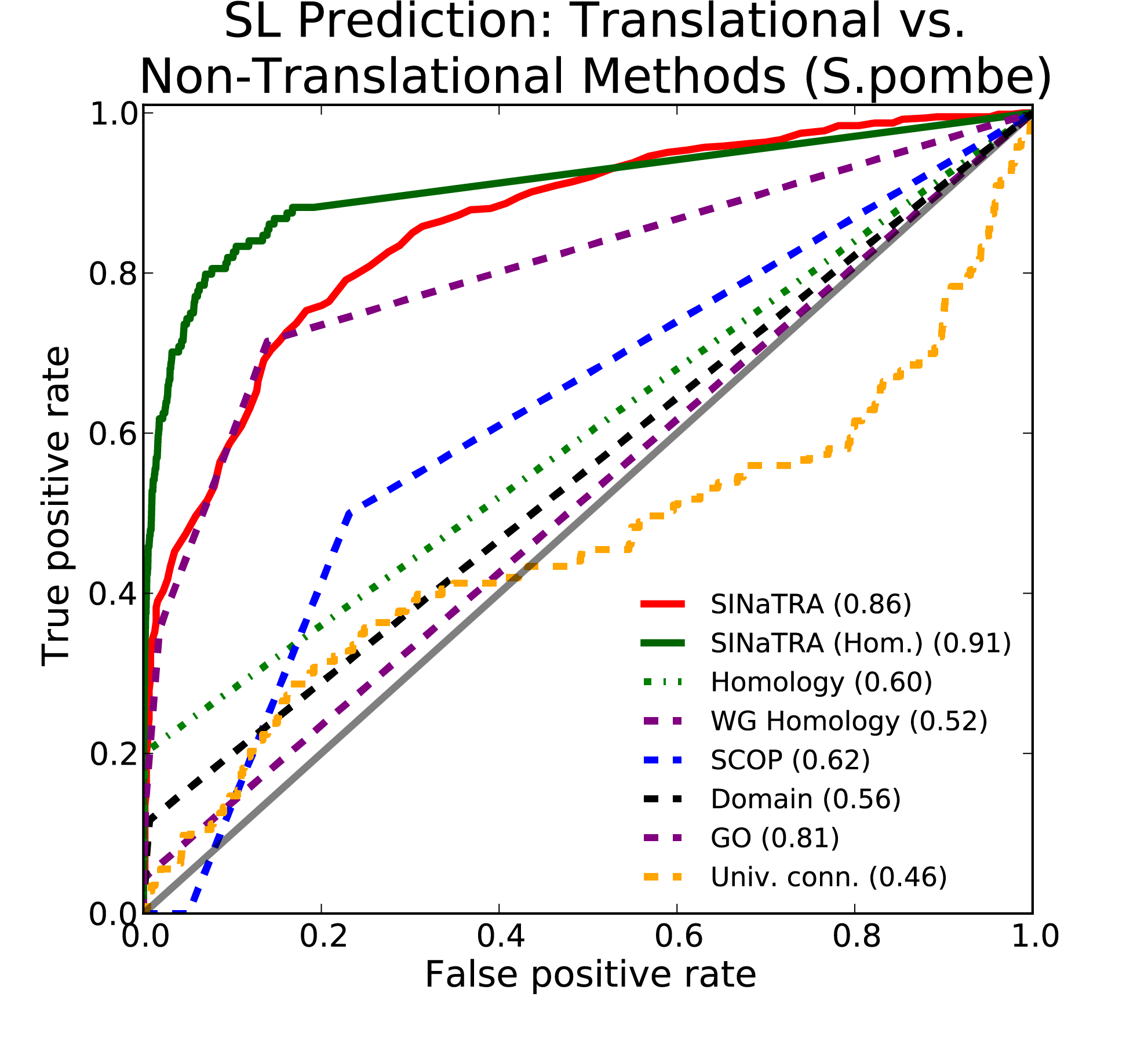

Supplement: S8 Fig — (TIF) [file pcbi.1004506.s008.tif]

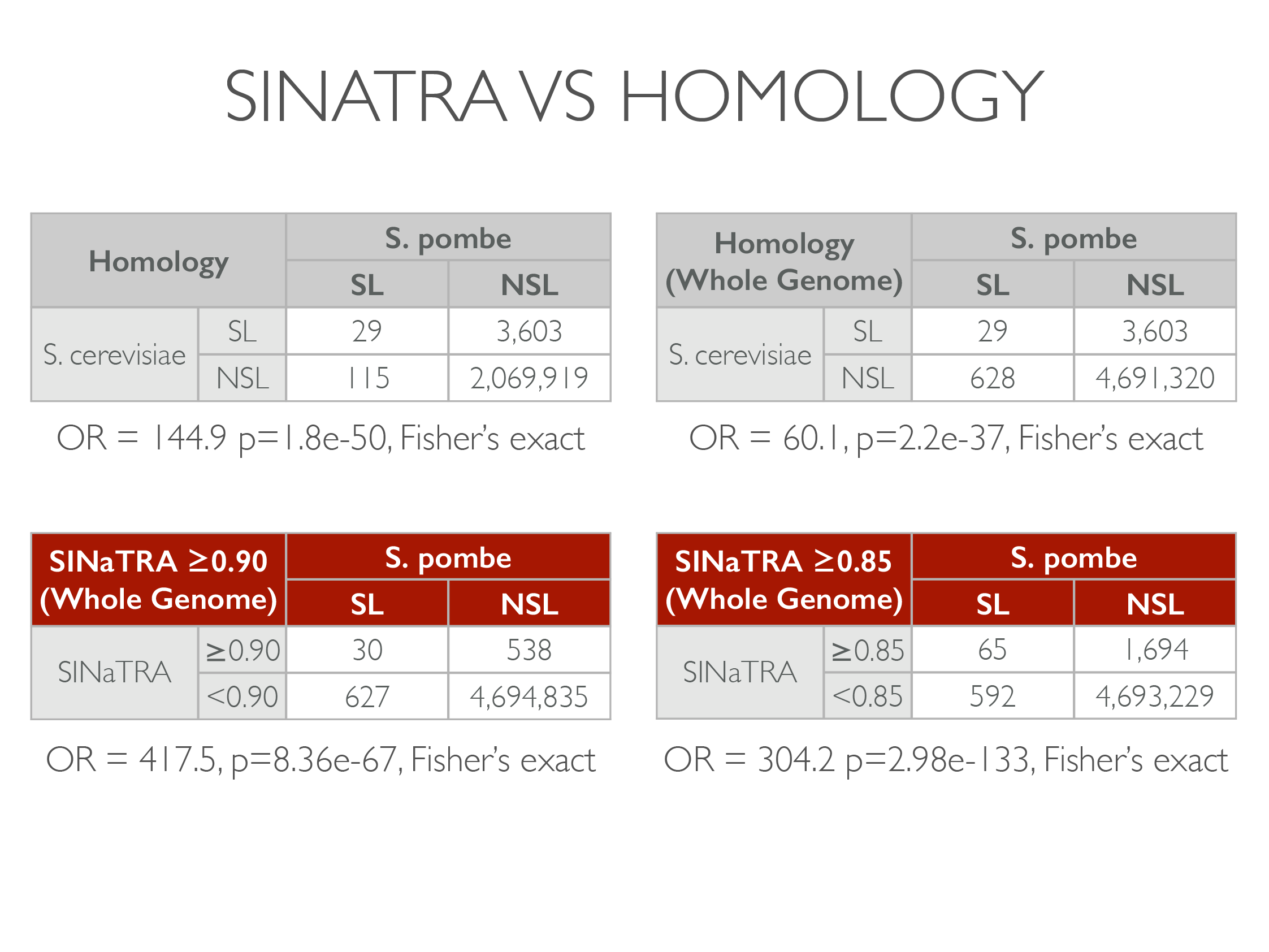

Supplement: S9 Fig — We found that the number of true positives, as well as the PPV, is significantly higher in SINaTRA-based methods than homology-based ones. See Materials and Methods for details. (TIF) [file pcbi.1004506.s009.tif]

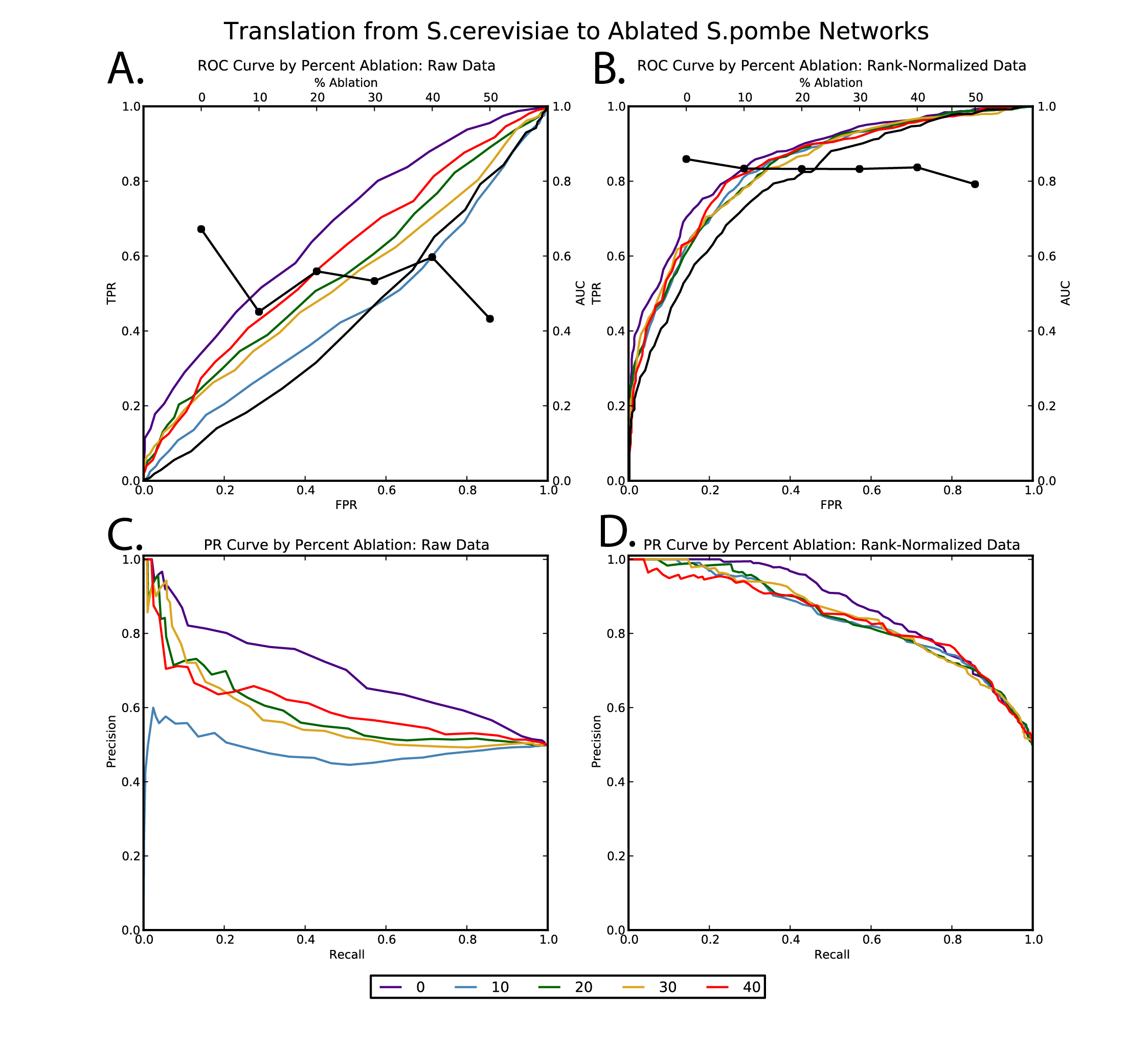

Supplement: S10 Fig — A. SL prediction from full S. cerevisiae to ablated S. pombe networks using untranslated parameters. Black line represents AUC, while colored lines represent ROC; red is highest ablation (50%), while violet is lowest (10%). B. SL prediction from full S. cerevisiae to ablated S. pombe networks using SINaTRA. Black line represents AUC, while colored lines represent ROC; red is highest ablation (50%), while violet is lowest (10%). C. Precision-recall curves of SL prediction from full S. cerevisiae to ablated S. pombe networks using untranslated parameters. D. Precision-recall curves of SL prediction from full S. cerevisiae to ablated S. pombe networks using SINaTRA. (TIF) [file pcbi.1004506.s010.tif]

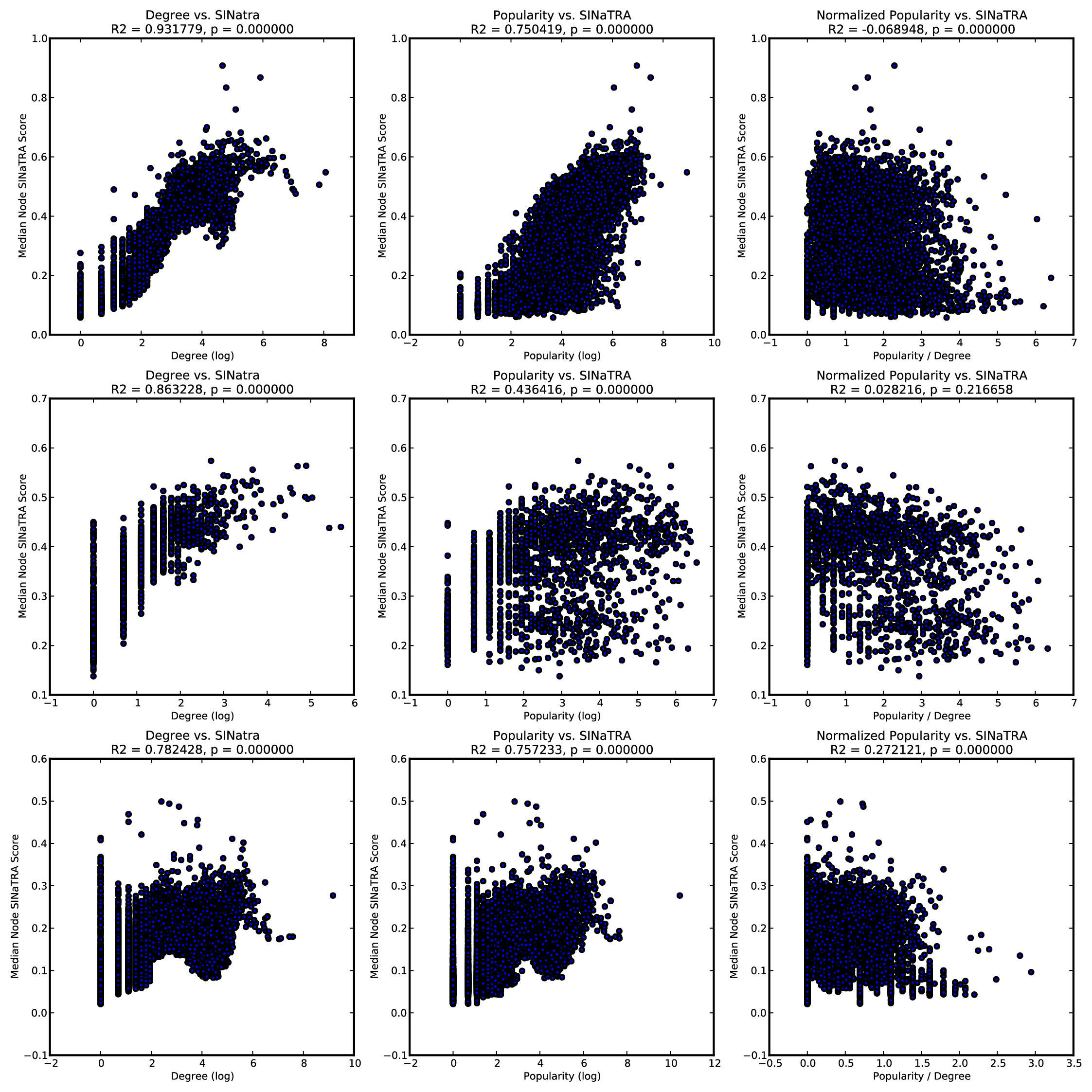

Supplement: S11 Fig — We found that, while SINaTRA score is correlated with the former two measures, it is not correlated with the latter, which gives a better approximation of research bias. (TIF) [file pcbi.1004506.s011.tif]

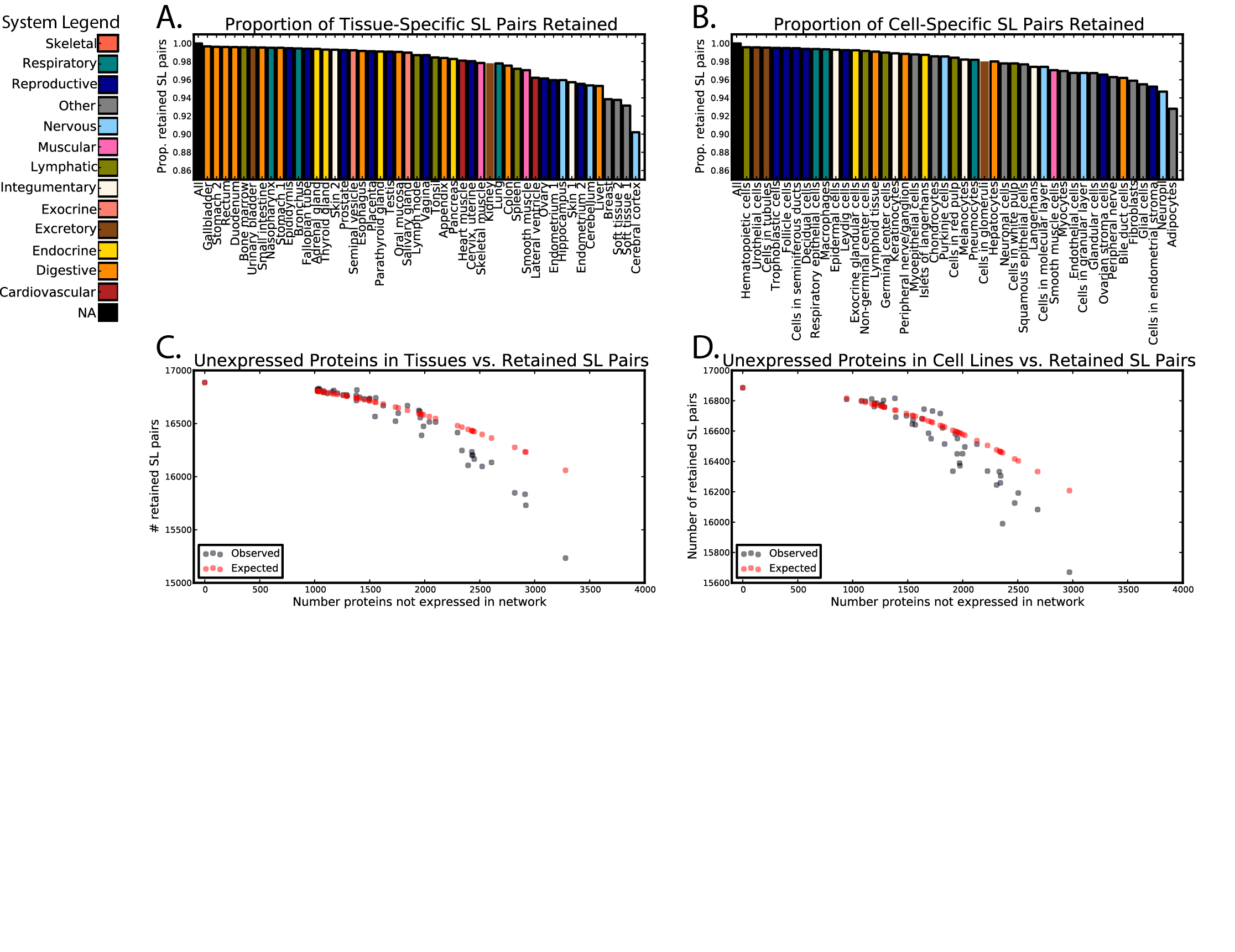

Supplement: S12 Fig — A) The proportion of retained SL pairs by tissue. Tissues are color-coded by the system to which they belong (legend: far left). B) The proportion of retained SL pairs by cell type. Cells were associated with tissue and mapped to system. Cells occurring in multiple tissues from different systems are coded as “other.” C) The observed number of retained tissue-specific SL pairs (blue) versus the expected number (red; model described in Materials and Methods). D) The observed (blue) vs. expected (red) number of retained cell-specific SL pairs. The presence of higher- or lower-than-expected numbers of retained SL pairs may indicate context-specific resistance or susceptibility to SL interactions. (TIF) [file pcbi.1004506.s012.tif]

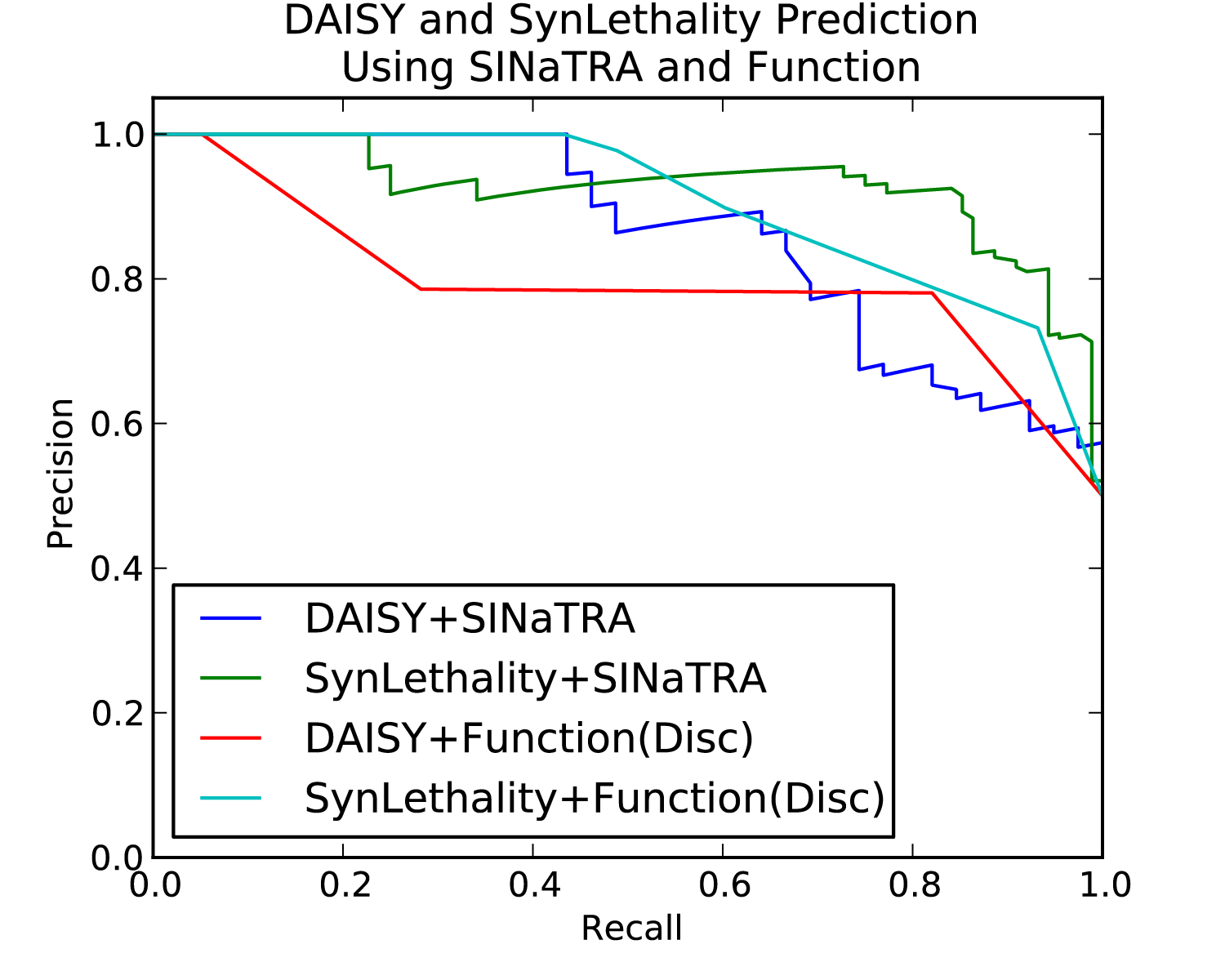

Supplement: S13 Fig — (TIF) [file pcbi.1004506.s013.tif]

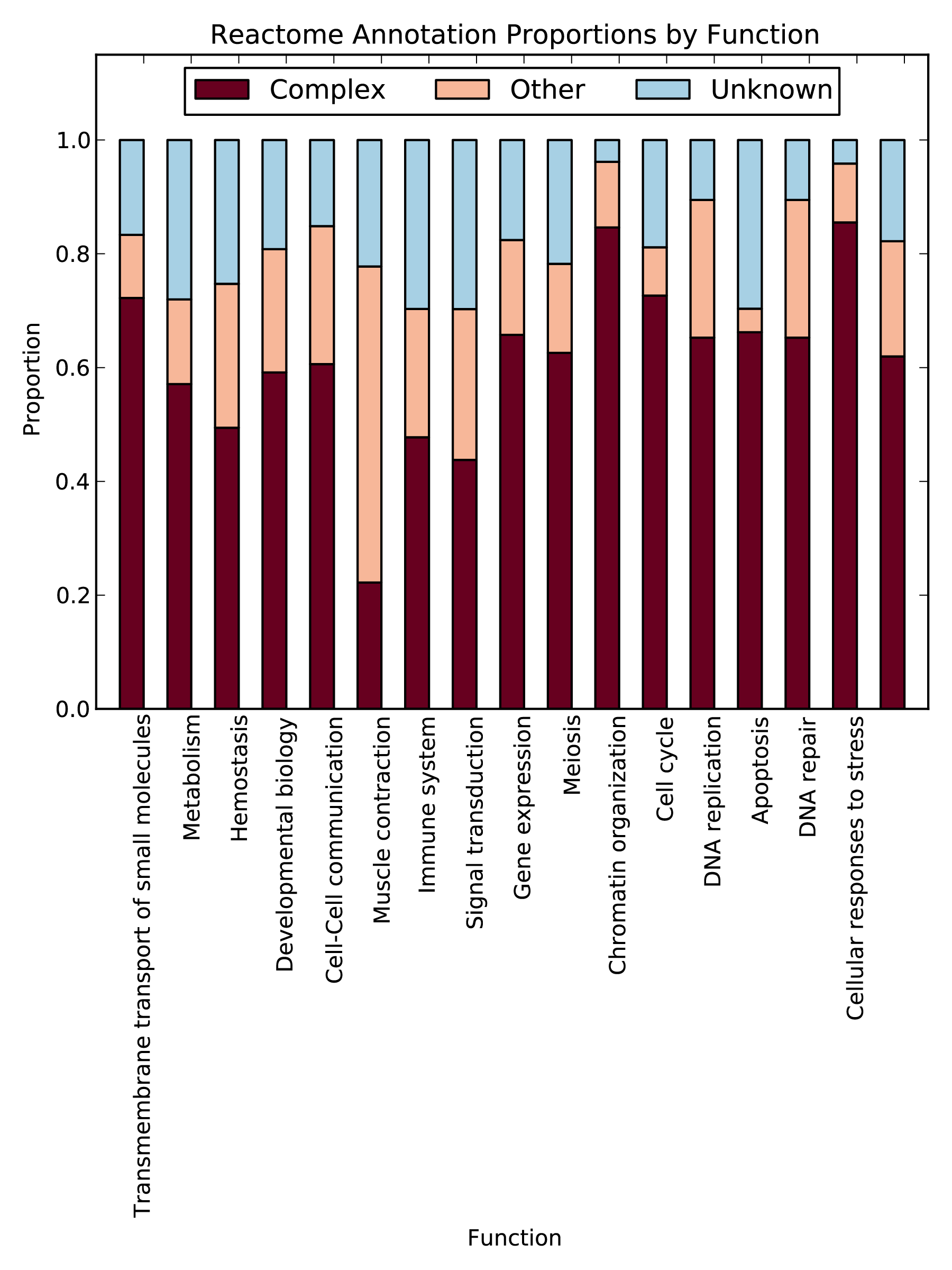

Supplement: S14 Fig — The fraction of SL pairs in each group is illustrated here by function. (TIF) [file pcbi.1004506.s014.tif]

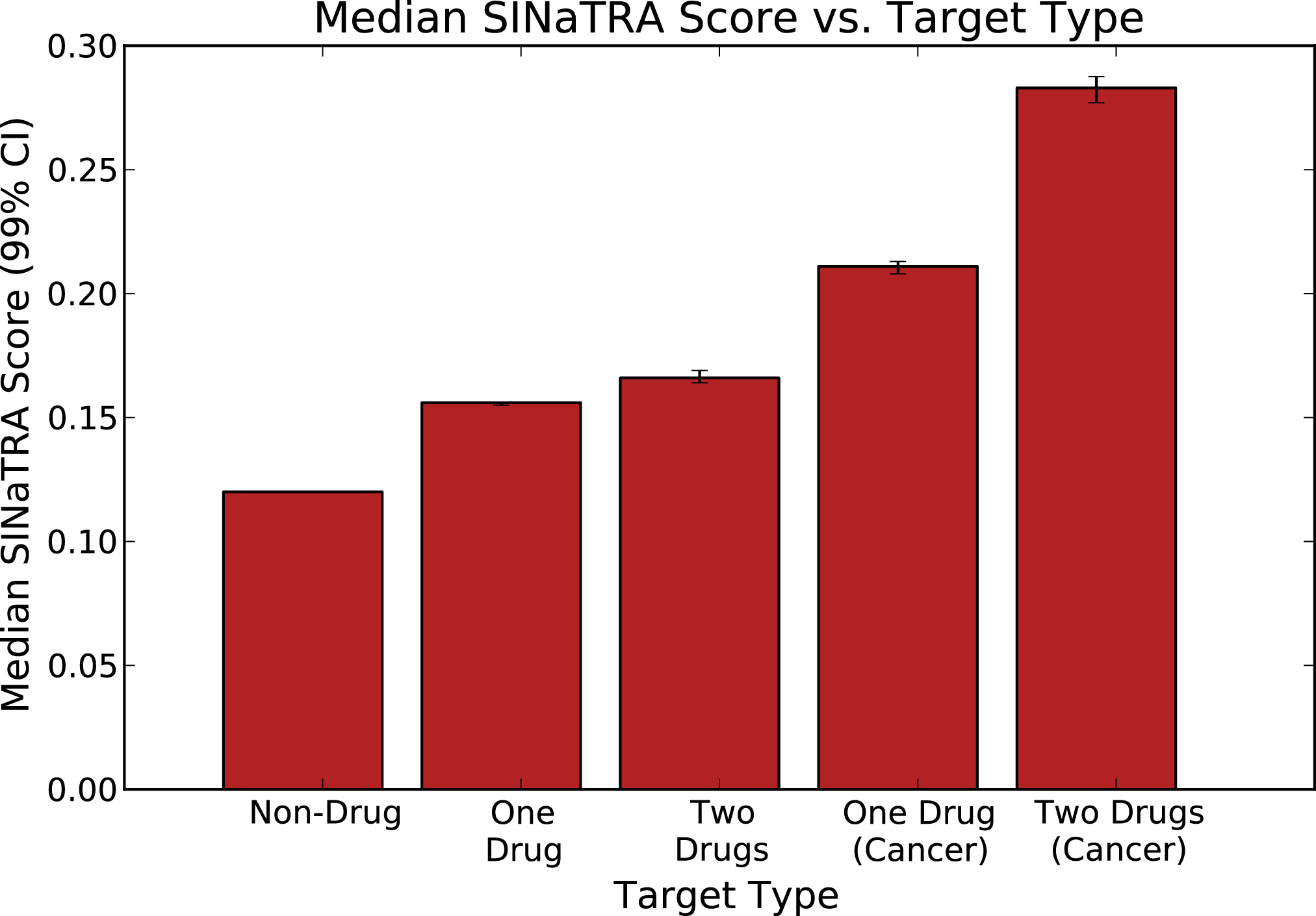

Supplement: S15 Fig — The differences are significant for all comparisons. (TIF) [file pcbi.1004506.s015.tif]

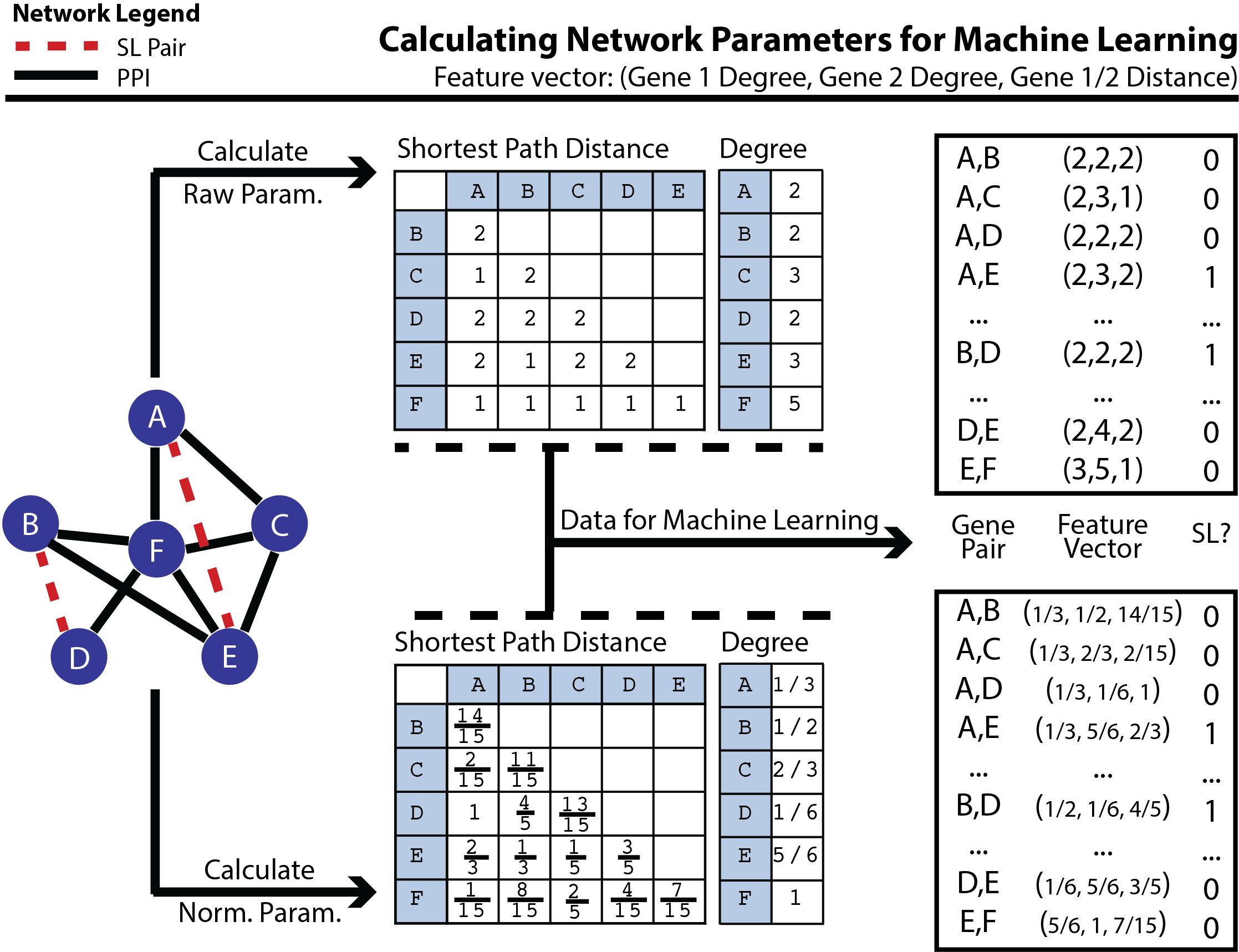

Supplement: S16 Fig — (TIF) [file pcbi.1004506.s016.tif]

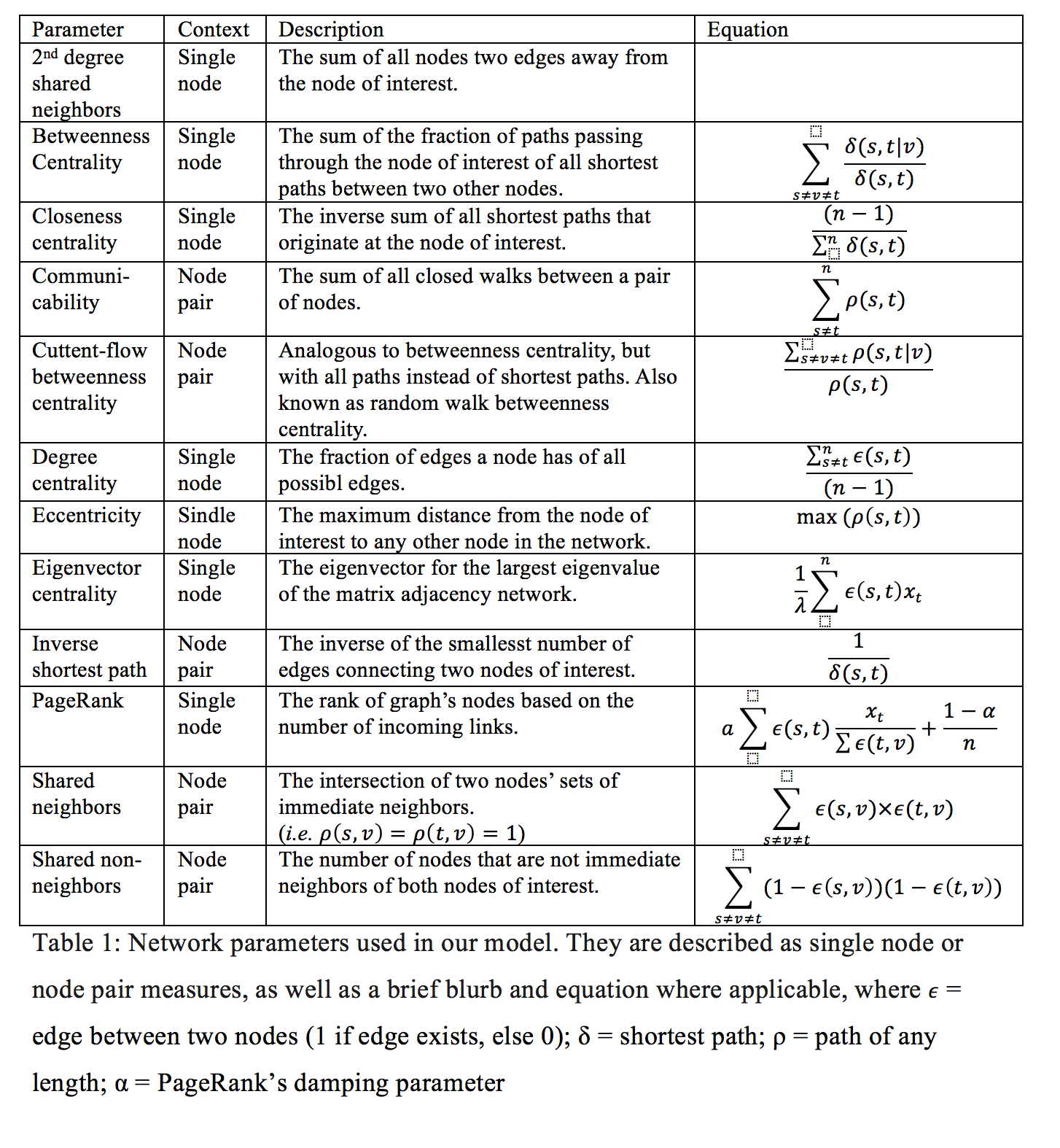

Supplement: S1 Table — (PNG) [file pcbi.1004506.s017.png]
