# Supplementary material for: Connectivity Homology Enables Inter-Species Network Models of Synthetic Lethality
Source: PLoS Comput Biol. 2015 Oct 9;11(10):e1004506. doi: 10.1371/journal.pcbi.1004506 (PMC4599967; doi:10.1371/journal.pcbi.1004506)
Supplement: S2 Table — (PDF) [file pcbi.1004506.s018.pdf]

		2nd Degree Shared Neighbors	Between-ness Centrality	Closeness Centrality	Communi-cability	Current-flow Betweenness Centrality	Degree Centrality	Eccen-tricity	Eigenvector Centrality	Inverse Shortest Path	PageRank	Shared Neighbors	Shared non-neighbors
**Cerevisae-Pombe**	**MWU**	29693.5	460889	2192	0	398166	413170	2	437106	101498.5	12745	256887.5	0
	**p-value**	9.84E-291	0.0011	<2.2E-16	<2.2E-16	1.31E-15	7.86E-12	<2.2E-16	5.56E-07	2.50E-224	<2.2E-16	3.10E-118	<2.2E-16
**Cerevisae-Mouse**	**MWU**	31873	442123	504	0	445945.5	194189	0	344992	80512	132671	233678	0
	**p-value**	4.56E-288	2.83E-06	<2.2E-16	<2.2E-16	1.25E-05	1.10E-125	<2.2E-16	1.69E-33	1.18E-247	2.70E-178	4.46E-145	<2.2E-16
**Cerevisae-Human**	**MWU**	311210.5	327722	369844	69	271969.5	196167	44895	350074	438865.5	31788	406604	0
	**p-value**	1.04E-48	3.70E-41	3.39E-24	<2.2E-16	2.96E-70	6.21E-123	<2.2E-16	1.81E-31	5.85E-08	3.40E-288	1.47E-15	<2.2E-16
**Pombe-Mouse**	**MWU**	495858	435956.5	445933	335172	387754.5	194674	20939	412643	480893	34263	488812.5	0
	**p-value**	0.3742	9.48E-08	1.41E-05	1.30E-37	1.62E-19	1.08E-125	<2.2E-16	6.66E-12	0.0602	3.67E-285	0.0205	<2.2E-16
**Pombe-Human**	**MWU**	98830	406552	19307	0	332705	197158	8371	436114	136340	3664	320172	0
	**p-value**	3.08E-212	6.56E-14	1.22E-303	<2.2E-16	2.27E-39	1.02E-122	<2.2E-16	3.76E-07	4.31E-186	<2.2E-16	2.45E-76	<2.2E-16
**Mouse-Human**	**MWU**	85225	447096	14237	0	451730	397249	0	477952	108443	10286	298150	0
	**p-value**	1.00E-226	1.22E-05	5.15E-310	<2.2E-16	6.97E-05	5.53E-16	<2.2E-16	0.0439	5.44E-216	<2.2E-16	2.76E-95	<2.2E-16
