# Supplementary material for: Connectivity Homology Enables Inter-Species Network Models of Synthetic Lethality
Source: PLoS Comput Biol. 2015 Oct 9;11(10):e1004506. doi: 10.1371/journal.pcbi.1004506 (PMC4599967; doi:10.1371/journal.pcbi.1004506)
Supplement: S4 Table — (PDF) [file pcbi.1004506.s020.pdf]

Gene 1	Gene 2	SL-Score
Gpbp1	Gtf2b	0.957
Sh3kbp1	Atxn2	0.954
Gpbp1	Spata24	0.953
Zfp513	Nkd2	0.952
Hist2h4	Srf	0.952
Bach1	Btg2	0.948
Gtf2b	Spata24	0.947
Syn2	Krt76	0.945
Ncam1	Pde10a	0.944
Ptpn6	Cd3e	0.944
Map2k4	Tmem115	0.943
Phc3	Fos	0.943
Ubb	Rnf14	0.943
Fadd	Atg5	0.942
Sap18	Hist1h2ai	0.942
Pias1	Sap18	0.941
Ep300	Myod1	0.941
Tcrb	Cd3e	0.94
Bach1	Crem	0.939
Egr2	Zfp110	0.939
Smc3	Atrx	0.939
Gnb1	Gnao1	0.938
Gnb1	Btk	0.938
Id1	Tfdp2	0.938
Pura	Mybl2	0.938
Pias3	Pias1	0.938
Hist3h2a	Phf17	0.938
H1f0	Ccna2	0.937
Sf3b1	Spata24	0.937
Dok2	Nck1	0.936
Hand2	Hand1	0.936
Ubb	Ulk1	0.936
Myo6	Plekhg5	0.936
Bach1	Hoxd13	0.935
Six6	Tle4	0.935
Hist2h4	H3	0.935
Syn2	Myo5a	0.935
Als2	Tcerg1	0.934
Hist2h4	Rnf14	0.934
Pura	Syn2	0.934
Barx2	Tle1	0.934
Btk	Gng2	0.934
Bag1	Dnajb6	0.933
Phc3	Spata24	0.933
Kif5c	Kif5a	0.933

Syn2	Fus	0.933
Atg5	Atg12	0.932
Ntrk1	Ngfr	0.932
Hist3h2a	Rnf14	0.932
Iqgap1	Pafah1b1	0.932
Id1	Hand1	0.931
Fadd	Fas	0.931
Slc11a1	Spata24	0.931
Egr2	Cflar	0.931
Atg5	Atg16l1	0.931
Tirap	Pag1	0.931
Ncoa6	Cbx7	0.931
Gpbp1	Taf1c	0.93
Id1	Cops8	0.93
Sirt1	Ep300	0.93
Rab7	Sun2	0.93
Pparg	Ifrd1	0.93
Pparg	Ncor1	0.93
Ubb	Pdlim4	0.93
Kif5c	Kif5b	0.93
H1f0	Ccnb1	0.93
Syn2	Capzb	0.93
Pura	Kif5a	0.929
Ncoa6	Spata24	0.929
Syn2	Nf1	0.929
Gtf2h1	Csde1	0.928
Egr2	Tbk1	0.928
Syn2	Bcr	0.928
Il17rc	Il17ra	0.927
Carm1	Ncoa2	0.927
Id1	Tsc22d3	0.927
Sp7	Dlx2	0.927
Aldoa	Suz12	0.927
Eya1	Neurog1	0.927
Pparg	Hdac6	0.927
Cask	Ambra1	0.927
Bach1	Men1	0.926
Lmnb1	Sun2	0.926
Egr2	Stam2	0.926
Ntrk1	Ulk1	0.926
Pparg	E2f1	0.926
Bach1	Col5a1	0.925
Usf1	Ccna2	0.925
Id1	Cops6	0.925
Barx2	Atf2	0.925
Pias3	Taf1c	0.925

Max	Huwe1	0.925
Ryr2	Sri	0.925
Bach1	Col3a1	0.924
Aldoa	Ngfr	0.924
Ubb	Anxa1	0.924
Dynll1	Tubb5	0.924
Hoxd13	Sox15	0.924
Tnf	Fadd	0.923
Gpbp1	Pcmt1	0.923
Rab7	Lbr	0.923
Egr2	Ntrk1	0.923
Ube2i	2-Sep	0.923
Ptpn6	Vav1	0.923
Gtf2h1	Aagab	0.922
Gtf2h1	Cdk7	0.922
Six6	Aes	0.922
Hist2h4	Ubb	0.922
Mtf1	Hoxa1	0.922
Irak4	Tirap	0.922
Egr2	Was	0.922
Dynll1	Tuba1b	0.922
Syn2	Eef1a1	0.922
Foxh1	Dlx2	0.922
Mecp2	Ifrd1	0.921
Gpbp1	Taf5	0.921
Gpbp1	Zfp592	0.921
Gtf2h1	Irf4	0.921
Zfp354a	H3	0.921
Smc2	Crem	0.921
Pparg	Ngfr	0.921
Pparg	Runx2	0.921
Ccnh	Cdk7	0.921
Mef2a	Foxh1	0.92
Epc1	Ncoa6	0.92
Id1	Ascl1	0.92
Id1	Otx2	0.92
Slc11a1	Atg16l1	0.92
Pias3	Zdhhc13	0.92
Sun2	Syne2	0.92
Irf5	Irak1	0.92
Pparg	Sykb	0.92
Gab1	Diap1	0.92
Epc1	Rarg	0.919
Gpbp1	Pias3	0.919
Pcmt1	Rxra	0.919
Sod1	Myog	0.919

Traf3	Ticam1	0.919
Mtf1	Maml1	0.919
Egr2	Sykb	0.919
Pparg	Nr0b2	0.919
Syn2	Ppp2cb	0.919
Syn2	Hspa9	0.919
Gab1	Atxn2	0.919
Sorbs1	Cd2ap	0.918
Gpbp1	Polr1b	0.918
Zfp513	Zscan2	0.918
Ubtf	Polr1b	0.918
Id3	Ivns1abp	0.918
Irf8	Tbk1	0.918
Id1	Irf4	0.918
Sod1	Zfp110	0.918
Gtf2h1	Ccnh	0.918
Slc11a1	H3f3a	0.918
Hist1h3f	Ccnb1	0.918
Pparg	Nr1h4	0.918
Syn2	Arhgef7	0.918
Syn2	Plec	0.918
Myo6	Gipc1	0.918
Ptrf	Ldhb	0.918
Bag1	Creb1	0.917
Id1	Eya3	0.917
Lmnb1	Myh9	0.917
Rab7	Serbp1	0.917
Siah2	Ncor1	0.917
Ppard	Foxh1	0.917
Pparg	Zfp110	0.917
Pparg	Fos	0.917
Scmh1	Spata24	0.917
Fas	Ncor1	0.917
Foxh1	Sox15	0.917
Gpbp1	Eya3	0.916
Rab7	Eif3i	0.916
Mtf2	Ivns1abp	0.916
Ubb	Polr1a	0.916
Ubb	Tuba1b	0.916
Clybl	Rapgef1	0.916
Taf1c	Taf1b	0.916
Bag1	Spna2	0.915
Usf1	Grip1	0.915
Jup	Ctnna1	0.915
Dok2	Rasa1	0.915
Irf8	Ngfr	0.915

Hist2h4	Rfx4	0.915
Egr2	Hdac6	0.915
Egr2	Ngfr	0.915
Pparg	Tbk1	0.915
Hist3h2a	Pdlim4	0.915
Six3	Tle4	0.914
Nrip1	Kcnd2	0.914
Bach1	Eif4enif1	0.913
Map2k4	Malt1	0.913
Rnh1	Dsg1a	0.913
Gtf2h1	Abtb1	0.913
Zfp354a	Hist2h2be	0.913
Max	Pcna	0.913
Mixl1	Pou4f2	0.913
Aldoa	Ntrk1	0.913
Aldoa	Krt73	0.913
Tirap	Tubg1	0.913
Pparg	Hist4h4	0.913
Ncoa6	Ppp2ca	0.913
Ube2d1	Cul7	0.913
Map2k4	Bcl10	0.912
Als2	Grin2d	0.912
Sod1	Ntrk1	0.912
Tle1	Atf2	0.912
Egr2	Phf20	0.912
Aldoa	Krt76	0.912
Ep300	Rela	0.912
Ppard	Nr1h4	0.912
Pparg	Ulk1	0.912
Pparg	Pdx1	0.912
Pparg	Phf12	0.912
Ncoa6	H2afx	0.912
Ubb	Psme3	0.912
Ubb	Phf2	0.912
Fancd2	Cflar	0.912
Klrb1c	Ambra1	0.912
Srf	Hdac5	0.912
Bag1	Suz12	0.911
Il17rc	Traf3ip2	0.911
Usf1	Hist3h2a	0.911
Gpbp1	Kat2a	0.911
Cryab	Fbxo4	0.911
Id3	Ttn	0.911
Six3	Aes	0.911
Egr2	Myog	0.911
Egr2	Tnk2	0.911

Nefl	Nefh	0.911
Ubb	Hist3h2a	0.911
Syn2	Casp3	0.911
Blnk	Gng2	0.911
Hist3h2a	Phf2	0.911
Mef2a	Mef2d	0.91
Map2k4	Mib2	0.91
Gpbp1	Hdac10	0.91
Lmnb1	Lbr	0.91
Polk	Pcna	0.91
Rab7	Rab5a	0.91
Pias3	Taf1a	0.91
Egr2	Arntl	0.91
Egr2	Ctcf	0.91
Egr2	Cdh1	0.91
Med1	Hmga1	0.91
Pparg	Mc4r	0.91
Ubb	Zfp277	0.91
Capn2	Vrk3	0.91
Dynll1	Tuba4a	0.91
Brca1	Brca2	0.91
Bach1	Hoxa13	0.909
Gtf2a1l	Taf13	0.909
Epc1	Cbx8	0.909
Epc1	Hist3h2a	0.909
Kat5	Atm	0.909
Lmnb1	Hsp90b1	0.909
Egr2	Junb	0.909
Aldoa	Myog	0.909
Aldoa	Ldha	0.909
Pparg	Msx2	0.909
Fancd2	Tbk1	0.909
Suz12	Plp1	0.909
Bag1	Ptk2	0.908
Psma4	Glud1	0.908
Psma4	Hnrnpm	0.908
Sin3b	Phf12	0.908
Irf8	Junb	0.908
Sod1	Prkcsh	0.908
Rab3d	Atp5d	0.908
Hist2h4	Mbip	0.908
Hist2h4	Zfp277	0.908
Pias3	Taf1b	0.908
Mll1	Rnf14	0.908
Zfp236	Maml1	0.908
Egr2	Prkcsh	0.908

Egr2	Notch1	0.908
Med1	Nrip1	0.908
H1f0	Cebpa	0.908
Syn2	Acta1	0.908
Git1	Arhgef7	0.908
Polr1b	Ctcf	0.908
Cdk4	Psmd10	0.908
Gab1	Sh3bp2	0.908
Gtf2a1l	Aes	0.907
Lmnb1	Eif3i	0.907
Gtf2h1	Tfdp2	0.907
Cdk5	Ccna2	0.907
Wwp2	Egr2	0.907
Tle1	Ifrd1	0.907
Ndel1	Nefl	0.907
Egr2	Smurf1	0.907
Egr2	Casp8	0.907
Pparg	Sh3gl2	0.907
H1	Ccnb1	0.907
Foxh1	Hoxa13	0.907
Bag1	Ppp1cb	0.906
Mef2a	Eif4enif1	0.906
Myh10	2900073G15	0.906
Gtf2h1	Gtf2f1	0.906
Six1	Neurog1	0.906
Hnf1b	Kat2b	0.906
Rab7	Syne2	0.906
Mixl1	Rbl1	0.906
Mixl1	Hoxc4	0.906
Ncam1	Atp1a3	0.906
Ube2i	Sumo3	0.906
Pparg	Arntl	0.906
Dbp	Clock	0.906
Hist3h2a	Nr1h4	0.906
Gab1	Irs2	0.906
Mef2a	Tnf	0.905
Gpbp1	Hist3h2a	0.905
Irf8	Msx2	0.905
Pcgf2	Spata24	0.905
Hist2h4	Trim27	0.905
Egr2	Skil	0.905
Bcl10	Mib2	0.905
Aldoa	Nfkbia	0.905
Fancd2	Sykb	0.905
Eif4e	Eif2a	0.905
Ifrd1	Rfxank	0.905

Tbrg1	Ppp1r9b	0.905
Spata24	Phc2	0.905
Bach1	Vsx2	0.904
Tnfaip3	Ssbp3	0.904
Psma4	Psmc2	0.904
Rsad2	Irf7	0.904
Als2	Nrip1	0.904
Epc1	Rbm39	0.904
Itch	Junb	0.904
Rnh1	Cttn	0.904
Id1	Abtb1	0.904
Lmnb1	Syne2	0.904
Rai14	Pdlim4	0.904
Pias3	Abt1	0.904
Zap70	Sla	0.904
Egr2	Eif2c2	0.904
Cops8	Cops6	0.904
Prkca	Actn2	0.904
Notch2	Smarcd3	0.904
Pparg	Reg1	0.904
Ubb	Cttn	0.904
Dynll1	Tubb2c	0.904
Ube2d1	Ptma	0.904
Dusp3	Vrk3	0.904
Mef2a	Ncor2	0.903
Map2k4	Mapk8ip1	0.903
Mecp2	Lbxcor1	0.903
Mecp2	Nr1h4	0.903
Shc1	Atxn2	0.903
Als2	Homer2	0.903
Usf1	Kat2a	0.903
Gpbp1	Csk	0.903
Smad9	Btg2	0.903
Rnh1	Yeats2	0.903
Id1	Gtf2h1	0.903
Cd2ap	Sykb	0.903
Gtf2h1	Zfp592	0.903
Hist2h4	Sfn	0.903
Hnf1a	Zfp236	0.903
Cdx1	Dlx1	0.903
Ywhag	Mark3	0.903
Egr2	Trim32	0.903
Aldoa	Zfp110	0.903
Med1	Tbx21	0.903
H1f0	Iqgap1	0.903
Syn2	Eef1a2	0.903

Itpr3	Trpc4	0.903
Map2k4	Peli2	0.902
Psma4	Lox	0.902
Gnb1	Adrbk1	0.902
Rnh1	Ubb	0.902
Id3	Hand1	0.902
Id3	Anxa1	0.902
Sod1	Myh3	0.902
Gtf2h1	Tgfb1i1	0.902
Skp1a	Mcl1	0.902
Egr2	Bcr	0.902
Egr2	Hist2h2bb	0.902
Smurf1	Runx2	0.902
Arrb1	Yes1	0.902
Zfp110	Ngfr	0.902
Ubb	Phf17	0.902
H1f0	Gtf2b	0.902
Fancd2	Fam175b	0.902
Dynll1	Hnrnph2	0.902
Thrb	Ncor1	0.902
Hist3h2a	Zfp277	0.902
Bach1	Zeb2	0.901
Syvn1	Hsp90b1	0.901
Psma4	Ptpn13	0.901
Als2	Cacng2	0.901
Dok2	Nck2	0.901
Rnh1	Hsp90b1	0.901
Id3	Ncoa2	0.901
Hist2h4	Ddx5	0.901
Hist2h4	Psme3	0.901
Tle1	Srf	0.901
5-Sep	7-Sep	0.901
Slc11a1	Phc2	0.901
Pias3	Cxxc1	0.901
Mtf1	Tox3	0.901
Egr2	Mcl1	0.901
Pparg	Fam175b	0.901
Ubb	Spata24	0.901
Fancd2	Junb	0.901
Syn2	Hspa2	0.901
Fasn	Atxn2	0.901
Sf1	Pin1	0.9
Bach1	Smad9	0.9
Bach1	Smurf1	0.9
Map2k4	Fadd	0.9
Epc1	Barx2	0.9

Cd40	Map2k7	0.9
Rnh1	Tfrc	0.9
Irf8	Sh3gl2	0.9
Sirt1	Trp53	0.9
Hnf1a	Hnf4a	0.9
Egr2	Gli2	0.9
Tmf1	Irak1	0.9
Ncam1	Zfp36	0.9
Trib2	Trib3	0.9
Pparg	Gfi1b	0.9
Phc3	Phc2	0.9
H1f0	Map2k7	0.9
Fbxw8	Skp1a	0.899
Bag1	Btrc	0.899
Bag1	Gnao1	0.899
Shc1	Sykb	0.899
Sin3b	Ifrd1	0.899
Id3	Hist2h4	0.899
Id3	Neurog2	0.899
Id3	Rfx4	0.899
Irf8	Flt1	0.899
Id1	Ncoa2	0.899
Hand1	Ascl1	0.899
Rab7	Ttn	0.899
Hist2h4	Neurog2	0.899
Pura	Atp5a1	0.899
Slc11a1	Zfp110	0.899
Slc11a1	Map1lc3a	0.899
Egr2	Fam175b	0.899
Aldoa	Actn4	0.899
Pparg	Ski	0.899
Pparg	Myod1	0.899
Pparg	Jund	0.899
Fancd2	Sh3gl2	0.899
Cask	Sh3gl1	0.899
Tmem115	Mapk8ip1	0.899
Mef2a	Col5a1	0.898
Map2k4	Khdrbs1	0.898
Id3	Pdcd6	0.898
Cd2ap	Dok3	0.898
Rac1	Rps6ka3	0.898
Fbxl2	Calm1	0.898
Lmnb1	Ptma	0.898
Pura	Smn1	0.898
Egr2	Csf1r	0.898
Aldoa	Ticam1	0.898

Sf3b2	Psmc3	0.898
Pparg	Cflar	0.898
Kdm4c	Kdm6b	0.898
Syn2	Trim32	0.898
Gtf3c1	Hoxd13	0.898
Hhex	Fos	0.898
Map2k6	Dusp2	0.897
Gtf2a1l	Pou6f1	0.897
Gnb1	Gng2	0.897
Eme1	Mus81	0.897
Psmb4	Hsp90aa1	0.897
Usp2	Ntrk1	0.897
Sod1	Casp8	0.897
Nr3c2	Stub1	0.897
Zfp369	Siah2	0.897
Pura	Tfdp2	0.897
Slc11a1	Prkcd	0.897
Mll1	Arntl	0.897
Egr2	Smn1	0.897
Atg5	Casp8	0.897
Mixl1	Hoxa11	0.897
Eya3	Irf7	0.897
Ncam1	Atp1a1	0.897
Phf20	Kdm6b	0.897
Notch2	Ryr2	0.897
Capn2	Mdk	0.897
Ifrd1	Pdx1	0.897
Crocc	Mapk8ip1	0.897
Id1	Cnot8	0.896
Gtf2h1	Gtf2h2	0.896
Ascl1	Neurog2	0.896
Tlr4	Ticam1	0.896
Egr2	Pax3	0.896
Egr2	Casp3	0.896
Egr2	Flt1	0.896
Klc1	Mapk8ip3	0.896
Tirap	Trim28	0.896
Syn2	Ppp3ca	0.896
Casp8	Ulk1	0.896
Sorbs1	Rapgef1	0.895
Als2	Grin2c	0.895
Id3	Hist3h2a	0.895
Id3	Acta2	0.895
Id1	Rbl1	0.895
Lmnb1	Rab7	0.895
Lmnb1	Fus	0.895

Gtf2h1	Zfp111	0.895
Ptk2	Gata4	0.895
Tcf12	Ascl3	0.895
Egr2	Msx2	0.895
Birc2	Khdrbs1	0.895
Eya3	Eya1	0.895
Pparg	Fancd2	0.895
Ifrd1	Msx2	0.895
Ube2d1	Cryge	0.895
Bag1	Bcl2l11	0.894
Bach1	Smarcd3	0.894
Mef2a	Hoxd13	0.894
Epc1	Tfdp1	0.894
Itch	Cflar	0.894
Rnh1	Zdhhc13	0.894
Rnh1	Ttn	0.894
Id3	Psme3	0.894
Id1	Tgfb1i1	0.894
Cd2ap	Sorbs2	0.894
Psmb1	Ptpn13	0.894
Usp2	Myh3	0.894
Gtf2h1	Zscan2	0.894
Rab7	Sun1	0.894
Hist2h4	Zfp292	0.894
Hist2h4	Phf2	0.894
Hist2h4	Taf1b	0.894
Zfp354a	Lmx1a	0.894
Max	Hspa1b	0.894
Aldoa	Eif2a	0.894
Pparg	Fyn	0.894
Pparg	Prkcsh	0.894
Limk2	Limk1	0.894
Hoxb13	Dlx5	0.894
Tbrg1	Ttc4	0.894
Lef1	Plagl2	0.894
Nrip1	Nr1h4	0.894
Usf1	Nr1h4	0.893
Id3	Krt15	0.893
Gtf2h1	Dlx5	0.893
Sirt1	Pparg	0.893
Pura	Rplp0	0.893
Pclo	Rapgef4	0.893
Irak4	Il1r1	0.893
Egr2	Ube2n	0.893
Egr2	Psmc3	0.893
Eya3	Neurod1	0.893

Aldoa	Anxa1	0.893
Dynll1	Krt42	0.893
Syn2	Itpr1	0.893
Syn2	Rps3	0.893
Arntl	Dbp	0.893
Gtf3c1	Hoxa10	0.893
Hist3h2a	Ctbp2	0.893
Ifrd1	Hist4h4	0.893
Cask	Ppfia2	0.893
Gtf2a1l	Tle4	0.892
Psma4	Cand2	0.892
Psma4	Hnrnpf	0.892
Epc1	Usf1	0.892
Rarg	Usf1	0.892
Gpbp1	Abt1	0.892
Rnh1	Krt8	0.892
Irf8	Ulk1	0.892
Id1	Hnf4g	0.892
Rac1	Sykb	0.892
Lmnb1	Hist2h4	0.892
Gtf2h1	Kdm5d	0.892
Sirt1	Fos	0.892
Rab3d	Hbb-b1	0.892
Pura	H1f0	0.892
Pura	Arhgap33	0.892
Hnf1a	Hnf4g	0.892
Zfp236	Nr1h4	0.892
Egr2	Cebpa	0.892
Pparg	Smyd1	0.892
Pparg	Cebpb	0.892
Ubb	Krt42	0.892
Ubb	Ttn	0.892
Rbpj	Notch1	0.892
Gab1	Lepr	0.892
Casp8	Flt1	0.892
Bag1	Itch	0.891
Bag1	Krt8	0.891
Bach1	Sf3b2	0.891
Bach1	Sip1	0.891
Mef2a	Dnajb6	0.891
Mecp2	Phf12	0.891
Gtf2a1l	Rnps1	0.891
Sorbs1	Sykb	0.891
Sorbs1	Atxn2	0.891
Lifr	Il6st	0.891
Gpbp1	Irf1	0.891

Gpbp1	Taf1b	0.891
Rnh1	Prss1	0.891
Phb2	Eef1a2	0.891
Gtf2h1	C1qbp	0.891
Gtf2h1	Med31	0.891
Gtf2h1	Zfp764	0.891
Ssbp3	Lmx1b	0.891
Hist2h4	Phf17	0.891
Mtf1	Cebpd	0.891
Mapkapk2	Phc2	0.891
Zfp236	Neurog2	0.891
Pitx1	Pin1	0.891
Egr2	Gsc	0.891
Egr2	Cttn	0.891
Mixl1	Emx2	0.891
Pparg	Irak1	0.891
Pparg	Ctcf	0.891
Pparg	Csf1r	0.891
Ncoa6	Rxra	0.891
Ubb	Gria1	0.891
Casp8	Fas	0.891
Fasn	Lepr	0.891
Rara	Ep300	0.89
Bach1	Kat2b	0.89
Mef2a	Col3a1	0.89
Gtf2a1l	Eya3	0.89
Hdac4	Ifrd1	0.89
Id1	Phb2	0.89
Rev1	Polh	0.89
Rps6kb1	Mtor	0.89
Hist2h4	Hist3h2a	0.89
Hist2h4	Pdlim4	0.89
Pura	Eef1a2	0.89
Tle1	Msx2	0.89
Pias3	Taf5	0.89
Prlr	Agap2	0.89
Gnao1	Ldha	0.89
Birc2	Malt1	0.89
Birc2	Chuk	0.89
Mixl1	Pou6f1	0.89
Aldoa	Ipo9	0.89
Aldoa	Acta1	0.89
Med1	Med16	0.89
Pparg	Usp8	0.89
Pparg	Srebf1	0.89
Pparg	Flt1	0.89

Fancd2	Ngfr	0.89
Fancd2	Cdh1	0.89
Dynll1	Hspa9	0.89
Syn2	Usp8	0.89
Rapgef4	Rims2	0.89
Rela	Jun	0.89
Ubqln2	Eif2a	0.89
Ptma	Cul7	0.89
Tmem115	Tab1	0.89
Lef1	Hoxa13	0.89
Fasn	H2afx	0.89
Foxh1	Hoxa10	0.89
Syvn1	Stx6	0.889
Mef2a	Mef2c	0.889
Gtf2a1l	Stat5b	0.889
Psma4	Atp2a2	0.889
Shc1	Stat3	0.889
Gpbp1	Hnf1b	0.889
Rnh1	Sfn	0.889
Id3	Kdm4b	0.889
Id3	Krt42	0.889
Id1	Tcf12	0.889
Lmo2	Lmo3	0.889
Traf6	Rela	0.889
Rab7	Hsp90b1	0.889
Rps6kb1	Rptor	0.889
Hist2h4	Tsc22d3	0.889
Egr2	Stat5a	0.889
Egr2	Nfkbia	0.889
Egr2	Pparg	0.889
Tle4	Lef1	0.889
Eya3	Neurog1	0.889
Mph1	Cbx7	0.889
Med1	Smad4	0.889
Pparg	Casp3	0.889
Capn2	Metap2	0.889
Ifrd1	Mef2c	0.889
Ppp1cc	Trp53	0.889
Mecp2	Barx2	0.888
Mecp2	Sfpi1	0.888
Carm1	Mef2c	0.888
Als2	Atp1a1	0.888
Als2	Gria1	0.888
Rnh1	Ldha	0.888
Pcmt1	Ppara	0.888
Id3	Zfp277	0.888

Id1	Med31	0.888
Id1	Tbx21	0.888
Cd2ap	Map2k7	0.888
Rac1	Lrp1	0.888
Sod1	Psmc3	0.888
Lmo2	Lmo4	0.888
Cdk5	Ccnh	0.888
Egr2	Pten	0.888
Mixl1	Olig1	0.888
Nbr1	Ubb	0.888
Nefl	Myo5a	0.888
Notch2	Sri	0.888
Pparg	Hist2h2bb	0.888
Pparg	Junb	0.888
Pparg	Flt3	0.888
Ubb	Calm2	0.888
Kif5a	Kcnc1	0.888
Dynll1	Actn4	0.888
Eef1a1	Krt42	0.888
Casp8	Msx2	0.888
Fas	Irf1	0.888
Mef2a	Rfxank	0.887
Als2	Kcnd2	0.887
Epc1	Mllt1	0.887
Ncstn	Rer1	0.887
Rnh1	Hist1h3f	0.887
Id3	Ywhaq	0.887
Id3	Rps27a	0.887
Irf8	Rer1	0.887
Hnf1b	Hnf1a	0.887
Nkx2-5	Hand1	0.887
Traip	Optn	0.887
Hist2h4	Foxp1	0.887
Hist2h4	Hdac5	0.887
Atf4	Fos	0.887
Mtf2	Mms19	0.887
Zfp236	Cebpd	0.887
Birc2	Tirap	0.887
Rb1	Cebpa	0.887
Hbb-b1	Cfl1	0.887
Eef1a1	Acta1	0.887
Zfp277	Lhx2	0.887
Hoxb13	Dlx1	0.887
Ltbr	Ticam1	0.887
Bad	Wasf1	0.886
Mef2a	Zeb2	0.886

Usf1	Clock	0.886
Rnh1	S100a4	0.886
Atf7ip	Ccnh	0.886
Id3	Polr1a	0.886
Cdk5	Nefl	0.886
Slc2a4	Daxx	0.886
Lbxcor1	Tle1	0.886
Hist2h4	Ube2i	0.886
Egr2	Fancd2	0.886
Eya3	Rbl1	0.886
Nbr1	Cttn	0.886
Aldoa	Arrb1	0.886
Pparg	Itpr1	0.886
Ubb	Krt76	0.886
Fancd2	Reg1	0.886
Klrb1c	Nr5a2	0.886
Hspa1b	Ppp1cc	0.886
Zfp277	Zfp592	0.886
Hoxa6	Pknox2	0.886
Tceb1	Wsb1	0.886
Tbrg1	Huwe1	0.886
Mef2a	Pknox1	0.885
Mecp2	Rbl2	0.885
Mecp2	Srebf1	0.885
Psma4	Tfrc	0.885
Gabra1	Gabrg2	0.885
Gpbp1	Sh3gl1	0.885
Rnh1	Ptpn13	0.885
Rnh1	Myo5a	0.885
Rnh1	Spata24	0.885
Id3	Dot1l	0.885
Psmb1	Hnrnpf	0.885
Lmo2	Zfp446	0.885
Six6	Vax2	0.885
Trps1	Zfp236	0.885
Wdr1	Wdr61	0.885
Tcf12	Pdcd6	0.885
Skp1a	Cul7	0.885
Mtf2	Rbl2	0.885
Egr2	Hoxc4	0.885
Mixl1	Trp73	0.885
Ncor2	Ncor1	0.885
Nbr1	Ulk1	0.885
Aldoa	Naca	0.885
Tirap	Ticam1	0.885
Sf3b2	Cblb	0.885

Ubb	Tubb5	0.885
Suz12	E130012A19	0.885
Casp8	Ngfr	0.885
Mef2a	Creb1	0.884
Map2k4	Skil	0.884
Lifr	Cntf	0.884
Zfp513	Zfp473	0.884
Id3	Cops4	0.884
Tcerg1	Sox15	0.884
Gtf2h1	Eya3	0.884
Sfrs3	Eef1a2	0.884
Rab3d	Gnb2l1	0.884
Hist2h4	Olig2	0.884
Pura	Cdk2	0.884
Ahi1	Krt73	0.884
Tle1	Phf12	0.884
Zfp354a	Ankrd32	0.884
Slc11a1	Keap1	0.884
Egr2	Stat1	0.884
Egr2	Atxn3	0.884
Bcl10	Peli2	0.884
Ezh1	Suz12	0.884
Aldoa	Plec	0.884
Aldoa	Psmc3	0.884
Atg16l1	Rab33b	0.884
Zfp473	Hoxd3	0.884
Pparg	Git1	0.884
Phc3	Ppp2ca	0.884
Ubb	Med14	0.884
H1f0	Psme3	0.884
Cdk1	Ccna2	0.884
Arhgap1	Pafah1b1	0.884
Ifrd1	E2f1	0.884
Daxx	Sumo1	0.884
Itpr3	Cav1	0.884
Nrip1	Sap18	0.884
Vezf1	Phf12	0.884
Bach1	Prdm16	0.883
Mecp2	Ncor1	0.883
Psma4	Flnc	0.883
Sorbs1	Itsn1	0.883
Usf1	Hnf1b	0.883
Id1	Map2k1	0.883
Id1	Nkx2-2	0.883
Usp2	Igf1r	0.883
Hist2h4	Hist2h2be	0.883

Tle1	Rbl2	0.883
Tle1	Hdac3	0.883
Nr1i2	Nrip1	0.883
Egr2	Fyn	0.883
Egr2	Itpr1	0.883
Egr2	Runx2	0.883
Eya3	Polr2i	0.883
Rbck1	Eya1	0.883
Aldoa	Irf5	0.883
Aldoa	Actn1	0.883
Pparg	Uimc1	0.883
Il17ra	Traf3ip2	0.883
Capn2	Fert2	0.883
Capn2	Blnk	0.883
Syn2	Hnrnpab	0.883
Bag1	Ppp2ca	0.882
Bag1	Atxn3	0.882
Mapkbp1	Spag9	0.882
Rnh1	Cct6a	0.882
Usp2	Irf5	0.882
Usp2	Zfp110	0.882
Lmnb1	Rab5a	0.882
Pura	Nrip2	0.882
Hnf1a	Irf7	0.882
Slc11a1	Gria3	0.882
Pias3	Srebf1	0.882
Mtf1	Hoxd3	0.882
Gnao1	Gng2	0.882
Egr2	Usp8	0.882
Egr2	Sh3gl2	0.882
Ncam1	Apbb1	0.882
Ncam1	Clu	0.882
Stam2	Usp8	0.882
Aldoa	Syn1	0.882
Nfkbia	Ikbkg	0.882
Atg16l1	Gria3	0.882
Pparg	Dnmt1	0.882
Pparg	Eif2c2	0.882
Pparg	Gli3	0.882
Pparg	Cebpa	0.882
Pparg	Mdm4	0.882
Mafk	Mll2	0.882
Fancd2	Was	0.882
Fancd2	Prkcsh	0.882
Rela	Ctnnb1	0.882
Srf	Hist2h2be	0.882

Rabl4	Hspb11	0.882
Csnk1e	Arntl	0.881
Bag1	Ldha	0.881
Gtf2a1l	Irf7	0.881
Shc1	Ntrk2	0.881
Sorbs1	Arhgap33	0.881
Epc1	Scmh1	0.881
Psmb1	Nap1l4	0.881
Usp2	Mc4r	0.881
Usp2	Sykb	0.881
Usp2	Sh3gl2	0.881
Lmnb1	Sf3b1	0.881
Gtf2h1	Hoxb4	0.881
Gtf2h1	Rnf14	0.881
Rai14	Polr1a	0.881
Hnf1a	Nr2f6	0.881
Hnf1a	Neurog2	0.881
Nr1i2	AW146020	0.881
Pias3	Spata24	0.881
Wdr61	Ctr9	0.881
Sp7	Dlx1	0.881
Mtf1	Mafk	0.881
Mtf1	Sertad1	0.881
Egr2	Irf5	0.881
Egr2	Tax1bp1	0.881
Egr2	Cebpb	0.881
Cyld	Irf5	0.881
Aldoa	Irf7	0.881
Kdm4c	Nr1h4	0.881
Ubb	Acta2	0.881
Fancd2	Msx2	0.881
Fancd2	Flt1	0.881
Capn2	Elk1	0.881
Capn2	Pea15a	0.881
Syn2	Atp5a1	0.881
Syn2	Ppfia2	0.881
Nfe2l2	Fos	0.881
Ifrd1	Gli2	0.881
Casp8	Gli3	0.881
Bag1	Atf3	0.88
Bach1	Ddx5	0.88
Bach1	Runx2	0.88
Rnh1	Ipo9	0.88
Atf7ip	Cdk7	0.88
Id3	Krt2	0.88
Bbs4	Fez1	0.88

Hist2h4	Tle6	0.88
Barx2	Creb1	0.88
Rai14	Ivns1abp	0.88
Gabrb2	Usp14	0.88
Zfp236	Tox3	0.88
Egr2	Dlx5	0.88
Eya3	Tbx21	0.88
Sf3b2	Klf10	0.88
Med1	Tbp	0.88
Pparg	Was	0.88
Pparg	Aes	0.88
Pparg	Mycn	0.88
Ubb	Med16	0.88
Ubb	Gsn	0.88
Tek	Irs2	0.88
Fancd2	Mc4r	0.88
Fancd2	Psmc3	0.88
Syn2	Git1	0.88
Pcna	Cdk2	0.88
Ptma	Jund	0.88
Tbrg1	Epas1	0.88
Fasn	Sap18	0.88
Fasn	Vav1	0.88
Nkd2	Zscan2	0.88
Zfp263	Sp7	0.879
Mef2a	Hoxa13	0.879
Gtf2a1l	Sp7	0.879
Sorbs3	Kndc1	0.879
Clip1	Pafah1b1	0.879
Als2	Nrcam	0.879
Rnh1	Hist1h2ai	0.879
Id1	Kat2b	0.879
Cd2ap	Dnajb6	0.879
Gtf2h1	Hcls1	0.879
Taf3	Hmga2	0.879
Traf6	Ywhaz	0.879
Pura	Sorbs2	0.879
Zfp354a	Lmo3	0.879
Otx2	Hoxb13	0.879
Slc11a1	Gria2	0.879
Slc11a1	Sf3b1	0.879
Mll1	Hist4h4	0.879
Otud7b	Ltbr	0.879
Tmf1	Cflar	0.879
Nbr1	Sh3gl2	0.879
Ube2e3	Cul7	0.879

Notch2	Aph1a	0.879
Notch2	Cdh1	0.879
Ubb	Tubb2a	0.879
Robo1	Slit3	0.879
H1f0	Nr3c1	0.879
H1f0	Atxn2	0.879
Dynll1	Hsp90aa1	0.879
Syn2	Lrrk2	0.879
Grb10	Irs2	0.879
Sun1	Syne2	0.879
Ubqln2	Telo2	0.879
Ubqln2	Psmc5	0.879
Hhex	Sox8	0.879
Mapk8ip1	Tab1	0.879
Cebpa	Psmd10	0.879
Mecp2	Polr1a	0.878
Clip1	Iqgap1	0.878
Lifr	Ctf2	0.878
Ubtf	Ctcf	0.878
Irf8	Cdh1	0.878
Id1	Trps1	0.878
Cd2ap	Atm	0.878
Psmb1	Hnrnpm	0.878
Tcerg1	Phf17	0.878
Atp6v1a	Actn1	0.878
Rab7	Sfn	0.878
Hist2h4	Dnmt1	0.878
Pura	Tgif1	0.878
Pura	Psmd10	0.878
Max	Ppp1r9b	0.878
Mtf2	Ezh1	0.878
Dlg3	Dlg1	0.878
Zfp236	Hoxa1	0.878
Ezh1	Aebp2	0.878
Ppard	Pawr	0.878
Pparg	Rbl2	0.878
Pparg	Wdtc1	0.878
Ubb	Try10	0.878
Ubb	Krt78	0.878
Blnk	Metap2	0.878
Lmb1	Eef1a2	0.878
Dvl1	Musk	0.877
Zfp263	Cdx1	0.877
Bag1	Syvn1	0.877
Bag1	Barx2	0.877
Bach1	Cops5	0.877

Mef2a	Myocd	0.877
Psma4	Psmb1	0.877
Sorbs3	Mdk	0.877
Sorbs1	Hck	0.877
Pik3r2	Gab2	0.877
Hist1h1e	Cbx5	0.877
Epc1	Mapkapk2	0.877
Rad51	Olig1	0.877
Cited4	Hoxd3	0.877
Rnh1	Mms19	0.877
Id1	Dot1l	0.877
Rev1	Polk	0.877
Rev1	Mad2l2	0.877
Usp2	Cflar	0.877
Usp2	Tbk1	0.877
Tcf4	Hand2	0.877
Hand1	Tcf12	0.877
Rab7	Gm5414	0.877
Atf4	Fam175b	0.877
Slc11a1	Ntrk1	0.877
Efs	Clybl	0.877
Sox2	Hoxc4	0.877
Egr2	Irak1	0.877
Mixl1	Eya1	0.877
Aldoa	Dynll1	0.877
Pparg	Thrb	0.877
Pparg	Pten	0.877
Pparg	Rhoa	0.877
Pparg	Uchl1	0.877
Pparg	Rer1	0.877
Phc3	H3f3a	0.877
Ubb	Trim27	0.877
H1f0	H1	0.877
H1f0	Suz12	0.877
Syn2	Arhgap33	0.877
Hist3h2a	Lass2	0.877
Casp8	Tbk1	0.877
Bad	Gck	0.876
Bag1	Foxp1	0.876
Bag1	Fos	0.876
Bach1	Gjb6	0.876
Syvn1	Rer1	0.876
Map2k6	Mib2	0.876
Als2	Grin2a	0.876
Als2	Actn2	0.876
Gpbp1	Kat2b	0.876

Cited4	Gmeb1	0.876
Meis2	Pbx4	0.876
Id1	Tcf4	0.876
Usp2	Junb	0.876
Sod1	Cttn	0.876
Pura	Ccne1	0.876
Ahi1	Krt76	0.876
Egr2	Lyn	0.876
Egr2	Gli3	0.876
Anapc7	Crem	0.876
Tmf1	Phf20	0.876
Aldoa	Krt16	0.876
Aldoa	Trim28	0.876
Aldoa	Hspa2	0.876
Pparg	Skil	0.876
Pparg	Hgs	0.876
Irak1	Reg1	0.876
Fancd2	Hist2h2bb	0.876
Fancd2	Gli3	0.876
Fancd2	Rer1	0.876
Cdk1	Ppp1ca	0.876
Tceb2	Nrbp1	0.876
Gab1	Csn2	0.876
Ksr1	Mark3	0.875
Bag1	Usp9x	0.875
Bag1	Itpr1	0.875
Bag1	Hhex	0.875
Mef2a	Pex6	0.875
Mef2a	Ifrd1	0.875
Mecp2	Mms19	0.875
Gtf2a1l	Vax2	0.875
Epc1	Cbx6	0.875
Trim26	Kdm6b	0.875
Rarg	Nr1h4	0.875
Gpbp1	Cited2	0.875
Gpbp1	Sertad1	0.875
Stk38	Mtf2	0.875
Rnh1	Actn4	0.875
Id1	Tle6	0.875
Rac1	Akap9	0.875
Usp2	Prkcsh	0.875
Zfp354a	Lmo1	0.875
Hnf1a	Tlx1	0.875
Pias3	Pparg	0.875
Egr2	Ppp2cb	0.875
Egr2	Dlg4	0.875

Eya3	Pbx2	0.875
Sf3b2	Smarcd3	0.875
Pparg	Nf1	0.875
Pparg	Psmc3	0.875
Pparg	Vdac1	0.875
H1f0	Uchl1	0.875
Fancd2	Cttn	0.875
Cdk1	Ccnb1	0.875
Suz12	Iqgap1	0.875
Numb	Ctnna1	0.875
Tceb1	Nrbp1	0.875
Cdk4	Cebpa	0.875
Gab1	Fos	0.875
Mapk7	Nod2	0.875
Foxh1	Prdm16	0.875
Cit	Rac1	0.874
Bag1	Snap25	0.874
Bag1	Hdac6	0.874
Bag1	Ppp1ca	0.874
Mecp2	Tle6	0.874
Mecp2	Pdx1	0.874
Gtf2a1l	Tsc22d3	0.874
Als2	Gria3	0.874
Usf1	Pitx2	0.874
Gpbp1	Tceb3	0.874
Cited4	Polr1b	0.874
Rnh1	2210010C04	0.874
Rnh1	Lox	0.874
Id3	Rsrc1	0.874
Cd2ap	Iqgap1	0.874
Sod1	Hsp90aa1	0.874
Lmnb1	Sfn	0.874
Gtf2h1	Tle6	0.874
Traf6	Stat3	0.874
Rab3d	Slc25a3	0.874
App	Dlg1	0.874
Hnf1a	Asb15	0.874
Slc11a1	Fau	0.874
Max	Cdkn2a	0.874
Mtf2	Srebf1	0.874
Egr2	Reg1	0.874
Cenpv	Ldhb	0.874
Aldoa	Irak1	0.874
Smurf1	Map3k2	0.874
Med1	Runx1t1	0.874
Ppard	Nr1i3	0.874

Kdm4c	E2f1	0.874
Phc3	E2f6	0.874
Tek	Csf1r	0.874
Syn2	Gab2	0.874
Foxh1	Olig1	0.874
Fau	Gria3	0.874
Ksr1	Brap	0.873
Bag1	Ywhag	0.873
Mef2a	Sf3b2	0.873
Mef2a	Rnf111	0.873
Map2k6	Malt1	0.873
Mecp2	Xpo1	0.873
Psma4	Nap1l4	0.873
Epha2	2210010C04I	0.873
Epc1	Sertad1	0.873
Usf1	Tcf12	0.873
Gpbp1	Gmnn	0.873
Rad51	Fancd2	0.873
Rad51	Atxn2	0.873
Rnh1	Atp2a2	0.873
Id2	Trim27	0.873
Id3	Tcf4	0.873
Irf8	Flt3	0.873
Psmb1	Lox	0.873
Psmb1	Tfrc	0.873
Usp2	Stam2	0.873
Gtf2h1	Med21	0.873
Gtf2h1	Pbx4	0.873
Gtf2h1	AW146020	0.873
Tcf12	Cops6	0.873
Tcf12	Hist3h2a	0.873
Pias3	Gtf2b	0.873
Inadl	Grin2c	0.873
Mapt	Egr2	0.873
Mtf1	Hoxa2	0.873
Mtf2	Bclaf1	0.873
Egr2	Rhoa	0.873
Egr2	Mycn	0.873
Tmf1	Myog	0.873
Eya3	Cnot8	0.873
Nbr1	Flt3	0.873
Cyld	Casp8	0.873
Aldoa	Hgs	0.873
Aldoa	Hspa9	0.873
Tirap	Ptk2b	0.873
Ncoa6	Hist3h2a	0.873

Ncoa6	Sf3b1	0.873
Mapk1	Cttn	0.873
Sf3b4	2500003M1(	0.873
Srf	Tbl1x	0.873
Ptrf	Eif3e	0.873
Il6st	Cntf	0.873
Fasn	Gab2	0.873
Bag1	Eif2c2	0.872
Syvn1	Aph1b	0.872
Mef2a	Bahd1	0.872
Map2k6	Tmem115	0.872
Mecp2	Kat5	0.872
Sorbs3	Rabgef1	0.872
Usf1	Notch1	0.872
Rnh1	Pkd1	0.872
Rnh1	Nphp3	0.872
Rnh1	Ankhd1	0.872
Id3	Tcf12	0.872
Sod1	Keap1	0.872
Gtf2h1	Hif1an	0.872
Gtf2h1	Foxn2	0.872
Gtf2h1	Mms19	0.872
Gtf2h1	Supt4h1	0.872
Gtf2h1	Zfp277	0.872
Gtf2h1	Asb1	0.872
Cdk5	Cdk2	0.872
Wbp11	Eif2a	0.872
Hist2h4	Hdac3	0.872
Pura	Grip1	0.872
Zfp354a	Srf	0.872
Pias3	Ube2i	0.872
Pias3	Hdac6	0.872
Mapt	Myh3	0.872
Mapt	Rnf19a	0.872
Egr2	Birc3	0.872
Egr2	Nfkb1	0.872
Egr2	Rer1	0.872
Egr2	Hras1	0.872
Tmf1	Tnk2	0.872
Nbr1	Cdh1	0.872
Pparg	Cdk7	0.872
Ncoa6	Phc2	0.872
Fancd2	Ulk1	0.872
Ptpn6	Tcrb	0.872
Hist3h2a	Hdac10	0.872
Casp8	Junb	0.872

Fbxw8	Rbx1	0.871
Bad	Prkaca	0.871
Bag1	Park2	0.871
Map2k4	Map2k1	0.871
Psma4	Hnrnph1	0.871
Sorbs3	Brap	0.871
Sorbs1	Grb10	0.871
Usf1	Ubtf	0.871
Cited4	Cebpd	0.871
Vps16	Vps45	0.871
Irf8	Cttn	0.871
Ss18	Atf2	0.871
Psmd7	Zfand2a	0.871
Lmnb1	Serbp1	0.871
Itgb1bp2	Chordc1	0.871
Mtf2	Ell	0.871
Egr2	Stat3	0.871
Egr2	Cacna1c	0.871
Egr2	Myd88	0.871
Pcgf1	Hist3h2a	0.871
Eya3	Med31	0.871
Nbr1	Ntrk1	0.871
Aldoa	Eif2c2	0.871
Tcp1	Calm1	0.871
Pparg	Satb2	0.871
Ubb	Fbxo2	0.871
Kif5a	Kif5b	0.871
Fancd2	Eif2c2	0.871
Foxp1	Phf2	0.871
Suz12	Ldha	0.871
Ifrd1	Cebpb	0.871
Ifrd1	Runx2	0.871
Zfp277	Rnf14	0.871
Mapk7	Pgr	0.871
Tbp	Taf1b	0.871
Vezf1	Hist3	0.871
Csnk1e	Per1	0.87
Bag1	Stub1	0.87
Bag1	Ppp3ca	0.87
Bach1	Pex6	0.87
Syvn1	Ppp1ca	0.87
Mef2a	Lef1	0.87
Psma4	Krt78	0.87
Shc1	Flt1	0.87
Epc1	Phc3	0.87
Gpbp1	Pias1	0.87

Rad51	Map2k7	0.87
Cited4	Rbl2	0.87
Cited4	Ell	0.87
Arhgef2	Ppp1r9b	0.87
Per1	Clock	0.87
Cd2ap	Psme3	0.87
Psmb1	Glud1	0.87
Sod1	Ntrk2	0.87
Lmo2	Lmo1	0.87
Gtf2h1	Ciita	0.87
Gtf2h1	Atf7	0.87
Tcfap2a	Cited2	0.87
Zfp354a	Lhx2	0.87
Bcl6	Rxra	0.87
Esr1	Rela	0.87
Dlg3	Ppp1cc	0.87
Egr2	Hgs	0.87
Egr2	Tardbp	0.87
Egr2	Dlg1	0.87
Egr2	Fos	0.87
Tmf1	Zfp110	0.87
Eya3	Nr3c1	0.87
Nbr1	Irf5	0.87
Nbr1	Fam175b	0.87
Prkca	Ubqln2	0.87
Siah2	Thrb	0.87
Siah2	Spry2	0.87
Hsf1	Irf3	0.87
Aldoa	Usp8	0.87
Leo1	Ctr9	0.87
Atg16l1	Fau	0.87
Sf3b2	Runx2	0.87
Notch2	Dll1	0.87
Pparg	Hsp90aa1	0.87
Pparg	Arid1b	0.87
Ubb	Pi4ka	0.87
Ubb	Myo5a	0.87
Polr1e	Ctcf	0.87
Fancd2	Ctcf	0.87
Syn2	Tubb2a	0.87
Gtf3c1	Foxh1	0.87
Suz12	Psmc2	0.87
Hist3h2a	Myb	0.87
Ubqln2	Ldha	0.87
Casp8	Rer1	0.87
Bag1	Rad9	0.869

Map2k6	Spag9	0.869
Map2k6	Tab1	0.869
Mecp2	Pparg	0.869
Sorbs1	Snx9	0.869
Als2	Lrp1	0.869
Epha2	Krt79	0.869
Rad51	Sap18	0.869
Cited4	Hoxa1	0.869
Cited4	Zfp592	0.869
Ncstn	Aph1a	0.869
Dcx	Usp9x	0.869
Rnh1	Tubb2a	0.869
Per2	Mll1	0.869
Id3	Trim27	0.869
Ssbp2	Lmo3	0.869
Gtf2h1	Rbl1	0.869
Sfrs3	Lmb1	0.869
Apba1	Ppfia2	0.869
Gata4	Gata6	0.869
Traf6	Trp53	0.869
Rps6kb1	Mlst8	0.869
Pura	Thoc4	0.869
Pura	Nrip1	0.869
Pura	Cdk7	0.869
Wdr61	Leo1	0.869
Psen2	Notch3	0.869
Krt8	Hsp90aa1	0.869
Zfp236	Sertad1	0.869
Wasf1	Gck	0.869
Egr2	Dnmt1	0.869
Eya3	Stat5b	0.869
Aldoa	Snap25	0.869
Aldoa	Krt78	0.869
Notch2	Grin2a	0.869
Pparg	Jak2	0.869
Pparg	Cdh1	0.869
Ncoa6	Scmh1	0.869
Ubb	Taf15	0.869
Kif5b	Kcnc1	0.869
H1f0	Ppp1cc	0.869
Dynll1	Actn1	0.869
Syn2	Rims2	0.869
Ube2d1	Dll1	0.869
Casp8	Prkcsh	0.869
Nrip1	Sirt2	0.869
Bag1	Sfpi1	0.868

Map2k6	Mapk8ip1	0.868
Mecp2	Atrx	0.868
Gtf2a1l	Tlx1	0.868
Sorbs1	Nck1	0.868
Als2	Kat2b	0.868
Rarg	Atf2	0.868
Usf1	Atf2	0.868
Gpbp1	Clock	0.868
Rnh1	Cryab	0.868
Id2	Atm	0.868
Id1	Foxn2	0.868
Pygo1	Lhx9	0.868
Usp2	Ndfip1	0.868
Lmnb1	Dnmt1	0.868
Lmnb1	Ttn	0.868
Crkl	Inpp5d	0.868
Rab3d	Dnajb1	0.868
Pura	Gab2	0.868
Rai14	Lhx9	0.868
Hnf1a	Spdef	0.868
Nr1i2	Ptpn6	0.868
Slc11a1	Ubb	0.868
Ar	Kdm4c	0.868
Dnajb6	Bahd1	0.868
Egr2	Flt3	0.868
Tirap	Csf1r	0.868
Pparg	Itga5	0.868
Pparg	Dlg4	0.868
Kdm4c	Tbx21	0.868
Il1r1	Il1rap	0.868
Irak1	Myd88	0.868
Fancd2	Pten	0.868
Fancd2	Casp8	0.868
Fancd2	Notch1	0.868
Hist3h2a	Sh3gl1	0.868
Tbrg1	Sobp	0.868
Bag1	Mllt3	0.867
Bach1	Ercc3	0.867
Syvn1	Psen2	0.867
Syvn1	Aph1a	0.867
Gpbp1	Zbtb17	0.867
Gpbp1	Myb	0.867
Cited4	Pcmt1	0.867
Rnh1	Rsrc1	0.867
Id3	Shank3	0.867
Irf8	Mdm4	0.867

Cd2ap	Msl1	0.867
Map2k1	Brap	0.867
Gtf2h1	Pdlim4	0.867
Traf3	Otud7b	0.867
Rab6	Rab5a	0.867
Hist2h4	Polr1a	0.867
Tle1	Pdx1	0.867
Nrg1	Brm1	0.867
Zfp354a	Hif1an	0.867
Nr1i2	Foxh1	0.867
Keap1	Zbtb16	0.867
Esr1	Stat3	0.867
Max	Ccnb1	0.867
Zfp236	Ncor2	0.867
Egr1	Pin1	0.867
Egr2	Uchl1	0.867
Ube2l3	Rnf31	0.867
Hbb-b1	Csf2rb	0.867
Aldoa	Lox	0.867
Sf3b2	Bmpr2	0.867
Srf	Tfdp1	0.867
Ifrd1	Phf12	0.867
Ubqln2	Eif3e	0.867
Ube2d1	Ube2g2	0.867
Mecp2	Hist3h2a	0.866
Mecp2	Ctcf	0.866
Shc1	Gab2	0.866
Epc1	Pcgf2	0.866
Gpbp1	Hoxa9	0.866
Rnh1	Acta2	0.866
Id2	Hmga1	0.866
Id3	Irf7	0.866
Ankrd1	Capn3	0.866
Sod1	Pkm2	0.866
Gtf2h1	Otx2	0.866
Gtf2h1	Mafk	0.866
Tcf4	Ascl3	0.866
Lbxcor1	Zbtb16	0.866
Rab7	Ube2i	0.866
Zfp369	Ngfr	0.866
Pura	Ccnb1	0.866
Rai14	Rfx4	0.866
Max	Hes6	0.866
Egr2	Snap25	0.866
Mixl1	Hoxd3	0.866
Nbr1	Dlg1	0.866

Trib2	Rbl1	0.866
Akr1b3	S100a4	0.866
Tirap	Vav1	0.866
Pparg	Myd88	0.866
Pparg	Tax1bp1	0.866
Pparg	Hras1	0.866
Pparg	Lass2	0.866
Kdm4c	Lhx2	0.866
Fancd2	Hdac6	0.866
Fancd2	Mycn	0.866
Dynll1	Plec	0.866
Cdx4	Dlx1	0.866
Inppl1	Ppp2r3a	0.866
Suz12	Eif2c2	0.866
Suz12	Ell	0.866
Cask	Dlg1	0.866
Mef2a	Prdm16	0.865
Mecp2	Myod1	0.865
Tnf	Tnfrsf1a	0.865
Tnfaip3	Ube2n	0.865
Gtf2a1l	Hnf4g	0.865
Shc1	Vav1	0.865
Sorbs1	Sla	0.865
Sorbs1	Ticam1	0.865
Rarg	Kat2a	0.865
Usf1	Kat2b	0.865
Cited4	Maml1	0.865
Rnh1	Krt14	0.865
Sgca	Cryab	0.865
Actbl2	Prss1	0.865
Per1	Arntl	0.865
Id3	Tlx1	0.865
Irf8	Mdm2	0.865
Id1	Sirt2	0.865
Cd2ap	Gtf2b	0.865
Rac1	Eif2c2	0.865
Usp2	Smn1	0.865
Sod1	Sykb	0.865
Sod1	Tbk1	0.865
Lmnb1	Zbtb16	0.865
Lmnb1	Sun1	0.865
Sirt1	Jun	0.865
Hist2h4	Taf15	0.865
Hist2h4	Brca1	0.865
Atf4	Jun	0.865
Slc11a1	Gria1	0.865

Slc11a1	Casp8	0.865
Grip1	Nrip1	0.865
Bcl6	Zfp111	0.865
Sox2	Jun	0.865
Mapt	Hdac6	0.865
Egr1	Sox9	0.865
Mixl1	Gtf3c1	0.865
Mixl1	Runx1t1	0.865
Tmf1	Ntrk1	0.865
Nbr1	Ngfr	0.865
Tbpl1	Spata24	0.865
Cenpv	Mllt1	0.865
Tlx1	Meis1	0.865
Hbb-b1	Dnajb1	0.865
Cyld	Ikbkg	0.865
Aldoa	Cycs	0.865
Pparg	Mdm2	0.865
Phc3	Zbtb33	0.865
Ubb	Ccar1	0.865
Ubb	Hsp90aa1	0.865
Fancd2	Mdm4	0.865
Syn2	Actr1a	0.865
Syn2	Pfn1	0.865
Gtf3c1	Dlx2	0.865
Hist3h2a	Gtf2b	0.865
Hist3h2a	Sertad1	0.865
Hist3h2a	Sfn	0.865
Ifrd1	Hdac5	0.865
Ubqln2	Psmc3	0.865
Fas	Uimc1	0.865
Fas	Ube3a	0.865
Bach1	Gata4	0.864
Map2k6	Map2k4	0.864
Gtf2a1l	Dlx1	0.864
Psma4	Naglu	0.864
Psma4	Krt77	0.864
Shc1	Dok3	0.864
Gabra1	Gabrb2	0.864
Uhrf1	Eme1	0.864
Epc1	Cbx7	0.864
Cited4	Zbtb9	0.864
Rnh1	Cand2	0.864
Rnh1	Trim28	0.864
Rnh1	Eef1a1	0.864
Id3	Krt73	0.864
Irf8	Gli2	0.864

Psmb4	Mitf	0.864
Mafb	Taf5	0.864
Sod1	Sh3gl2	0.864
Ift81	1500035H01	0.864
Sirt1	Rela	0.864
Rab3d	Ldhb	0.864
Hist2h4	Prpf31	0.864
Pura	Fbxo4	0.864
Skp1a	Skp2	0.864
Slc11a1	Cbx7	0.864
Ccnd3	Cdk4	0.864
Adcy3	Flot1	0.864
Mapt	Smn1	0.864
Egr2	Cdkn1a	0.864
Egr2	Ulk1	0.864
Mixl1	Foxh1	0.864
Tmf1	Fam175b	0.864
Nbr1	Runx2	0.864
Nbr1	Ddx41	0.864
Prkca	Prkcc	0.864
Aldoa	Krt42	0.864
Neurog2	Olig2	0.864
Ppard	Ppara	0.864
Pparg	Ccar1	0.864
Pparg	Rela	0.864
Pparg	Dlg1	0.864
Kdm4c	Pdlim4	0.864
Syn2	Capza1	0.864
Nr1i3	Ppp2ca	0.864
Ppp1cc	Cdkn2a	0.864
Ubqln2	Hspa9	0.864
Csnk1e	Apc	0.863
Mapkbp1	Dusp2	0.863
Mecp2	Trps1	0.863
Mecp2	Kat2b	0.863
Gtf2a1l	Nr3c1	0.863
Shc1	Cebpb	0.863
Sorbs1	Gata4	0.863
Col5a1	Smad2	0.863
Cited4	Hnf1b	0.863
Rnh1	Kpnb1	0.863
Id3	Cops8	0.863
Id1	Cops4	0.863
Id1	Jun	0.863
Gtf2h1	Nkx2-2	0.863
Gtf2h1	Sirt2	0.863

Fadd	Ripk1	0.863
Traf2	Ube2n	0.863
Pura	Fzr1	0.863
Nr1i2	Ppard	0.863
Nr1i2	Mef2c	0.863
Slc11a1	Ulk1	0.863
Pias3	Fem1a	0.863
Pias3	Naca	0.863
Esr1	Arid1b	0.863
Tlr4	Irak1	0.863
Efr3b	Pi4ka	0.863
Mtf1	Zfp236	0.863
Egr2	Vim	0.863
Mixl1	Dlx1	0.863
Tmf1	Reg1	0.863
Cyld	Junb	0.863
Aldoa	Hnrnph2	0.863
Pparg	Map3k2	0.863
Ncoa6	Tfdp1	0.863
H1f0	Gtf3c1	0.863
Chordc1	Hsp90aa1	0.863
H1	Ccna1	0.863
Hba-a1	Atp5d	0.863
Srf	Fos	0.863
Suz12	Dnajb1	0.863
Hist3h2a	Zfp592	0.863
Zfp277	Zscan2	0.863
Tbrg1	Uqcrc1	0.863
Map2k6	Birc2	0.862
Mecp2	Smarca1	0.862
Mecp2	Hist4h4	0.862
Als2	Gria2	0.862
Krt10	2210010C04I	0.862
Cited4	Stk38	0.862
Cited4	Nr1h4	0.862
Rnh1	Dynll1	0.862
Cd2ap	Flt1	0.862
Rac1	Ppp5c	0.862
Phb2	Sfrs3	0.862
Eif3b	Cfl1	0.862
Sod1	Polk	0.862
Sod1	Atp5a1	0.862
Gtf2h1	Cnot8	0.862
Gtf2h1	H3f3a	0.862
Gata4	Smad2	0.862
Pura	Git1	0.862

Ring1	Phc2	0.862
Tle1	Setx	0.862
Tcf12	Trim27	0.862
Pias3	Pax6	0.862
Ccnd1	Fbxo4	0.862
Ctnnd1	Cdh1	0.862
Rnf19a	Pias1	0.862
Egr2	Tmf1	0.862
Egr2	Nbr1	0.862
Egr2	Cblb	0.862
Egr2	Jun	0.862
Cenpv	Dnajb1	0.862
Aldoa	Eef1a1	0.862
Aldoa	Tubb5	0.862
Tirap	Tceb1	0.862
Pparg	Itpr3	0.862
Pparg	Casp8	0.862
Pparg	Sox8	0.862
Pparg	Nos2	0.862
Pparg	Gli2	0.862
Fancd2	Bcr	0.862
Dynll1	Eef1a1	0.862
Cops6	Cops4	0.862
Hba-a1	Hspa9	0.862
Casp8	Sh3gl2	0.862
Nrip1	Vdr	0.862
Notch1	Smarcd3	0.862
Bag1	Mafb	0.861
Mef2a	Gata4	0.861
Tnf	Ripk1	0.861
Gtf2a1l	Kdm4b	0.861
Sorbs1	Sh3kbp1	0.861
Sorbs1	Zap70	0.861
Sorbs1	Inppl1	0.861
Gabra1	Ppp3ca	0.861
Epc1	Ncoa1	0.861
Epc1	Kat2a	0.861
Epc1	Myod1	0.861
Bcl11b	Suz12	0.861
Irf8	Tax1bp1	0.861
Usp2	Ntrk2	0.861
Six3	Eya1	0.861
Nkx2-5	Hand2	0.861
Nod1	Chordc1	0.861
Traf6	Myd88	0.861
Rab5b	Dvl3	0.861

Pura	Cdk4	0.861
Pura	Cebpa	0.861
Ascl1	Tcf12	0.861
Pias3	Nfkbia	0.861
Egr1	Bahd1	0.861
Egr2	Smad2	0.861
Egr2	Mdm4	0.861
Atg5	Rab33b	0.861
Nbr1	Ntrk2	0.861
Nbr1	Reg1	0.861
Prkca	Mcl1	0.861
Akr1b3	Eprs	0.861
Med1	Smarcd3	0.861
Pparg	Cttn	0.861
Ubb	Hnrnpf	0.861
Ubb	Sfn	0.861
Ubb	Plec	0.861
H1f0	Pcna	0.861
H1f0	Vav1	0.861
Fancd2	Uimc1	0.861
Dynll1	Krt76	0.861
H1	Cdk1	0.861
Map3k5	Rassf1	0.861
Itpr1	Ufd1l	0.861
Gtf3c1	Atxn2	0.861
Suz12	Atxn2	0.861
Hist3h2a	Psme3	0.861
Hdac3	Ncor1	0.861
Casp3	Slc1a2	0.861
Fbxw8	Cul7	0.86
Ksr1	Map2k1	0.86
Bag1	Kcnb1	0.86
Bach1	Klf10	0.86
Map2k4	Sash1	0.86
Map2k4	Traf3ip2	0.86
Mecp2	Nkd2	0.86
Gtf2a1l	Vdr	0.86
Psma4	Actn4	0.86
Shc1	Itch	0.86
Shc1	Csf1r	0.86
Usf1	Myod1	0.86
Rad51	H2afx	0.86
Irf9	Pou2f1	0.86
Id3	Dsg1a	0.86
Id1	Hif1a	0.86
Cd2ap	Erbb2	0.86

Cd2ap	Inpp5d	0.86
Raf1	Akap9	0.86
Rac1	Actn2	0.86
Sod1	Hspa5	0.86
Traf6	Irf5	0.86
Pura	Ccnd2	0.86
Pura	Fus	0.86
Nr1i2	Polr3f	0.86
Ywhag	Lrrk2	0.86
Egfr	Ptpn11	0.86
Esr1	Pax3	0.86
Mtf2	Rpl28	0.86
Ncoa2	Mef2c	0.86
Egr2	Ntrk2	0.86
Egr2	Hdac5	0.86
Tmf1	Fancd2	0.86
Magi2	Pex6	0.86
Nbr1	Sykb	0.86
Ncam1	Dnm1	0.86
Stam2	Hgs	0.86
Pparg	Spry2	0.86
Pparg	Ppp2cb	0.86
Pparg	Notch1	0.86
Ppargc1a	Ncor1	0.86
Ubb	Hspa2	0.86
Fancd2	Atxn3	0.86
Eef1a1	Hsp90aa1	0.86
Blnk	Vrk3	0.86
Brca1	Nbn	0.86
Cask	Sh3gl2	0.86
Fas	Mdm2	0.86
Foxh1	Vdr	0.86
Sf1	Pitx1	0.859
Bad	Sox9	0.859
Bach1	Smad5	0.859
Mef2a	Itch	0.859
Mef2a	Ddx5	0.859
Map2k6	Stk39	0.859
Map2k6	Tnip3	0.859
Map2k6	Traf3ip2	0.859
Spna2	Ywhab	0.859
Gtf2a1l	Pknox2	0.859
Psma4	Actn1	0.859
Sorbs3	Rfxank	0.859
Epc1	Tcf12	0.859
Rad51	Psme3	0.859

Actbl2	Hnrnpa2b1	0.859
Cry1	Arntl	0.859
Id2	Hmga2	0.859
Id2	Myod1	0.859
Id2	Rnf14	0.859
Id3	Actc1	0.859
Id1	Gps1	0.859
Col3a1	Smad4	0.859
Psmb5	Ngfr	0.859
Psmb1	Atp2a2	0.859
Usp2	Was	0.859
Usp2	Pten	0.859
Usp2	Eif2c2	0.859
Sod1	Eif2a	0.859
Lmo2	Lhx5	0.859
Lmo2	Lhx4	0.859
Gtf2h1	Kat2b	0.859
Gtf2h1	Hif1a	0.859
Atp6v1a	Actn4	0.859
Gata4	Smarcd3	0.859
Ssbp3	Med16	0.859
Rab3d	Tollip	0.859
Pura	Kat2a	0.859
Hnf1a	Zdhhc13	0.859
Pias3	Hsf1	0.859
Mtf2	Trim35	0.859
Ncoa3	Sap18	0.859
Lbr	Hist4h4	0.859
Tmf1	Skil	0.859
Nbr1	Ski	0.859
Hbb-b1	Fbln2	0.859
Ppargc1a	Fos	0.859
Ncoa6	E2f6	0.859
Ubb	Mysm1	0.859
Dynll1	Gcn1l1	0.859
Syn2	Aph1a	0.859
Fyn	Csf1r	0.859
Eef1a1	Capzb	0.859
Rbpj	Smarcd3	0.859
Gria2	Gria3	0.859
Sumo1	Mdm2	0.859
Bag1	Dnajb1	0.858
Map2k6	Mapkapk2	0.858
Map2k4	Tnip3	0.858
Mecp2	Msx2	0.858
Mecp2	Cebpa	0.858

Bcl2l11	Bcl2	0.858
Psma4	Eprs	0.858
Als2	Accn1	0.858
Rarg	Kat2b	0.858
Cited4	Tox3	0.858
Cited4	Rfxank	0.858
Sgca	Ahnak	0.858
Cry1	Clock	0.858
Per2	Arntl	0.858
Per2	Clock	0.858
Id3	Krt14	0.858
Id1	Sox8	0.858
Cd2ap	Atr	0.858
Homer2	Grm5	0.858
Psmb1	Dnaja3	0.858
Usp2	Polk	0.858
Sod1	Fancd2	0.858
Gtf2h1	E4f1	0.858
Rab5b	Srebf2	0.858
Zfp369	Sertad1	0.858
Hist2h4	Stub1	0.858
Hist2h4	Zzz3	0.858
Tle1	Taz	0.858
Atf4	Egln3	0.858
Nr1i2	Cebpd	0.858
Adrbk1	Gng2	0.858
Mapt	Lbr	0.858
Zfp236	Hoxa2	0.858
Ctf2	Il6st	0.858
Mixl1	Ifnar2	0.858
Tmf1	Pou6f1	0.858
Aldoa	Vdac1	0.858
Aldoa	Prkcsh	0.858
Akr1b3	Prdm16	0.858
Tirap	Il1r1	0.858
Cbx2	Phc3	0.858
Pparg	Lyn	0.858
Pparg	Foxo3	0.858
Ncoa6	Bcor	0.858
Kdm4c	Arid1b	0.858
Ski	Gli3	0.858
Dbnl	Plec	0.858
Dynll1	Eef2	0.858
Dynll1	Atp5a1	0.858
Gria1	Lrp1	0.858
Suz12	Jarid2	0.858

Nup62	Nxf1	0.858
Ifrd1	Ppp2ca	0.858
Ppp1cc	Cdk4	0.858
Ubqln2	Fbln2	0.858
Sf3b1	Phc2	0.858
Map2k4	Map3k1	0.857
Mecp2	Rela	0.857
Tnfaip3	Ikbkg	0.857
Sorbs1	Fyn	0.857
Rin1	Ywhab	0.857
Rad51	Atr	0.857
Rad51	Iqgap1	0.857
Bcl11b	Gnao1	0.857
Bcl11b	Atxn2	0.857
Actbl2	Mapkapk2	0.857
Cry2	Fbxl3	0.857
Id2	Kat2b	0.857
Cd2ap	Rps6ka3	0.857
Usp2	Tnk2	0.857
Sod1	Ngfr	0.857
Lmnb1	Syncrip	0.857
Fadd	Lrdd	0.857
Sirt1	Nr0b2	0.857
Pura	Nr1h4	0.857
Eif4enif1	Smad4	0.857
Hnf1a	Sp2	0.857
Slc11a1	Bmi1	0.857
Ccnd2	Cdk4	0.857
Mtf1	Rorb	0.857
Egr2	Ddx41	0.857
Ncam1	Mbp	0.857
Siah2	Spry4	0.857
Cenpv	Eno3	0.857
Vim	Nphp1	0.857
Hbb-b1	Slc25a3	0.857
Aldoa	Foxp1	0.857
Aldoa	Psmc5	0.857
Akr1b3	Hnrnpa1	0.857
Stat5a	Stat5b	0.857
Zfp473	Nkd2	0.857
Pparg	Tab3	0.857
Pparg	Mpl	0.857
Ubb	Rps3	0.857
Thoc4	Map2k7	0.857
Dynll1	Ruvbl2	0.857
Syn2	Gsn	0.857

Hba-a1	Fbln2	0.857
Adcy8	Ppp2ca	0.857
Hist3h2a	Spata24	0.857
Casp8	Vdac1	0.857
Nrip1	Cebpa	0.857
Nphp1	Tuba1b	0.857
Syvn1	Herpud1	0.856
Mef2a	Zfp128	0.856
Psma4	Myo1c	0.856
Shc1	Sorbs1	0.856
Shc1	Sh3kbp1	0.856
Sorbs1	Ppp1ca	0.856
Gabbr1	Kif1b	0.856
Epc1	Cbx2	0.856
Usf1	Hif1a	0.856
Rnh1	Krt16	0.856
Rnh1	Try10	0.856
Sgca	Pgm5	0.856
Actbl2	Mllt1	0.856
Irf8	Reg1	0.856
Psmb1	Cct6a	0.856
Psmb1	Actn4	0.856
Irak3	Casp6	0.856
Usp2	Flt1	0.856
Sod1	Slc11a1	0.856
Sod1	Cflar	0.856
Lmnb1	Gm5414	0.856
Lmo2	Lmx1b	0.856
Lmo2	Lhx1	0.856
Gtf2h1	Snai1	0.856
Cdk5	Hist1h1a	0.856
Nkx2-5	Tbx5	0.856
Rab7	Myh9	0.856
Rab3d	Pkm2	0.856
Hist2h4	Pcna	0.856
Ahi1	Actb	0.856
Rai14	Zfp277	0.856
Rai14	Lhx2	0.856
Tcf12	Tle6	0.856
Slc11a1	Nbr1	0.856
Mapt	Camk1	0.856
Zfp236	Mycn	0.856
Egr1	Creb1	0.856
Egr2	Hhex	0.856
Eya3	Tle6	0.856
Cyld	Ntrk2	0.856

Aldoa	Ubqln2	0.856
Aldoa	Telo2	0.856
Aldoa	Tuba1b	0.856
Pparg	Prkdc	0.856
Ubb	Naca	0.856
Ubb	Mapk3	0.856
Dok3	Inpp5d	0.856
Isl2	Lhx4	0.856
Fancd2	Arntl	0.856
Fancd2	Rhoa	0.856
Fancd2	Gli2	0.856
Grin1	Ctnnb1	0.856
Aes	Msx1	0.856
Rassf1	Daxx	0.856
Blnk	Mdk	0.856
Rps13	Cfl1	0.856
Lef1	Pin1	0.856
Dlx1	Dlx2	0.856
Jund	Fos	0.856
Bag1	Cdk2	0.855
Bag1	Sox8	0.855
Mecp2	Asb6	0.855
Tnfaip3	Med16	0.855
Gtf2a1l	Hnf1a	0.855
Rad51	Hoxc12	0.855
Cited4	Zfp236	0.855
Vps16	Stx8	0.855
Rnh1	Anapc2	0.855
Rnh1	Vim	0.855
Irf8	Ctcf	0.855
Psmb5	Ntrk1	0.855
Usp2	Ski	0.855
Lmnb1	Baz1b	0.855
Traf6	Fbxo32	0.855
Nr1i2	Prdx5	0.855
Slc11a1	Cbx2	0.855
Utrn	Snta1	0.855
Egr2	Hsp90aa1	0.855
Nbr1	Sgta	0.855
Nbr1	Atg16l1	0.855
Prkcd	Atg16l1	0.855
Med1	Smad3	0.855
Pparg	Bcr	0.855
Ncoa6	Ctbp2	0.855
Phc3	Sf3b1	0.855
Ubb	Trim30	0.855

Ubb	Ldhb	0.855
Taf1a	Taf1b	0.855
Fancd2	Casp3	0.855
Brca1	Jun	0.855
Zfp277	Taf1b	0.855
Wwp1	Ticam1	0.855
Ltbr	Casp9	0.855
Foxh1	Tbx5	0.855
Jun	Fos	0.855
Sf1	Egr1	0.854
Mef2a	Mecom	0.854
Mecp2	Abtb1	0.854
Mecp2	Zfp236	0.854
Mecp2	Zfp111	0.854
Psma4	Ndufa4	0.854
Psma4	Dnaja3	0.854
Psma4	Cct6a	0.854
Sorbs1	Dlg1	0.854
Epha2	Krt6a	0.854
Rarg	Raf1	0.854
Rarg	Sfpi1	0.854
Rad51	Dnajb6	0.854
Rnh1	Myh9	0.854
Cry1	Timeless	0.854
Id3	Flnc	0.854
Id3	Gps1	0.854
Id3	Sfpi1	0.854
Id1	Ncoa1	0.854
Ss18	Ezh2	0.854
Eif3b	Rbm3	0.854
Eif3b	Fbln2	0.854
Sod1	Igf1r	0.854
Sod1	Irf5	0.854
Lmnb1	Lbxcor1	0.854
Lmo2	Lmx1a	0.854
Ssbp3	Lass2	0.854
Wbp11	Gli2	0.854
Rab5b	Tubb4	0.854
Rab3d	Dlat	0.854
Rab3d	Uqcrc1	0.854
Pura	Nefh	0.854
Tcf12	Hoxc4	0.854
Ywhah	Lrrk2	0.854
Psen2	Pde10a	0.854
Mtf1	Mycn	0.854
Irak4	Irak1	0.854

Egr2	Nfatc1	0.854
Nbr1	Fancd2	0.854
Nbr1	Ctcf	0.854
Cenpv	Tollip	0.854
Hbb-b1	Telo2	0.854
Tirap	Prpf31	0.854
Ncoa6	Grin1	0.854
Ubb	Tubb6	0.854
H1f0	Bclaf1	0.854
Smarce1	Ppp1cc	0.854
Map3k5	Srebf2	0.854
Gps1	Hoxd12	0.854
Hist3h2a	Bmi1	0.854
Tbx21	Dlx1	0.854
Fas	Sumo1	0.854
Cdkn1a	Mdm4	0.854
Rara	Ywhaz	0.853
Syvn1	Hdac6	0.853
Sorbs3	LOC674895	0.853
Cited4	Hdac10	0.853
Axin2	Gsk3b	0.853
Id3	Krt17	0.853
Id3	Ep300	0.853
Id3	Ankhd1	0.853
Cd2ap	Suz12	0.853
Psmb1	Cand2	0.853
Rac1	Ppp3ca	0.853
Rab7	Cbx5	0.853
Pura	Ncl	0.853
Pura	Calm1	0.853
Rai14	Phf2	0.853
Zfp354a	Cbx5	0.853
Naglu	Try10	0.853
Pias3	Brpf1	0.853
Cdx2	Dlx5	0.853
Efs	Gab1	0.853
Sqstm1	Ulk1	0.853
Pitx1	Egr1	0.853
Egr2	Mdm2	0.853
Tle4	Pax3	0.853
Nbr1	Phf20	0.853
Siah2	Spry1	0.853
Bhlhe41	Rfx4	0.853
Pparg	Jun	0.853
Trip4	Lass5	0.853
Zfp110	Actn4	0.853

Phc3	Usp7	0.853
Fancd2	Vdac1	0.853
Snapc4	Zfp111	0.853
Trim30	Gria1	0.853
Hba-a1	Cfl1	0.853
Pten	Ndfip1	0.853
Ikbkb	Reg1	0.853
Srf	H3f3a	0.853
Blnk	Arid3a	0.853
Tbrg1	Rpl28	0.853
Casp8	Tax1bp1	0.853
Bat1a	Gli2	0.853
Bat1a	Csf1r	0.853
Bag1	Brca1	0.852
Mef2a	Ankra2	0.852
Mecp2	Hnf1b	0.852
Mecp2	Hnf4a	0.852
Mecp2	Hdac9	0.852
Psma4	Krt14	0.852
Sorbs3	Cul5	0.852
Rarg	Myocd	0.852
Krt10	Lox	0.852
Gpbp1	Phf17	0.852
Itch	Ripk1	0.852
Rnh1	Anxa2	0.852
Rnh1	Krt77	0.852
Rnh1	Hsp90aa1	0.852
Id3	Otx2	0.852
Irf8	Foxo3	0.852
Cd2ap	Pxn	0.852
Zfand2a	Psmd14	0.852
Usp2	Gbp2	0.852
Usp2	Poli	0.852
Usp2	Flt3	0.852
Sod1	Ripk1	0.852
Lmnb1	Ncl	0.852
Lmnb1	Ddx5	0.852
Park2	Sh3gl2	0.852
Sirt1	Hnf4a	0.852
Sirt1	Nr1h4	0.852
Traf6	Mdm2	0.852
Hist2h4	Eif4a2	0.852
Hist2h4	Med16	0.852
Pura	Mark3	0.852
Pura	Ddx5	0.852
Senp6	Sumo3	0.852

Tle1	Myod1	0.852
Hnf1a	Polr1a	0.852
Naglu	Spata24	0.852
Naglu	Eprs	0.852
Nphp3	Krt79	0.852
Egr1	Mcl1	0.852
Egr2	Calr	0.852
Egr2	Ccar1	0.852
Egr2	Ikbkg	0.852
Pias1	Setdb1	0.852
Gsc	Ifrd1	0.852
Magi2	Foxh1	0.852
Nbr1	Nos2	0.852
Ncam1	Syn1	0.852
Akr1b3	Pdia6	0.852
Camk2a	Dlg1	0.852
Krt73	Nphp1	0.852
Ppard	Pparg	0.852
Pparg	Trp53	0.852
Pparg	Atxn3	0.852
Fancd2	Map3k2	0.852
Fancd2	Flt3	0.852
Suz12	Syn1	0.852
Pcna	Cdk4	0.852
Arhgef7	Lrrk2	0.852
Ubqln2	Rpl7	0.852
Gab1	Fasn	0.852
Bag1	Cyld	0.851
Psma4	Tubb6	0.851
Shc1	Erbb2	0.851
Sorbs1	Cav1	0.851
Rsad2	Irak1	0.851
Rad51	Gtf2b	0.851
Rad51	Hoxa2	0.851
Rnh1	Atp5a1	0.851
Bcl11b	Dnajb6	0.851
Sgca	Ldb3	0.851
Cd2ap	Map2k1	0.851
Psmb4	Cflar	0.851
Psmb1	Naglu	0.851
Psmb1	Eprs	0.851
Usp2	Mycn	0.851
Sod1	Junb	0.851
Slc2a4	Mapk8	0.851
Traf6	Hspa1b	0.851
Rab7	Strap	0.851

Rab3d	Ckm	0.851
Naglu	Prdx1	0.851
Mtf2	Trip4	0.851
Birc3	Ticam1	0.851
Eya3	Pknox2	0.851
Nbr1	Zfp110	0.851
Nbr1	Mycn	0.851
Nbr1	Gli3	0.851
Ncam1	Arhgef7	0.851
Tlx1	Pbx1	0.851
Rb1	Cdk2	0.851
Aldoa	Ldb3	0.851
Akr1b3	Purb	0.851
Tirap	Jak1	0.851
Atg12	Rab33b	0.851
Sf3b2	Smad2	0.851
Notch2	Notch4	0.851
Ppard	Thrb	0.851
Kdm4c	Sirt2	0.851
Ubb	Myl12b	0.851
Ubb	Hsp90b1	0.851
Ewsr1	Nkd2	0.851
Scmh1	Gmnn	0.851
Blnk	Pea15a	0.851
Hist3h2a	Tsc22d3	0.851
Ubqln2	Prdm16	0.851
Sf3b1	Bmi1	0.851
Mapkbp1	Slc2a4	0.85
Psma4	Prkacb	0.85
Sorbs1	Dok1	0.85
Epc1	Ascl1	0.85
Krt10	Glud1	0.85
Rnh1	Pcp2	0.85
Rnh1	Actn1	0.85
Rnh1	Hspa2	0.85
Irf8	Cebpb	0.85
Id1	Pbx2	0.85
Psmb4	Ntrk1	0.85
Psmb1	Krt78	0.85
Kat5	Dnmt1	0.85
Usp2	Nr1h3	0.85
Usp2	Egr2	0.85
Usp2	Skil	0.85
Sod1	Smn1	0.85
Sod1	Anxa1	0.85
Lmnb1	Hnrnpk	0.85

Gtf2h1	Pbx2	0.85
Trps1	Dynll1	0.85
Traip	Irf3	0.85
Pura	Ccnd1	0.85
Pura	Kcnd2	0.85
Pura	E2f3	0.85
Ring1	Cbx7	0.85
Ascl1	Trim27	0.85
App	Bace1	0.85
Pknox1	Smad2	0.85
Naglu	Myl12b	0.85
Slc11a1	Ar	0.85
Ndel1	Pafah1b1	0.85
Nbr1	Was	0.85
Ube2l3	Eya1	0.85
Aldoa	Hsp90aa1	0.85
Akr1b3	Gm5414	0.85
Akr1b3	Alb	0.85
Sf3b2	Foxp1	0.85
H1f0	Hspa2	0.85
Map3k5	Ltbr	0.85
Grin1	Ppp1cc	0.85
Sufu	Lgals3	0.85
Hist3h2a	Cbx7	0.85
Hist3h2a	Csk	0.85
Casp8	Gria3	0.85
Casp8	Uimc1	0.85
Zfp263	Cxxc1	0.849
Bag1	Nhlrc1	0.849
Bag1	Aldoa	0.849
Bach1	Mecom	0.849
Map2k4	Tab3	0.849
Gtf2a1l	Irf9	0.849
Psma4	Krt17	0.849
Shc1	Egfr	0.849
Epha2	Try5	0.849
Krt10	Prss1	0.849
Cited4	Setx	0.849
Rnh1	Actc1	0.849
Rnh1	Actb	0.849
Cryab	Ccnd1	0.849
Id3	Hnf4g	0.849
Id1	Trim27	0.849
Cd2ap	Kat5	0.849
Cd2ap	Gata3	0.849
Cdk6	Rb1	0.849

Psmb4	Rps27a	0.849
Rac1	Ppp2ca	0.849
Plcb4	Grm1	0.849
Sod1	Krt14	0.849
Sod1	Tnk2	0.849
Sod1	Fam175b	0.849
Sod1	Reg1	0.849
Lmnb1	Rbx1	0.849
Gtf2h1	Trps1	0.849
Traf6	Ncoa3	0.849
Traf6	Smad2	0.849
Rab3d	Vdac2	0.849
Pura	Trim32	0.849
Tle1	Mycn	0.849
Psen2	Notch2	0.849
Efs	Ptk2b	0.849
Egr2	Pax6	0.849
Mixl1	Spdef	0.849
Ncor2	Gli2	0.849
Cops8	Cops5	0.849
Nbr1	Tnk2	0.849
Nbr1	Flt1	0.849
Cenpv	Psmc2	0.849
Gsk3b	Apc	0.849
Gsk3b	Ctnnb1	0.849
Hbb-b1	Ldha	0.849
Cyld	Cflar	0.849
Akr1b3	Cfl1	0.849
Ncoa6	Mdc1	0.849
Phc3	Gon4l	0.849
Ubb	Dlg1	0.849
Fancd2	Cacna1c	0.849
Fancd2	Nbn	0.849
Kat2b	Myod1	0.849
Cbl	Inpp5d	0.849
Smad4	Pex6	0.849
Mapk8	Trp73	0.849
Rps13	Pdia6	0.849
Hist3h2a	Tfdp1	0.849
Gtf2b	Gmnn	0.849
Zfp277	Lass2	0.849
Bag1	Pkm2	0.848
Map2k4	Itch	0.848
Map2k4	Tab1	0.848
Shc1	Dlg1	0.848
Sorbs1	Spna1	0.848

Sorbs1	Eps8	0.848
Sorbs1	Cblc	0.848
Epc1	Gata4	0.848
Rad51	Msl1	0.848
Rad51	Nbn	0.848
Itch	Traf2	0.848
Rnh1	Id3	0.848
Rnh1	Aldoa	0.848
Bcl11b	Map2k7	0.848
Bcl11b	Ldhb	0.848
Id2	Phf17	0.848
Id3	Ubb	0.848
Id1	Cdk1	0.848
Cd2ap	Ldlr	0.848
Cd2ap	Nr3c1	0.848
Homer2	Grm1	0.848
Psmb5	Fam175b	0.848
Smad6	Smurf1	0.848
Phb2	Lmb1	0.848
Usp2	Reg1	0.848
Gtf2h1	Tbx21	0.848
Cdk5	Cdk4	0.848
Wdr1	Leo1	0.848
Ssbp3	Lhx2	0.848
Wwp2	Gsc	0.848
Traf6	Ikbkg	0.848
Rab3d	Ptges3	0.848
Ccdc101	Mbip	0.848
Tcf12	Hhex	0.848
Nr1i2	Pias1	0.848
Nr1i2	Pawr	0.848
Slc11a1	Mapk3	0.848
Psen2	Atp1a3	0.848
Mll1	Dbp	0.848
Max	Lbr	0.848
Zfp236	Hnf4a	0.848
Sqstm1	Cyld	0.848
Zap70	Was	0.848
Egr1	Pparg	0.848
Egr2	Jak2	0.848
Eya3	Rel	0.848
Prkce	Irf5	0.848
Aldoa	Atp5a1	0.848
Akr1b3	Rpl22	0.848
Akr1b3	Rpl7	0.848
Hspa5	Nphp1	0.848

Pparg	Cacna1c	0.848
Pparg	Rxra	0.848
Ubb	Smarce1	0.848
Fancd2	Hgs	0.848
Syn2	Ppp1r9b	0.848
Grin2a	Notch3	0.848
Ubqln2	Prkcsh	0.848
Crocc	Mapk8ip2	0.848
Lef1	Foxh1	0.848
Lef1	Jun	0.848
Lef1	Sumo1	0.848
Bag1	Stxbp1	0.847
Bag1	Actr1a	0.847
Map2k6	Sash1	0.847
Ap1b1	Hes1	0.847
Mecp2	Usf1	0.847
Mecp2	Neurog2	0.847
Mecp2	Zfp292	0.847
Sorbs1	Sorbs2	0.847
Zfp467	Stat1	0.847
Epc1	Csnk2a2	0.847
Gpbp1	Smyd2	0.847
Spna1	Sorbs2	0.847
Dok2	Bcr	0.847
Bcl11b	Nr1h4	0.847
Sin3b	Lbxcor1	0.847
Id2	Ubb	0.847
Id2	Ddx5	0.847
Id3	Map2k1	0.847
Id3	Med14	0.847
Irf8	Notch1	0.847
Irf8	Csf1r	0.847
Id1	Gtf2e2	0.847
Id1	Uchl1	0.847
Cd2ap	Lyn	0.847
Psmb4	Tbk1	0.847
Usp2	Psmc3	0.847
Usp2	Ulk1	0.847
Kit	Vav1	0.847
Hist2h4	Pbx4	0.847
Hist2h4	Mysm1	0.847
Ahi1	Krt42	0.847
Barx2	Fos	0.847
Hnf1a	Pawr	0.847
Tcf12	Rfx4	0.847
Nr1i2	Sirt2	0.847

Slc11a1	Ntrk2	0.847
Bcl6	Tbrg1	0.847
Mapt	Tsnax	0.847
Egr2	Ep300	0.847
Mixl1	Sox15	0.847
Stat3	Jun	0.847
Eya3	Smyd2	0.847
Nbr1	Cacna1c	0.847
Nbr1	Atxn3	0.847
Siah2	Thra	0.847
Cenpv	Cdh13	0.847
Ube2i	Serbp1	0.847
Aldoa	Traf3ip2	0.847
Snap25	Hgs	0.847
Sf3b2	Vdr	0.847
Notch2	Notch3	0.847
Pparg	Ppargc1a	0.847
Ubb	Nid2	0.847
Ubb	Olig2	0.847
Itga5	Atxn3	0.847
Fancd2	Tax1bp1	0.847
Hmga1	Cebpb	0.847
Gtf3c1	Gtf2b	0.847
Gtf3c1	Tbx21	0.847
Cask	Sh3gl3	0.847
Fasn	Phc2	0.847
Bach1	Zfp128	0.846
Mapkbp1	Map2k4	0.846
Map2k4	Irak1	0.846
Mecp2	Trip4	0.846
Epha2	Krt17	0.846
Epha2	Pkd1	0.846
Ercc3	Smad9	0.846
Usf1	Rarb	0.846
Gpbp1	Ifnar2	0.846
Cited4	Ss18	0.846
Cited4	Neurog2	0.846
Rnh1	Dnaja3	0.846
Rnh1	Krt73	0.846
Rnh1	Nid2	0.846
Rnh1	Eea1	0.846
Id3	Sfn	0.846
Irf8	Hras1	0.846
Cd2ap	Nck1	0.846
Cd2ap	H1f0	0.846
Psmb5	Zfp110	0.846

Psmb1	Nucb1	0.846
Usp2	Bcl3	0.846
Lmo2	Lhx2	0.846
Lmo2	Lhx9	0.846
Park2	Bcl2	0.846
Rab3d	Mog	0.846
Rab3d	Rab3b	0.846
Pura	Hspa2	0.846
Hnf1a	Zfp110	0.846
Skp1a	Myog	0.846
Ywhah	Mark3	0.846
Ccnd2	Cdk2	0.846
Psen2	Notch4	0.846
Mapt	Herpud1	0.846
Nbr1	Bace1	0.846
Nbr1	Stam2	0.846
Nbr1	Myog	0.846
Nbr1	Arntl	0.846
Nbr1	Mdm4	0.846
Ncam1	Lrrk2	0.846
Hbb-b1	Atp5d	0.846
Akr1b3	Eif4b	0.846
Sf3b2	Myod1	0.846
Pparg	Trpc4	0.846
Ncoa6	Bmi1	0.846
Ubb	Psmd11	0.846
Ubb	Actn1	0.846
Fancd2	Usp8	0.846
Grin2c	Gria1	0.846
Eif4e	Eif3e	0.846
Snapc4	Med20	0.846
Snapc4	Zfp82	0.846
Rps13	Atp5d	0.846
Rps13	Gm5414	0.846
Myc	Cdkn2a	0.846
Hhex	Jun	0.846
Sgcb	Sgcg	0.846
Cops3	Cops6	0.845
Bag1	Rac1	0.845
Mecp2	Rarg	0.845
Shc3	Atxn2	0.845
Psma4	Krt10	0.845
Shc1	Fyn	0.845
Sorbs3	Rock2	0.845
Epha2	Amfr	0.845
Rarg	Tcf12	0.845

Rarg	Hist3h2a	0.845
Ednra	Yes1	0.845
Rnh1	Psmb4	0.845
Rnh1	Fabp1	0.845
Bcl11b	Stxbp1	0.845
Sgca	Flot1	0.845
Actbl2	Eif3b	0.845
Actbl2	Eif4b	0.845
Per2	Timeless	0.845
Id2	E2f2	0.845
Id2	Cebpd	0.845
Irf8	Ddx41	0.845
Raf1	Sh3bp2	0.845
Psmb5	Calr	0.845
Psmb5	Gli3	0.845
Rac1	Hspa1b	0.845
Usp2	Fancd2	0.845
Usp2	Hgs	0.845
Sod1	Rps27a	0.845
Sod1	Aldoa	0.845
Ywhab	Mark3	0.845
Pura	Krt76	0.845
Pura	Ncor1	0.845
Tle1	Aebp2	0.845
Tle1	Hist4h4	0.845
Tcf12	Phf2	0.845
Hist1h1a	Cdk1	0.845
Egfr	Jak2	0.845
Sox2	Sumo1	0.845
Mtf2	Lass5	0.845
Mtf2	Polr1a	0.845
Sqstm1	Zfp110	0.845
Egr1	E2f1	0.845
Hist1h3f	Cdk1	0.845
Cenpv	Vdac2	0.845
Aldoa	Prdm16	0.845
Akr1b3	Ube2v2	0.845
Tirap	Cul5	0.845
Ncoa6	Zbtb33	0.845
Ubb	Stub1	0.845
Dll4	Dapk1	0.845
Gtf3c1	Iqgap1	0.845
Lcp1	Atxn2	0.845
Ubqln2	Hnrnpa1	0.845
Trp53	Mdm2	0.845
Vezf1	H2afx	0.845

Bag1	Esr1	0.844
Ap1b1	Pcmt1	0.844
Mecp2	Hes1	0.844
Mecp2	Ercc8	0.844
Mecp2	Ddx20	0.844
Shcbp1	Edc4	0.844
Psma4	Nucb1	0.844
Usf1	Myocd	0.844
Cited4	Clock	0.844
Rnh1	Sik3	0.844
Rnh1	Krt17	0.844
Rnh1	Naca	0.844
Id3	Sfrs2	0.844
Id3	Uchl1	0.844
Irf8	Ube4b	0.844
Id1	Nupr1	0.844
Raf1	Kndc1	0.844
Psmb5	Myh9	0.844
Psmb1	Ipo9	0.844
Usp2	Ezh2	0.844
Usp2	Myog	0.844
Sod1	Flt1	0.844
Lmnb1	Keap1	0.844
Six1	Eya3	0.844
Rab6	Tfrc	0.844
Rab3d	Cnp	0.844
Hist2h4	Rps3	0.844
Pura	Tfdp1	0.844
Ahi1	Tuba1b	0.844
Naglu	Adam6b	0.844
Keap1	Sqstm1	0.844
Irs1	Irs2	0.844
Mtf2	Kdm5d	0.844
Zfp236	Arntl	0.844
Flnc	Nphp1	0.844
Eya3	Nf1	0.844
Ezh1	Jarid2	0.844
Cenpv	Ldha	0.844
Aldoa	Itpr1	0.844
Aldoa	Tfrc	0.844
Ep300	Trp53	0.844
Pparg	Hdac5	0.844
Pparg	Flot1	0.844
Ncoa6	Phc3	0.844
Ncoa6	Med14	0.844
Robo1	Ighmbp2	0.844

H1f0	Inpp5d	0.844
Dynll1	Hnrnpm	0.844
Numb	Cdh1	0.844
Mtor	Rptor	0.844
Casp8	Slc1a2	0.844
Casp8	Csf1r	0.844
Nrip1	Rps5	0.844
Bag1	Epm2a	0.843
Gtf2a1l	Spata24	0.843
Shc1	Kit	0.843
Shc1	Gab1	0.843
Shc1	Diap1	0.843
Sorbs1	Asb6	0.843
Rnf41	Irak1	0.843
Epha2	Sfrs2	0.843
Epc1	Mph1	0.843
Cited4	Sh3gl1	0.843
Rnh1	Flnc	0.843
Actbl2	Cand2	0.843
Actbl2	Hba-a1	0.843
Id2	E2f1	0.843
Id3	Krt8	0.843
Id3	Krt76	0.843
Psmb5	Polk	0.843
Psmb4	Zfp110	0.843
Nedd9	Myo18a	0.843
Usp2	Nr3c2	0.843
Eif3b	Rps13	0.843
Sod1	Alb	0.843
Gtf2h1	Zfp292	0.843
Iqcb1	Tubb5	0.843
Ywhab	Lrrk2	0.843
Rab5b	Map3k5	0.843
Rab4a	Srebf2	0.843
Pura	Syn1	0.843
Barx2	Jun	0.843
Tcf12	Hoxd13	0.843
Naglu	Tuba1b	0.843
Slc11a1	Phc3	0.843
Mtf1	Hoxb1	0.843
Ndel1	Nefh	0.843
Sqstm1	Ntrk1	0.843
Egr2	Tcp1	0.843
Mixl1	Med16	0.843
Ezh2	Arntl	0.843
Prkce	Gab1	0.843

Cyld	Was	0.843
Cyld	Myd88	0.843
Aldoa	Myod1	0.843
Aldoa	Tax1bp1	0.843
Akr1b3	Ube2v1	0.843
Akr1b3	Hba-a1	0.843
Akr1b3	Eno3	0.843
Tirap	Insr	0.843
Ep300	Nr1h4	0.843
Lin7c	Cask	0.843
Sf3b2	Smad4	0.843
Med1	Med14	0.843
Ubb	Nphp1	0.843
Fancd2	Slc1a2	0.843
Dbnl	Fus	0.843
Eef1a1	Pdia6	0.843
Rps13	Nr1h4	0.843
Myod1	Tsc22d3	0.843
Cdk2	Cdkn1b	0.843
Cdk2	Cebpa	0.843
Vezf1	Sap18	0.843
Mecp2	Smad4	0.842
Mecp2	Med16	0.842
Mecp2	Fos	0.842
Shc3	Diap1	0.842
Psma4	Kpnb1	0.842
Shc1	Frs2	0.842
Gabbr1	Jakmip1	0.842
Rarg	Rarb	0.842
Rad51	Vav1	0.842
Smc1b	Smc3	0.842
Phc1	Scmh1	0.842
Smad9	Klf10	0.842
Rnh1	Phb2	0.842
Rnh1	Polr2b	0.842
Id3	Cops7b	0.842
Cd2ap	Fyn	0.842
Tcerg1	Cdk2	0.842
Tcerg1	Ctnna1	0.842
Usp2	Keap1	0.842
Usp2	Krt8	0.842
Usp2	Casp8	0.842
Eif3b	Hbb-b1	0.842
Sod1	Phf20	0.842
Sod1	Tubb5	0.842
Crkl	Clybl	0.842

Pcgf2	Phc2	0.842
Traf3	Ltbr	0.842
Rab3d	Rab3c	0.842
Rab3d	Eno3	0.842
Hist2h4	Tada3	0.842
Bub1	Smc2	0.842
Pura	Pcna	0.842
Zfp354a	Sertad1	0.842
Efs	Sh3bp2	0.842
Egr1	Fbxw7	0.842
Mixl1	Hoxc12	0.842
Mixl1	Hoxa10	0.842
Tmf1	Hdac6	0.842
Nbr1	Rhoa	0.842
Nbr1	Msx2	0.842
Nbr1	Tbk1	0.842
Prkcd	Trim30	0.842
Cenpv	Sf3b1	0.842
Akr1b3	Fbln2	0.842
Akr1b3	Asap2	0.842
Akr1b3	Eif3e	0.842
Tirap	Tubb5	0.842
Snap25	Sgta	0.842
Ubb	Mapk1	0.842
Fancd2	Runx2	0.842
Dynll1	Aifm1	0.842
Eef1a1	Tubb5	0.842
Gtf3c1	Sap18	0.842
Hist3h2a	Hoxa9	0.842
Hist3h2a	Taf1b	0.842
Hist3h2a	Cebpa	0.842
Ubqln2	Eno3	0.842
Wwp1	Actn4	0.842
Mef2a	Smad4	0.841
Ap1b1	Zbtb17	0.841
Mecp2	Zdhhc6	0.841
Mecp2	Ss18	0.841
Gtf2a1l	Spdef	0.841
Sorbs1	Flot1	0.841
Epha2	Rsrc1	0.841
Epc1	Kat2b	0.841
Rarg	Gata4	0.841
Usf1	Tle6	0.841
Rnh1	Psma1	0.841
Rnh1	Actg1	0.841
Rnh1	Efhd2	0.841

Actbl2	Akr1b3	0.841
Id3	Prss1	0.841
Id3	Mysm1	0.841
Cd2ap	Pkd1	0.841
Cul3	Zbtb16	0.841
Raf1	Diap1	0.841
Psmb5	Flnc	0.841
Usp2	Pkd2	0.841
Usp2	Erbb2	0.841
Usp2	Phf20	0.841
Eif3b	Nrip1	0.841
Mzf1	Zfp768	0.841
Abl1	Rasa1	0.841
Tle1	Satb2	0.841
Tle1	Mapk3	0.841
Naglu	Thrap3	0.841
Pias3	Dnmt1	0.841
Tipin	Timeless	0.841
Grip1	Kat2b	0.841
Egr2	Uimc1	0.841
Bcl2	Ppp1cc	0.841
Gtf2e2	Gtf2e1	0.841
Tmf1	Gsc	0.841
Ncor2	Rfxank	0.841
Nbr1	Mc4r	0.841
Bhlhe41	Pbx4	0.841
Cenpv	Cnp	0.841
Mapk8ip3	Mapk8ip2	0.841
Akr1b3	Calm2	0.841
Akr1b3	Ptrf	0.841
Akr1b3	Psmc5	0.841
Sf3b2	Kat2b	0.841
Ubb	Ddx5	0.841
Ubb	Ankhd1	0.841
H1f0	Rps6ka3	0.841
Fancd2	Fyn	0.841
Fancd2	Ccar1	0.841
Fancd2	Cebpa	0.841
Dbnl	Atxn2	0.841
H1	Cdk2	0.841
Map3k5	Cdk9	0.841
Hba-a1	Pdia6	0.841
Gtf3c1	H2afx	0.841
Rps13	Rpsa	0.841
Hist3h2a	Clock	0.841
Ubqln2	Pcbp1	0.841

Tceb1	Socs2	0.841
Wwp1	Ngfr	0.841
Tbp	Dlx2	0.841
Tbp	Hoxa10	0.841
Casp8	Gli2	0.841
Topbp1	Lpxn	0.841
Fau	Bmi1	0.841
Hif1a	Egln3	0.841
Trp73	Trp63	0.841
Bag1	Atf4	0.84
Bag1	Dnaja1	0.84
Syvn1	Ncstn	0.84
Sumo2	Nfkbia	0.84
Shc3	Syngap1	0.84
Psma4	Jup	0.84
Sorbs1	Arhgef7	0.84
Als2	Ntrk2	0.84
Rarg	Socs7	0.84
Cited4	Kat2a	0.84
Cd40	Traf2	0.84
Rnh1	Plec	0.84
Rnh1	Prdx1	0.84
Bcl11b	Ldha	0.84
Id3	Gfi1	0.84
Id3	Ncoa1	0.84
Id3	Cops6	0.84
Id3	Pi4ka	0.84
Cd2ap	Park7	0.84
Rac1	Dlg4	0.84
Usp2	Fam175b	0.84
Usp2	Hist2h2bb	0.84
Pcbd1	Hnf1a	0.84
Lmnb1	Rorc	0.84
Lmo2	Zfp593	0.84
Lmo2	Csdc2	0.84
Gtf2h1	Mapk8	0.84
Pcp2	Ctnna1	0.84
Rab3d	Cenpv	0.84
Pura	Ccnd3	0.84
Tle1	Tef	0.84
Tle1	Mapk9	0.84
Zfp354a	Cbx1	0.84
Hnf1a	Pax3	0.84
Naglu	Serpinb7	0.84
Nr1i2	Hnf4g	0.84
Efs	Map1lc3b	0.84

Max	Eif2a	0.84
Mtf1	Zfp275	0.84
Prkaca	Gck	0.84
Egr2	Psmd2	0.84
Birc2	Map3k7	0.84
Tmf1	Smn1	0.84
Tmf1	Irf5	0.84
Nbr1	Mcl1	0.84
Nbr1	Cflar	0.84
Nbr1	Junb	0.84
Cenpv	Ptges3	0.84
Hbb-b1	Ptrf	0.84
Aldoa	Lgals3	0.84
Sall4	Ewsr1	0.84
Ppargc1a	Ccnh	0.84
Ncoa6	Ppara	0.84
Itpr1	Ube4b	0.84
Trim30	Map1lc3a	0.84
Hba-a1	Vdac2	0.84
Gtf3c1	Psme3	0.84
Rps13	Kcnd2	0.84
Zfp277	Olig2	0.84
Foxh1	Ing1	0.84
Bag1	Bcl11b	0.839
Bag1	Psmc2	0.839
B2m	Cflar	0.839
Map2k4	Ube2n	0.839
Psma4	Psmb4	0.839
Psma4	Rps27a	0.839
Psma4	Ipo9	0.839
Sorbs1	Pxn	0.839
Sorbs1	Bcar1	0.839
Sorbs1	Inpp5d	0.839
Sorbs1	Csf1r	0.839
Rarg	Ncoa1	0.839
Cited4	Ssbp3	0.839
Cited4	Myb	0.839
Smad9	Gjb6	0.839
Rnh1	Hnrnph2	0.839
Rnh1	Alb	0.839
Bcl11b	Eif2c2	0.839
Actbl2	Yaf2	0.839
Id1	Rel	0.839
Cd2ap	Eps8	0.839
Ssbp2	Lmo1	0.839
Raf1	Dlg2	0.839

Rac1	Runx2	0.839
Rev1	Pcna	0.839
Usp2	Mdm4	0.839
Sod1	Nfkbia	0.839
Lmnb1	Strap	0.839
Cdk5	Cdk1	0.839
Ptk2	Ppp1ca	0.839
Traf6	Sqstm1	0.839
Ahi1	Krt15	0.839
Ahi1	Hspa9	0.839
Rai14	Isl2	0.839
Rai14	Sirt2	0.839
Nr1i2	Nr1i3	0.839
Psen2	Ryr2	0.839
Efs	Csn2	0.839
Zbtb16	Dnmt1	0.839
Zfp236	Dynll1	0.839
Sla2	Dok3	0.839
Egr2	Trp53	0.839
Klc1	Mapk8ip2	0.839
Birc2	Ube2n	0.839
Ube2n	Ikbkg	0.839
Bace1	Dlg1	0.839
Ube2i	Eif3i	0.839
Gsk3b	Lrrk2	0.839
Aldoa	Cops4	0.839
Akr1b3	Ckm	0.839
Sf3b2	Foxh1	0.839
Med1	Cdk1	0.839
Pparg	Ube4b	0.839
Ppargc1a	Kat2b	0.839
Ppara	Trim24	0.839
Ubb	Tubb2c	0.839
Pxn	Mdk	0.839
Irak1	Nr0b2	0.839
Grin2a	Dlg2	0.839
Rbx1	Ptma	0.839
Smad4	Prdm16	0.839
Smad2	Prdm16	0.839
Gtf3c1	Sox15	0.839
Rps13	Nrip1	0.839
Ubqln2	Cand1	0.839
Ubqln2	Eprs	0.839
Casp8	Nos2	0.839
Bat1a	Tbk1	0.839
Bag1	Map3k1	0.838

Map2k4	Tab2	0.838
Mecp2	Kif2a	0.838
Ptprq	Cdh1	0.838
Cyfip2	Wasf2	0.838
Usf1	Ncoa1	0.838
Krt10	Actn1	0.838
Sgca	Ptrf	0.838
Actbl2	Ube2v1	0.838
Actbl2	Eif4a1	0.838
Per2	Usp2	0.838
Id2	Taf1b	0.838
Id3	Krt79	0.838
Id3	Med16	0.838
Psmb4	Krt2	0.838
Eif3b	Atp5d	0.838
Eif3b	Telo2	0.838
Eif3b	Kcnd2	0.838
Sod1	Acta1	0.838
Sod1	Hist2h2bb	0.838
Sod1	Rer1	0.838
Gtf2h1	Hnf4g	0.838
Hand2	Hoxd13	0.838
Hand1	Fhl2	0.838
Psen1	Notch2	0.838
Traf6	Vcp	0.838
Rab5b	Rab4a	0.838
Hist2h4	Eif4a1	0.838
Ccdc101	Tada3	0.838
Hnf1a	Tox3	0.838
Nr1i2	Rnf19a	0.838
Nr1i2	Tgif1	0.838
Pias3	Nr0b2	0.838
Mtf2	Smarca1	0.838
Zfp236	Hoxd3	0.838
Kat2a	Csrp2bp	0.838
Egr2	Olig2	0.838
Birc2	Tmem115	0.838
Birc3	Sh3gl2	0.838
Mixl1	Stat2	0.838
Mixl1	Hoxa2	0.838
Tmf1	Pten	0.838
Foxn2	Sirt2	0.838
Tbpl1	Gmnn	0.838
Ube2e3	Ptma	0.838
Akr1b3	Eif4a1	0.838
Tirap	Irak1	0.838

Tirap	Tceb2	0.838
Pparg	Spry4	0.838
Ppara	Rxra	0.838
Ubb	Taf1b	0.838
Fancd2	Tardbp	0.838
Fancd2	Dlg1	0.838
Fancd2	Nos2	0.838
Dynll1	Hnrnpf	0.838
Grb2	Csf1r	0.838
Eef1a1	Nf1	0.838
Chmp5	Epas1	0.838
Ubqln2	Alb	0.838
Bag1	Cul2	0.837
Bag1	Ppp1cc	0.837
Mecp2	Gsc	0.837
Psma4	Pcmtd1	0.837
Shc1	Nedd4	0.837
Trim66	Lbr	0.837
Rnh1	Sfrs2	0.837
Bcl11b	Hist3h2a	0.837
Id3	Sfrs1	0.837
Id3	Glud1	0.837
Cul3	Keap1	0.837
Psmb5	Krt14	0.837
Psmb5	Stam2	0.837
Psmb5	Actn4	0.837
Psmb5	Tbk1	0.837
Usp2	Map3k2	0.837
Usp2	Ngfr	0.837
Eif3b	Pdia6	0.837
Lmnb1	Ube2i	0.837
Traip	Irf1	0.837
Rab5b	Rab3a	0.837
Rab3d	Vdac1	0.837
Hist2h4	Ivns1abp	0.837
Pura	Kat2b	0.837
Gabrg2	Ppp3ca	0.837
Otx2	Alx4	0.837
Nr1i2	Sap18	0.837
Grm1	Itpr1	0.837
Mapt	Tab2	0.837
Mtf2	Rps13	0.837
Dnaja3	Hspa9	0.837
Zfp236	Pax3	0.837
Egr2	Sgta	0.837
Egr2	Slc1a2	0.837

Birc2	Diap1	0.837
Mixl1	Tbx21	0.837
Nbr1	Vim	0.837
Nbr1	Nfkbia	0.837
Nbr1	Gria1	0.837
Prkcd	Gria1	0.837
Prkcd	Actn2	0.837
Trib2	Reg1	0.837
Atm	Dnmt1	0.837
Pparg	Slc1a2	0.837
Kdm4c	Zfp592	0.837
Ubb	Brca1	0.837
Tef	Gtf2h2	0.837
Fancd2	Tab3	0.837
Fancd2	Hras1	0.837
Map3k1	Map2k7	0.837
Hba-a1	Rpl22	0.837
Cblb	Ticam1	0.837
Gtf3c1	Vav1	0.837
Rps13	Fus	0.837
Rabl4	Ift20	0.837
Hist3h2a	Ppp2ca	0.837
Ifrd1	Mef2d	0.837
Ubqln2	Asap2	0.837
Usp7	Mdm4	0.837
Bag1	Hoxc8	0.836
Bag1	Pfn1	0.836
Bag1	Ptrf	0.836
Bag1	Slc25a3	0.836
Mef2a	Gli2	0.836
Map2k6	Tab3	0.836
Mecp2	Foxo3	0.836
Shc1	Nck1	0.836
Sorbs1	Tlr4	0.836
Sorbs1	Cblb	0.836
Epha2	Sik3	0.836
Gpbp1	Creb1	0.836
Cited4	Ssbp4	0.836
Bcl11b	Iqgap1	0.836
Tcf3	Id3	0.836
Cry2	Per2	0.836
Id3	Rpl4	0.836
Id3	Krt77	0.836
Id3	Bhlhe41	0.836
Irf8	Atxn3	0.836
Psmb5	Trim28	0.836

Psmb1	Ube2a	0.836
Usp2	Smurf1	0.836
Usp2	Ube4b	0.836
Sod1	Prss1	0.836
Sod1	Egr2	0.836
Gtf2h1	Csdc2	0.836
Cdk5	E2f1	0.836
Six1	Eya1	0.836
Crkl	Bcar1	0.836
Mzf1	Zscan2	0.836
Jdp2	Pbx4	0.836
5-Sep	2-Sep	0.836
Naglu	Tubb5	0.836
Naglu	Hoxb9	0.836
Btbd10	Ttc3	0.836
Efs	Atxn2	0.836
Esr1	Ar	0.836
Tlr4	Cblb	0.836
Sox2	Irf1	0.836
Mapt	Gsc	0.836
Mtf2	Suz12	0.836
Zfp236	Zfp275	0.836
Mixl1	Nr4a1	0.836
Nbr1	Hgs	0.836
Hbb-b1	Irf3	0.836
Hbb-b1	Ticam1	0.836
Ep300	Kat2b	0.836
Trip4	Foxp1	0.836
Zfp110	Sh3gl2	0.836
Zfpm1	Ifrd1	0.836
Fancd2	Ppp2cb	0.836
Syn2	Vasp	0.836
Git1	Arhgef6	0.836
Eef1a1	Tubb6	0.836
Smad2	Sh3gl2	0.836
Hba-a1	Psmc5	0.836
Suz12	Sap18	0.836
Ubqln2	Rpl22	0.836
Zfp277	Tsc22d3	0.836
Zfp277	Csde1	0.836
Mapk7	Ptges3	0.836
Vps11	Stx6	0.836
Syvn1	Efhd2	0.835
Map2k4	Ecsit	0.835
Mecp2	Etv6	0.835
Sorbs1	Egfr	0.835

Sorbs1	Pdlim7	0.835
Creb3l4	Anapc7	0.835
Epha2	Pkp1	0.835
Epha2	Zdhhc13	0.835
Rad51	Rps6ka3	0.835
Rnh1	Tpi1	0.835
Rnh1	Hspa9	0.835
Bcl11b	Gtf2b	0.835
Myl9	Epas1	0.835
Actbl2	Ckm	0.835
Id3	Zdhhc13	0.835
Psmb5	Krt77	0.835
Psmb5	Hsp90b1	0.835
Psmb1	Ndufa4	0.835
Usp2	Rela	0.835
Sod1	Mycn	0.835
Cdk5	Ccnb1	0.835
Park2	Bax	0.835
Traf2	Ube2d3	0.835
Rab5b	Ywhaq	0.835
Hist2h4	Trip4	0.835
Pura	Rps6	0.835
Pura	Kif5b	0.835
Rai14	Trip4	0.835
Ldb2	Lmo3	0.835
Zfp354a	Isl2	0.835
Naglu	Krt42	0.835
Keap1	Ptma	0.835
Efs	Bcar1	0.835
Efs	Diap1	0.835
Inadl	Grin2a	0.835
Sox2	Uimc1	0.835
Mtf1	Arntl	0.835
Mtf2	C1qbp	0.835
Zfp236	Hoxb1	0.835
Cacng2	Gria2	0.835
Tmf1	Rhoa	0.835
Nbr1	Gli2	0.835
Aldoa	Slc1a2	0.835
Akr1b3	Ldha	0.835
Ppard	Trim24	0.835
Ubb	Pcna	0.835
Thoc4	Vcp	0.835
Trim30	Mapk3	0.835
Rps13	Prkcsh	0.835
Ifrd1	Tal1	0.835

Ccna1	Cdk2	0.835
Irf3	Tbk1	0.835
Nrip1	Ap2m1	0.835
Nrip1	Rps3	0.835
Syvn1	Eea1	0.834
Map2k6	Tab2	0.834
Psma4	Aifm1	0.834
Shc1	Grin1	0.834
Col5a1	Smad4	0.834
Usf1	Sfpi1	0.834
Gpbp1	Sertad2	0.834
Rad51	Gata3	0.834
Cited4	Ewsr1	0.834
Ncstn	Herpud1	0.834
Rnh1	Psma7	0.834
Rnh1	Atp5d	0.834
Bcl11b	Atf2	0.834
Actbl2	Pik3ca	0.834
Id2	Pcna	0.834
Id2	Ccna1	0.834
Id3	Tubb2a	0.834
Id3	Hnrnpf	0.834
Psmb4	Myog	0.834
Rnf2	Runx1	0.834
Tcerg1	Mdm2	0.834
LOC677235	Dclk2	0.834
Usp2	Pklr	0.834
Eif3b	Anxa1	0.834
Eif3b	Eif2a	0.834
Sod1	Skil	0.834
Lmo2	Pitx2	0.834
Crk	Irs1	0.834
Lbxcor1	Hdac1	0.834
Traf2	Map3k1	0.834
Ahi1	Hsp90ab1	0.834
Tle1	Clock	0.834
Nr1i2	Rorb	0.834
Slc11a1	Mph1	0.834
Tlr4	Bcl10	0.834
Tlr4	Ube2n	0.834
Max	Ccna1	0.834
Max	Trp53	0.834
Myh3	Neurl2	0.834
Birc2	Map3k1	0.834
Tmf1	Stam2	0.834
Eya3	Pbx1	0.834

Dab1	Was	0.834
Nbr1	Smurf1	0.834
Nbr1	Psmc3	0.834
Nbr1	Uimc1	0.834
Prkcb	Rps6ka3	0.834
Prkcd	Ntrk2	0.834
Bhlhe41	Pdcd6	0.834
Cenpv	Slc25a3	0.834
Cenpv	Uqcrc1	0.834
Ppp2r1a	Ppp2ca	0.834
Cyld	Ntrk1	0.834
Akr1b3	Dsp	0.834
Akr1b3	Atp5d	0.834
Pparg	Cdkn2a	0.834
Trip4	Mafk	0.834
Phc3	Bmi1	0.834
Mafk	Nfe2	0.834
Dbnl	Gab2	0.834
Dynll1	Pafah1b1	0.834
Hba-a1	Palm	0.834
Hba-a1	Efhd2	0.834
Gria1	Gria2	0.834
Mapk8	Daxx	0.834
Vezf1	Atxn2	0.834
Socs2	Lepr	0.834
Bag1	Hsp90ab1	0.833
Bag1	Syn1	0.833
Map2k6	Bcl10	0.833
Mecp2	Kat2a	0.833
Mecp2	Stag2	0.833
Mecp2	Ncor2	0.833
Arhgap32	Rac1	0.833
Psma4	Myh9	0.833
Shc1	Dusp2	0.833
Shc1	Ptpn11	0.833
Shc1	Map1lc3b	0.833
Epha2	Glud1	0.833
Epc1	Myocd	0.833
Flna	Rps27a	0.833
Usf1	Cebpa	0.833
Gpbp1	Rxrg	0.833
Rph3al	Rims1	0.833
Rad51	Gtf3c1	0.833
Cited4	Mtf1	0.833
Cited4	Hoxa2	0.833
Ednra	Arrb1	0.833

Itch	Smad2	0.833
Bcl11b	Dnajb1	0.833
Myl9	Tada3	0.833
Actbl2	Cenpv	0.833
Cry1	Per3	0.833
Id2	Psme3	0.833
Id3	Trip4	0.833
Irf8	Runx2	0.833
Id1	Bhlhe41	0.833
Psmb5	Reg1	0.833
Usp2	Itga5	0.833
Eif3b	Eif3e	0.833
Sod1	Nr1h3	0.833
Sod1	Tuba1b	0.833
Pkd2	Herpud1	0.833
Traf2	Ikbkg	0.833
Hist2h4	Rpl4	0.833
Hist2h4	Lass2	0.833
Pura	Ctnnd1	0.833
Zfp354a	Foxq1	0.833
Tcf12	Neurog2	0.833
Cbx3	Trim28	0.833
Naglu	Hsp90b1	0.833
Naglu	Nefm	0.833
Pdia3	Slc25a3	0.833
Cops2	Gsk3b	0.833
Efs	Khdrbs1	0.833
Bcl6	Ppp1r9b	0.833
Max	Atm	0.833
Mtf2	Nkd2	0.833
Ncoa3	Rnf19a	0.833
Foxn2	Zfp277	0.833
Nbr1	Vdac1	0.833
Ncam1	Apoe	0.833
Bhlhe41	Prpf31	0.833
Cenpv	Yaf2	0.833
Ube2i	Phf17	0.833
Ube2i	Sumo1	0.833
Zfp768	Zfp473	0.833
Hbb-b1	Ubqln2	0.833
Aldoa	Tuba4a	0.833
Ep300	Cebpa	0.833
Trip4	Ubb	0.833
H1f0	Sap18	0.833
H1f0	H2afx	0.833
Dynll1	Rcn2	0.833

Cops7b	Gps1	0.833
Map3k7	Ikbkg	0.833
Wnt1	Fzd8	0.833
Hba-a1	Telo2	0.833
Rps13	Fbln2	0.833
Ppp1cc	Cdk2	0.833
Zfp277	Gtf2f1	0.833
Ppp2ca	Ppp2r3a	0.833
Gab1	Cdh1	0.833
Casp8	Ddx41	0.833
Topbp1	Fbxo28	0.833
Bag1	Stat3	0.832
B2m	Tbk1	0.832
Bach1	Zmym2	0.832
Mecp2	Pds5a	0.832
Psma4	Mms19	0.832
Psma4	Rcn2	0.832
Sorbs1	Kit	0.832
Sorbs1	Efs	0.832
Sorbs1	Bcl2	0.832
Pik3r1	Csf1r	0.832
Krt10	Mtap2	0.832
Rad51	Atm	0.832
Rad51	H1f0	0.832
Cited4	Ep300	0.832
Cited4	Hnf4a	0.832
Rnh1	Eprs	0.832
Bcl11b	Vav1	0.832
Per1	Usp2	0.832
Id2	Neurog2	0.832
Id2	Rfx4	0.832
Irf8	Nos2	0.832
Psmb5	Fancd2	0.832
Psmb5	Mdm4	0.832
Psmb4	Tubb5	0.832
Psmb1	Flnc	0.832
Psmb1	Exoc4	0.832
Usp2	Adrbk1	0.832
Eif3b	Rps6	0.832
Eif3b	Rpl22	0.832
Sod1	Stam2	0.832
Sod1	Psmd2	0.832
Traf3	Map3k1	0.832
Rab7	Ywhag	0.832
Zfp369	Zfp110	0.832
Pura	Ywhah	0.832

Tcf12	Pbx4	0.832
Naglu	Calm2	0.832
Naglu	Hspa9	0.832
Orc2l	Zfp592	0.832
Nr1i2	Lgals3	0.832
Slc11a1	Sqstm1	0.832
Pias3	Gmnn	0.832
Tlr4	Birc3	0.832
Mapt	Tmf1	0.832
Mtf2	Asb6	0.832
Pou2f1	Tle4	0.832
Birc3	Casp3	0.832
Mixl1	Hoxd13	0.832
Mixl1	Nkd2	0.832
Prkcd	Ulk1	0.832
Prkce	Kdr	0.832
Hbb-b1	Eno3	0.832
Cbx5	Hist4h4	0.832
Zfp473	Zfp275	0.832
Pparg	Smad2	0.832
Pparg	Spry1	0.832
Pparg	Hdac3	0.832
Pparg	Ctnnb1	0.832
Ubb	Thrap3	0.832
Fancd2	Hdac5	0.832
Fancd2	Foxo3	0.832
Fancd2	Ddx41	0.832
Syn2	Hdac1	0.832
Syn2	Nos1	0.832
Grin2a	Dll1	0.832
Cebpd	Lass2	0.832
Trim30	Ngfr	0.832
Cblb	Myd88	0.832
Blnk	Elk1	0.832
Hist3h2a	E2f6	0.832
Gtf2b	Tbp	0.832
Arrb2	Usp7	0.832
Tceb2	Wsb1	0.832
Hoxa9	Phf17	0.832
Fzr1	Aurka	0.832
Wwp1	Tax1bp1	0.832
Casp8	Fos	0.832
Nxt1	Nxf1	0.832
Map2k4	Birc2	0.831
Mecp2	Stat5a	0.831
Mecp2	Nfya	0.831

Gtf2a1l	Meis2	0.831
Shc1	Pag1	0.831
Sorbs1	Erbb2	0.831
Rnf41	Myd88	0.831
Epha2	Naglu	0.831
Epha2	Dsp	0.831
Epc1	Sfpi1	0.831
Flna	Tuba1b	0.831
Rad51	Nde1	0.831
Smad9	Mxi1	0.831
Esrrb	Tbp	0.831
Rnh1	Dnaja2	0.831
Bcl11b	Foxp1	0.831
Id2	Polr1a	0.831
Id3	Naglu	0.831
Id3	Cdk1	0.831
Id1	Smad2	0.831
Psmb4	Actn4	0.831
Phb2	Myod1	0.831
Sod1	Pkd2	0.831
Sod1	Nbr1	0.831
Sod1	Mc4r	0.831
Sod1	Krt42	0.831
Lmnb1	Nup153	0.831
Mzf1	Zfp473	0.831
Rab7	Tnpo1	0.831
Hist2h4	Smarce1	0.831
Hist2h4	Satb1	0.831
Rai14	Rnf14	0.831
Nr1i2	Thrb	0.831
Ccnd1	Cdk4	0.831
Mapt	Stat1	0.831
Egr2	Map3k2	0.831
Egr2	Vamp2	0.831
Per3	Clock	0.831
Gsc	Pparg	0.831
Nbr1	Foxo3	0.831
Nbr1	Csf1r	0.831
Prkce	Ppp3ca	0.831
Cenpv	Ndufa9	0.831
Ube2i	Pdlim4	0.831
Rb1	Psmd10	0.831
Dnajc1	Hsp90b1	0.831
Aldoa	Map3k1	0.831
Aldoa	Nphp1	0.831
Aldoa	Alb	0.831

Akr1b3	Cand1	0.831
Foxa1	Dlx1	0.831
Notch2	Sufu	0.831
Pparg	Vamp2	0.831
Rhoq	Cblb	0.831
Uba52	Map2k7	0.831
Ubb	Map1lc3a	0.831
Dbnl	Vav1	0.831
Dynll1	Krt78	0.831
Dynll1	Nphp1	0.831
Suz12	Rpl28	0.831
Rps13	Telo2	0.831
Pcna	Ppp1cc	0.831
Rpl7	Eif3e	0.831
Casp8	Fau	0.831
Bat1a	Ngfr	0.831
Bag1	Mapk8	0.83
B2m	Zfp110	0.83
Mapkbp1	Atf2	0.83
Tnfaip3	Traf2	0.83
Trim26	Phf20	0.83
Axin2	Apc	0.83
Rnh1	Dnaja1	0.83
Rnh1	Naglu	0.83
Rnh1	Amfr	0.83
Rnh1	Krt76	0.83
Bcl11b	Nr3c1	0.83
Per1	Per2	0.83
Per1	Timeless	0.83
Id3	Yeats2	0.83
Id3	Prpf31	0.83
Cd2ap	Ssbp3	0.83
Psmb5	Actg1	0.83
Psmb5	Phf20	0.83
Psmb5	Was	0.83
Psmb4	Pkd2	0.83
Psmb1	Tubb6	0.83
Psmb1	Myo1c	0.83
Usp2	Becn1	0.83
Eif3b	Rps16	0.83
Eif3b	G3bp1	0.83
Eif3b	Cand2	0.83
Eif3b	Hba-a1	0.83
Eif3b	Ube2v2	0.83
Sod1	Erbb2	0.83
Sod1	Msx2	0.83

Lmnb1	Tnpo1	0.83
Lmnb1	Ddx3y	0.83
Six1	Neurod1	0.83
Hist2h4	Myod1	0.83
Nr1i2	Setdb1	0.83
Egr2	Ilk	0.83
Birc2	Tnip3	0.83
Tmf1	Fyn	0.83
Tmf1	Was	0.83
Nbr1	Camk1	0.83
Nbr1	Hist2h2bb	0.83
Ncam1	Mog	0.83
Bhlhe41	Psme3	0.83
Cenpv	Ckm	0.83
Cenpv	Cbx7	0.83
Ppp2r1a	Ppp2r3a	0.83
Ube2i	Pax6	0.83
Cyld	Rela	0.83
Aldoa	Calm2	0.83
Akr1b3	Tubb5	0.83
Akr1b3	Hnrnpk	0.83
Tirap	Bclaf1	0.83
Ep300	Foxo3	0.83
Atg16l1	Ulk1	0.83
Pparg	Ikbkg	0.83
Pparg	Tardbp	0.83
Pparg	Rxrg	0.83
Kdm4c	Actn2	0.83
Ubb	Myo1c	0.83
Thoc4	Kat2b	0.83
Arntl	Clock	0.83
Polr1b	Zfp592	0.83
Sufu	Hist1h2ai	0.83
Stub1	Atxn3	0.83
Blnk	Cttn	0.83
Suz12	Vdac2	0.83
Rps13	Ubqln2	0.83
Hist3h2a	Rpsa	0.83
Hist3h2a	Sf3b1	0.83
Mlst8	Mtor	0.83
Ppp5c	Dmrtc2	0.83
Gab1	Cav3	0.83
Topbp1	Elf1	0.83
Zfp263	Mzf1	0.829
Spna2	Ncam1	0.829
Gtf2a1l	Id3	0.829

Gtf2a1l	Meis1	0.829
Shc1	Hck	0.829
Shc1	Ptk2b	0.829
Sorbs1	Abl1	0.829
Creb3l4	Smc2	0.829
Epc1	Pcgf1	0.829
Cited4	Tle1	0.829
Cited4	Csk	0.829
Bcl11b	Usp9x	0.829
Bcl11b	Kat2b	0.829
Id2	Ccnb1	0.829
Id2	Eif2a	0.829
Id3	2210010C04	0.829
Id3	Ndufa4	0.829
Id3	Ascl1	0.829
Cd2ap	Bax	0.829
Psmb5	Tsnax	0.829
Psmb1	Hsph1	0.829
Psmb1	Aifm1	0.829
Tcerg1	Kcnd2	0.829
Usp2	Sgta	0.829
Eif3b	Dsp	0.829
Sod1	Hspa9	0.829
Sod1	Ldha	0.829
Lmnb1	Ywhag	0.829
Lmnb1	Hnrnph2	0.829
Polk	Mad2l2	0.829
Traf2	Ripk1	0.829
Sos1	Spry4	0.829
Hnf1a	Snapc4	0.829
Atf3	Hdac3	0.829
Mapt	Dlg3	0.829
Egr1	Bhlhe41	0.829
Lbr	Cbx5	0.829
Bcl2l1	Ppp1cc	0.829
Stat3	Csf1r	0.829
Gsc	Gli3	0.829
Ezh2	Setx	0.829
Nbr1	Cyld	0.829
Nbr1	Skil	0.829
Nbr1	Notch1	0.829
Bhlhe41	Trim27	0.829
Hbb-b1	Uqcrc1	0.829
Sf3b2	Hoxa13	0.829
Map3k7	Myd88	0.829
Eif4a1	Cfl1	0.829

Eif4a1	Fbln2	0.829
Eef1a1	Tuba1a	0.829
Smad2	Sykb	0.829
Smad2	Ctnnb1	0.829
Smad3	Rela	0.829
Snapc4	Polr1a	0.829
Hba-a1	Ube2v2	0.829
Hba-a1	Ptrf	0.829
Hba-a1	Eea1	0.829
Hba-a1	Eno3	0.829
Hba-a1	Asap2	0.829
Rnf170	Erlin2	0.829
Gtf3c1	Dlx5	0.829
Suz12	Slc25a3	0.829
Rps13	Psmc3	0.829
Zfp277	Pdlim4	0.829
Tceb2	Asb2	0.829
Ppp5c	Cttn	0.829
Fasn	Strap	0.829
Sox8	Jun	0.829
Bach1	Mxi1	0.828
Mecp2	Rarb	0.828
Mecp2	Hdac10	0.828
Ttc7b	Pi4ka	0.828
Gtf2a1l	Wwp1	0.828
Shc1	Sos1	0.828
Shc1	Bcr	0.828
Sorbs3	Braf	0.828
Epc1	Med14	0.828
Usf1	Gata4	0.828
Dok2	Inpp5d	0.828
Cul1	Skp1a	0.828
Rnh1	Try5	0.828
Rnh1	Sod1	0.828
Rnh1	Ahi1	0.828
Rnh1	Cct3	0.828
Rnh1	Anxa1	0.828
Rnh1	Hspb1	0.828
Bcl11b	Plp1	0.828
Actbl2	Scmh1	0.828
Cd2ap	Zap70	0.828
Ngly1	Amfr	0.828
Psmb5	Cflar	0.828
Psmb5	Sykb	0.828
Psmb5	Cdh1	0.828
Rac1	Dlg1	0.828

Ankrd1	Ttn	0.828
Tcerg1	Klf4	0.828
Usp2	Apc	0.828
Eif3b	Purb	0.828
Eif3b	Tceb1	0.828
Eif3b	Eno3	0.828
Sod1	Atp5b	0.828
Lmo2	Isl2	0.828
Iqcb1	Myh9	0.828
Krt14	Ahi1	0.828
Rab7	Ik	0.828
Rab3d	Suz12	0.828
Pura	Arid1b	0.828
Pura	Rps3	0.828
Rai14	Med16	0.828
Polh	Pcna	0.828
Max	Tuba1a	0.828
Zfp236	Pparg	0.828
Zfp236	Fos	0.828
Myh3	Cryge	0.828
Egr2	Dab1	0.828
Tle4	Aes	0.828
Tmf1	Jak3	0.828
Nbr1	Bcr	0.828
Nbr1	Pdgfrb	0.828
Prkcd	Gria2	0.828
Rb1	E2f1	0.828
Mcl1	Fbxw7	0.828
Hbb-b1	Ldhb	0.828
Aldoa	Taf15	0.828
Aldoa	Psmc2	0.828
Aldoa	Tubb2c	0.828
Stat5b	Jak2	0.828
Sf3b2	Jun	0.828
Pparg	Tet2	0.828
Zfp110	Gli3	0.828
Ubb	Rpsa	0.828
Ubb	Prkdc	0.828
Tek	Cdh1	0.828
H1f0	Cdk5r1	0.828
Fancd2	Lyn	0.828
Fancd2	Zfp277	0.828
Thoc4	Rbm3	0.828
Dbnl	Cttn	0.828
Map3k1	Jun	0.828
Cdc37	Lrrk2	0.828

Snapc4	Foxh1	0.828
Hba-a1	Rps13	0.828
Hba-a1	Hnrnpm	0.828
Hsp90ab1	Nphp1	0.828
Suz12	H2afx	0.828
Hist3h2a	Usp7	0.828
Tbl1x	Nrip1	0.828
Tbrg1	Ngfr	0.828
Casp8	Flt3	0.828
Rfwd2	Fasn	0.827
Mef2a	Ppp2ca	0.827
Map2k4	Cd40	0.827
Shc1	Dok2	0.827
Epc1	Atf2	0.827
Krt10	Ahi1	0.827
Gpbp1	Mitf	0.827
Dok1	Nck2	0.827
Cd40	Traf3	0.827
Dcx	Dclk1	0.827
Rnh1	Hba-a1	0.827
Cd2ap	Ccnb1	0.827
Zbtb7c	Hdac3	0.827
Psmb5	Myh3	0.827
Psmb4	Stam2	0.827
Psmb1	Calm2	0.827
Tcerg1	Hnrnpab	0.827
Tcerg1	2500003M10	0.827
Usp2	Uimc1	0.827
Sod1	Ar	0.827
Sod1	Gria2	0.827
Sod1	Gli3	0.827
Ssbp3	Lhx9	0.827
Pura	Actr1a	0.827
Pura	Ppp2ca	0.827
Ahi1	Rps27a	0.827
Tle1	Zfp521	0.827
Ldb2	Lmx1a	0.827
Lmx1b	Zfp446	0.827
Hnf1a	Brpf1	0.827
Hnf1a	Foxh1	0.827
Tcf12	Cops5	0.827
Naglu	Aco1	0.827
Naglu	Ccr9	0.827
Max	Tpi1	0.827
Mapt	Dusp1	0.827
Hspa8	Myd88	0.827

Ncoa1	Nrip1	0.827
Nr6a1	Dnmt3b	0.827
Tle4	Msx1	0.827
Mixl1	Tardbp	0.827
Stat3	Tmf1	0.827
Magi2	Vdr	0.827
Cenpv	Mog	0.827
Ube2n	Map3k1	0.827
Stat5a	Agap2	0.827
Eya1	Neurod1	0.827
Pparg	Lhb	0.827
Ppargc1a	Hoxb1	0.827
Trip4	Med16	0.827
Zfp110	Flt1	0.827
Syn2	Ppp1cc	0.827
Grin2a	Notch4	0.827
Hba-a1	Eif3e	0.827
Cc2d2a	Tuba1b	0.827
Suz12	Uqcrc1	0.827
Rps13	Cycs	0.827
Vezf1	Hoxa2	0.827
Bag1	Tcf4	0.826
Bag1	Hoxa9	0.826
B2m	Ntrk1	0.826
Mecp2	Polr3f	0.826
Mecp2	Tet2	0.826
Psma4	Calm2	0.826
Epha2	Dsg1a	0.826
Epha2	Prss1	0.826
Epc1	Rarb	0.826
Cd40	Ikbkg	0.826
Rnh1	Calm2	0.826
Sin3b	Etv6	0.826
Cry1	Ezh2	0.826
Id3	Anxa2	0.826
Psmb5	Actn1	0.826
Pdia2	Hsp90b1	0.826
Usp2	Grb10	0.826
Usp2	Ccar1	0.826
Usp2	Nos2	0.826
Usp2	Foxo3	0.826
Sod1	Krt2	0.826
Sod1	Cops4	0.826
Sod1	Prdm16	0.826
Pura	Ppp1cb	0.826
Rai14	Srebf1	0.826

Tle1	Fos	0.826
Hnf1a	Tle6	0.826
Naglu	Glt1d1	0.826
Nr1i2	Tial1	0.826
Sox2	Pdx1	0.826
Etv6	Sfpi1	0.826
Egr2	Tsg101	0.826
Prkcd	Ntrk1	0.826
Bhlhe41	Neurog2	0.826
Bhlhe41	Ubb	0.826
Cenpv	Scmh1	0.826
Hbb-b1	Gnb2l1	0.826
Hbb-b1	Pdia6	0.826
Aldoa	Cfl1	0.826
Akr1b3	Fbxo6	0.826
Snca	Snap25	0.826
Trip4	Kat2b	0.826
Zfp110	Tbk1	0.826
Fancd2	Uchl1	0.826
Pbx2	Meis1	0.826
Smad2	Myod1	0.826
Hba-a1	Eprs	0.826
Setx	Jarid2	0.826
Med16	Nkd2	0.826
Gtf3c1	Phf2	0.826
Nup62	Nxt1	0.826
Gmnn	Spata24	0.826
Casp8	Mdm4	0.826
Vezf1	Strap	0.826
Topbp1	Ints3	0.826
Zfp263	Zfp473	0.825
Bag1	Bag4	0.825
Mapkbp1	Mapk8ip3	0.825
Mecp2	Cebpb	0.825
Ttc7b	Efr3b	0.825
Psma4	Krt2	0.825
Sorbs1	Map1lc3b	0.825
Sorbs1	Insr	0.825
Pik3r1	Irs2	0.825
Cyfip2	Abl2	0.825
Epha2	Yeats2	0.825
Gpbp1	Nfya	0.825
Cited4	Pcbd1	0.825
Cited4	Trim35	0.825
Meis3	Jdp2	0.825
Rnh1	Shank3	0.825

Rnh1	Ndufa4	0.825
Rnh1	Hist1h4a	0.825
Rnh1	Mtap1a	0.825
Rnh1	Nphp1	0.825
Bcl11b	Atr	0.825
Actbl2	Dsp	0.825
Id2	Cdk1	0.825
Id2	Med16	0.825
Homer1	Grid2	0.825
Psmb5	Notch1	0.825
Psmb5	Atxn3	0.825
Psmb4	Phf20	0.825
Psmb4	Tnk2	0.825
Psmb4	Skil	0.825
Rac1	Nf1	0.825
Rac1	Ctnnb1	0.825
Usp2	Mapt	0.825
Usp2	Camk1	0.825
Usp2	Mcl1	0.825
Eif3b	Ldb3	0.825
Eif3b	Gm5414	0.825
Eif3b	Hnrnpa1	0.825
Sod1	Nr3c2	0.825
Sod1	Pklr	0.825
Sod1	Was	0.825
Crk	Ywhah	0.825
Apba1	Lin7c	0.825
Pcgf2	Cbx7	0.825
Traf6	Ntrk1	0.825
Traf6	Stub1	0.825
Hist2h4	Ascl1	0.825
Ldb2	Tal2	0.825
Naglu	Actn1	0.825
Dusp1	Ppp2cb	0.825
Birc3	Flt3	0.825
Prkce	Rps6ka3	0.825
Bhlhe41	Hist3h2a	0.825
Hbb-b1	Rps13	0.825
Cyld	Dlg1	0.825
Akr1b3	Krt73	0.825
Sf3b2	Zmym2	0.825
Pparg	Pdgfrb	0.825
Ncoa6	Mll2	0.825
Trip4	Zfp764	0.825
Ubb	Nefm	0.825
H1f0	Ccna1	0.825

Fancd2	Gtf2f1	0.825
Fancd2	Csf1r	0.825
Dynll1	Tuba1a	0.825
Map3k5	Tax1bp1	0.825
Cdk1	Ccna1	0.825
Rps13	Psmc5	0.825
Hist3h2a	Olig2	0.825
Ubqln2	Eef2	0.825
Ptma	Ube2g2	0.825
Gab1	Tuba4a	0.825
Topbp1	Nol12	0.825
Topbp1	Cytsb	0.825
Bag1	Hadha	0.824
Bag1	Grin1	0.824
Map2k3	Map2k6	0.824
Mecp2	Ubtf	0.824
Shc1	Sla	0.824
Sorbs1	Lyn	0.824
Sorbs1	Flt1	0.824
Als2	Sqstm1	0.824
Krt10	Anxa1	0.824
Gpbp1	Taf6	0.824
Smad9	Psmc3	0.824
Dok1	Nck1	0.824
Cul1	Myog	0.824
Rnh1	Eif4a2	0.824
Rnh1	Nap1l4	0.824
Rnh1	Ccar1	0.824
Rnh1	Aifm1	0.824
Rnh1	Myl12b	0.824
Rnh1	Vdac2	0.824
Bcl11b	Cd2ap	0.824
Bcl11b	Tcf12	0.824
Bcl11b	Rps6ka3	0.824
Bcl11b	Psmc2	0.824
Myl9	Pex19	0.824
Actbl2	Mph1	0.824
Id3	Hnf1a	0.824
Cd2ap	Hck	0.824
Psmb5	Traf3	0.824
Psmb5	Ntrk2	0.824
Psmb5	Mycn	0.824
Psmb5	Sh3gl2	0.824
Psmb4	Ripk1	0.824
Psmb1	Mms19	0.824
LOC677235	Ybx1	0.824

Usp2	Psma7	0.824
Usp2	Tab3	0.824
Eif3b	Eprs	0.824
Eif3b	Asap2	0.824
Sfrs2	Fus	0.824
Crkl	Cblb	0.824
Rab3d	Usp9x	0.824
Pura	Psma1	0.824
Pura	Cdk1	0.824
Rai14	Pdcd6	0.824
Rai14	Neurog2	0.824
Pbx3	Hoxa6	0.824
Efs	Cav3	0.824
Egr1	Irak1	0.824
Mixl1	Hoxd12	0.824
Prkce	Mcl1	0.824
Bhlhe41	Ddx5	0.824
Cenpv	Csnk2b	0.824
Ube2i	Sfn	0.824
Ube2i	Daxx	0.824
Ube2e3	Ube2g2	0.824
Cyld	Nfkbia	0.824
Cyld	Ulk1	0.824
Akr1b3	Anxa1	0.824
Akr1b3	Ubqln2	0.824
Pparg	Bat1a	0.824
Ppargc1a	Jun	0.824
Trip4	Ddx5	0.824
Kdm4c	Lass2	0.824
Ubb	Prdx1	0.824
Ubb	Tsc22d3	0.824
Klf10	Smad5	0.824
Ddx5	Smad2	0.824
Dynll1	Pcbp1	0.824
Map3k5	Rnf11	0.824
Eif4a1	Atp5d	0.824
Hba-a1	Cycs	0.824
Hba-a1	Hnrnpa1	0.824
Lrp1	Dlg4	0.824
Suz12	Pcna	0.824
Suz12	Psme3	0.824
Suz12	Vav1	0.824
Rps13	Tceb2	0.824
Rpl7	Rps3	0.824
Topbp1	Tuba1c	0.824
Bat1a	Prkcsh	0.824

Msx2	Runx2	0.824
Socs3	Tceb1	0.823
Dvl2	Pklr	0.823
Bag1	Smad3	0.823
Mecp2	Rb1	0.823
Psma4	Hsph1	0.823
Psma4	Krt73	0.823
Psma4	Pcbp1	0.823
Krt10	Flnc	0.823
Krt10	Atp5a1	0.823
Cd40	Ube2n	0.823
Bcl11b	Pkm2	0.823
Id2	Pdlim4	0.823
Id3	Try10	0.823
Id3	Ccar1	0.823
Irf8	Fos	0.823
Id1	Smad4	0.823
Cd2ap	Nde1	0.823
Psmb5	Krt76	0.823
Psmb5	Ppp2cb	0.823
Psmb5	Sfn	0.823
Psmb4	Actn1	0.823
Psmd7	Psmd14	0.823
Invs	Myh9	0.823
Usp2	Mll1	0.823
Usp2	Ctnnb1	0.823
Usp2	Atxn3	0.823
Eif3b	Syncrip	0.823
Eif3b	Ube2v1	0.823
Eif3b	Ubqln2	0.823
Sod1	Krt15	0.823
Lmnb1	Ik	0.823
Iqcb1	Rps27a	0.823
Crkl	Rapgef1	0.823
Traf6	Htt	0.823
Traf6	Jun	0.823
Rab7	Bat1a	0.823
Rab3d	Aldoa	0.823
Pura	Ywhag	0.823
Tle1	E2f1	0.823
Ldb2	Lhx2	0.823
Naglu	Abca8a	0.823
Pdia3	Tubb4	0.823
Sox2	Msx2	0.823
Mapt	Vcp	0.823
Egr2	Snca	0.823

Bcl10	Irak1	0.823
Tmf1	Stat5a	0.823
Tmf1	Ski	0.823
Eya3	Smad4	0.823
Neurl2	Cryge	0.823
Nbr1	Itga5	0.823
Ncam1	E130012A19	0.823
Prkca	Lepr	0.823
Cenpv	Ptrf	0.823
Ube2n	Tax1bp1	0.823
Hbb-b1	Psmc2	0.823
Kdm4c	Hes6	0.823
Zfp110	Junb	0.823
Phc3	Hist3h2a	0.823
Irak1	Peli2	0.823
Smarce1	Brca1	0.823
Syn2	Hsp90ab1	0.823
Smad2	Mdm4	0.823
Dll4	Dll1	0.823
Rps13	Ube2v2	0.823
Hoxd10	Olig1	0.823
Ccna2	Cdkn1b	0.823
Zfp277	Phf17	0.823
Itpr3	Flot1	0.823
Mef2c	Gli2	0.823
Casp8	Cttn	0.823
Topbp1	Ap3s1	0.823
Map2k4	Mapk8	0.822
Mecp2	Per1	0.822
Npm1	Trp53	0.822
Epha2	Anxa1	0.822
Epc1	Bcl11b	0.822
Gpbp1	Lyn	0.822
Cited4	Sfpi1	0.822
Rnh1	Thrap3	0.822
Bcl11b	Kat2a	0.822
Bcl11b	Tle6	0.822
Bcl11b	Hif1a	0.822
Myl9	Itgb5	0.822
Cry2	Arntl	0.822
Id3	Naca	0.822
Id1	Ar	0.822
Cd2ap	Vcp	0.822
Psmb5	Arntl	0.822
Psmb4	Tuba1b	0.822
Fbxl2	Tcp1	0.822

Usp2	Arntl	0.822
Usp2	Vdac1	0.822
Usp2	Rnf14	0.822
Pcbd1	Hnf1b	0.822
Eif3b	Pkp1	0.822
Sod1	Pik3ca	0.822
Sod1	Anxa2	0.822
Lmnb1	Rbm10	0.822
Taf3	Hmga1	0.822
Gata3	Ppara	0.822
Mxi1	Smad5	0.822
Abl1	Dok3	0.822
Rab5b	Cdc37	0.822
Rab6	Rab27a	0.822
Rab3d	Cdh13	0.822
Pura	G3bp1	0.822
Pura	Rbm3	0.822
Ldb1	Ciita	0.822
Skp1a	Fbxw7	0.822
Pias3	Sp3	0.822
Erbb2	Egfr	0.822
Ccne1	H1	0.822
Sox2	Ifrd1	0.822
Sox2	Cdh1	0.822
Zfp236	Nr0b2	0.822
Dnajb6	Map2k7	0.822
Egr2	Cyld	0.822
Mixl1	Hoxa9	0.822
Nfkbiz	Nfkb1	0.822
Magi2	Snai1	0.822
Nbr1	Cebpa	0.822
Aph1b	Mbp	0.822
Cyld	Zfp110	0.822
Aldoa	Hspb1	0.822
Aldoa	Dnajb1	0.822
Aldoa	Hnrnpm	0.822
Stat5a	Jak2	0.822
Efna1	Sbds	0.822
Zfp473	Foxp1	0.822
Pparg	Cblb	0.822
Ppargc1a	Hdac3	0.822
Kdm4c	Jun	0.822
Ubb	Hspa9	0.822
Ubb	Tuba4a	0.822
Fancd2	Nfatc1	0.822
Syn2	Cbl	0.822

Syn2	Hspa1b	0.822
Was	Dlg1	0.822
Map3k5	Mapk8ip2	0.822
Med16	Trp73	0.822
Rps13	Eno3	0.822
Rps13	Myl12b	0.822
Zfp277	Hoxb4	0.822
Ltbr	Tax1bp1	0.822
Topbp1	Utp15	0.822
Fasn	Elf1	0.822
Map2k4	Tlr4	0.821
Gtf2a1l	Sox8	0.821
Shc3	Dlg2	0.821
Psma4	Tuba1b	0.821
Sorbs1	Irs1	0.821
Sorbs1	Cav3	0.821
Gpbp1	Sfpi1	0.821
Gpbp1	Msx1	0.821
Cited4	Aebp2	0.821
Cul1	Fbxl20	0.821
Myl9	Ttc4	0.821
Gtf2a1	Hnf1a	0.821
Irf8	Cebpa	0.821
Id1	Ascl3	0.821
Cd2ap	Sla	0.821
Ssbp2	Lmx1a	0.821
Psmb5	Krt73	0.821
Psmb4	Erbb2	0.821
Psmb1	Rcn2	0.821
Psmb1	Pcbp1	0.821
Invs	Tubb5	0.821
Usp2	Nlrx1	0.821
Usp2	Csnk1d	0.821
Usp2	Usp8	0.821
Usp2	Casp3	0.821
Usp2	Cebpa	0.821
Eif3b	Rps4x	0.821
Eif3b	Cycs	0.821
Sod1	Pten	0.821
Sod1	Mdm4	0.821
Lmnb1	Mtap	0.821
Lmnb1	Larp4b	0.821
Cdk5	Ccne1	0.821
Traf6	Lrrk2	0.821
Zfp369	Ntrk1	0.821
Hist2h4	Rpsa	0.821

Rai14	Zfp592	0.821
Tle1	Eed	0.821
Ldb2	Lmo1	0.821
Pias3	Msx1	0.821
Sox2	Pax3	0.821
Sox2	Hist4h4	0.821
Birc2	Map2k7	0.821
Tmf1	Nbr1	0.821
Eya3	Leo1	0.821
Prkce	Clybl	0.821
Ptprc	Cdh17	0.821
Rb1	Ppp1cc	0.821
Cyld	Smad2	0.821
Ankrd17	Tfrc	0.821
Aldoa	Gnb2l1	0.821
Tirap	Cblb	0.821
Snap25	Vamp2	0.821
Trip4	Trim27	0.821
Trip4	Med20	0.821
Phc3	Sp3	0.821
Dynll1	Trim2	0.821
Syn2	Mark4	0.821
Eif4a1	Telo2	0.821
Trim30	Ulk1	0.821
Hba-a1	Gm5414	0.821
Sufu	Sap18	0.821
Ifrd1	Sp1	0.821
Mapk7	Slc34a1	0.821
Mapk7	Ryk	0.821
Lef1	Smarcd3	0.821
Topbp1	Rbm5	0.821
Foxh1	Dlx5	0.821
Socs3	Tceb2	0.82
Bag1	Atp6v0d2	0.82
Bag1	Cops5	0.82
Mapkbp1	Cd40	0.82
Map2k4	Myd88	0.82
Mecp2	Sin3b	0.82
Mecp2	Gata4	0.82
Tnfaip3	Ube2d3	0.82
Psma4	Dynll1	0.82
Shc1	Dab1	0.82
Als2	Klhl17	0.82
Mark2	Ywhah	0.82
Gpbp1	Gtf2a1	0.82
Rad51	Bclaf1	0.82

Cited4	Mtf2	0.82
Meis3	Pbx4	0.82
Rnh1	Atp5b	0.82
Rnh1	Pkm2	0.82
Bcl11b	Kat5	0.82
Bcl11b	Gata4	0.82
Myl9	Tbrg1	0.82
Id2	Foxp1	0.82
Id2	Zfp292	0.82
Id2	Cebpa	0.82
Id2	Runx2	0.82
Id3	Krt16	0.82
Id3	Tubb6	0.82
Id1	Ep300	0.82
Psmb4	Nr1h3	0.82
Tcerg1	Gli2	0.82
LOC677235	Exoc4	0.82
Usp2	Msx2	0.82
Usp2	Rer1	0.82
Eif3b	Nrip2	0.82
Eif3b	Rpsa	0.82
Lmnb1	Nup88	0.82
Lmnb1	Cbx5	0.82
Lmnb1	Trim28	0.82
Hand1	Sox15	0.82
Traf2	Map3k5	0.82
Traf6	Igf1r	0.82
Traf6	Ski	0.82
Rab5b	Mbp	0.82
Pura	Usp8	0.82
Pura	Casp3	0.82
Pclo	Rims2	0.82
Sos1	Spry1	0.82
Zfp354a	Myb	0.82
Pias3	Polr2a	0.82
Sox2	Smarca4	0.82
Ndel1	Myo5a	0.82
Egr2	Ube4b	0.82
Xiap	Map3k2	0.82
Stat3	Irak1	0.82
Tmf1	Mc4r	0.82
Nbr1	Eif2c2	0.82
Nbr1	Prkcsh	0.82
Hbb-b1	Cycs	0.82
Aldoa	Hnrnpf	0.82
Aldoa	Ptrf	0.82

Aldoa	Fbln2	0.82
Aldoa	Hnrnpk	0.82
Sf3b2	Zfp128	0.82
Pparg	Nr5a1	0.82
Pparg	Insr	0.82
Ppargc1a	Tgfb1i1	0.82
Kdm4c	Lhx9	0.82
Zfp110	Msx2	0.82
Zfp110	Uimc1	0.82
H1f0	Foxp1	0.82
Fancd2	Ube4b	0.82
Thoc4	Ccnb1	0.82
Itpr1	Grid2	0.82
Smad2	Ndfip1	0.82
Smad2	Hoxa10	0.82
Fkbp14	Rpsa	0.82
Hba-a1	Rpl7	0.82
Suz12	Gtf2b	0.82
Suz12	Ldhb	0.82
Rps13	Pcbp1	0.82
Rps13	Ap2m1	0.82
Ubqln2	Hnrnpk	0.82
Lrrk2	Ctnnb1	0.82
Gmnn	Taf1c	0.82
Fbxw7	Pin1	0.82
Bag1	Sirt1	0.819
B2m	Stam2	0.819
Map2k6	Vav1	0.819
Mecp2	Sirt1	0.819
Mecp2	Gfi1b	0.819
Mecp2	Jun	0.819
Btrc	Reg1	0.819
Psma4	Ubxn2b	0.819
Shc1	Shank3	0.819
Shc1	Jak2	0.819
Als2	Arc	0.819
Rarg	Cebpa	0.819
Gpbp1	Sp3	0.819
Irf9	Hoxb8	0.819
Stk38	Ezh1	0.819
Synj1	Park2	0.819
Lancl2	Slc25a5	0.819
Rnh1	Pkp1	0.819
Bcl11b	Cdk6	0.819
Bcl11b	Gata3	0.819
Bcl11b	Ssbp3	0.819

Bcl11b	Cebpa	0.819
Sgk1	Scn8a	0.819
Sgk1	Scnn1a	0.819
Sin3a	Phf12	0.819
Id2	Phf2	0.819
Id3	Brca1	0.819
Cd2ap	Herpud1	0.819
Cd2ap	Gtf3c1	0.819
Homer1	Grm5	0.819
Psmb5	Mcl1	0.819
LOC677235	Inpp5e	0.819
Eif3b	Akr1b3	0.819
Eif3b	Ckm	0.819
Eif3b	Eif4b	0.819
Psen1	Notch3	0.819
Kit	Jak2	0.819
Traf3	Tlr4	0.819
Rab5b	Arhgef7	0.819
Pura	Skp2	0.819
Ripk1	Tax1bp1	0.819
Nkx3-2	Ikbkb	0.819
Hnf1a	Ncor2	0.819
Hist1h1a	Ccne1	0.819
Utrn	Sgcd	0.819
Esr1	Map1lc3b	0.819
Mtf2	Med16	0.819
Xiap	Pten	0.819
Nbr1	Ccar1	0.819
Tab2	Tmem115	0.819
Ube2i	Zfp277	0.819
Pax3	Lef1	0.819
Sf3b2	Mecom	0.819
Ubb	Pbx4	0.819
Ubb	Prpf31	0.819
Tek	Gab2	0.819
Fancd2	Itpr1	0.819
Snapc4	Olig1	0.819
Snapc4	Rorc	0.819
Gtf3c1	Suz12	0.819
Angptl2	Fez1	0.819
Slc25a4	Pdia6	0.819
Rps13	Rpl7	0.819
Ubqln2	Myh1	0.819
Lmo3	Tal1	0.819
Cdk2	Cdkn1a	0.819
Nrip1	Lass2	0.819

Topbp1	Cdc5l	0.819
Topbp1	Fam98a	0.819
Fbxw8	Cul1	0.818
Dvl2	Smurf1	0.818
Bag1	Pxn	0.818
Bag1	Tollip	0.818
Mef2a	Pbx1	0.818
Mef2a	Runx2	0.818
Map2k4	Map2k7	0.818
Mecp2	Tcf21	0.818
Mecp2	Pax3	0.818
Gtf2a1l	Zfp292	0.818
Shcbp1	Mov10	0.818
Shc1	Crkl	0.818
Shc1	Sh3bp2	0.818
Als2	Dlg4	0.818
Flna	Myh9	0.818
Flna	Krt2	0.818
Cited4	Ets2	0.818
Phc1	Mapkapk2	0.818
Rnh1	Eif4b	0.818
Bcl11b	H1f0	0.818
Sgk1	Scn10a	0.818
Sgca	Nos1	0.818
Actbl2	Myh3	0.818
Id2	Tsc22d3	0.818
Id3	Ncl	0.818
Id1	Cdkn1b	0.818
Cd2ap	Psmd7	0.818
Raf1	Gab1	0.818
Psmb5	Ptk2	0.818
Psmb5	Smurf1	0.818
Psmb5	Hspb1	0.818
Psmb4	Casp8	0.818
Psmb1	Actn1	0.818
Rac1	Camk2b	0.818
Usp2	Stat3	0.818
Usp2	Bcr	0.818
Usp2	Cttn	0.818
Hnrnpa2b1	Cct2	0.818
Eif3b	Ptrf	0.818
Eif3b	Nr1h4	0.818
Sod1	Krt79	0.818
Rab3d	Ptrf	0.818
Pura	Rps16	0.818
Pura	Ewsr1	0.818

Ldb1	Med16	0.818
Hnf1a	Kat2b	0.818
Naglu	Actn4	0.818
Naglu	Cdc7	0.818
Psen2	Sri	0.818
Usp9x	Gnao1	0.818
Bcl6	Rxrg	0.818
Max	Zfp111	0.818
Sox2	Reg1	0.818
Sox2	Phf12	0.818
Mapt	Erg	0.818
Mtf2	Rps27a	0.818
Etv6	Rrm1	0.818
Zfp236	Ssbp4	0.818
Egr2	Nfe2l2	0.818
Mixl1	Gtf2e2	0.818
Nbr1	Casp3	0.818
Nbr1	Tardbp	0.818
Ncam1	Hspa5	0.818
Hbb-b1	Rpl22	0.818
Aldoa	Nr0b2	0.818
Pax5	Myb	0.818
Ncoa6	Morf4l1	0.818
Zfp110	Rer1	0.818
Zfpm1	Rbl2	0.818
Irak1	Il1rap	0.818
Grin2a	Kcnd2	0.818
Smad2	Flt3	0.818
Suz12	Vdac1	0.818
Rps13	Asap2	0.818
Rps13	Hnrnpa1	0.818
Rpl7	Pdlim7	0.818
Tbrg1	Rpl9	0.818
Tbp	Taf1c	0.818
Casp8	Hras1	0.818
Casp8	Cebpb	0.818
Topbp1	Aplf	0.818
Topbp1	Zfml	0.818
Bag1	Cenpv	0.817
Bag1	Uqcrc1	0.817
Mecp2	Bsx	0.817
Mecp2	Dzip3	0.817
Mecp2	Ankra2	0.817
Gtf2a1l	Phf17	0.817
Psma4	Rnh1	0.817
Shc1	Spag9	0.817

Shc1	Cd300a	0.817
Shc1	Skp2	0.817
Epha2	Flnc	0.817
Rarg	Tle6	0.817
Rad51	Fasn	0.817
Cited4	Hoxb1	0.817
Cd40	Mapk8	0.817
Rnh1	Psmb1	0.817
Rnh1	Fbxo6	0.817
Rnh1	Clta	0.817
Actbl2	Rplp0	0.817
Id2	Zfp277	0.817
Irf8	Fas	0.817
Cd2ap	Map1lc3b	0.817
Psmb5	Ctcf	0.817
Psmb4	Keap1	0.817
Psmb4	Sykb	0.817
Tcerg1	Nid2	0.817
Tcerg1	Ctnnb1	0.817
Usp2	Hdac6	0.817
Usp2	Gli3	0.817
Usp2	Epas1	0.817
Eif3b	Mdh2	0.817
Sod1	Ptk2	0.817
Sod1	Hspa8	0.817
Ahi1	Krt2	0.817
Ripk1	Cyld	0.817
Rai14	Phf17	0.817
Sox2	Slc1a2	0.817
Sox2	Ulk1	0.817
Mapt	Ilk	0.817
Mapt	Stat3	0.817
Dnajb6	Rps6ka3	0.817
Rnf19a	Sufu	0.817
Egr2	Xiap	0.817
Mixl1	Dlx5	0.817
Mixl1	Dmrtc2	0.817
Cops8	Gsk3b	0.817
Nbr1	Map3k2	0.817
Nbr1	Rer1	0.817
Prkcb	Neurod1	0.817
Prkce	Diap1	0.817
Cenpv	Suz12	0.817
Ube2i	Phf2	0.817
Hbb-b1	Anxa1	0.817
Hbb-b1	Cops4	0.817

Cyld	Ndfip1	0.817
Aldoa	Tuba1a	0.817
Akr1b3	Tubb6	0.817
Cbx1	Trim28	0.817
Sf3b2	Hif1a	0.817
Pparg	Ripk2	0.817
Rhoq	Pxn	0.817
Zfp110	Slc1a2	0.817
Capn2	Dusp3	0.817
Eif4a1	Pdia6	0.817
Eif4a1	Eif3e	0.817
Rbx1	Sf3b1	0.817
Itpr1	Nploc4	0.817
Gps1	Uchl1	0.817
Suz12	Morc3	0.817
Suz12	Aebp2	0.817
Hdac3	Jun	0.817
Sertad1	Lass2	0.817
Zfp277	Gtf2h2	0.817
Zfp277	Dlx5	0.817
Cul5	Socs2	0.817
Lef1	Cdh1	0.817
Map2k3	Dusp2	0.816
Tnfaip3	Ripk1	0.816
Psma4	Krt15	0.816
Shc1	Tyk2	0.816
Sorbs1	Sla2	0.816
Pik3r1	Grb10	0.816
Hist1h1e	Cbx3	0.816
Epha2	Lin54	0.816
Usf1	Etv4	0.816
Bcl11b	Snap25	0.816
Bcl11b	Itpr1	0.816
Bcl11b	Cebpb	0.816
Myl9	Nucb1	0.816
Myl9	Hap1	0.816
Tcf3	Smarca4	0.816
Per2	Per3	0.816
Id2	Olig2	0.816
Id3	Actb	0.816
Cd2ap	Ptpn11	0.816
Psmb5	Nr1h3	0.816
Rac1	Cttn	0.816
Smad6	Runx2	0.816
LOC677235	Wdr37	0.816
Usp2	Sod1	0.816

Usp2	Scn8a	0.816
Usp2	Scnn1a	0.816
Usp2	Sirt1	0.816
Usp2	Gsc	0.816
Usp2	Ripk2	0.816
Usp2	Cdh1	0.816
Eif3b	Rpl7	0.816
Sod1	Ski	0.816
Sod1	Flt3	0.816
Lmnb1	Nup214	0.816
Lmnb1	Tor1aip1	0.816
Lmnb1	Top2a	0.816
Nkx2-5	Mef2c	0.816
Itgb1bp2	Hsp90aa1	0.816
Traf6	Ndn	0.816
Traf6	Grin1	0.816
Rab5b	Taok1	0.816
Bub1	Dapk1	0.816
Pura	Hist1h1a	0.816
Pura	Ina	0.816
Ahi1	Tubb5	0.816
Bax	Ppp1cc	0.816
Naglu	Hnrnpf	0.816
Naglu	Nisch	0.816
Max	Trim32	0.816
Mapt	Calr	0.816
Mtf1	Ssbp4	0.816
Mtf2	Aebp2	0.816
Mtf2	Hdac10	0.816
Dnajb6	Myocd	0.816
Pou2f1	Aes	0.816
Flnc	Hist1h2ai	0.816
Birc3	Mapk8ip1	0.816
Actb	Nphp1	0.816
Tmf1	Map3k2	0.816
Nbr1	Cebpb	0.816
Prkce	Fus	0.816
Ube2l3	Chuk	0.816
Aldoa	Myo1c	0.816
Akr1b3	Myog	0.816
Akr1b3	Rps13	0.816
Trip4	Snapc4	0.816
Ubb	Cdh2	0.816
Pxn	Arhgef7	0.816
H1f0	Plec	0.816
Fancd2	Dnmt1	0.816

Thoc4	Nr3c1	0.816
Dbnl	Casp3	0.816
Dynll1	Calm2	0.816
Dynll1	Hspa4	0.816
Dynll1	Eprs	0.816
Map3k5	Mapk14	0.816
Smad4	Lef1	0.816
Hba-a1	Mdh2	0.816
Cblb	Nfatc1	0.816
Suz12	Atp5d	0.816
Pcna	Cdkn1a	0.816
Mcm6	Lhx2	0.816
Pdlim5	Enah	0.816
Lef1	Ctnna1	0.816
Topbp1	Ncapg2	0.816
Topbp1	Mphosph10	0.816
Bat1a	Hras1	0.816
Foxh1	Dlx1	0.816
Irf1	Sumo1	0.816
Bag1	Hdac3	0.815
Mecp2	Rorc	0.815
Gtf2a1l	Nfkbia	0.815
Psma4	Exoc4	0.815
Shc1	Inpp5d	0.815
Cited4	Mixl1	0.815
Mtap2	Grin1	0.815
Bcl11b	Atm	0.815
Sgk1	Scn3a	0.815
Id3	Rai14	0.815
Id3	Krt6a	0.815
Id3	Ciita	0.815
Id3	Tubb5	0.815
Psmb5	Pkd2	0.815
Psmb5	Myog	0.815
Psmb4	Cct6a	0.815
Psmb4	Ngfr	0.815
Psmb1	Pcmtd1	0.815
Psmb1	Rps27a	0.815
Usp2	Mpl	0.815
Sod1	Krt76	0.815
Sod1	Telo2	0.815
Sod1	Ulk1	0.815
Atp6v1a	Traf6	0.815
Gata4	Med1	0.815
Tle1	Arntl	0.815
Tle1	Kdm5b	0.815

Psen2	Eea1	0.815
Esr1	Sh3bp2	0.815
Sox2	Pparg	0.815
Mapt	Ube2n	0.815
Mtf2	Thoc4	0.815
Egr2	Prkdc	0.815
Egr2	Foxo3	0.815
Gtf2e2	Tardbp	0.815
Tmf1	Fert2	0.815
Tmf1	Uchl1	0.815
Gsc	Cacna1c	0.815
Crebbp	Rela	0.815
Nbr1	Ube2n	0.815
Nbr1	Ppp2cb	0.815
Nbr1	Usp8	0.815
Prkce	Calm1	0.815
Bhlhe41	Kat2b	0.815
Pparg	Nfe2l2	0.815
Ubb	Myod1	0.815
Tle6	Foxp1	0.815
Fancd2	Prkdc	0.815
Map3k1	Mapk8	0.815
Eif4a1	Rps13	0.815
Smad2	Cflar	0.815
Smad2	Mycn	0.815
Hba-a1	Hnrnpk	0.815
Cc2d2a	Krt42	0.815
Suz12	Rps13	0.815
Rps13	Ptrf	0.815
Iqgap1	Cdc42	0.815
Zfp277	Phf2	0.815
Zfp277	H3f3a	0.815
Rpl7	Hspa9	0.815
Tbp	Sumo1	0.815
Bat1a	Rab5a	0.815
Bat1a	Flt1	0.815
Cd3e	Vav1	0.815
Dll1	Notch1	0.815
Bag1	Ldhb	0.814
Map2k4	Chuk	0.814
Ptprq	Ctnna1	0.814
Trim66	Chaf1a	0.814
Rcor2	Lmo3	0.814
Ncstn	Hsp90b1	0.814
Dok1	Inpp5d	0.814
Cd40	Birc3	0.814

Card11	Sash1	0.814
Rnh1	Cep290	0.814
Rnh1	Mtap6	0.814
Bcl11b	Ccnb1	0.814
Actbl2	Eef1g	0.814
Id3	Bag2	0.814
Id3	Amfr	0.814
Id3	Zdhhc15	0.814
Cd2ap	Sh2b2	0.814
Homer2	Homer1	0.814
Psmb5	Smn1	0.814
Psmb5	Hsp90aa1	0.814
Zfand2a	Psma1	0.814
Rac1	Dlg2	0.814
Usp2	Traf3	0.814
Usp2	Ctcf	0.814
Usp2	Notch1	0.814
Hnrnpa2b1	Ccnd2	0.814
Eif3b	Hnrnpk	0.814
Eif3b	Rps5	0.814
Sod1	Traf6	0.814
Sod1	Vim	0.814
Sod1	Atp5d	0.814
Sod1	Foxo3	0.814
Sod1	Hnrnpa1	0.814
Gtf2h1	Ankrd49	0.814
Pcgf2	Mph1	0.814
Traf6	Nphp1	0.814
Bub1	Anapc7	0.814
Hcst	Klrk1	0.814
Rai14	Abtb1	0.814
Zfp354a	Ubr5	0.814
Efs	Kdr	0.814
Mapt	Sykb	0.814
Mtf2	BC018507	0.814
Mtf2	Nap1l4	0.814
Tctn1	Krt2	0.814
Tctn1	Rps27a	0.814
Egr2	Plagl2	0.814
Birc3	Ltbr	0.814
Tmf1	Csf1r	0.814
Rbck1	Rnf31	0.814
Bhlhe41	Bahd1	0.814
Akr1b3	Cycs	0.814
Akr1b3	Tuba1b	0.814
Akr1b3	Eif2a	0.814

Tirap	Trim8	0.814
Itgb1	Cdh17	0.814
Trim35	Jarid2	0.814
Kdm4c	Ppp1cc	0.814
Kdm4c	Lef1	0.814
Ubb	Tuba1a	0.814
Smad2	Junb	0.814
Snapc4	Ing1	0.814
Hba-a1	Purb	0.814
Hba-a1	Alb	0.814
Hsp90aa1	Nphp1	0.814
Gtf3c1	Hoxd12	0.814
Suz12	Fasn	0.814
Cask	Mpp5	0.814
Top2a	Eif3i	0.814
Sf3b1	Dgcr8	0.814
Casp8	Cdh1	0.814
Lef1	Prdm16	0.814
Topbp1	Arfgap2	0.814
Bat1a	Hsp90b1	0.814
Bat1a	Cebpb	0.814
Cav1	Flot1	0.814
Bag1	Dctn1	0.813
Bag1	Ckm	0.813
Bag1	Efhd2	0.813
B2m	Was	0.813
Map2k2	Ptk2b	0.813
Mecp2	Per2	0.813
Mecp2	Hnf1a	0.813
Shc1	Dok1	0.813
Shc1	Pxn	0.813
Shc1	Nfatc1	0.813
Pik3r1	Irs1	0.813
Als2	Actg1	0.813
Epha2	Cryab	0.813
Epc1	Tle6	0.813
LOC674895	Map2k1	0.813
Flna	Jup	0.813
Krt10	Invs	0.813
Gpbp1	Nf1	0.813
Cited4	Hnf1a	0.813
Axin1	Lrrk2	0.813
Rnh1	Med14	0.813
Actbl2	Krt14	0.813
Id2	Sfn	0.813
Cd2ap	Cdk6	0.813

Ssbp2	Lmo4	0.813
Psmb5	Uchl1	0.813
Psmb4	Psmb1	0.813
Tcerg1	Sumo1	0.813
Usp2	Pea15b	0.813
Usp2	Tsnax	0.813
Eif3b	Acta1	0.813
Sod1	Tmf1	0.813
Cdk5	Ccnd1	0.813
Tcf4	Tcf12	0.813
Crkl	Zap70	0.813
Traf6	Poli	0.813
Rab3d	Ndufa9	0.813
Rab4a	Map3k5	0.813
Naglu	Pcbp1	0.813
Esr1	Pou6f1	0.813
Mtf2	Polr3f	0.813
Zfp236	Pax6	0.813
Ctnnd1	Lrp1	0.813
Egr2	Ufd1l	0.813
Birc2	Tab2	0.813
Birc2	Mapk8ip1	0.813
Nbr1	Uchl1	0.813
Nbr1	Slc1a2	0.813
Prkca	Cul5	0.813
Bhlhe41	Polr1a	0.813
Aldoa	Rnf11	0.813
Sall4	Hdac1	0.813
Sf3b2	Sumo1	0.813
Pparg	Fem1a	0.813
Kdm4c	Klf4	0.813
Kdm4c	Smarcd3	0.813
Zfp110	Psmc3	0.813
Ubb	Eif4a1	0.813
Ubb	Zfp292	0.813
Tek	Fos	0.813
Fancd2	Cebpb	0.813
Dynll1	Hsp90ab1	0.813
Cdk1	Pcna	0.813
Mpnd	Fez1	0.813
Mdc1	Nbn	0.813
Angptl2	Uqcrc1	0.813
Ptma	Rorc	0.813
Ruvbl2	Nrbp1	0.813
Sf3b1	Hnrnpk	0.813
Nrip1	Ncor1	0.813

Vezf1	Fasn	0.813
Map2k4	Pik3r1	0.812
Ap1b1	Cited2	0.812
Mecp2	Gon4l	0.812
Mecp2	Kdm5b	0.812
Shc3	Cav3	0.812
Psma4	Myh10	0.812
Psma4	Tubb5	0.812
Shc1	Traf6	0.812
Shc1	Ppp2r3a	0.812
Sorbs1	Cd40	0.812
Epha2	Ndufa4	0.812
Usf1	Nupr1	0.812
Gpbp1	E2f1	0.812
Axin2	Ctnnb1	0.812
Rnh1	Prkdc	0.812
Bcl11b	Pitx2	0.812
Per1	Per3	0.812
Id2	Pbx4	0.812
Id2	Hist3h2a	0.812
Id2	Psmd10	0.812
Id3	Pkp1	0.812
Id3	Psmd11	0.812
Irf8	Irf1	0.812
Cd2ap	Nedd4	0.812
Cdk6	Ccnd1	0.812
Cdk6	Cdkn1b	0.812
Suv420h1	Rbl1	0.812
Psmb5	Bcr	0.812
Psmb4	Krt15	0.812
Rnf2	Hdac2	0.812
LOC677235	Arhgap33	0.812
Usp2	Fbxl3	0.812
Eif3b	Pkm2	0.812
Eif3b	Aldoa	0.812
Sod1	Krt8	0.812
Sod1	Cand2	0.812
Sod1	Mapk3	0.812
Sod1	Hdac6	0.812
Sod1	Rpl7	0.812
Slc2a4	Flot1	0.812
Pura	Dbnl	0.812
Tcf12	Bhlhe41	0.812
Hist1h1a	Cdk2	0.812
Esr1	Hhex	0.812
Esr1	Gli1	0.812

Mapt	Nlrx1	0.812
Egr2	Tab3	0.812
Mixl1	Ewsr1	0.812
Tmf1	Smurf1	0.812
Tmf1	Sh3gl2	0.812
Foxn2	Hoxb4	0.812
Dag1	Cav3	0.812
Nbr1	Prkcd	0.812
Hsf1	Hdac6	0.812
Hbb-b1	Mog	0.812
Aldoa	Tnfrsf13c	0.812
Aldoa	Ubap2l	0.812
Akr1b3	Tubb2b	0.812
Hnf4a	Ppargc1a	0.812
Pparg	Cdkn1a	0.812
Kdm4c	Ctnnb1	0.812
Zfp110	Fancd2	0.812
Zfp110	Reg1	0.812
Dnm1	Tuba1a	0.812
Dynll1	Myo1c	0.812
Syn2	Tufm	0.812
Eif4a1	Eprs	0.812
Grin2a	Cttn	0.812
Klrb1c	Nr1i3	0.812
Mapk8	Jun	0.812
Rps13	Eif2a	0.812
Rps13	Eif3e	0.812
Hist3h2a	Hif1a	0.812
Slit3	Ighmbp2	0.812
Vezf1	Vav1	0.812
Topbp1	Ap2a1	0.812
Ap1b1	Eif4g3	0.811
Mecp2	Ccne1	0.811
Mecp2	Nfkb1	0.811
Mecp2	Jarid2	0.811
Shc1	Phf20	0.811
Flna	Krt77	0.811
Rarg	Hif1a	0.811
Jup	Pcp2	0.811
Itch	Tax1bp1	0.811
Rnh1	Glud1	0.811
Rnh1	Invs	0.811
Rnh1	Hsp90ab1	0.811
Bcl11b	Cul2	0.811
Bcl11b	Ncoa1	0.811
Bcl11b	Atp5d	0.811

Id2	Cdk4	0.811
Cd2ap	Nfatc1	0.811
Rac1	Vdac1	0.811
Nedd9	Gnb2l1	0.811
Usp2	Dusp1	0.811
Usp2	Tab2	0.811
Usp2	Dlg1	0.811
Eif3b	Tuba3a	0.811
Sod1	Poli	0.811
Lmna	Iqcb1	0.811
Iqcb1	Actb	0.811
Gfi1	Pias3	0.811
Gata6	Med1	0.811
Epm2a	V1rd20	0.811
Traf3	Birc3	0.811
Traf6	Fos	0.811
Bub1	Crem	0.811
Ccdc101	Map2k7	0.811
Tcf12	Dot1l	0.811
Tcf12	Polr1a	0.811
Psen2	Plp1	0.811
Max	Tsix	0.811
Dnajb6	H1f0	0.811
Tmf1	Bcr	0.811
Tmf1	Ppp2cb	0.811
Foxn2	Gtf2h2	0.811
Nbr1	Mapk3	0.811
Hbb-b1	Hnrnpa1	0.811
Cyld	Hhex	0.811
Cyld	Fos	0.811
Aldoa	Rps13	0.811
Akr1b3	Adrm1	0.811
Med1	Bmi1	0.811
Ppargc1a	Zfp111	0.811
Ncoa6	Cbfb	0.811
Trip4	Dnmt1	0.811
Kdm4c	Mef2d	0.811
Zfp110	Sykb	0.811
Uba52	Atxn2	0.811
H1f0	Cdkn1b	0.811
Fancd2	Brca1	0.811
Pbx1	Hoxa6	0.811
Eif4a1	Hba-a1	0.811
Smad2	Pex6	0.811
Angptl2	Sobp	0.811
Ifrd1	Gon4l	0.811

Topbp1	Rbm8a	0.811
Bat1a	Tax1bp1	0.811
Trpc4	Cav1	0.811
Jun	Ctnnb1	0.811
Bag1	Ezh2	0.81
Bag1	Rela	0.81
Mef2a	Ywhab	0.81
Map2k2	Cttn	0.81
Ap1b1	Csk	0.81
Shc1	Nbr1	0.81
Shc1	Clybl	0.81
Sorbs1	Pik3r1	0.81
Sorbs1	Smad7	0.81
Col5a1	Smad3	0.81
Dok1	Rasa1	0.81
Cd40	Birc2	0.81
Rnh1	Hsph1	0.81
Rnh1	Pi4ka	0.81
Rnh1	Pcbp1	0.81
Bcl11b	Map2k1	0.81
Bcl11b	Bax	0.81
Dync1h1	Kif5b	0.81
Id2	Jarid2	0.81
Id3	Eif4a2	0.81
Irf8	Rhoa	0.81
Psmb4	Nap1l4	0.81
Psmb1	Dynll1	0.81
Rac1	Hras1	0.81
Aip	Calcoco1	0.81
LOC677235	Frmd4b	0.81
Usp2	Foxp1	0.81
Usp2	Runx2	0.81
Hnrnpa2b1	Adcy8	0.81
Eif3b	Cops4	0.81
Sod1	Krt6a	0.81
Iqcb1	Nphp1	0.81
Traf6	Chordc1	0.81
Hist2h4	Ikbkg	0.81
Tcf12	Rarb	0.81
Mapt	Cflar	0.81
Nlrx1	Ikbkg	0.81
Dnajb6	Ifrd1	0.81
Ncoa3	Lass2	0.81
Egr2	Rela	0.81
Hbb-b1	Ldb3	0.81
Aldoa	Ube2d3	0.81

Akr1b3	Cops4	0.81
Cbx2	Bmi1	0.81
Gm10358	Actr1a	0.81
Sf3b2	Rnf111	0.81
Zfp473	Ssbp4	0.81
Pparg	Ms4a2	0.81
Kdm4c	Myod1	0.81
Zfp110	Skil	0.81
Pxn	Limk1	0.81
Dbnl	Aph1a	0.81
Syn2	Dclk1	0.81
Syn2	Hdac2	0.81
Eif4a1	Eno3	0.81
Cdk1	Ppp1cc	0.81
Smad1	Psmc3	0.81
Hba-a1	Ubqln2	0.81
Chmp5	Tbrg1	0.81
Rps13	Rpl22	0.81
Gab1	Csf1r	0.81
Tbp	Plagl2	0.81
Casp8	Cdkn1a	0.81
Casp8	Foxo3	0.81
Dr1	Zzz3	0.81
Bag1	Vcp	0.809
Mecp2	Ncoa1	0.809
Mecp2	Lass5	0.809
Tnfaip3	Tnfrsf1a	0.809
Gtf2a1l	Gata6	0.809
Gtf2a1l	Sfpi1	0.809
Psma4	Atp5a1	0.809
Epha2	Actc1	0.809
Rad51	Etv4	0.809
Cited4	Hist3h2a	0.809
Rnh1	Bag2	0.809
Bcl11b	Syt1	0.809
Sh3kbp1	Inpp5d	0.809
Cry1	Fbxl3	0.809
Id2	Hoxa10	0.809
Id3	Rnf14	0.809
Id1	Myf5	0.809
Suv420h1	Rbl2	0.809
Psmb5	Tardbp	0.809
Psmb4	Myh3	0.809
Psmb4	Krt77	0.809
Psmb4	Fyn	0.809
Psmb4	Prkcsh	0.809

Psmb1	Krt2	0.809
Usp2	Slc1a2	0.809
Eif3b	Ap2a1	0.809
Cmtm3	Map4k1	0.809
Sod1	Ywhaz	0.809
Sod1	Atg16l1	0.809
Lmnb1	Hnrnpu	0.809
Lmnb1	Nfe2l2	0.809
Gtf2h1	Gtf2e1	0.809
Sirt1	Ppargc1a	0.809
Rab7	Anxa3	0.809
Pura	Atp5b	0.809
Ldb2	Lhx4	0.809
Ccnd1	Cdkn1a	0.809
Psen2	Cacna1c	0.809
Efs	Prkce	0.809
Esr1	Pxn	0.809
Tlr4	Ikbkg	0.809
Max	Iqgap1	0.809
Sox3	Chek1	0.809
Mapt	Nbr1	0.809
Mapt	Stub1	0.809
Mapt	Fbxo2	0.809
Egr2	Sp1	0.809
Sgcz	Sgcb	0.809
Tmf1	Cblb	0.809
Tmf1	Usp8	0.809
Musk	Pak1	0.809
Nbr1	Irak1	0.809
Ncam1	Cntn1	0.809
Prkce	Peli2	0.809
Cenpv	Gnb2l1	0.809
Cenpv	Plp1	0.809
Hbb-b1	Suz12	0.809
Ncoa6	Taf15	0.809
Ncoa6	Runx1	0.809
Zfp110	Pten	0.809
Phc3	Cbx4	0.809
Ubb	Dsp	0.809
Ubb	Gria2	0.809
Zfpm1	Pdx1	0.809
Il1r1	Irak1	0.809
Dbnl	Sfn	0.809
Chordc1	Hsp90ab1	0.809
Eef1a1	Dclk1	0.809
Chmp5	Tada3	0.809

Vcp	Ufd1l	0.809
Suz12	Ptrf	0.809
Rps13	Hnrnpk	0.809
Ubqln2	Kars	0.809
Rpl7	Rps5	0.809
Casp8	Jun	0.809
Topbp1	Mad2l1	0.809
Bat1a	Eif3i	0.809
Fasn	Nup155	0.809
Cops3	Gps1	0.808
Csnk1e	Per2	0.808
Ldlrap1	Topbp1	0.808
Map2k3	Stk39	0.808
Map2k4	Map3k7	0.808
Gjb3	Gmcl1	0.808
Psma4	Gabra1	0.808
Epha2	Rnh1	0.808
Flna	Tubb5	0.808
Rad51	Kat5	0.808
Zbtb3	Zbtb39	0.808
Bcl11b	Rarb	0.808
Actbl2	Pcgf1	0.808
Irf8	Dlg1	0.808
Cd2ap	Abl1	0.808
Zbtb7c	Ncor1	0.808
Cul3	Ezh2	0.808
Psmb5	Gata6	0.808
Tcerg1	Rfx2	0.808
Tcerg1	Ap2m1	0.808
LOC677235	Dock7	0.808
Usp2	Ube2n	0.808
Sod1	Lasp1	0.808
Sod1	Eif2c2	0.808
Ywhab	Arhgef7	0.808
Fadd	Cflar	0.808
Fadd	Casp8	0.808
Psen1	Aph1b	0.808
Rab3d	Stxbp1	0.808
Rab3d	Ldha	0.808
Pura	Ywhaz	0.808
Pura	Ccna1	0.808
Stoml3	Adcy3	0.808
Esr1	Rhoq	0.808
Esr1	Gsk3a	0.808
Sox2	Mdm2	0.808
Etv6	Tet2	0.808

Etv6	Clock	0.808
Sla2	Was	0.808
Egr2	Plk1	0.808
Birc2	Mib2	0.808
Tmf1	Camk1	0.808
Magi2	Chuk	0.808
Nbr1	Pparg	0.808
Nbr1	Ripk2	0.808
Prkcd	Ubb	0.808
Prkce	Sh3bp2	0.808
Hbb-b1	Acta1	0.808
Cyld	Sykb	0.808
Akr1b3	Hist1h2ai	0.808
Tirap	Jak2	0.808
Smarcb1	Fos	0.808
Zfp446	Isl2	0.808
Zfp473	Zscan2	0.808
Ppargc1a	Nr1h4	0.808
Phc3	Tfdp1	0.808
Ubb	Hspb1	0.808
Dok3	Cbl	0.808
H1f0	Ltb	0.808
Capn2	Pcyt1a	0.808
Eef1a1	Actn1	0.808
Hba-a1	Pcbp1	0.808
Chmp5	Ttc4	0.808
Polr1b	Taf1b	0.808
Suz12	Strap	0.808
Hist3h2a	Rxrg	0.808
Hist3h2a	Foxo3	0.808
Hoxd10	Hoxa11	0.808
Rpl7	Fbln2	0.808
Sf3b1	Usp7	0.808
Tbp	Nrip1	0.808
Bag1	Bax	0.807
Bag1	Gnb2l1	0.807
Bag1	Vdac2	0.807
Acaa2	Actn4	0.807
Ap1b1	Rnps1	0.807
Mecp2	Bcl11b	0.807
Gtf2a1l	Gmnn	0.807
Flna	Hsp90aa1	0.807
Gpbp1	Cnot3	0.807
Cited4	Khdrbs1	0.807
Cited4	E2f1	0.807
Spna1	Grin1	0.807

Rnh1	Eef2	0.807
Id3	Ercc8	0.807
Id3	Taf15	0.807
Id3	Spdef	0.807
Raf1	Hras1	0.807
Raf1	Tubg1	0.807
Psmb5	Tnk2	0.807
Psmb4	Reg1	0.807
Invs	Hspa8	0.807
Tcerg1	Lims1	0.807
Usp2	Ptk2	0.807
Usp2	App	0.807
Usp2	Ccne1	0.807
Usp2	Per3	0.807
Usp2	Pdgfrb	0.807
Sod1	Gsc	0.807
Gtf2i	Egr2	0.807
Ptk2	Bcl2	0.807
Tcf7l1	Esr1	0.807
Traf2	Birc3	0.807
Traf6	Irf3	0.807
Rab3d	Slc1a2	0.807
Ccdc101	Nup88	0.807
Barx2	Srf	0.807
Efs	Csf1r	0.807
Esr1	Diap1	0.807
Dnajb6	Atm	0.807
Egr2	Bcl2	0.807
Egr2	Cd79a	0.807
Egr2	Vdac1	0.807
Stat3	Stat1	0.807
Ncor2	Nr1h4	0.807
Krt77	Nphp1	0.807
Nbr1	Pten	0.807
Prkcd	Zfp110	0.807
Prkce	Dnm1	0.807
Bhlhe41	Pcna	0.807
Cenpv	Usp7	0.807
Cyld	Hsp90aa1	0.807
Aldoa	Vcp	0.807
Smurf1	Pard6a	0.807
Pik3c2b	Arhgap31	0.807
Pparg	Thra	0.807
Ncoa6	Usp7	0.807
H1f0	Cdkn1a	0.807
Gm7611	Dclk2	0.807

Nfe2l2	Ptma	0.807
Map3k7	Mib2	0.807
Map3k7	Peli2	0.807
Ppia	Hist4h4	0.807
Ubqln2	Mylpf	0.807
Zfp277	Rps3	0.807
Tceb1	Asb2	0.807
Rpl7	Fbxw7	0.807
Ppp5c	Dlg2	0.807
Src	Csf1r	0.807
Topbp1	Nup155	0.807
Ctnnb1	Cdh1	0.807
Rara	Nrip1	0.806
Mef2a	Col1a1	0.806
Map2k6	Atf2	0.806
Map2k6	Peli2	0.806
Mecp2	Smyd1	0.806
Mecp2	Snapc4	0.806
Mecp2	Hoxb1	0.806
Psma4	Krt42	0.806
Shc1	Msx2	0.806
Shc1	Lepr	0.806
Ptprq	Ctnnd1	0.806
Npm1	Tcf3	0.806
Als2	Ap2b1	0.806
Epha2	Rps27a	0.806
Epc1	Actg1	0.806
Flna	Psma1	0.806
Usf1	Tal1	0.806
Usf1	Foxo3	0.806
Rad51	Dnm1l	0.806
Stk38	Eed	0.806
Dok1	Bcr	0.806
Rnh1	Dab1	0.806
Sgk1	Scn2a1	0.806
Cd2ap	Ap2b1	0.806
Cd2ap	Foxp1	0.806
Cd2ap	Cblb	0.806
Homer1	Itpr1	0.806
Psmb4	Birc3	0.806
Psmb1	Krt77	0.806
Usp2	Scn5a	0.806
Usp2	Mitf	0.806
Eif3b	Grip1	0.806
Sod1	Calr	0.806
Sod1	Sfn	0.806

Sod1	Hnrnpm	0.806
Lmnb2	Lmnb1	0.806
Iqcb1	Hspa8	0.806
Iqcb1	Tuba1b	0.806
Tcf7l2	Hnf4a	0.806
Pkd2	Vcp	0.806
Traf6	Zfp110	0.806
Hist2h4	Ep300	0.806
Pura	Irs1	0.806
Pura	Rfpl4	0.806
Pura	Rpsa	0.806
Ahi1	Mks1	0.806
Tle1	Ezh2	0.806
Hnf1a	Emx1	0.806
Tcf12	Zfp277	0.806
Cops2	Setdb1	0.806
Ccnd1	Ppp1cc	0.806
Usp9x	Neurl2	0.806
Max	Bcl2	0.806
Mapt	Casp9	0.806
Pou2f1	Hoxb8	0.806
Egr2	Myod1	0.806
Pias1	Sufu	0.806
Birc3	Mdm4	0.806
Ezh2	Suz12	0.806
Prkce	Map1lc3b	0.806
Ube2i	Olig2	0.806
Cyld	Ngfr	0.806
Aldoa	Hsp90ab1	0.806
Aldoa	Eprs	0.806
Akr1b3	Pcbp1	0.806
Trip4	Polr1a	0.806
Zfpm1	Mef2d	0.806
Syn2	Nedd4l	0.806
Eif4a1	Prpf31	0.806
Eif4a1	Phf2	0.806
Grin1	Dlg1	0.806
Smad4	Smarcd3	0.806
Polr1b	Atrx	0.806
Arrb2	Nbn	0.806
Trim67	D3Bwg0562e	0.806
Gmnn	Tbp	0.806
Src	Ctnnb1	0.806
Nrip1	Ap2a2	0.806
Bat1a	Fos	0.806
Oaz3	Ociad1	0.806

Socs1	Jak2	0.805
Bag1	Pfn2	0.805
Tet1	Ifrd1	0.805
Map2k4	Traf3	0.805
Mecp2	Pitx2	0.805
Mecp2	Sox9	0.805
Shc1	Reg1	0.805
Rnf41	Traf6	0.805
Krt10	Jup	0.805
Krt10	Tubb6	0.805
Rnh1	Psmb5	0.805
Tcf3	Myod1	0.805
Cry1	Cry2	0.805
Id2	Tle6	0.805
Id3	Try5	0.805
Id3	Myh9	0.805
Irf8	Slc1a2	0.805
Golgb1	Naglu	0.805
Psmb4	Krt42	0.805
Psmb1	Bag2	0.805
LOC677235	Kif21b	0.805
Usp2	Scn9a	0.805
Eif3b	Eif4a1	0.805
Sod1	Hgs	0.805
Gtf2h1	Trip4	0.805
Iqcb1	Krt2	0.805
Traf2	Ltbr	0.805
Traf6	Cflar	0.805
Ccdc101	Gspt1	0.805
Ccdc101	Etv3	0.805
Ccdc101	Topbp1	0.805
Pklr	Smurf2	0.805
Naglu	Esyt1	0.805
Mll1	H3	0.805
Esr1	Jak2	0.805
Mtf2	Zfp82	0.805
Nphp3	Krt2	0.805
Nphp3	Tpi1	0.805
Flnc	Krt78	0.805
Kat2a	Mbip	0.805
Birc2	Ticam1	0.805
Mixl1	Dlx2	0.805
Tmf1	Lyn	0.805
Ube2l3	Ikbkb	0.805
Hbb-b1	Eif2a	0.805
Hbb-b1	Hnrnpk	0.805

Aldoa	Il17ra	0.805
Aldoa	Map3k7	0.805
Trip4	Ankrd49	0.805
Kdm4c	Atrx	0.805
Zfp110	Mc4r	0.805
Zfpm1	Hist4h4	0.805
D930014E17	Sh3bp2	0.805
Syn2	Enah	0.805
Eif4a1	Tsc22d3	0.805
Eif4a1	Hnrnpa1	0.805
Ubqln2	Mutyh	0.805
Tbrg1	Mcm10	0.805
Lef1	Pgr	0.805
Ell	Jarid2	0.805
Bag1	Smarcb1	0.804
Bach1	Myod1	0.804
Ap1b1	Atad5	0.804
Shc1	Irf5	0.804
Epc1	Taf15	0.804
Flna	Krt14	0.804
Gpbp1	Lass2	0.804
Rad51	Map2k1	0.804
Rad51	Brca2	0.804
Rad51	Strap	0.804
Cited4	Pygo1	0.804
Lancl2	Dclk2	0.804
Card11	Tmem115	0.804
Rnh1	Akr1b3	0.804
Dync1h1	Nde1	0.804
Id3	Rpsa	0.804
Psmb5	Erbb2	0.804
Psmb5	Casp3	0.804
Psmb4	Polk	0.804
Psmb4	Myh9	0.804
Psmb4	Aldoa	0.804
Kdm4b	Arid1b	0.804
LOC677235	Cacnb1	0.804
Usp2	Park2	0.804
Usp2	Dlg3	0.804
Usp2	Mdm2	0.804
Sod1	Adrbk1	0.804
Sod1	Myod1	0.804
Sod1	Psmc5	0.804
Cdk5	Suds3	0.804
Traf6	Tirap	0.804
Rab3d	Gnao1	0.804

Hist2h4	Psmd2	0.804
Ccdc101	Nup214	0.804
Ccdc101	Nckap1l	0.804
Sh2b2	Insr	0.804
Rai14	Lmx1b	0.804
App	Stub1	0.804
Zfp354a	Vezf1	0.804
Pias3	Taf10	0.804
Psen2	Psenen	0.804
Mtf2	Clock	0.804
Nphp3	Krt73	0.804
Etv6	Atxn1	0.804
Dnajb6	Bclaf1	0.804
Ncoa3	Lgals3	0.804
Egr2	Mc4r	0.804
Birc3	Map3k1	0.804
Mixl1	Hoxb13	0.804
Nbr1	Pax6	0.804
Nbr1	Ube4b	0.804
Cenpv	Hbb-b1	0.804
Hbb-b1	Myog	0.804
Hbb-b1	Vdac2	0.804
Tirap	Ifngr1	0.804
Rhoq	Flot1	0.804
Ubb	Tle6	0.804
Ubb	Map4k4	0.804
Isl2	Ssbp4	0.804
Tek	Ncor1	0.804
Fancd2	Pcyt1a	0.804
Syn2	Mtap6	0.804
Eif4a1	Ube2v2	0.804
Eif4a1	Ubqln2	0.804
Eif4a1	Rpl22	0.804
Eif4a1	Asap2	0.804
Smad2	Sox15	0.804
Snapc4	Ankra2	0.804
Hba-a1	Eif2a	0.804
Mapk1	Mdk	0.804
Rps13	Rpl28	0.804
Ubqln2	Psmd2	0.804
Ubqln2	9130011E15I	0.804
Ppp5c	Hoxb8	0.804
Prmt5	Pabpc1	0.803
Bag1	Vdac1	0.803
Map2k6	Ube2n	0.803
Mecp2	Sufu	0.803

Mecp2	E2f1	0.803
Psma4	Cct3	0.803
Psma4	Prim1	0.803
Sorbs1	Dok3	0.803
Rnf41	Tbk1	0.803
Hist1h1e	Chaf1a	0.803
Epc1	Pitx2	0.803
Flna	Krt73	0.803
Rarg	Cebpb	0.803
Krt10	Krt78	0.803
Krt10	Hnrnpm	0.803
Cited4	Rsrc1	0.803
Cited4	Nfe2l2	0.803
Dok2	Dok1	0.803
Rnh1	Cox5a	0.803
Bcl11b	Hap1	0.803
Id3	Hsp90aa1	0.803
Cd2ap	Gtf2e1	0.803
Ngly1	Vcp	0.803
Psmb5	Krt8	0.803
Psmb5	Rhoa	0.803
Psmb4	Hdac6	0.803
Rac1	Pla2g4a	0.803
Usp2	Stat1	0.803
Usp2	Myod1	0.803
Lmnb2	Ttn	0.803
Traip	Traf3ip2	0.803
Hist2h4	Pdcd6	0.803
Pura	Tceb2	0.803
Ccdc101	Kat2a	0.803
Ccdc101	Fasn	0.803
Lcp2	Blnk	0.803
Lmx1b	Ssbp4	0.803
Tcf12	Zdhhc15	0.803
Tcf12	Taf15	0.803
Slc11a1	Cyld	0.803
Cops2	Thrb	0.803
Prlr	Stat5a	0.803
Efs	Prkaca	0.803
Esr1	Clybl	0.803
Esr1	Gab1	0.803
Mtf2	Snapc4	0.803
Zfp236	Ppargc1a	0.803
Ndel1	Ppp1r9b	0.803
Egr2	Rnf115	0.803
Adam10	Cask	0.803

Foxn2	Dlx5	0.803
Magi2	Ddx5	0.803
Nbr1	Hdac5	0.803
Prkcb	Fus	0.803
Prkce	Il1r1	0.803
Prkce	Ppp5c	0.803
Bhlhe41	Eif4a2	0.803
Ube2i	Sun1	0.803
Ube2i	Irf1	0.803
Hbb-b1	Syn1	0.803
Hbb-b1	Dclk2	0.803
Cyld	Rnf31	0.803
Ankrd17	Ipo9	0.803
Akr1b3	Mdh2	0.803
Ubb	Adam6b	0.803
Zfpm1	Sip1	0.803
Zfpm1	Phf12	0.803
Fancd2	Dlg4	0.803
Dbnl	Syn1	0.803
Dynll1	Tfrc	0.803
Eif4a1	Cycs	0.803
Eif4a1	Zfp292	0.803
Eif4a1	Rpsa	0.803
Eif4ebp1	Vrk3	0.803
Eef1a1	Rps13	0.803
Smad2	Lef1	0.803
Snapc4	Srebf1	0.803
Hba-a1	Prdm16	0.803
Smc1a	Pds5a	0.803
Rps13	Cand1	0.803
Rps13	Eprs	0.803
Ppia	Kdm5b	0.803
Arrb2	Hipk1	0.803
Zfp277	Sirt2	0.803
Rpl7	Prkcsh	0.803
Top2a	Rab5a	0.803
Casp8	Mpl	0.803
Lef1	Sox8	0.803
Nrip1	Prdx5	0.803
Shc1	Met	0.802
Sorbs1	Crk	0.802
Pik3r1	Dok1	0.802
Epc1	Ring1	0.802
Rarg	Bcl11b	0.802
Esrrb	Rnf2	0.802
Stk38	Ezh2	0.802

Tnfrsf1a	Ripk1	0.802
Cul1	Skp2	0.802
Rnh1	Nucb1	0.802
Rnh1	Tuba1a	0.802
Bcl11b	Sap18	0.802
Actbl2	Eno1	0.802
Id3	Id1	0.802
Irf8	Fam175b	0.802
Pias4	Srebf1	0.802
Raf1	Dlg4	0.802
Psmb5	Sun2	0.802
Psmb5	Ulk1	0.802
Psmb5	Dlg1	0.802
Psmb4	Adrbk1	0.802
Psmb4	Csf1r	0.802
Invs	Ahi1	0.802
Eif3b	Ap2m1	0.802
Sod1	Uimc1	0.802
Pcp2	Gnao1	0.802
Vangl2	Dvl3	0.802
Crk	Arhgap33	0.802
Traf6	Nphp4	0.802
Traf6	Nfkbia	0.802
Traf6	Sh3gl2	0.802
Rab3d	Nhlrc1	0.802
Rai14	Polr3f	0.802
Tle1	Ppia	0.802
Gjb6	Smad5	0.802
Hnf1a	Ppargc1a	0.802
Naglu	Tatdn2	0.802
Orc2l	Zfp111	0.802
Ush1c	Plekhb1	0.802
Ccnd1	Rb1	0.802
Ptpn11	Gab1	0.802
Nphp3	Krt15	0.802
Tctn1	Krt15	0.802
Tctn1	Tubb5	0.802
Malt1	Card10	0.802
Nbr1	Vav1	0.802
Prkca	Prkce	0.802
Prkcd	Mapk6	0.802
Prkcd	Casp8	0.802
Prkce	Reg1	0.802
Prkce	Ppp2ca	0.802
Hsf1	Srebf1	0.802
Aldoa	Eef2	0.802

Nfkb1	Relb	0.802
Sf3b2	Smad3	0.802
Pparg	Vcp	0.802
Zfp110	Ticam1	0.802
Uba52	Cfl1	0.802
Uba52	Fbln2	0.802
Fancd2	Tsg101	0.802
Fancd2	Mpl	0.802
Fancd2	Cdkn1a	0.802
Fancd2	Fos	0.802
Dbnl	Usp8	0.802
Eif4a1	Psmc5	0.802
Eef1a1	Mib2	0.802
Rbx1	Fbxo6	0.802
Ssbp4	Lhx9	0.802
Smad5	Psmc3	0.802
Spry3	Spry1	0.802
Angptl2	Huwe1	0.802
Rps13	Tceb1	0.802
Ifrd1	Sp3	0.802
Zfp277	Asb1	0.802
Mef2c	Sap18	0.802
Tbl1x	Pin1	0.802
Casp8	Pcyt1a	0.802
Bat1a	Sh3gl2	0.802
Fasn	Ddx41	0.802
Bag1	Ctnnb1	0.801
Ldlrap1	Etv3	0.801
Mecp2	Ep300	0.801
Mecp2	Zfp82	0.801
Tnfaip3	Tax1bp1	0.801
Shc3	Nos1	0.801
Psma4	Psma2	0.801
Psma4	Psma6	0.801
L3mbtl2	Ewsr1	0.801
Rad51	Cd2ap	0.801
Rad51	Nr3c1	0.801
Cited4	Myod1	0.801
Cited4	Jarid2	0.801
Cul1	Fbxo2	0.801
Bcl11b	Nhlrc1	0.801
Sgca	Sgcg	0.801
Sin3b	Ddx20	0.801
Ubtf	Rb1	0.801
Id3	Nupr1	0.801
Cd2ap	Rnps1	0.801

Cd2ap	Egfr	0.801
Psmb4	Zfand2a	0.801
Psmb4	Hspa8	0.801
Psmb4	Was	0.801
Psmb4	Fam175b	0.801
Psmb1	Psma7	0.801
Psmb1	Kpnb1	0.801
Psmb1	Myh9	0.801
Usp2	Scnn1b	0.801
Usp2	Gata6	0.801
Usp2	Stub1	0.801
Usp2	Uchl1	0.801
Hnrnpa2b1	Anxa1	0.801
Eif3b	Pcbp1	0.801
Sod1	Krt73	0.801
Sod1	Tpi1	0.801
Sod1	Vcp	0.801
Sod1	Mpl	0.801
Sod1	Ube4b	0.801
Gm5920	Actr1a	0.801
2900073G15	Grin1	0.801
Traf6	Nlrx1	0.801
Rab5b	Cdc42	0.801
Rab3d	Foxp1	0.801
Bub1	H3f3a	0.801
Rai14	Hdac10	0.801
Tcf12	Eif4a1	0.801
Hist1h1a	Cdk4	0.801
Naglu	Tfrc	0.801
Ccne1	Cdk2	0.801
Egfr	Src	0.801
Esr1	Tle4	0.801
Max	Dnajb6	0.801
Max	Rps6ka3	0.801
Ctnnd1	Ctnnb1	0.801
Ywhaz	Aph1a	0.801
Magi2	Sf3b2	0.801
Nbr1	Fyn	0.801
Ube2n	Map3k7	0.801
Ube2l3	Sufu	0.801
Hbb-b1	Eif3e	0.801
Cyld	Map3k1	0.801
Aldoa	Ikbkg	0.801
Aldoa	Wwp1	0.801
Akr1b3	Ldb3	0.801
Rrp8	Topbp1	0.801

Ppargc1a	Nfya	0.801
Ncoa6	Cbx4	0.801
Ppara	Gfi1b	0.801
Ubb	Hoxb9	0.801
Tek	Grb2	0.801
Fancd2	Hsp90aa1	0.801
Fancd2	Psmd2	0.801
Smarce1	Tbx21	0.801
Dbnl	H2afx	0.801
Dynll1	Hspb1	0.801
Eif4a1	Zfp277	0.801
Eif4a1	Pdlim4	0.801
Eef1a1	Irf7	0.801
Spry3	Spry4	0.801
Rela	Hdac1	0.801
Cdk2	Trp53	0.801
Ptma	Fos	0.801
Sf3b1	Rorc	0.801
Sf3b1	E2f6	0.801
Casp8	Ube4b	0.801
Casp9	Myd88	0.801
Bat1a	Cdkn1a	0.801
Fasn	Mad2l1	0.801
Gli3	Hist1h2ai	0.801
Esrra	Ppargc1a	0.8
Tmem43	Ptpn13	0.8
Ap1b1	Rps19bp1	0.8
Ap1b1	Gata3	0.8
Mecp2	Epc1	0.8
Shc1	Igf1r	0.8
Shc1	Ulk1	0.8
Pik3r1	Fyn	0.8
Krt10	Nphp1	0.8
Gpbp1	Taf1a	0.8
Cited4	Fos	0.8
Esrrb	Smarca4	0.8
Lancl2	Exoc4	0.8
Bcl11b	Epm2a	0.8
Bcl11b	Cox5a	0.8
Bcl11b	Ep300	0.8
Actbl2	Csnk2a2	0.8
Tcf3	Neurog2	0.8
Id2	Cdk9	0.8
Id2	Cebpb	0.8
Psmb4	Ipo9	0.8
Psmb4	Casp3	0.8

Sod1	Casp3	0.8
Sod1	Hnrnpk	0.8
Lmnb1	Cbx3	0.8
Lmnb1	Nphp1	0.8
Gm5920	Frmd4b	0.8
Ywhab	Crk	0.8
Pcp2	Actn1	0.8
Polk	Smn1	0.8
Traf6	Dok3	0.8
Ccnt1	Mllt1	0.8
Hnf1a	Otx2	0.8
Naglu	Pi4ka	0.8
Slc11a1	Cbfb	0.8
Nr2f6	Gmeb1	0.8
Tmsb4x	Ilk	0.8
Max	Map2k7	0.8
Mapt	Pdcd6ip	0.8
Mapt	Psmc3	0.8
Ctnnd1	Grin1	0.8
Ndel1	Eef1a2	0.8
Birc3	Nos2	0.8
Mks1	Krt78	0.8
Prkcb	Ppp3ca	0.8
Bhlhe41	Zfp292	0.8
Cenpv	Atp5d	0.8
Ube2i	Rnf14	0.8
Cyld	Atg16l1	0.8
Akr1b3	Telo2	0.8
Tirap	Tubb2c	0.8
Ncoa6	Csnk2b	0.8
Ncoa6	Ep400	0.8
Zfp110	Trim24	0.8
Smarce1	Ikbkg	0.8
Syn2	Arhgef6	0.8
Map3k7	Rnf31	0.8
Eif4a1	Gm5414	0.8
Eef1a1	Cycs	0.8
Ctr9	Ruvbl2	0.8
Smad2	Cttn	0.8
Tbrg1	Iffo2	0.8
Tbrg1	Tyw1	0.8
Casp8	Runx2	0.8
Fasn	Lpxn	0.8
Sp1	Jun	0.8
