# Supplementary material for: Connectivity Homology Enables Inter-Species Network Models of Synthetic Lethality
Source: PLoS Comput Biol. 2015 Oct 9;11(10):e1004506. doi: 10.1371/journal.pcbi.1004506 (PMC4599967; doi:10.1371/journal.pcbi.1004506)
Supplement: S5 Table — (PDF) [file pcbi.1004506.s021.pdf]

Gene 1		Gene 2		SINaTRA Score
ID	Symbol	ID	Symbol	
28998	MRPL13	65080	MRPL44	0.962
28998	MRPL13	23256	SCFD1	0.954
5983	RFC3	5981	RFC1	0.965
5983	RFC3	5884	RAD17	0.98
5983	RFC3	23476	BRD4	0.974
83540	NUF2	11130	ZWINT	0.968
273	AMPH	10188	TNK2	0.971
273	AMPH	6453	ITSN1	0.954
273	AMPH	160	AP2A1	0.951
273	AMPH	50618	ITSN2	0.953
273	AMPH	1785	DNM2	0.966
273	AMPH	23523	CABIN1	0.961
9525	VPS4B	10534	SSSCA1	0.968
9525	VPS4B	9736	USP34	0.957
9525	VPS4B	7298	TYMS	0.966
9525	VPS4B	8882	ZNF259	0.96
9525	VPS4B	9559	VPS26A	0.954
9525	VPS4B	283237	TTC9C	0.972
9525	VPS4B	10277	UBE4B	0.959
9527	GOSR1	6811	STX5	0.959
9252	RPS6KA5	51776	ZAK	0.956
84126	ATRIP	11073	TOPBP1	0.98
10892	MALT1	84433	CARD11	0.967
10892	MALT1	5588	PRKCQ	0.977
10892	MALT1	9099	USP2	0.979
22878	KIAA1012	83696	TRAPPC9	0.961
29763	PACSIN3	7456	WIPF1	0.982
29763	PACSIN3	7454	WAS	0.963
29763	PACSIN3	6453	ITSN1	0.966
29763	PACSIN3	1785	DNM2	0.968
23054	NCOA6	9757	MLL4	0.969
23054	NCOA6	51586	MED15	0.964
7185	TRAF1	8717	TRADD	0.96
7185	TRAF1	8737	RIPK1	0.957
7185	TRAF1	55072	RNF31	0.958
7185	TRAF1	9020	MAP3K14	0.952
7185	TRAF1	8837	CFLAR	0.951
7185	TRAF1	10616	RBCK1	0.95
7187	TRAF3	8737	RIPK1	0.957
7187	TRAF3	4591	TRIM37	0.962
7187	TRAF3	4804	NGFR	0.955
7187	TRAF3	4615	MYD88	0.95
7187	TRAF3	868	CBLB	0.962

7187	TRAF3	10133	OPTN	0.954
7187	TRAF3	51567	TTRAP	0.955
7456	WIPF1	7454	WAS	0.951
7456	WIPF1	11252	PACSIN2	0.979
7454	WAS	3702	ITK	0.964
7453	WARS	158	ADSL	0.965
7453	WARS	56474	CTPS2	0.963
7453	WARS	9329	GTF3C4	0.964
7453	WARS	7511	XPNPEP1	0.975
65080	MRPL44	65005	MRPL9	0.976
65080	MRPL44	54534	MRPL50	0.967
65080	MRPL44	28977	MRPL42	0.958
65080	MRPL44	64975	MRPL41	0.956
65080	MRPL44	64978	MRPL38	0.962
55626	AMBRA1	1468	SLC25A10	0.956
55626	AMBRA1	57521	RPTOR	0.954
55626	AMBRA1	23192	ATG4B	0.95
55626	AMBRA1	8408	ULK1	0.965
2319	FLOT2	11160	ERLIN2	0.975
2319	FLOT2	10211	FLOT1	0.954
65260	C1orf163	57466	SFRS15	0.966
4729	NDUFV2	4728	NDUFS8	0.969
4729	NDUFV2	4720	NDUFS2	0.969
4729	NDUFV2	4722	NDUFS3	0.961
4729	NDUFV2	4723	NDUFV1	0.952
4726	NDUFS6	4720	NDUFS2	0.96
4720	NDUFS2	4722	NDUFS3	0.964
4720	NDUFS2	4723	NDUFV1	0.955
4722	NDUFS3	4723	NDUFV1	0.958
11284	PNKP	3980	LIG3	0.953
11284	PNKP	7518	XRCC4	0.95
51100	SH3GLB1	26128	KIAA1279	0.951
51100	SH3GLB1	3267	AGFG1	0.952
51100	SH3GLB1	5528	PPP2R5D	0.956
10392	NOD1	8767	RIPK2	0.957
10393	ANAPC10	11065	UBE2C	0.951
10393	ANAPC10	9232	PTTG1	0.97
10393	ANAPC10	10297	APC2	0.971
6810	STX4	6844	VAMP2	0.958
6810	STX4	8673	VAMP8	0.974
6810	STX4	9217	VAPB	0.966
6810	STX4	8773	SNAP23	0.976
6810	STX4	8775	NAPA	0.958
6817	SULT1A1	7169	TPM2	0.957
6817	SULT1A1	7320	UBE2B	0.95
6817	SULT1A1	7347	UCHL3	0.952

6817	SULT1A1	146691	TOM1L2	0.952
6817	SULT1A1	7171	TPM4	0.95
6817	SULT1A1	11075	STMN2	0.95
9757	MLL4	9070	ASH2L	0.95
8848	TSC22D1	4091	SMAD6	0.966
8915	BCL10	7132	TNFRSF1A	0.953
1762	DMWD	23239	PHLPP1	0.953
58533	SNX6	3020	H3F3A	0.954
58533	SNX6	5074	PAWR	0.988
58533	SNX6	10533	ATG7	0.979
58533	SNX6	327	APEH	0.975
58533	SNX6	6470	SHMT1	0.981
58533	SNX6	6642	SNX1	0.967
58533	SNX6	8723	SNX4	0.956
58533	SNX6	81611	ANP32E	0.95
58533	SNX6	55737	VPS35	0.955
58533	SNX6	64855	FAM129B	0.957
58533	SNX6	51699	VPS29	0.961
8717	TRADD	7124	TNF	0.958
8717	TRADD	355	FAS	0.967
8717	TRADD	7133	TNFRSF1B	0.965
79705	LRRK1	3635	INPP5D	0.965
79705	LRRK1	6654	SOS1	0.97
79705	LRRK1	613	BCR	0.956
9520	NPEPPS	6197	RPS6KA3	0.965
6778	STAT6	8648	NCOA1	0.958
6774	STAT3	960	CD44	0.951
6776	STAT5A	2057	EPOR	0.963
6777	STAT5B	3643	INSR	0.967
6777	STAT5B	58490	RPRD1B	0.963
9968	MED12	7067	THRA	0.957
9968	MED12	80306	MED28	0.955
9969	MED13	79991	OBFC1	0.95
9969	MED13	9325	TRIP4	0.976
9969	MED13	9444	QKI	0.95
3891	KRT85	9101	USP8	0.968
84108	PCGF6	23269	MGA	0.964
84108	PCGF6	7027	TFDP1	0.957
84108	PCGF6	1876	E2F6	0.958
64928	MRPL14	51069	MRPL2	0.954
22818	COPZ1	1729	DIAPH1	0.951
22818	COPZ1	3831	KLC1	0.961
22818	COPZ1	26958	COPG2	0.959
22818	COPZ1	7090	TLE3	0.961
112939	NACC1	54880	BCOR	0.956
10188	TNK2	9564	BCAR1	0.979

10188	TNK2	6478	SIAH2	0.951
10188	TNK2	7525	YES1	0.957
10188	TNK2	5159	PDGFRB	0.969
1155	TBCB	23071	ERP44	0.971
1155	TBCB	4643	MYO1E	0.951
23071	ERP44	10979	FERMT2	0.969
23071	ERP44	5062	PAK2	0.971
23071	ERP44	11235	PDCD10	0.962
23071	ERP44	5198	PFAS	0.953
112869	CCDC101	51562	MBIP	0.959
29127	RACGAP1	23164	MPRIP	0.969
29127	RACGAP1	9493	KIF23	0.957
8520	HAT1	8314	BAP1	0.97
4299	AFF1	4297	MLL	0.981
4299	AFF1	10614	HEXIM1	0.979
4299	AFF1	23476	BRD4	0.97
4299	AFF1	904	CCNT1	0.969
4297	MLL	904	CCNT1	0.965
4297	MLL	9070	ASH2L	0.953
4296	MAP3K11	6416	MAP2K4	0.955
4296	MAP3K11	5609	MAP2K7	0.964
23380	SRGAP2	55763	EXOC1	0.954
81567	TXNDC5	9689	BZW1	0.951
55644	OSGEP	7165	TPD52L2	0.971
55644	OSGEP	26128	KIAA1279	0.95
55644	OSGEP	8882	ZNF259	0.973
55644	OSGEP	51002	TPRKB	0.975
55644	OSGEP	573	BAG1	0.95
55644	OSGEP	58490	RPRD1B	0.957
55644	OSGEP	60491	NIF3L1	0.965
55644	OSGEP	8270	LAGE3	0.971
55644	OSGEP	64175	LEPRE1	0.965
55644	OSGEP	26136	TES	0.972
55644	OSGEP	51569	UFM1	0.965
55644	OSGEP	10055	SAE1	0.953
5867	RAB4A	79874	RABEP2	0.964
5861	RAB1A	4860	PNP	0.958
5861	RAB1A	8766	RAB11A	0.95
5861	RAB1A	136319	MTPN	0.956
5861	RAB1A	7879	RAB7A	0.964
5861	RAB1A	9230	RAB11B	0.971
5861	RAB1A	523	ATP6V1A	0.959
5861	RAB1A	2665	GDI2	0.978
2186	BPTF	6594	SMARCA1	0.954
2189	FANCG	675	BRCA2	0.967
57646	USP28	8314	BAP1	0.959

57646	USP28	64219	PJA1	0.951
57646	USP28	8078	USP5	0.954
57646	USP28	23032	USP33	0.986
57646	USP28	7347	UCHL3	0.967
57646	USP28	8027	STAM	0.959
57646	USP28	9101	USP8	0.966
57646	USP28	63967	CLSPN	0.954
159	ADSS	9948	WDR1	0.967
159	ADSS	3417	IDH1	0.964
159	ADSS	3336	HSPE1	0.968
159	ADSS	6888	TALDO1	0.958
159	ADSS	29082	CHMP4A	0.956
159	ADSS	54108	CHRAC1	0.96
159	ADSS	5763	PTMS	0.975
159	ADSS	1633	DCK	0.976
158	ADSL	22978	NT5C2	0.962
158	ADSL	55666	NPLOC4	0.964
158	ADSL	10226	PLIN3	0.969
158	ADSL	8539	API5	0.97
158	ADSL	7511	XPNPEP1	0.96
158	ADSL	29978	UBQLN2	0.97
158	ADSL	10785	WDR4	0.966
158	ADSL	10055	SAE1	0.968
6900	CNTN2	4801	NFYB	0.97
9191	DEDD	8837	CFLAR	0.955
9191	DEDD	843	CASP10	0.951
6238	RRBP1	58477	SRPRB	0.966
6238	RRBP1	10084	PQBP1	0.962
6238	RRBP1	5937	RBMS1	0.973
6238	RRBP1	4701	NDUFA7	0.957
91	ACVR1B	4091	SMAD6	0.965
8861	LDB1	51592	TRIM33	0.966
8861	LDB1	6886	TAL1	0.961
811	CALR	6455	SH3GL1	0.954
813	CALU	51194	IPO11	0.952
813	CALU	10094	ARPC3	0.971
815	CAMK2A	5071	PARK2	0.959
57132	CHMP1B	51510	CHMP5	0.965
11130	ZWINT	79003	MIS12	0.952
11130	ZWINT	79980	DSN1	0.964
11130	ZWINT	10403	NDC80	0.972
3020	H3F3A	10951	CBX1	0.95
3020	H3F3A	55723	ASF1B	0.95
55726	C12orf11	26173	INTS1	0.956
8737	RIPK1	7188	TRAF5	0.971
8737	RIPK1	7128	TNFAIP3	0.956

8737	RIPK1	7124	TNF	0.957
8737	RIPK1	1540	CYLD	0.964
8737	RIPK1	331	XIAP	0.953
8737	RIPK1	355	FAS	0.979
8737	RIPK1	64127	NOD2	0.969
8737	RIPK1	8837	CFLAR	0.963
8737	RIPK1	8767	RIPK2	0.959
8737	RIPK1	8887	TAX1BP1	0.967
8737	RIPK1	843	CASP10	0.978
8737	RIPK1	841	CASP8	0.955
8737	RIPK1	26133	TRPC4AP	0.962
8737	RIPK1	148022	TICAM1	0.968
8737	RIPK1	7132	TNFRSF1A	0.957
8737	RIPK1	329	BIRC2	0.964
8737	RIPK1	10616	RBCK1	0.974
8737	RIPK1	10133	OPTN	0.983
8737	RIPK1	7335	UBE2V1	0.96
8737	RIPK1	11035	RIPK3	0.98
8737	RIPK1	8795	TNFRSF10B	0.951
8737	RIPK1	23118	TAB2	0.959
8737	RIPK1	9099	USP2	0.974
8737	RIPK1	8772	FADD	0.959
4199	ME1	6904	TBCD	0.951
4199	ME1	7358	UGDH	0.963
55120	FANCL	2176	FANCC	0.962
55120	FANCL	2177	FANCD2	0.95
55120	FANCL	3280	HES1	0.963
23244	PDS5A	23063	WAPAL	0.97
23244	PDS5A	10274	STAG1	0.97
27005	USP21	57787	MARK4	0.959
27005	USP21	23032	USP33	0.968
27005	USP21	2011	MARK2	0.968
27005	USP21	7347	UCHL3	0.956
27005	USP21	10616	RBCK1	0.973
27005	USP21	57159	TRIM54	0.955
27005	USP21	9099	USP2	0.977
9948	WDR1	3336	HSPE1	0.957
9948	WDR1	5763	PTMS	0.955
9948	WDR1	4830	NME1	0.97
56474	CTPS2	10226	PLIN3	0.959
57787	MARK4	2011	MARK2	0.955
57787	MARK4	50855	PARD6A	0.967
140576	S100A16	9217	VAPB	0.97
140576	S100A16	8803	SUCLA2	0.961
5074	PAWR	327	APEH	0.967
5074	PAWR	6470	SHMT1	0.968

5074	PAWR	833	CARS	0.972
5074	PAWR	6643	SNX2	0.966
5074	PAWR	5496	PPM1G	0.959
5074	PAWR	51699	VPS29	0.964
9564	BCAR1	5829	PXN	0.953
9564	BCAR1	3932	LCK	0.974
9564	BCAR1	5782	PTPN12	0.955
9564	BCAR1	4867	NPHP1	0.952
9564	BCAR1	7205	TRIP6	0.951
9564	BCAR1	10525	HYOU1	0.954
9564	BCAR1	23607	CD2AP	0.957
9564	BCAR1	1399	CRKL	0.952
8500	PPFIA1	5802	PTPRS	0.961
8500	PPFIA1	5528	PPP2R5D	0.953
23013	SPEN	862	RUNX1T1	0.966
26524	LATS2	9113	LATS1	0.952
26524	LATS2	55233	MOBKL1B	0.953
26524	LATS2	84962	JUB	0.964
26524	LATS2	6615	SNAI1	0.952
11160	ERLIN2	7353	UFD1L	0.962
11160	ERLIN2	3638	INSIG1	0.969
11160	ERLIN2	79139	DERL1	0.963
11160	ERLIN2	84447	SYVN1	0.953
7188	TRAF5	4591	TRIM37	0.956
7188	TRAF5	63893	UBE2O	0.968
7188	TRAF5	9020	MAP3K14	0.962
7188	TRAF5	4804	NGFR	0.95
7188	TRAF5	148022	TICAM1	0.975
7188	TRAF5	51567	TTRAP	0.965
7188	TRAF5	10293	TRAIP	0.959
56647	BCCIP	8942	KYNU	0.96
56647	BCCIP	55577	NAGK	0.955
79991	OBFC1	9440	MED17	0.95
79991	OBFC1	9442	MED27	0.97
79991	OBFC1	10025	MED16	0.955
79991	OBFC1	10001	MED6	0.959
79991	OBFC1	9439	MED23	0.96
51147	ING4	79960	PHF17	0.964
51147	ING4	64769	MEAF6	0.96
6258	RXRG	84962	JUB	0.96
10533	ATG7	27430	MAT2B	0.953
10533	ATG7	6642	SNX1	0.953
10533	ATG7	64422	ATG3	0.968
10533	ATG7	6904	TBCD	0.956
10534	SSSCA1	10460	TACC3	0.962
10534	SSSCA1	29978	UBQLN2	0.968

10539	GLRX3	3417	IDH1	0.971
10539	GLRX3	79180	EFHD2	0.95
3476	IGBP1	25824	PRDX5	0.954
835	CASP2	637	BID	0.971
835	CASP2	843	CASP10	0.967
835	CASP2	841	CASP8	0.954
835	CASP2	840	CASP7	0.969
8881	CDC16	51592	TRIM33	0.95
8881	CDC16	4085	MAD2L1	0.959
8881	CDC16	26271	FBXO5	0.959
8881	CDC16	10297	APC2	0.952
8883	NAE1	5910	RAP1GDS1	0.966
8883	NAE1	5049	PAFAH1B2	0.964
8883	NAE1	5504	PPP1R2	0.977
80854	SETD7	6879	TAF7	0.973
80854	SETD7	6878	TAF6	0.972
1729	DIAPH1	3831	KLC1	0.967
9459	ARHGEF6	7046	TGFBR1	0.953
9459	ARHGEF6	8874	ARHGEF7	0.972
55339	WDR33	81608	FIP1L1	0.95
6416	MAP2K4	5608	MAP2K6	0.952
6416	MAP2K4	5601	MAPK9	0.966
6416	MAP2K4	5602	MAPK10	0.963
6416	MAP2K4	5609	MAP2K7	0.969
8315	BRAP	3265	HRAS	0.98
8315	BRAP	5604	MAP2K1	0.988
8315	BRAP	7375	USP4	0.952
8315	BRAP	673	BRAF	0.953
8312	AXIN1	1855	DVL1	0.96
6738	TROVE2	7360	UGP2	0.955
8318	CDC45L	23594	ORC6L	0.958
8318	CDC45L	23595	ORC3L	0.972
8318	CDC45L	5001	ORC5L	0.973
8318	CDC45L	55388	MCM10	0.972
9929	JOSD1	57159	TRIM54	0.955
215	ABCD1	5825	ABCD3	0.958
327	APEH	6470	SHMT1	0.952
327	APEH	833	CARS	0.956
327	APEH	22824	HSPA4L	0.956
327	APEH	10059	DNM1L	0.974
3643	INSR	10451	VAV3	0.966
3643	INSR	9021	SOCS3	0.975
3643	INSR	8660	IRS2	0.958
3643	INSR	8651	SOCS1	0.971
2551	GABPA	6670	SP3	0.978
64969	MRPS5	60488	MRPS35	0.951

915	CD3D	79139	DERL1	0.959
64219	PJA1	7347	UCHL3	0.954
7169	TPM2	7170	TPM3	0.952
7163	TPD52	7165	TPD52L2	0.98
7165	TPD52L2	4234	METTL1	0.964
7165	TPD52L2	5202	PFDN2	0.966
7165	TPD52L2	80755	AARSD1	0.975
7165	TPD52L2	51194	IPO11	0.967
7165	TPD52L2	5528	PPP2R5D	0.986
7165	TPD52L2	10096	ACTR3	0.955
7165	TPD52L2	29978	UBQLN2	0.969
7165	TPD52L2	10055	SAE1	0.956
7165	TPD52L2	10552	ARPC1A	0.969
8078	USP5	523	ATP6V1A	0.95
637	BID	5599	MAPK8	0.952
637	BID	598	BCL2L1	0.952
637	BID	355	FAS	0.968
637	BID	836	CASP3	0.956
637	BID	843	CASP10	0.952
637	BID	841	CASP8	0.953
637	BID	7132	TNFRSF1A	0.959
637	BID	4170	MCL1	0.958
637	BID	8795	TNFRSF10B	0.959
637	BID	8772	FADD	0.969
3996	LLGL1	5584	PRKCI	0.957
23032	USP33	9738	CP110	0.959
23032	USP33	84676	TRIM63	0.963
23032	USP33	57159	TRIM54	0.96
23032	USP33	84461	NEURL4	0.953
23644	EDC4	167227	DCP2	0.961
23644	EDC4	4836	NMT1	0.956
23133	PHF8	9070	ASH2L	0.962
5825	ABCD3	5824	PEX19	0.95
5825	ABCD3	53918	PELO	0.956
10452	TOMM40	56993	TOMM22	0.962
10452	TOMM40	51119	SBDS	0.952
196	AHR	2963	GTF2F2	0.958
10458	BAIAP2	2011	MARK2	0.96
10458	BAIAP2	3831	KLC1	0.955
10458	BAIAP2	89953	KLC4	0.966
10458	BAIAP2	5062	PAK2	0.957
10458	BAIAP2	501	ALDH7A1	0.957
10458	BAIAP2	10298	PAK4	0.952
10458	BAIAP2	995	CDC25C	0.976
10101	NUBP2	5721	PSME2	0.959
10101	NUBP2	5202	PFDN2	0.968

10101	NUBP2	7320	UBE2B	0.95
10101	NUBP2	11344	TWF2	0.951
10101	NUBP2	10226	PLIN3	0.954
10101	NUBP2	11328	FKBP9	0.956
10101	NUBP2	55239	OGFOD1	0.958
10101	NUBP2	25824	PRDX5	0.955
10101	NUBP2	134492	NUDCD2	0.955
10101	NUBP2	8539	API5	0.951
10101	NUBP2	54926	UBE2R2	0.95
10101	NUBP2	7511	XPNPEP1	0.958
10101	NUBP2	29978	UBQLN2	0.955
10101	NUBP2	7336	UBE2V2	0.952
10101	NUBP2	9296	ATP6V1F	0.955
10101	NUBP2	11034	DSTN	0.966
10101	NUBP2	10802	SEC24A	0.951
10101	NUBP2	55236	UBA6	0.963
2768	GNA12	2931	GSK3A	0.961
9150	CTDP1	9325	TRIP4	0.96
9150	CTDP1	23476	BRD4	0.95
9736	USP34	29978	UBQLN2	0.958
9736	USP34	10802	SEC24A	0.95
9738	CP110	8924	HERC2	0.958
857	CAV1	355	FAS	0.952
857	CAV1	960	CD44	0.961
84759	PCGF1	23429	RYBP	0.955
5226	PGD	501	ALDH7A1	0.965
8900	CCNA1	51343	FZR1	0.953
54732	TMED9	9554	SEC22B	0.969
3069	HDLBP	51028	VPS36	0.962
26173	INTS1	5437	POLR2H	0.968
26173	INTS1	23613	ZMYND8	0.977
26173	INTS1	54973	CPSF3L	0.964
26173	INTS1	5519	PPP2R1B	0.95
26173	INTS1	55756	INTS9	0.955
6478	SIAH2	6497	SKI	0.971
6472	SHMT2	6642	SNX1	0.952
6470	SHMT1	833	CARS	0.965
6470	SHMT1	3035	HARS	0.955
6470	SHMT1	7265	TTC1	0.953
6470	SHMT1	51699	VPS29	0.966
6477	SIAH1	51132	RLIM	0.955
3665	IRF7	7128	TNFAIP3	0.954
3665	IRF7	8772	FADD	0.952
3667	IRS1	5336	PLCG2	0.953
3667	IRS1	5290	PIK3CA	0.969
3663	IRF5	2873	GPS1	0.957

150684	COMMD1	5971	RELB	0.961
4437	MSH3	5883	RAD9A	0.984
4437	MSH3	5810	RAD1	0.98
4437	MSH3	5395	PMS2	0.986
4437	MSH3	84464	BTBD12	0.967
90390	MED30	5435	POLR2F	0.955
8085	MLL2	80335	WDR82	0.958
8085	MLL2	7403	KDM6A	0.952
8085	MLL2	9070	ASH2L	0.963
8924	HERC2	7337	UBE3A	0.953
57082	CASC5	10403	NDC80	0.954
4236	MFAP1	79753	SNIP1	0.971
4234	METTL1	55666	NPLOC4	0.962
4234	METTL1	1810	DR1	0.961
4234	METTL1	51194	IPO11	0.966
4233	MET	23624	CBLC	0.95
4233	MET	6654	SOS1	0.976
5590	PRKCZ	50855	PARD6A	0.953
317	APAF1	842	CASP9	0.979
11200	CHEK2	5527	PPP2R5C	0.95
65008	MRPL1	51073	MRPL4	0.957
65003	MRPL11	64978	MRPL38	0.963
65005	MRPL9	11222	MRPL3	0.963
65005	MRPL9	9801	MRPL19	0.953
65005	MRPL9	51069	MRPL2	0.985
27258	LSM3	25804	LSM4	0.95
27258	LSM3	11157	LSM6	0.966
8697	CDC23	56916	SMARCAD1	0.95
8697	CDC23	51592	TRIM33	0.95
8697	CDC23	11065	UBE2C	0.955
8697	CDC23	26271	FBXO5	0.968
10914	PAPOLA	10726	NUDC	0.96
10919	EHMT2	1788	DNMT3A	0.973
7703	PCGF2	1912	PHC2	0.956
7703	PCGF2	23429	RYBP	0.967
7703	PCGF2	10138	YAF2	0.967
26127	FGFR1OP2	85369	FAM40A	0.954
26127	FGFR1OP2	54764	ZRANB1	0.966
81855	SFXN3	94081	SFXN1	0.981
81857	MED25	7067	THRA	0.953
81857	MED25	80306	MED28	0.95
81858	SHARPIN	7124	TNF	0.954
81858	SHARPIN	64127	NOD2	0.953
81858	SHARPIN	10616	RBCK1	0.953
26128	KIAA1279	6455	SH3GL1	0.956
4591	TRIM37	4804	NGFR	0.953

4591	TRIM37	9513	FXR2	0.957
4846	NOS3	2639	GCDH	0.952
5201	PFDN1	5202	PFDN2	0.964
5201	PFDN1	3035	HARS	0.952
5203	PFDN4	5202	PFDN2	0.96
5203	PFDN4	7411	VBP1	0.963
5202	PFDN2	5204	PFDN5	0.965
5202	PFDN2	5516	PPP2CB	0.958
5202	PFDN2	29978	UBQLN2	0.978
5202	PFDN2	7411	VBP1	0.968
5204	PFDN5	4659	PPP1R12A	0.953
5204	PFDN5	7411	VBP1	0.957
6455	SH3GL1	573	BAG1	0.955
6455	SH3GL1	2960	GTF2E1	0.959
6455	SH3GL1	56904	SH3GLB2	0.973
91833	WDR20	23239	PHLPP1	0.962
91833	WDR20	64854	USP46	0.95
55090	MED9	5439	POLR2J	0.96
3956	LGALS1	8624	PSMG1	0.963
8353	HIST1H3E	5252	PHF1	0.97
8353	HIST1H3E	8208	CHAF1B	0.97
8353	HIST1H3E	55723	ASF1B	0.959
5782	PTPN12	7360	UGP2	0.962
116985	ARAP1	3635	INPP5D	0.971
85369	FAM40A	54764	ZRANB1	0.962
85363	TRIM5	4331	MNAT1	0.957
85363	TRIM5	7681	MKRN3	0.95
85363	TRIM5	7336	UBE2V2	0.975
85363	TRIM5	7335	UBE2V1	0.95
85363	TRIM5	22954	TRIM32	0.961
85363	TRIM5	9099	USP2	0.963
6338	SCNN1B	10053	AP1M2	0.961
55666	NPLOC4	10611	PDLIM5	0.954
55666	NPLOC4	10226	PLIN3	0.953
55666	NPLOC4	55197	RPRD1A	0.957
55666	NPLOC4	10785	WDR4	0.952
10451	VAV3	5159	PDGFRB	0.962
23049	SMG1	2935	GSPT1	0.965
152006	RNF38	9666	DZIP3	0.953
152006	RNF38	55905	RNF114	0.954
7124	TNF	355	FAS	0.955
7124	TNF	55072	RNF31	0.966
7124	TNF	7133	TNFRSF1B	0.976
7124	TNF	10616	RBCK1	0.955
7124	TNF	10133	OPTN	0.952
7124	TNF	10318	TNIP1	0.972

598	BCL2L1	578	BAK1	0.954
598	BCL2L1	7159	TP53BP2	0.963
4215	MAP3K3	2011	MARK2	0.952
4216	MAP3K4	9618	TRAF4	0.957
4216	MAP3K4	5609	MAP2K7	0.957
4218	RAB8A	6342	SCP2	0.968
4218	RAB8A	9230	RAB11B	0.965
171568	POLR3H	51082	POLR1D	0.95
1540	CYLD	8975	USP13	0.951
5573	PRKAR1A	11215	AKAP11	0.952
3265	HRAS	5604	MAP2K1	0.951
3265	HRAS	9610	RIN1	0.955
3265	HRAS	5290	PIK3CA	0.966
79590	MRPL24	51069	MRPL2	0.952
79590	MRPL24	84311	MRPL45	0.966
79590	MRPL24	64976	MRPL40	0.961
79590	MRPL24	51253	MRPL37	0.953
79590	MRPL24	51073	MRPL4	0.955
331	XIAP	332	BIRC5	0.954
330	BIRC3	332	BIRC5	0.968
330	BIRC3	55072	RNF31	0.955
330	BIRC3	842	CASP9	0.954
330	BIRC3	9666	DZIP3	0.961
330	BIRC3	7132	TNFRSF1A	0.953
330	BIRC3	27429	HTRA2	0.95
332	BIRC5	842	CASP9	0.985
332	BIRC5	56616	DIABLO	0.952
332	BIRC5	3619	INCENP	0.972
332	BIRC5	55143	CDCA8	0.981
11222	MRPL3	28977	MRPL42	0.966
11222	MRPL3	51073	MRPL4	0.965
11222	MRPL3	124995	MRPL10	0.958
51343	FZR1	9232	PTTG1	0.956
51433	ANAPC5	56916	SMARCAD1	0.952
51433	ANAPC5	51592	TRIM33	0.954
51433	ANAPC5	11065	UBE2C	0.956
51433	ANAPC5	26271	FBXO5	0.95
10938	EHD1	8539	API5	0.955
9443	MED7	6720	SREBF1	0.96
9443	MED7	80306	MED28	0.976
9443	MED7	6837	MED22	0.959
9440	MED17	9325	TRIP4	0.954
8882	ZNF259	7336	UBE2V2	0.958
8882	ZNF259	51569	UFM1	0.951
81876	RAB1B	9230	RAB11B	0.951
2011	MARK2	4140	MARK3	0.956

2011	MARK2	50855	PARD6A	0.982
2011	MARK2	51564	HDAC7	0.969
10592	SMC2	9918	NCAPD2	0.957
10592	SMC2	64151	NCAPG	0.955
54700	RRN3	84172	POLR1B	0.96
27430	MAT2B	6642	SNX1	0.963
7015	TERT	10445	MCRS1	0.95
9113	LATS1	11186	RASSF1	0.966
9112	MTA1	4331	MNAT1	0.953
9442	MED27	9325	TRIP4	0.955
55636	CHD7	55193	PBRM1	0.955
6925	TCF4	6886	TAL1	0.969
6925	TCF4	3397	ID1	0.953
6925	TCF4	3399	ID3	0.955
28957	MRPS28	28977	MRPL42	0.954
28957	MRPS28	64975	MRPL41	0.951
28957	MRPS28	4701	NDUFA7	0.953
4005	LMO2	6886	TAL1	0.96
3417	IDH1	2805	GOT1	0.953
3417	IDH1	79180	EFHD2	0.951
3417	IDH1	3336	HSPE1	0.954
3417	IDH1	6888	TALDO1	0.956
3417	IDH1	5763	PTMS	0.973
3417	IDH1	4830	NME1	0.965
578	BAK1	581	BAX	0.974
578	BAK1	4170	MCL1	0.952
29945	ANAPC4	4085	MAD2L1	0.956
29945	ANAPC4	26271	FBXO5	0.953
571	BACH1	11073	TOPBP1	0.97
571	BACH1	7975	MAFK	0.95
571	BACH1	4097	MAFG	0.968
571	BACH1	675	BRCA2	0.971
2801	GOLGA2	64689	GORASP1	0.959
5436	POLR2G	9400	RECQL5	0.97
5436	POLR2G	2963	GTF2F2	0.959
5436	POLR2G	26512	INTS6	0.953
5436	POLR2G	80306	MED28	0.97
5436	POLR2G	65123	INTS3	0.952
5437	POLR2H	661	POLR3D	0.957
5437	POLR2H	2963	GTF2F2	0.962
5437	POLR2H	25885	POLR1A	0.977
5437	POLR2H	2959	GTF2B	0.959
5437	POLR2H	1788	DNMT3A	0.975
5434	POLR2E	2963	GTF2F2	0.951
5434	POLR2E	2959	GTF2B	0.95
5439	POLR2J	80306	MED28	0.959

58477	SRPRB	10084	PQBP1	0.955
58477	SRPRB	5937	RBMS1	0.964
58477	SRPRB	51119	SBDS	0.957
58477	SRPRB	9997	SCO2	0.955
197131	UBR1	523	ATP6V1A	0.953
5883	RAD9A	5884	RAD17	0.967
5883	RAD9A	11073	TOPBP1	0.97
5883	RAD9A	7266	DNAJC7	0.95
5883	RAD9A	63967	CLSPN	0.97
23186	RCOR1	6886	TAL1	0.958
23186	RCOR1	5978	REST	0.985
23186	RCOR1	57504	MTA3	0.959
23186	RCOR1	6615	SNAI1	0.973
23633	KPNA6	3839	KPNA3	0.956
25929	GEMIN5	6726	SRP9	0.95
355	FAS	8837	CFLAR	0.965
355	FAS	843	CASP10	0.96
355	FAS	7132	TNFRSF1A	0.954
355	FAS	8772	FADD	0.959
11073	TOPBP1	5395	PMS2	0.974
7508	XPC	2965	GTF2H1	0.957
10952	SEC61B	439	ASNA1	0.971
10952	SEC61B	29927	SEC61A1	0.961
10952	SEC61B	6449	SGTA	0.95
10956	OS9	51465	UBE2J1	0.963
4800	NFYA	4801	NFYB	0.972
4800	NFYA	22926	ATF6	0.962
4802	NFYC	6878	TAF6	0.952
2072	ERCC4	2067	ERCC1	0.955
2072	ERCC4	84464	BTBD12	0.966
8975	USP13	7353	UFD1L	0.951
84661	DPY30	9070	ASH2L	0.95
6497	SKI	4204	MECP2	0.961
51013	EXOSC1	118460	EXOSC6	0.952
79657	RPAP3	50855	PARD6A	0.955
28977	MRPL42	51253	MRPL37	0.96
28973	MRPS18B	60488	MRPS35	0.952
4026	LPP	51002	TPRKB	0.968
4026	LPP	3267	AGFG1	0.95
4026	LPP	51194	IPO11	0.951
4026	LPP	5528	PPP2R5D	0.967
4026	LPP	10055	SAE1	0.956
55763	EXOC1	667	DST	0.958
55763	EXOC1	60412	EXOC4	0.959
55763	EXOC1	284058	KIAA1267	0.95
51069	MRPL2	64976	MRPL40	0.956

51069	MRPL2	51253	MRPL37	0.96
51069	MRPL2	6150	MRPL23	0.961
51608	C7orf20	1478	CSTF2	0.97
55588	MED29	2963	GTF2F2	0.952
55588	MED29	80306	MED28	0.951
57187	THOC2	55854	ZC3H15	0.962
9328	GTF3C5	9329	GTF3C4	0.96
9328	GTF3C5	10623	POLR3C	0.981
9328	GTF3C5	9330	GTF3C3	0.963
9328	GTF3C5	2976	GTF3C2	0.968
9329	GTF3C4	10623	POLR3C	0.962
9329	GTF3C4	9330	GTF3C3	0.965
9329	GTF3C4	3098	HK1	0.954
9329	GTF3C4	7511	XPNPEP1	0.972
9329	GTF3C4	30844	EHD4	0.963
9329	GTF3C4	2976	GTF3C2	0.956
26973	CHORDC1	55577	NAGK	0.954
550	AUP1	6400	SEL1L	0.952
550	AUP1	1650	DDOST	0.979
550	AUP1	51465	UBE2J1	0.976
550	AUP1	84447	SYVN1	0.962
79003	MIS12	10403	NDC80	0.956
5530	PPP3CA	4898	NRD1	0.96
5530	PPP3CA	64175	LEPRE1	0.958
5530	PPP3CA	7411	VBP1	0.951
5537	PPP6C	23243	ANKRD28	0.968
5536	PPP5C	5910	RAP1GDS1	0.952
5536	PPP5C	5049	PAFAH1B2	0.966
5536	PPP5C	64785	GINS3	0.964
5536	PPP5C	1676	DFFA	0.958
5536	PPP5C	2937	GSS	0.966
23613	ZMYND8	65123	INTS3	0.954
51479	ANKFY1	6093	ROCK1	0.968
3831	KLC1	89953	KLC4	0.962
3831	KLC1	372	ARCN1	0.95
3831	KLC1	7090	TLE3	0.952
10979	FERMT2	1676	DFFA	0.968
10979	FERMT2	501	ALDH7A1	0.967
56916	SMARCAD1	55929	DMAP1	0.952
2057	EPOR	8660	IRS2	0.952
2057	EPOR	3635	INPP5D	0.957
2057	EPOR	1399	CRKL	0.954
9402	GRAP2	11184	MAP4K1	0.954
9400	RECQL5	5433	POLR2D	0.952
10621	POLR3F	10623	POLR3C	0.977
7325	UBE2E2	7681	MKRN3	0.956

7320	UBE2B	9296	ATP6V1F	0.95
7320	UBE2B	10802	SEC24A	0.953
10084	PQBP1	5937	RBMS1	0.959
10084	PQBP1	7419	VDAC3	0.953
10084	PQBP1	9997	SCO2	0.958
10084	PQBP1	9877	ZC3H11A	0.964
10084	PQBP1	4701	NDUFA7	0.961
7067	THRA	10025	MED16	0.951
7067	THRA	2874	GPS2	0.973
7064	THOP1	309	ANXA6	0.951
7064	THOP1	1633	DCK	0.972
8624	PSMG1	84262	PSMG3	0.959
9984	THOC1	84321	THOC3	0.951
51592	TRIM33	64682	ANAPC1	0.95
9616	RNF7	51619	UBE2D4	0.951
9616	RNF7	7681	MKRN3	0.977
9616	RNF7	997	CDC34	0.96
9341	VAMP3	8673	VAMP8	0.968
9611	NCOR1	2959	GTF2B	0.952
9611	NCOR1	6878	TAF6	0.95
9618	TRAF4	7041	TGFB1I1	0.97
9618	TRAF4	8767	RIPK2	0.965
1810	DR1	51194	IPO11	0.958
1810	DR1	51562	MBIP	0.96
7290	HIRA	23523	CABIN1	0.973
26958	COPG2	11316	COPE	0.956
23075	SWAP70	10606	PAICS	0.959
23075	SWAP70	5007	OSBP	0.95
23075	SWAP70	5527	PPP2R5C	0.965
1601	DAB2	160	AP2A1	0.962
5479	PPIB	5937	RBMS1	0.986
5479	PPIB	57794	SF4	0.982
5479	PPIB	10469	TIMM44	0.979
5479	PPIB	9877	ZC3H11A	0.976
9894	TELO2	80230	RUFY1	0.959
9894	TELO2	55011	PIH1D1	0.953
55705	IPO9	23534	TNPO3	0.958
51082	POLR1D	84172	POLR1B	0.961
51082	POLR1D	25885	POLR1A	0.956
5519	PPP2R1B	11235	PDCD10	0.955
5519	PPP2R1B	5528	PPP2R5D	0.976
5511	PPP1R8	8726	EED	0.963
5516	PPP2CB	5528	PPP2R5D	0.968
3035	HARS	60528	ELAC2	0.957
3035	HARS	57510	XPO5	0.952
3035	HARS	7265	TTC1	0.953

3035	HARS	64746	ACBD3	0.951
3815	KIT	7006	TEC	0.964
3815	KIT	868	CBLB	0.965
9425	CDYL	5978	REST	0.965
5289	PIK3C3	30849	PIK3R4	0.974
5289	PIK3C3	8678	BECN1	0.959
9021	SOCS3	7852	CXCR4	0.961
9282	MED14	23476	BRD4	0.964
9282	MED14	9444	QKI	0.956
7046	TGFBR1	960	CD44	0.954
23708	GSPT2	5962	RDX	0.969
23708	GSPT2	2935	GSPT1	0.958
11344	TWF2	55239	OGFOD1	0.97
11344	TWF2	824	CAPN2	0.982
11344	TWF2	830	CAPZA2	0.953
11344	TWF2	3098	HK1	0.965
11344	TWF2	440	ASNS	0.962
11344	TWF2	9601	PDIA4	0.966
11345	GABARAPL2	64422	ATG3	0.955
11345	GABARAPL2	23192	ATG4B	0.957
11345	GABARAPL2	8408	ULK1	0.958
51024	FIS1	10059	DNM1L	0.951
64127	NOD2	55072	RNF31	0.973
64127	NOD2	329	BIRC2	0.96
64127	NOD2	10616	RBCK1	0.977
9361	LONP1	22983	MAST1	0.951
9361	LONP1	24138	IFIT5	0.957
1831	TSC22D3	6446	SGK1	0.961
10611	PDLIM5	51194	IPO11	0.952
57037	ANKMY2	10226	PLIN3	0.965
57037	ANKMY2	55197	RPRD1A	0.954
10614	HEXIM1	23476	BRD4	0.979
10614	HEXIM1	904	CCNT1	0.97
29924	EPN1	160	AP2A1	0.972
29924	EPN1	30846	EHD2	0.953
29924	EPN1	10053	AP1M2	0.95
23658	LSM5	11157	LSM6	0.968
8473	OGT	23640	HSPBP1	0.95
8473	OGT	10785	WDR4	0.953
55201	MAP1S	83593	RASSF5	0.953
2237	FEN1	60528	ELAC2	0.958
5937	RBMS1	8417	STX7	0.979
5937	RBMS1	51119	SBDS	0.963
5937	RBMS1	8724	SNX3	0.963
5937	RBMS1	10469	TIMM44	0.97
5937	RBMS1	6342	SCP2	0.979

5937	RBMS1	9877	ZC3H11A	0.972
8125	ANP32A	10226	PLIN3	0.963
23595	ORC3L	5001	ORC5L	0.967
23595	ORC3L	55388	MCM10	0.966
7360	UGP2	7791	ZYX	0.954
7360	UGP2	11252	PACSIN2	0.971
7360	UGP2	10726	NUDC	0.962
7027	TFDP1	1876	E2F6	0.961
7027	TFDP1	23429	RYBP	0.963
10226	PLIN3	55197	RPRD1A	0.968
10226	PLIN3	6726	SRP9	0.957
10226	PLIN3	51194	IPO11	0.969
10226	PLIN3	10785	WDR4	0.963
7791	ZYX	26136	TES	0.954
79184	BRCC3	9043	SPAG9	0.955
79184	BRCC3	675	BRCA2	0.955
8660	IRS2	5290	PIK3CA	0.954
8660	IRS2	8651	SOCS1	0.951
11328	FKBP9	8539	API5	0.962
11328	FKBP9	11235	PDCD10	0.956
11328	FKBP9	8994	LIMD1	0.952
11328	FKBP9	55236	UBA6	0.958
11328	FKBP9	55148	UBR7	0.952
55072	RNF31	8767	RIPK2	0.967
55072	RNF31	7706	TRIM25	0.969
55072	RNF31	10616	RBCK1	0.952
51009	DERL2	51465	UBE2J1	0.961
51009	DERL2	84447	SYVN1	0.956
51002	TPRKB	3267	AGFG1	0.962
51002	TPRKB	51194	IPO11	0.953
51002	TPRKB	10092	ARPC5	0.961
8089	YEATS4	3015	H2AFZ	0.961
8089	YEATS4	54556	ING3	0.95
8089	YEATS4	55929	DMAP1	0.967
6642	SNX1	7358	UGDH	0.956
6643	SNX2	5962	RDX	0.954
6643	SNX2	2935	GSPT1	0.952
6643	SNX2	5159	PDGFRB	0.956
57697	FANCM	2176	FANCC	0.95
1855	DVL1	1857	DVL3	0.953
1855	DVL1	1453	CSNK1D	0.95
2176	FANCC	3280	HES1	0.951
5433	POLR2D	80306	MED28	0.957
5433	POLR2D	65123	INTS3	0.951
84296	GINS4	64785	GINS3	0.956
3267	AGFG1	51056	LAP3	0.956

3267	AGFG1	64175	LEPRE1	0.955
6886	TAL1	861	RUNX1	0.955
6886	TAL1	6304	SATB1	0.953
6884	TAF13	51616	TAF9B	0.961
6884	TAF13	6878	TAF6	0.969
6884	TAF13	6872	TAF1	0.972
6888	TALDO1	1633	DCK	0.959
602	BCL3	5971	RELB	0.954
468	ATF4	2959	GTF2B	0.954
468	ATF4	3726	JUNB	0.955
8417	STX7	51119	SBDS	0.968
8417	STX7	57794	SF4	0.963
8417	STX7	8724	SNX3	0.972
8417	STX7	4905	NSF	0.98
8417	STX7	55573	CDV3	0.955
8417	STX7	9997	SCO2	0.979
8417	STX7	6342	SCP2	0.967
8417	STX7	8673	VAMP8	0.975
8417	STX7	4701	NDUFA7	0.969
4140	MARK3	51564	HDAC7	0.96
4140	MARK3	995	CDC25C	0.975
55239	OGFOD1	55236	UBA6	0.96
5910	RAP1GDS1	1892	ECHS1	0.959
5910	RAP1GDS1	5049	PAFAH1B2	0.958
5910	RAP1GDS1	5504	PPP1R2	0.957
5910	RAP1GDS1	5198	PFAS	0.952
5910	RAP1GDS1	29789	OLA1	0.963
64682	ANAPC1	9232	PTTG1	0.959
57599	WDR48	23239	PHLPP1	0.958
64425	POLR1E	25885	POLR1A	0.95
64422	ATG3	9140	ATG12	0.97
64422	ATG3	445	ASS1	0.969
64422	ATG3	9474	ATG5	0.965
64422	ATG3	23192	ATG4B	0.96
2617	GARS	7358	UGDH	0.965
7347	UCHL3	7335	UBE2V1	0.95
7347	UCHL3	9296	ATP6V1F	0.954
7347	UCHL3	55236	UBA6	0.959
7347	UCHL3	57159	TRIM54	0.959
29082	CHMP4A	1633	DCK	0.953
29082	CHMP4A	27183	VPS4A	0.951
9020	MAP3K14	8837	CFLAR	0.966
9020	MAP3K14	4791	NFKB2	0.969
9020	MAP3K14	843	CASP10	0.967
29882	ANAPC2	27338	UBE2S	0.956
29883	CNOT7	8317	CDC7	0.957

29888	STRN4	29115	SAP30BP	0.95
29888	STRN4	5527	PPP2R5C	0.957
23637	RABGAP1	7879	RAB7A	0.951
1052	CEBPD	51176	LEF1	0.966
1052	CEBPD	2623	GATA1	0.953
1052	CEBPD	3726	JUNB	0.954
1054	CEBPG	8061	FOSL1	0.966
1054	CEBPG	3726	JUNB	0.973
5358	PLS3	5763	PTMS	0.957
5358	PLS3	4830	NME1	0.958
5358	PLS3	1633	DCK	0.96
11047	ADRM1	10422	UBAC1	0.956
54108	CHRAC1	5763	PTMS	0.968
8648	NCOA1	9325	TRIP4	0.964
8648	NCOA1	2959	GTF2B	0.96
8649	MAPKSP1	56993	TOMM22	0.962
8649	MAPKSP1	5604	MAP2K1	0.975
1876	E2F6	23429	RYBP	0.976
1874	E2F4	9070	ASH2L	0.956
10499	NCOA2	8856	NR1I2	0.955
57819	LSM2	8449	DHX16	0.955
10018	BCL2L11	4170	MCL1	0.959
661	POLR3D	10623	POLR3C	0.955
9821	RB1CC1	8408	ULK1	0.957
23168	RTF1	6917	TCEA1	0.972
23168	RTF1	1788	DNMT3A	0.966
23168	RTF1	80349	WDR61	0.982
5978	REST	23309	SIN3B	0.953
5971	RELB	5977	DPF2	0.954
10951	CBX1	9557	CHD1L	0.971
10951	CBX1	4204	MECP2	0.961
10951	CBX1	1788	DNMT3A	0.979
84171	LOXL4	10053	AP1M2	0.955
84172	POLR1B	25885	POLR1A	0.98
84311	MRPL45	64978	MRPL38	0.95
84311	MRPL45	51253	MRPL37	0.961
84313	VPS25	51028	VPS36	0.972
2965	GTF2H1	4331	MNAT1	0.962
2965	GTF2H1	6917	TCEA1	0.958
2965	GTF2H1	2068	ERCC2	0.951
2961	GTF2E2	2963	GTF2F2	0.962
2960	GTF2E1	6917	TCEA1	0.95
2960	GTF2E1	6301	SARS	0.952
2960	GTF2E1	56904	SH3GLB2	0.953
2963	GTF2F2	6917	TCEA1	0.96
2962	GTF2F1	6872	TAF1	0.956

4331	MNAT1	2071	ERCC3	0.957
4331	MNAT1	56658	TRIM39	0.956
4331	MNAT1	7681	MKRN3	0.953
4331	MNAT1	9099	USP2	0.971
5763	PTMS	1633	DCK	0.971
5184	PEPD	8942	KYNU	0.973
5184	PEPD	445	ASS1	0.959
5184	PEPD	840	CASP7	0.971
5184	PEPD	9615	GDA	0.964
5184	PEPD	5476	CTSA	0.972
5184	PEPD	10072	DPP3	0.963
5184	PEPD	2936	GSR	0.974
6602	SMARCD1	6603	SMARCD2	0.968
6602	SMARCD1	29117	BRD7	0.952
6602	SMARCD1	2623	GATA1	0.974
6603	SMARCD2	9031	BAZ1B	0.959
1892	ECHS1	64785	GINS3	0.954
1892	ECHS1	5198	PFAS	0.951
1892	ECHS1	2937	GSS	0.972
4928	NUP98	8480	RAE1	0.963
5162	PDHB	1737	DLAT	0.976
80335	WDR82	9070	ASH2L	0.966
80335	WDR82	11091	WDR5	0.954
84433	CARD11	5588	PRKCQ	0.968
84433	CARD11	9099	USP2	0.976
6844	VAMP2	9554	SEC22B	0.962
6844	VAMP2	8673	VAMP8	0.954
6844	VAMP2	9217	VAPB	0.966
6844	VAMP2	8773	SNAP23	0.957
648	BMI1	10336	PCGF3	0.951
9646	CTR9	6917	TCEA1	0.952
9646	CTR9	80349	WDR61	0.963
10746	MAP3K2	5609	MAP2K7	0.958
79101	TAF1D	25885	POLR1A	0.96
4659	PPP1R12A	64175	LEPRE1	0.954
51119	SBDS	8724	SNX3	0.961
51119	SBDS	10469	TIMM44	0.954
51119	SBDS	9997	SCO2	0.956
51119	SBDS	10277	UBE4B	0.959
8837	CFLAR	8767	RIPK2	0.957
8837	CFLAR	8772	FADD	0.956
1759	DNM1	11252	PACSIN2	0.952
8767	RIPK2	7132	TNFRSF1A	0.958
8767	RIPK2	23118	TAB2	0.956
8766	RAB11A	7879	RAB7A	0.962
8766	RAB11A	9230	RAB11B	0.954

8766	RAB11A	2665	GDI2	0.952
55752	11-Sep	10801	9-Sep	0.951
80755	AARSD1	6119	RPA3	0.96
80755	AARSD1	56904	SH3GLB2	0.965
8539	API5	7511	XPNPEP1	0.973
8027	STAM	9146	HGS	0.957
8027	STAM	9101	USP8	0.953
8027	STAM	407	ARR3	0.953
8027	STAM	29978	UBQLN2	0.954
8027	STAM	11075	STMN2	0.955
51135	IRAK4	114609	TIRAP	0.957
162	AP1B1	9685	CLINT1	0.967
163	AP2B1	9685	CLINT1	0.951
10363	HMG20A	57504	MTA3	0.963
57504	MTA3	23199	KIAA0182	0.981
57504	MTA3	57459	GATAD2B	0.981
64746	ACBD3	6301	SARS	0.955
64746	ACBD3	11267	SNF8	0.965
64746	ACBD3	51028	VPS36	0.953
64746	ACBD3	26003	GORASP2	0.958
5605	MAP2K2	5604	MAP2K1	0.965
5605	MAP2K2	673	BRAF	0.961
5604	MAP2K1	369	ARAF	0.972
5604	MAP2K1	673	BRAF	0.959
5604	MAP2K1	65125	WNK1	0.975
5601	MAPK9	5609	MAP2K7	0.98
9632	SEC24C	10802	SEC24A	0.954
9325	TRIP4	29079	MED4	0.962
9325	TRIP4	9439	MED23	0.973
9325	TRIP4	51586	MED15	0.968
9325	TRIP4	80306	MED28	0.96
8942	KYNU	55577	NAGK	0.984
8942	KYNU	10944	C11orf58	0.982
8942	KYNU	6611	SMS	0.99
8942	KYNU	2936	GSR	0.987
23256	SCFD1	64976	MRPL40	0.966
23256	SCFD1	4905	NSF	0.966
23256	SCFD1	8773	SNAP23	0.972
57794	SF4	8724	SNX3	0.95
57794	SF4	9877	ZC3H11A	0.962
5049	PAFAH1B2	2937	GSS	0.955
89953	KLC4	64837	KLC2	0.951
23062	GGA2	3482	IGF2R	0.952
23063	WAPAL	10274	STAG1	0.972
29966	STRN3	5524	PPP2R4	0.956
55658	RNF126	373	TRIM23	0.96

56658	TRIM39	9099	USP2	0.956
4613	MYCN	4914	NTRK1	0.955
10523	CHERP	1659	DHX8	0.956
824	CAPN2	3098	HK1	0.975
824	CAPN2	9601	PDIA4	0.955
826	CAPNS1	4643	MYO1E	0.955
5252	PHF1	8726	EED	0.963
5252	PHF1	3090	HIC1	0.958
11127	KIF3A	22920	KIFAP3	0.959
3015	H2AFZ	55929	DMAP1	0.951
8724	SNX3	9877	ZC3H11A	0.963
8726	EED	3090	HIC1	0.969
50855	PARD6A	5584	PRKCI	0.961
50855	PARD6A	56288	PARD3	0.954
50855	PARD6A	84612	PARD6B	0.965
5660	PSAP	6836	SURF4	0.956
23239	PHLPP1	64854	USP46	0.95
6400	SEL1L	51465	UBE2J1	0.961
6400	SEL1L	79139	DERL1	0.978
6400	SEL1L	84447	SYVN1	0.965
836	CASP3	843	CASP10	0.957
8887	TAX1BP1	4646	MYO6	0.958
8887	TAX1BP1	10318	TNIP1	0.982
64975	MRPL41	51253	MRPL37	0.957
64975	MRPL41	124995	MRPL10	0.967
5062	PAK2	54926	UBE2R2	0.962
9577	BRE	675	BRCA2	0.952
79718	TBL1XR1	2874	GPS2	0.965
30001	ERO1L	9601	PDIA4	0.957
30001	ERO1L	23640	HSPBP1	0.954
30001	ERO1L	10785	WDR4	0.955
30001	ERO1L	64175	LEPRE1	0.956
8061	FOSL1	3726	JUNB	0.979
1453	CSNK1D	2273	FHL1	0.959
1453	CSNK1D	94274	PPP1R14A	0.962
4798	NFRKB	10445	MCRS1	0.952
6904	TBCD	6301	SARS	0.965
6904	TBCD	55759	WDR12	0.959
6904	TBCD	51699	VPS29	0.969
6904	TBCD	26003	GORASP2	0.971
64785	GINS3	2937	GSS	0.965
64786	TBC1D15	10059	DNM1L	0.973
51176	LEF1	4092	SMAD7	0.957
10320	IKZF1	1107	CHD3	0.951
10320	IKZF1	2623	GATA1	0.975
10320	IKZF1	23309	SIN3B	0.957

7979	SHFM1	55756	INTS9	0.968
7979	SHFM1	26512	INTS6	0.974
7979	SHFM1	675	BRCA2	0.97
9140	ATG12	9474	ATG5	0.971
9140	ATG12	23192	ATG4B	0.964
9493	KIF23	9101	USP8	0.976
9146	HGS	9372	ZFYVE9	0.961
9146	HGS	10254	STAM2	0.956
373	TRIM23	9099	USP2	0.966
372	ARCN1	11316	COPE	0.951
843	CASP10	842	CASP9	0.96
843	CASP10	840	CASP7	0.979
843	CASP10	8797	TNFRSF10A	0.965
842	CASP9	841	CASP8	0.959
842	CASP9	581	BAX	0.974
842	CASP9	329	BIRC2	0.956
841	CASP8	840	CASP7	0.967
841	CASP8	10134	BCAP31	0.955
4905	NSF	8773	SNAP23	0.962
4905	NSF	8775	NAPA	0.972
6464	SHC1	868	CBLB	0.952
26092	TOR1AIP1	55183	RIF1	0.979
114609	TIRAP	7099	TLR4	0.967
5001	ORC5L	5000	ORC4L	0.959
5001	ORC5L	8317	CDC7	0.959
5000	ORC4L	55388	MCM10	0.974
904	CCNT1	123169	LEO1	0.954
904	CCNT1	6879	TAF7	0.962
904	CCNT1	6872	TAF1	0.966
9559	VPS26A	9296	ATP6V1F	0.95
9559	VPS26A	51028	VPS36	0.957
9559	VPS26A	10059	DNM1L	0.967
9559	VPS26A	10726	NUDC	0.959
9554	SEC22B	9217	VAPB	0.982
64223	MLST8	253260	RICTOR	0.95
84993	UBL7	283237	TTC9C	0.955
84993	UBL7	29978	UBQLN2	0.956
5584	PRKCI	84612	PARD6B	0.952
5588	PRKCQ	5170	PDPK1	0.973
64769	MEAF6	7994	MYST3	0.952
8317	CDC7	55388	MCM10	0.956
868	CBLB	23607	CD2AP	0.964
868	CBLB	7535	ZAP70	0.97
868	CBLB	1399	CRKL	0.97
868	CBLB	25	ABL1	0.96
868	CBLB	7410	VAV2	0.969

868	CBLB	6850	SYK	0.966
10623	POLR3C	9330	GTF3C3	0.959
10623	POLR3C	2976	GTF3C2	0.968
861	RUNX1	865	CBFB	0.959
861	RUNX1	7994	MYST3	0.953
864	RUNX3	4090	SMAD5	0.951
9330	GTF3C3	29894	CPSF1	0.971
9444	QKI	51586	MED15	0.954
3054	HCFC1	9070	ASH2L	0.95
4597	MVD	2936	GSR	0.956
81611	ANP32E	5962	RDX	0.953
6446	SGK1	5598	MAPK7	0.959
6917	TCEA1	123169	LEO1	0.955
6917	TCEA1	2959	GTF2B	0.962
6917	TCEA1	80349	WDR61	0.969
92483	LDHAL6B	55577	NAGK	0.95
60412	EXOC4	149371	EXOC8	0.958
960	CD44	7410	VAV2	0.977
3098	HK1	11235	PDCD10	0.962
1612	DAPK1	8772	FADD	0.954
1676	DFFA	5198	PFAS	0.959
64965	MRPS9	51021	MRPS16	0.95
4204	MECP2	6670	SP3	0.955
148022	TICAM1	7099	TLR4	0.961
148022	TICAM1	54476	RNF216	0.96
23404	EXOSC2	57379	AICDA	0.968
6118	RPA2	29978	UBQLN2	0.969
6119	RPA3	60491	NIF3L1	0.95
9101	USP8	10254	STAM2	0.953
9101	USP8	57159	TRIM54	0.956
136319	MTPN	523	ATP6V1A	0.953
10589	DRAP1	51616	TAF9B	0.966
11186	RASSF1	83593	RASSF5	0.956
7419	VDAC3	6342	SCP2	0.952
7419	VDAC3	9218	VAPA	0.954
7419	VDAC3	9217	VAPB	0.95
7419	VDAC3	4701	NDUFA7	0.954
440	ASNS	2937	GSS	0.95
445	ASS1	1977	EIF4E	0.955
445	ASS1	5476	CTSA	0.967
445	ASS1	10072	DPP3	0.965
283237	TTC9C	7336	UBE2V2	0.957
283237	TTC9C	7335	UBE2V1	0.954
7706	TRIM25	10616	RBCK1	0.968
7706	TRIM25	51619	UBE2D4	0.95
3635	INPP5D	613	BCR	0.971

55577	NAGK	10944	C11orf58	0.964
7133	TNFRSF1B	10293	TRAIP	0.971
581	BAX	4170	MCL1	0.963
1436	CSF1R	6654	SOS1	0.973
11235	PDCD10	5198	PFAS	0.952
11235	PDCD10	55148	UBR7	0.95
55388	MCM10	4171	MCM2	0.95
51400	PPME1	10785	WDR4	0.95
23429	RYBP	54880	BCOR	0.964
9601	PDIA4	501	ALDH7A1	0.959
10944	C11orf58	9627	SNCAIP	0.952
10944	C11orf58	6611	SMS	0.979
79874	RABEP2	23607	CD2AP	0.95
10616	RBCK1	27338	UBE2S	0.963
10616	RBCK1	9099	USP2	0.958
2065	ERBB3	7535	ZAP70	0.966
2065	ERBB3	1399	CRKL	0.952
2065	ERBB3	3572	IL6ST	0.955
2067	ERCC1	6879	TAF7	0.964
2067	ERCC1	6878	TAF6	0.951
2067	ERCC1	84464	BTBD12	0.957
60626	RIC8A	80349	WDR61	0.967
467	ATF3	4097	MAFG	0.96
123169	LEO1	1788	DNMT3A	0.971
123169	LEO1	80349	WDR61	0.968
10134	BCAP31	79139	DERL1	0.955
10133	OPTN	10318	TNIP1	0.973
10138	YAF2	54880	BCOR	0.95
84262	PSMG3	10059	DNM1L	0.975
29950	SERTAD1	894	CCND2	0.968
5525	PPP2R5A	5527	PPP2R5C	0.972
5528	PPP2R5D	10095	ARPC1B	0.977
5528	PPP2R5D	5905	RANGAP1	0.952
51465	UBE2J1	55432	YOD1	0.971
51465	UBE2J1	79139	DERL1	0.971
51465	UBE2J1	84447	SYVN1	0.956
23624	CBLC	7535	ZAP70	0.966
23192	ATG4B	8408	ULK1	0.975
25936	NSL1	10403	NDC80	0.952
7511	XPNPEP1	55236	UBA6	0.959
55905	RNF114	10293	TRAIP	0.966
7994	MYST3	84289	ING5	0.952
7994	MYST3	11091	WDR5	0.972
79813	EHMT1	1788	DNMT3A	0.974
11267	SNF8	51028	VPS36	0.961
54880	BCOR	10336	PCGF3	0.966

10092	ARPC5	10094	ARPC3	0.95
28969	BZW2	5198	PFAS	0.954
7070	THY1	9997	SCO2	0.967
55759	WDR12	51028	VPS36	0.96
55759	WDR12	7251	TSG101	0.955
51616	TAF9B	6872	TAF1	0.962
10403	NDC80	9212	AURKB	0.951
23154	NCDN	5504	PPP1R2	0.969
84246	MED10	80306	MED28	0.96
29978	UBQLN2	10785	WDR4	0.96
23607	CD2AP	23325	KIAA1033	0.955
7681	MKRN3	7336	UBE2V2	0.973
369	ARAF	673	BRAF	0.96
369	ARAF	65125	WNK1	0.965
81608	FIP1L1	29894	CPSF1	0.953
4831	NME2	4830	NME1	0.976
5290	PIK3CA	9138	ARHGEF1	0.956
7336	UBE2V2	51569	UFM1	0.964
7335	UBE2V1	9296	ATP6V1F	0.955
10212	DDX39	6611	SMS	0.95
50626	CYHR1	10059	DNM1L	0.978
9997	SCO2	6342	SCP2	0.955
9997	SCO2	4701	NDUFA7	0.962
11034	DSTN	10802	SEC24A	0.953
11035	RIPK3	8772	FADD	0.96
51586	MED15	80306	MED28	0.962
26271	FBXO5	27338	UBE2S	0.967
26271	FBXO5	51529	ANAPC11	0.963
5906	RAP1A	6009	RHEB	0.951
10785	WDR4	64175	LEPRE1	0.957
10785	WDR4	10055	SAE1	0.962
2957	GTF2A1	6878	TAF6	0.951
2957	GTF2A1	6872	TAF1	0.966
2957	GTF2A1	26003	GORASP2	0.957
11075	STMN2	10802	SEC24A	0.967
8673	VAMP8	8773	SNAP23	0.964
8673	VAMP8	8775	NAPA	0.971
4091	SMAD6	11059	WWP1	0.965
4091	SMAD6	657	BMPR1A	0.957
25836	NIPBL	6672	SP100	0.967
6654	SOS1	613	BCR	0.986
10801	9-Sep	8801	SUCLG2	0.954
10802	SEC24A	10059	DNM1L	0.969
8795	TNFRSF10B	8797	TNFRSF10A	0.951
8797	TNFRSF10A	8772	FADD	0.963
5962	RDX	7430	EZR	0.95

5962	RDX	2935	GSPT1	0.98
9230	RAB11B	2665	GDI2	0.963
9232	PTTG1	51529	ANAPC11	0.969
10274	STAG1	10735	STAG2	0.951
8651	SOCS1	7410	VAV2	0.97
51699	VPS29	51028	VPS36	0.96
2107	ETF1	2935	GSPT1	0.967
54472	TOLLIP	3554	IL1R1	0.955
54472	TOLLIP	23543	RBM9	0.955
891	CCNB1	995	CDC25C	0.957
892	CCNC	80306	MED28	0.959
6879	TAF7	6878	TAF6	0.963
6879	TAF7	6872	TAF1	0.976
6878	TAF6	6873	TAF2	0.95
6873	TAF2	6872	TAF1	0.959
55432	YOD1	57159	TRIM54	0.966
989	7-Sep	6836	SURF4	0.968
57466	SFRS15	9217	VAPB	0.954
27338	UBE2S	51529	ANAPC11	0.962
9218	VAPA	9217	VAPB	0.971
9070	ASH2L	7391	USF1	0.951
10297	APC2	51529	ANAPC11	0.956
9877	ZC3H11A	4701	NDUFA7	0.963
11091	WDR5	80349	WDR61	0.959
